# Supplementary figures and images for: Aldolase-regulated G3BP1/2+ condensates control insulin mRNA storage in beta cells (part 1 of 4)
Source: EMBO J. 2025 May 12;44(13):3669–96. doi: 10.1038/s44318-025-00448-7 (PMC12216156; doi:10.1038/s44318-025-00448-7)

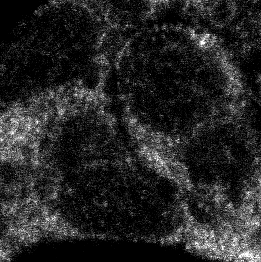

Supplement: Supplementary file 6 — Source data Fig. 1 [file 44318_2025_448_MOESM6_ESM.zip › Figure 1/Fig 1D/Composite.tif]

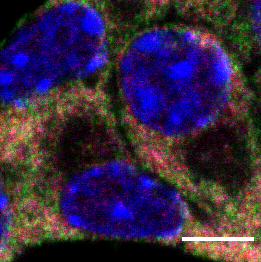

Supplement: Supplementary file 6 — Source data Fig. 1 [file 44318_2025_448_MOESM6_ESM.zip › Figure 1/Fig 1D/Composite.tif (RGB).tif]

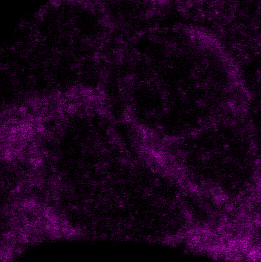

Supplement: Supplementary file 6 — Source data Fig. 1 [file 44318_2025_448_MOESM6_ESM.zip › Figure 1/Fig 1D/G3BP2.tif]

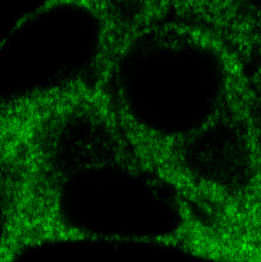

Supplement: Supplementary file 6 — Source data Fig. 1 [file 44318_2025_448_MOESM6_ESM.zip › Figure 1/Fig 1D/G3BP1.tif]

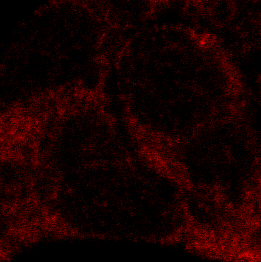

Supplement: Supplementary file 6 — Source data Fig. 1 [file 44318_2025_448_MOESM6_ESM.zip › Figure 1/Fig 1D/EIF3B.tif]

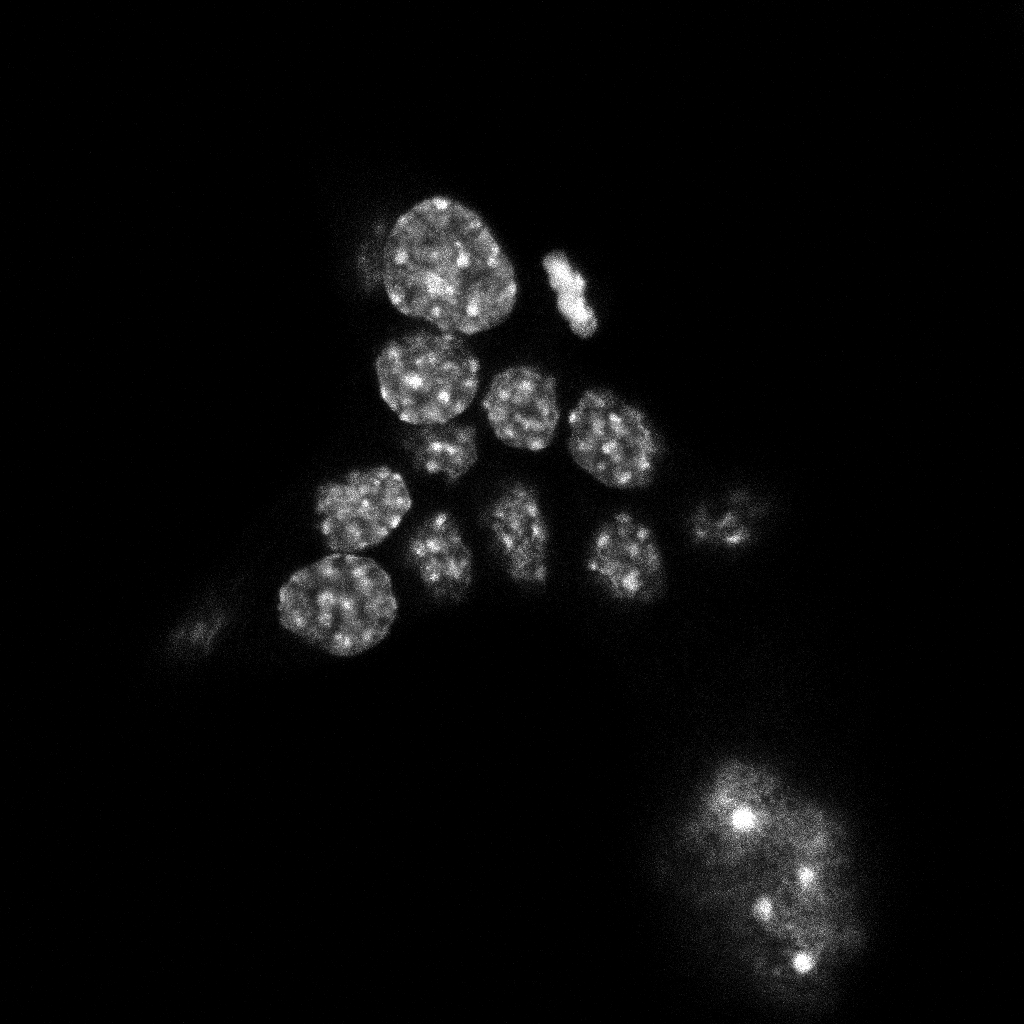

Supplement: Supplementary file 6 — Source data Fig. 1 [file 44318_2025_448_MOESM6_ESM.zip › Figure 1/Fig 1C/Complete image Fig 1C.tif]

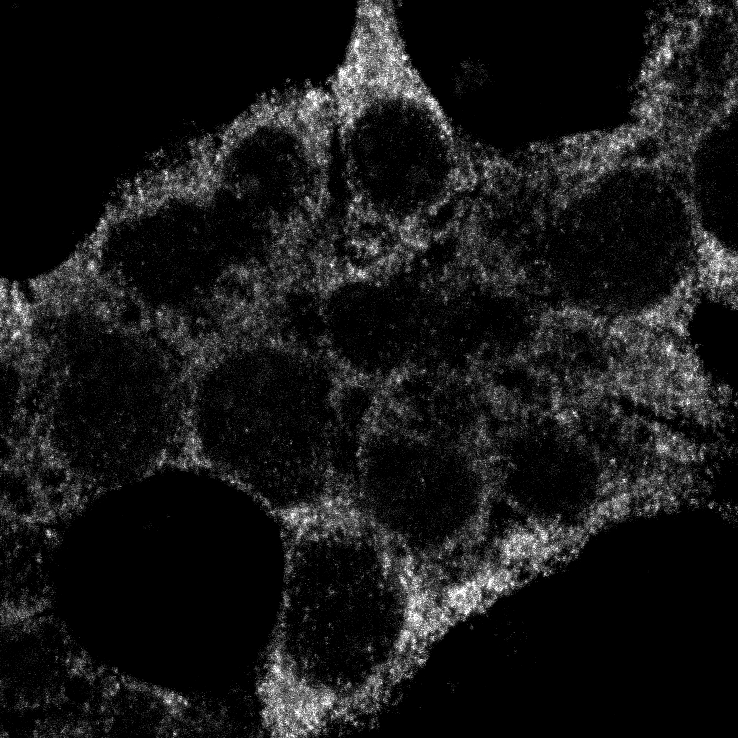

Supplement: Supplementary file 6 — Source data Fig. 1 [file 44318_2025_448_MOESM6_ESM.zip › Figure 1/Fig 1F/G3BP1-INS1-2 mRNA 16-7/Fig 1F G3BP1-eIf3b-Ins1-2mRNA complete image.tif]

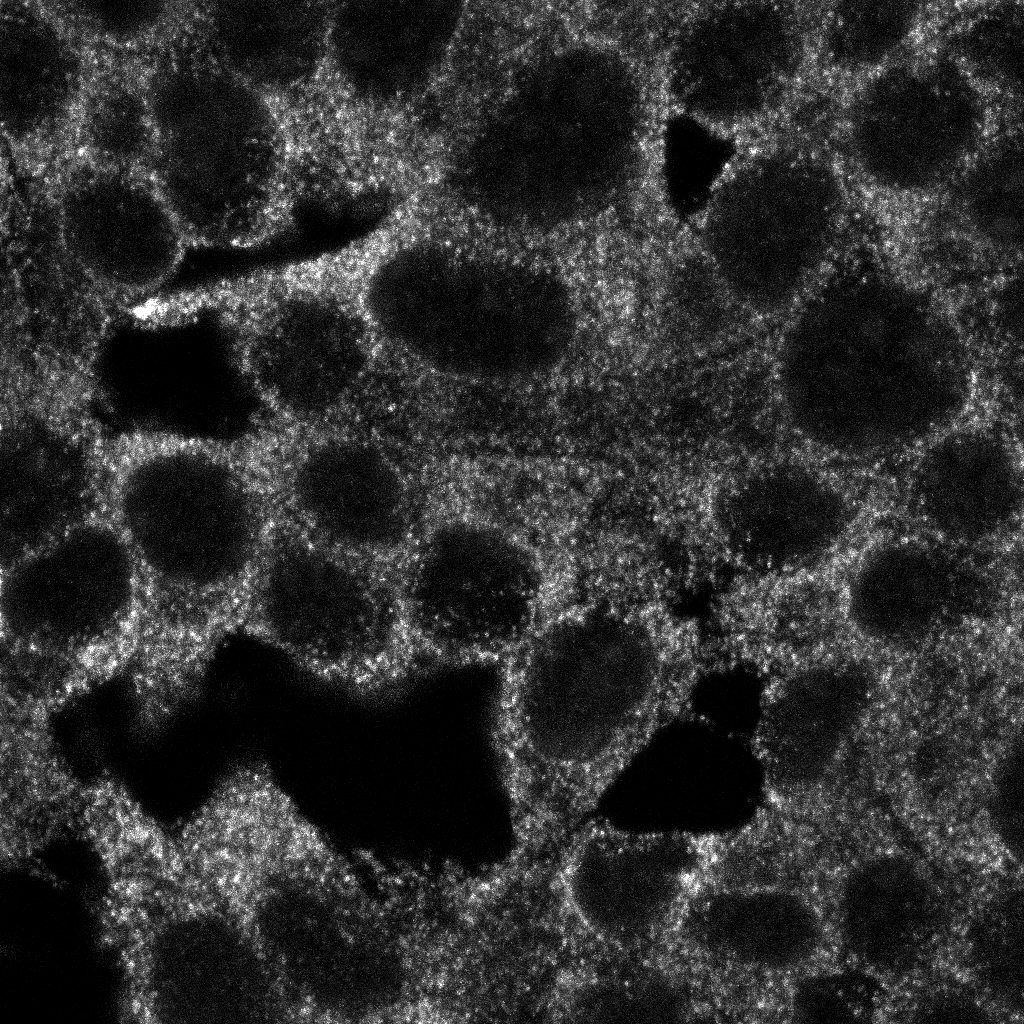

Supplement: Supplementary file 6 — Source data Fig. 1 [file 44318_2025_448_MOESM6_ESM.zip › Figure 1/Fig 1F/G3BP2-INS1-2 mRNA 16.7/Composite.tif]

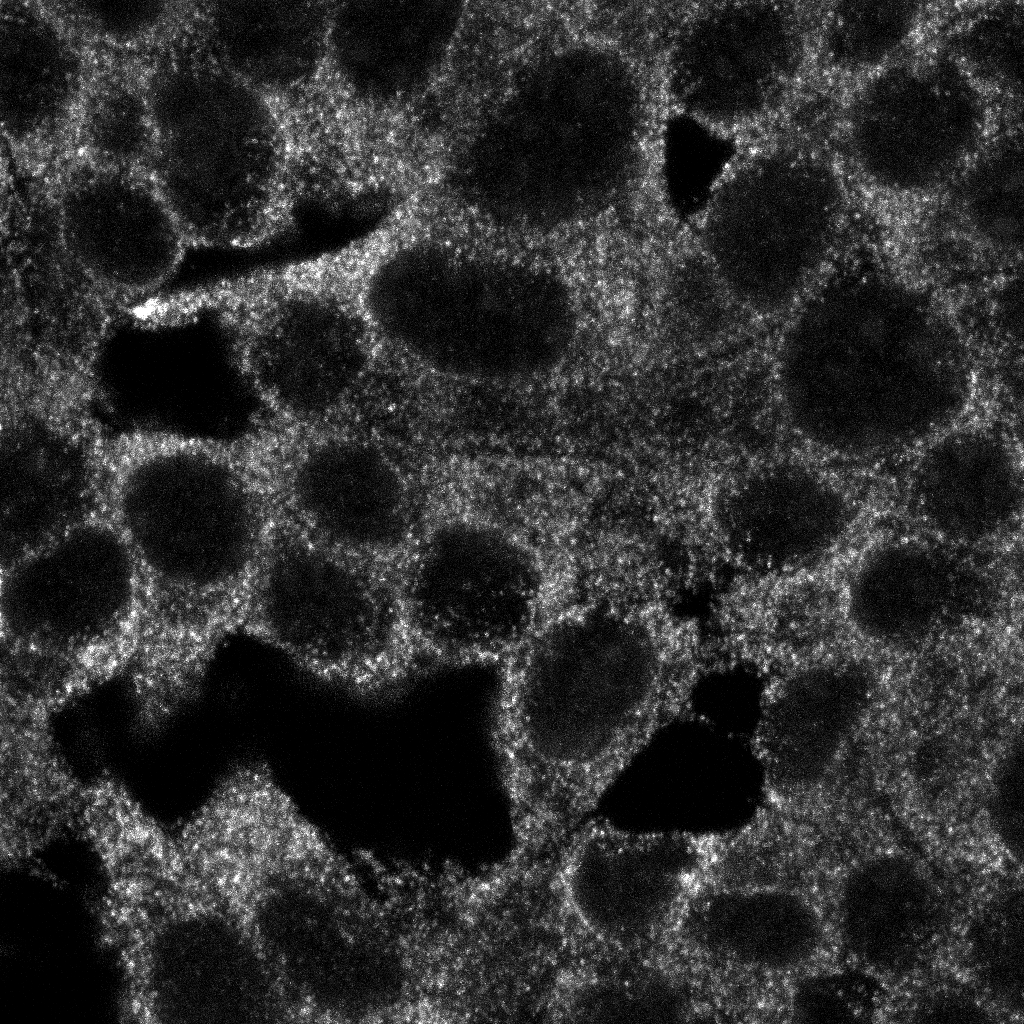

Supplement: Supplementary file 6 — Source data Fig. 1 [file 44318_2025_448_MOESM6_ESM.zip › Figure 1/Fig 1F/G3BP2-INS1-2 mRNA 16.7/Fig 1F G3BP2-eIf3b-Ins1-2mRNA complete image.tif]

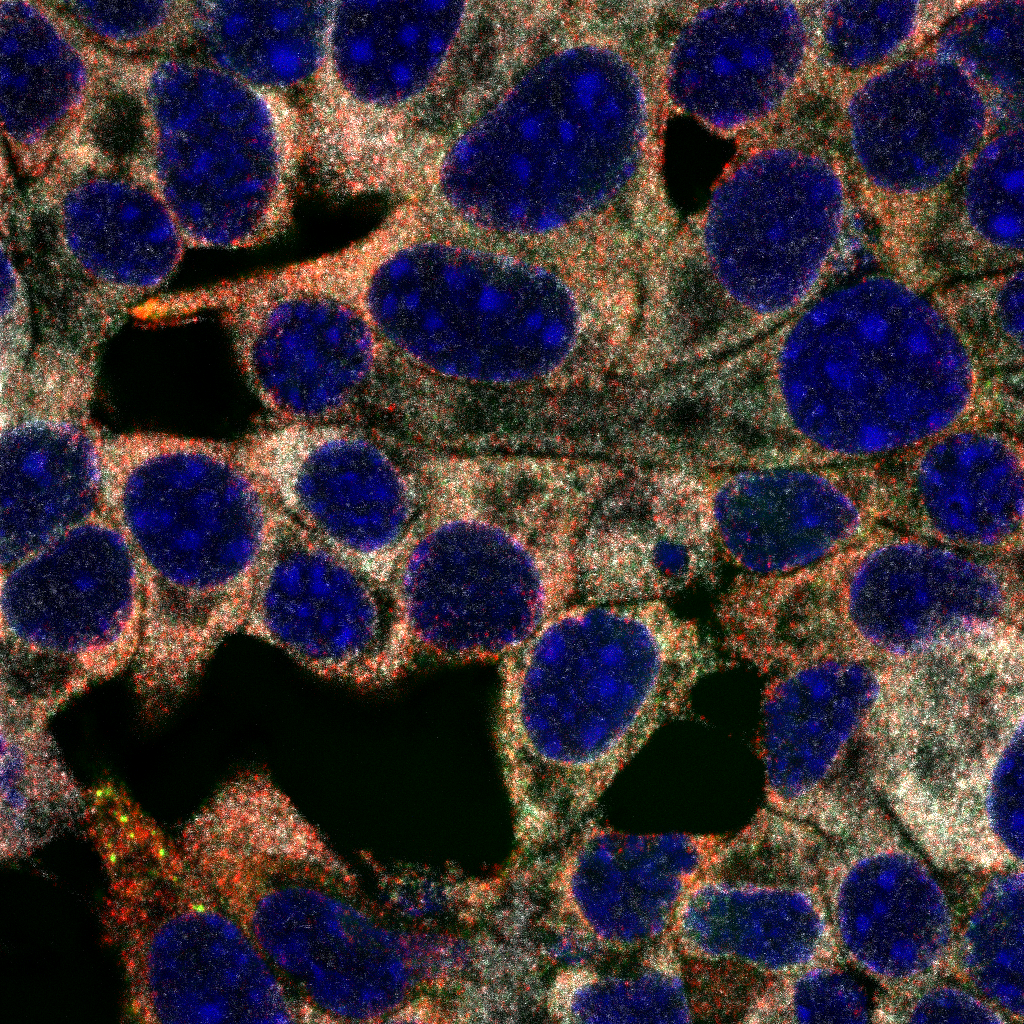

Supplement: Supplementary file 6 — Source data Fig. 1 [file 44318_2025_448_MOESM6_ESM.zip › Figure 1/Fig 1F/G3BP2-INS1-2 mRNA 16.7/Composite2.tif (RGB).tif]

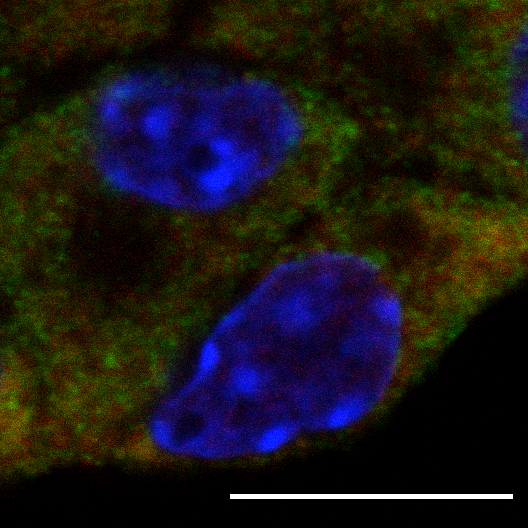

Supplement: Supplementary file 6 — Source data Fig. 1 [file 44318_2025_448_MOESM6_ESM.zip › Figure 1/Fig 1F/lacZ mRNA 16.7/Composite.tif (RGB).tif]

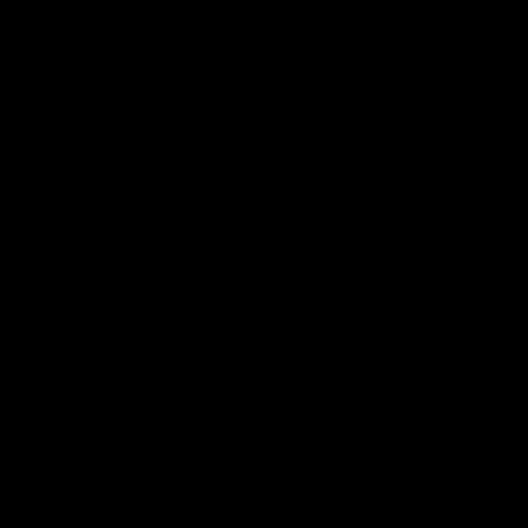

Supplement: Supplementary file 6 — Source data Fig. 1 [file 44318_2025_448_MOESM6_ESM.zip › Figure 1/Fig 1F/lacZ mRNA 16.7/lacz mrna.tif]

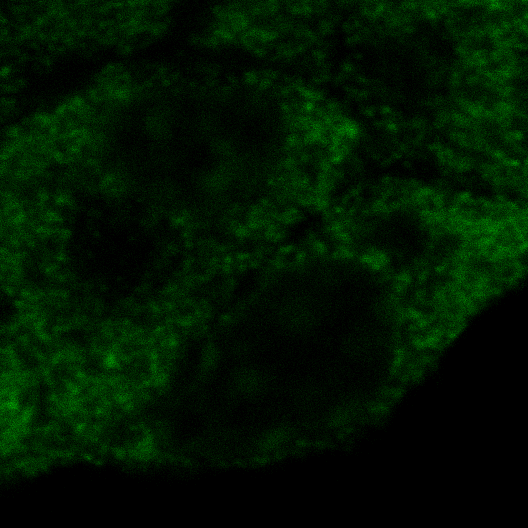

Supplement: Supplementary file 6 — Source data Fig. 1 [file 44318_2025_448_MOESM6_ESM.zip › Figure 1/Fig 1F/lacZ mRNA 16.7/G3BP1.tif]

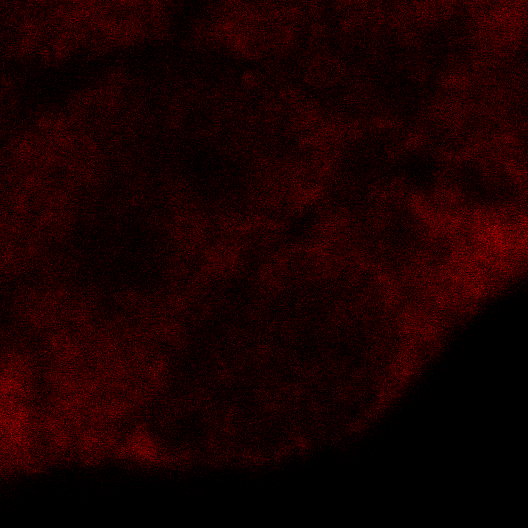

Supplement: Supplementary file 6 — Source data Fig. 1 [file 44318_2025_448_MOESM6_ESM.zip › Figure 1/Fig 1F/lacZ mRNA 16.7/EIF3B.tif]

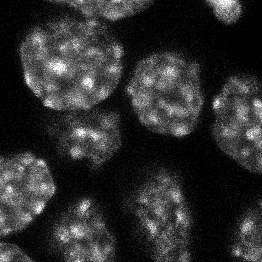

Supplement: Supplementary file 6 — Source data Fig. 1 [file 44318_2025_448_MOESM6_ESM.zip › Figure 1/Fig 1C/crop 18,07 (Fig 1C)/composite.tif]

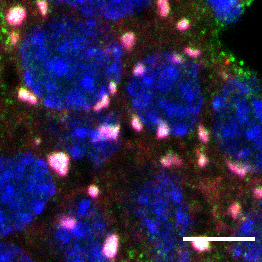

Supplement: Supplementary file 6 — Source data Fig. 1 [file 44318_2025_448_MOESM6_ESM.zip › Figure 1/Fig 1C/crop 18,07 (Fig 1C)/composite.tif (RGB).tif]

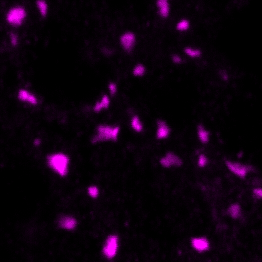

Supplement: Supplementary file 6 — Source data Fig. 1 [file 44318_2025_448_MOESM6_ESM.zip › Figure 1/Fig 1C/crop 18,07 (Fig 1C)/G3BP2.tif]

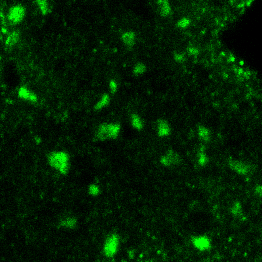

Supplement: Supplementary file 6 — Source data Fig. 1 [file 44318_2025_448_MOESM6_ESM.zip › Figure 1/Fig 1C/crop 18,07 (Fig 1C)/G3BP1.tif]

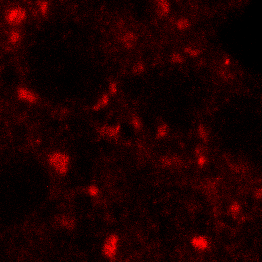

Supplement: Supplementary file 6 — Source data Fig. 1 [file 44318_2025_448_MOESM6_ESM.zip › Figure 1/Fig 1C/crop 18,07 (Fig 1C)/EIF3B.tif]

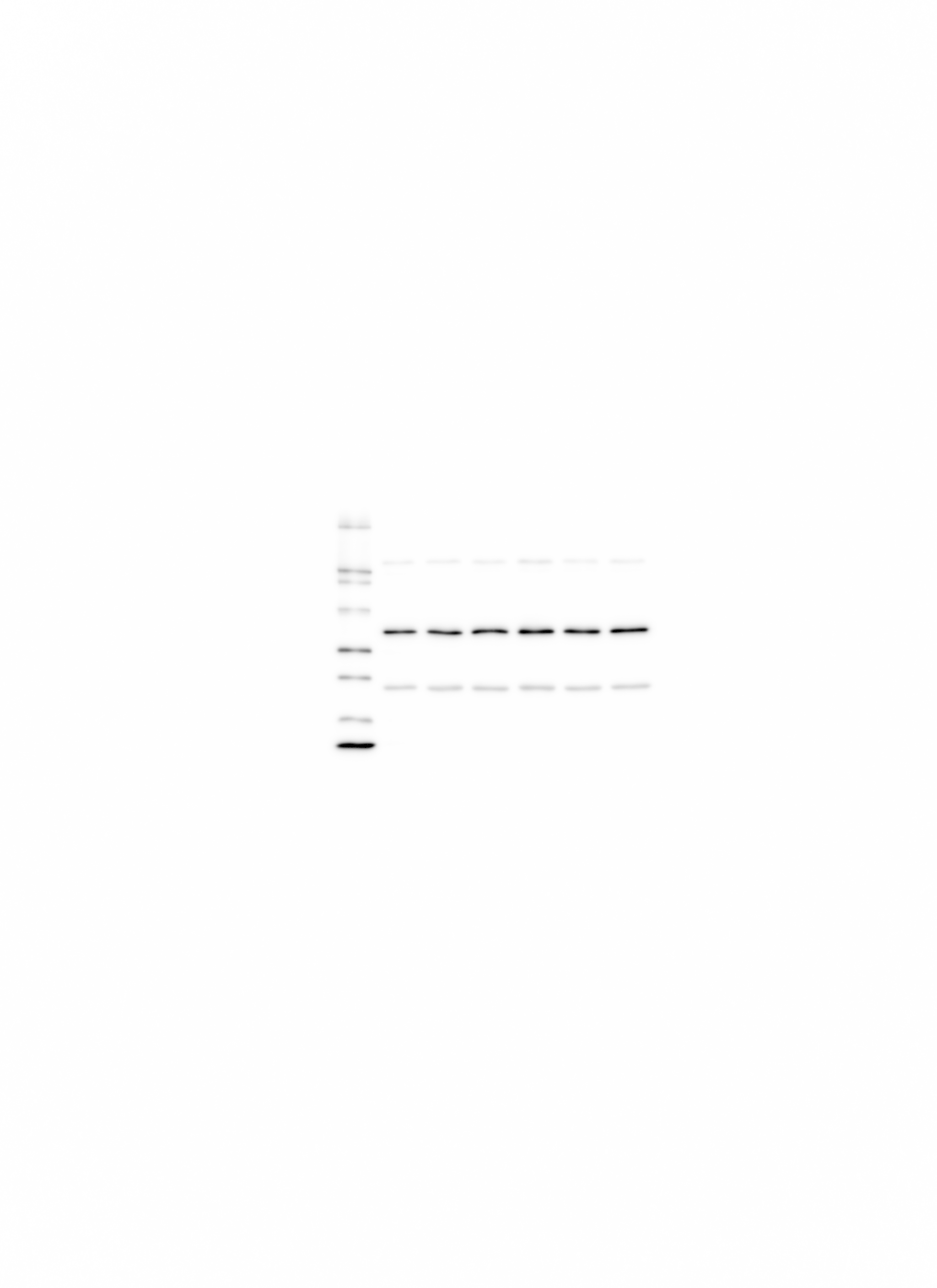

Supplement: Supplementary file 6 — Source data Fig. 1 [file 44318_2025_448_MOESM6_ESM.zip › Figure 1/Fig 1B/G3BP1/(G3BP1 crop) 04.04.23 G3BP1 n1 K8 Ars-2.8 gtub1 2023.04.04_09.47.01_Ch.tif]

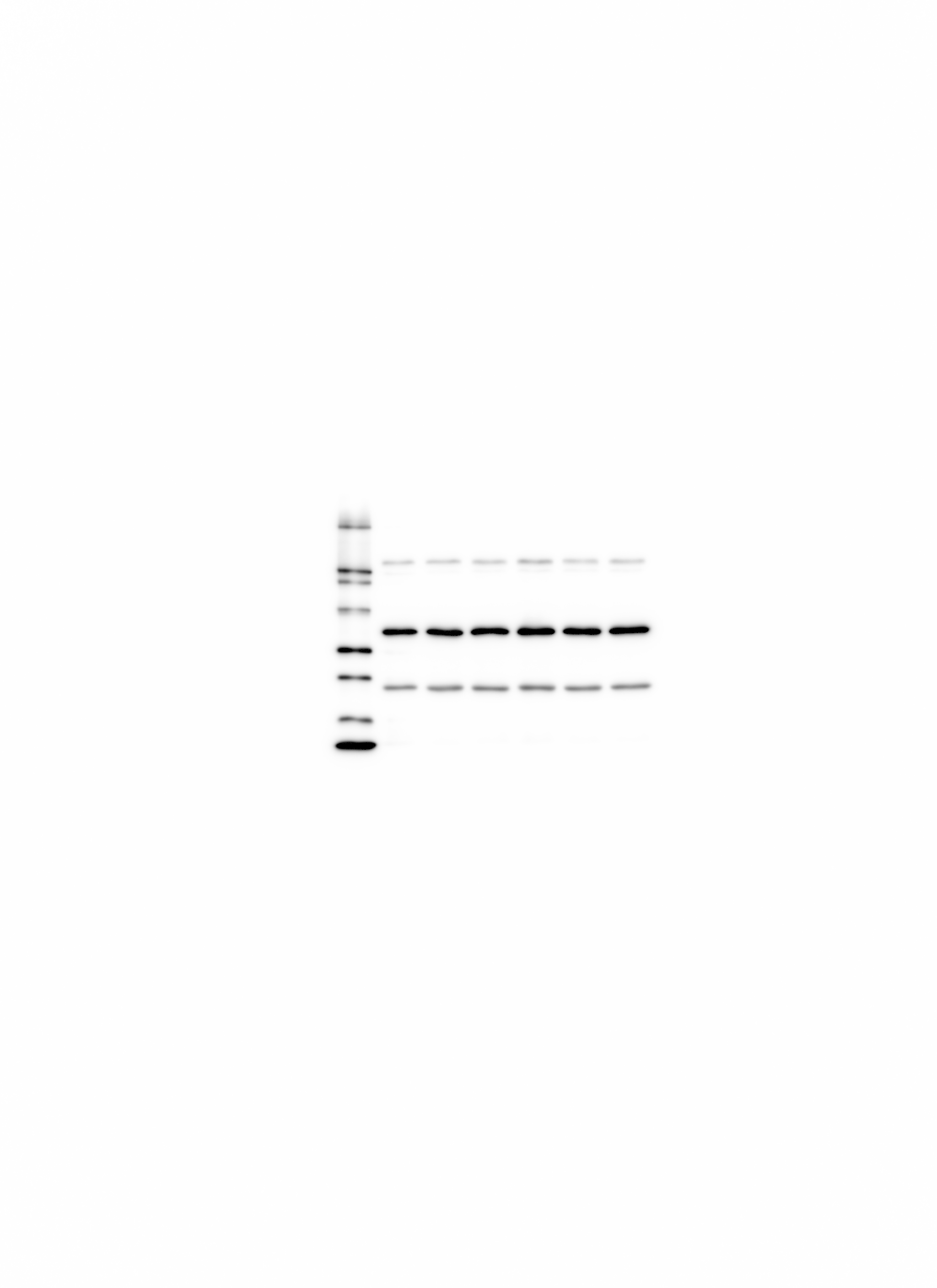

Supplement: Supplementary file 6 — Source data Fig. 1 [file 44318_2025_448_MOESM6_ESM.zip › Figure 1/Fig 1B/G3BP1/(Tubulin crop) 04.04.23 G3BP1 n1 K8 Ars-2.8 gtub2 2023.04.04_09.49.05_Ch.tif]

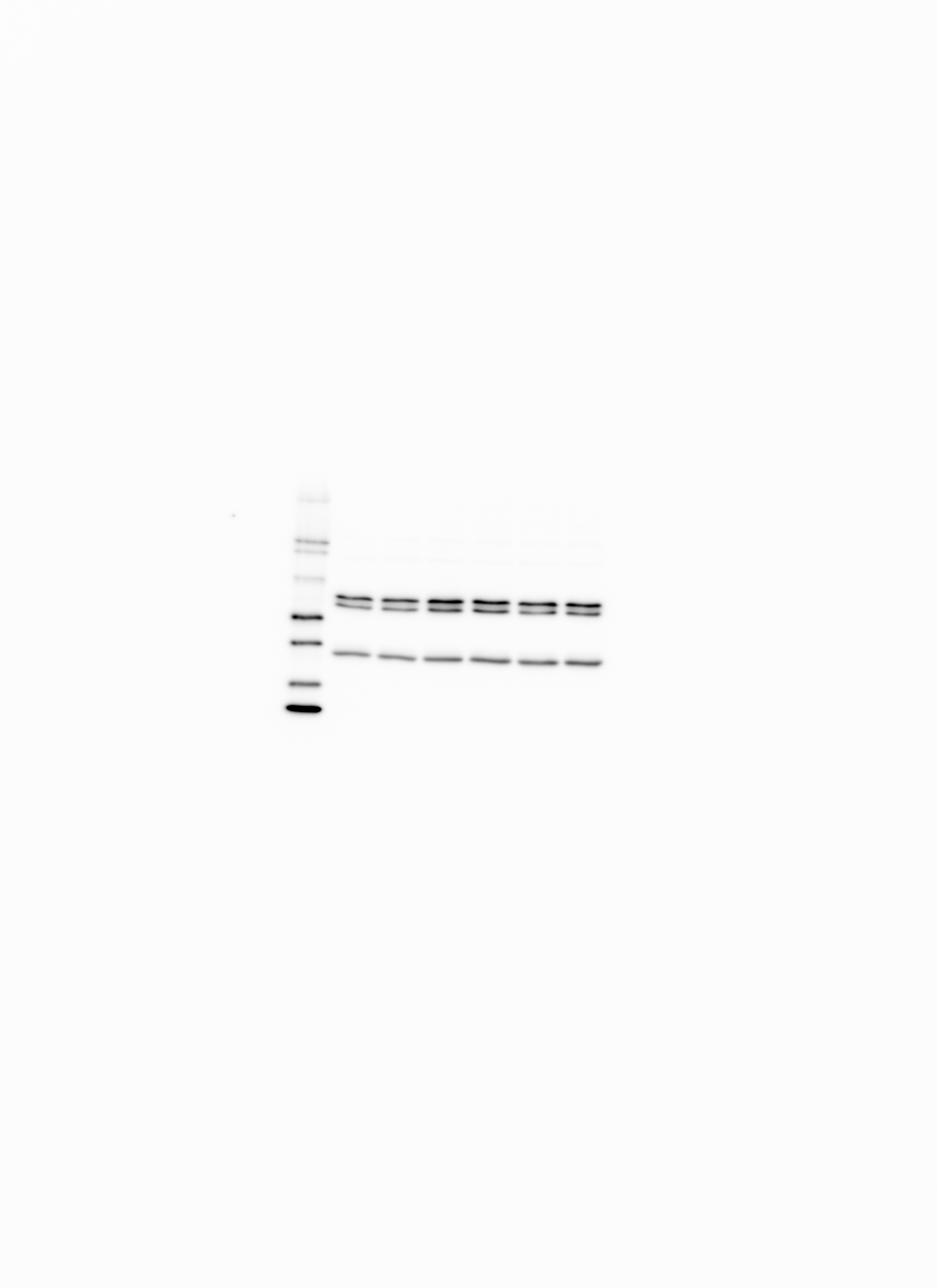

Supplement: Supplementary file 6 — Source data Fig. 1 [file 44318_2025_448_MOESM6_ESM.zip › Figure 1/Fig 1B/G3BP2/(Tubulin not included in figure) 04.04.23 G3BP2 n1 K8 Ars-2.8 gtub 2023.04.04_09.59.26_Ch2.tif]

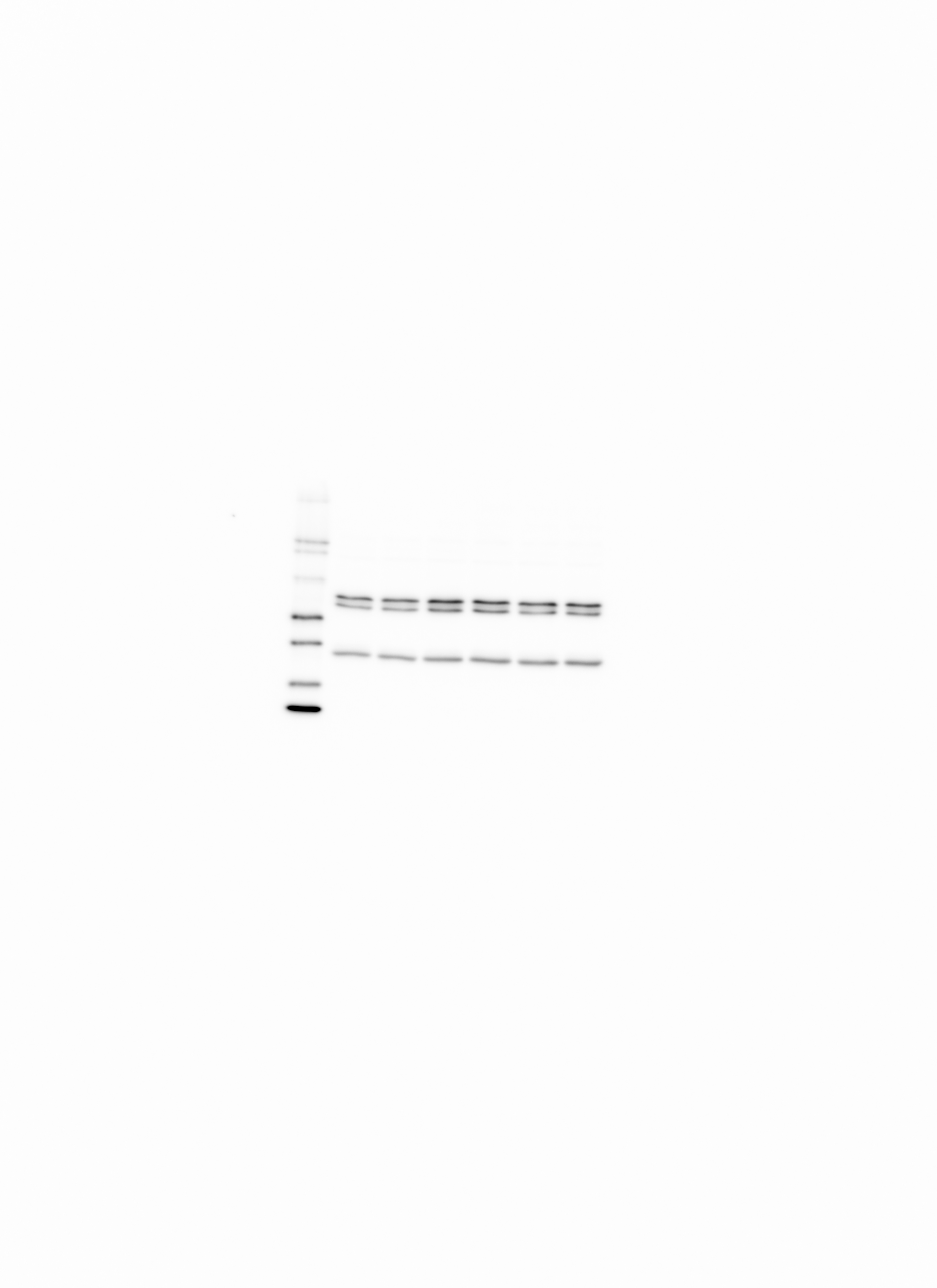

Supplement: Supplementary file 6 — Source data Fig. 1 [file 44318_2025_448_MOESM6_ESM.zip › Figure 1/Fig 1B/G3BP2/(G3BP2 crop) 04.04.23 G3BP2 n1 K8 Ars-2.8 gtub 2023.04.04_09.59.26_Ch.tif]

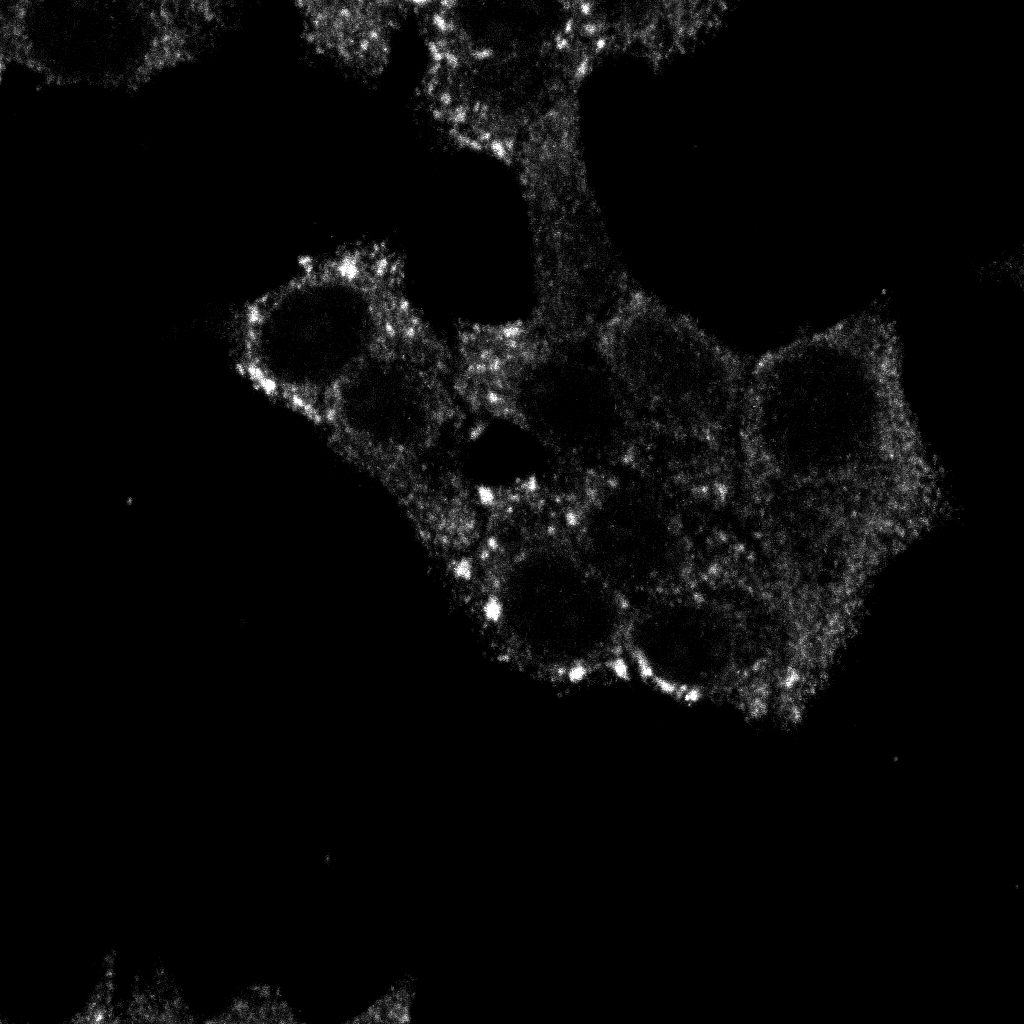

Supplement: Supplementary file 6 — Source data Fig. 1 [file 44318_2025_448_MOESM6_ESM.zip › Figure 1/Fig 1E/G3BP1-INS1-2 mRNA 2.8/Fig 1E G3BP1-eIf3b-Ins1-2mRNA complete image.tif]

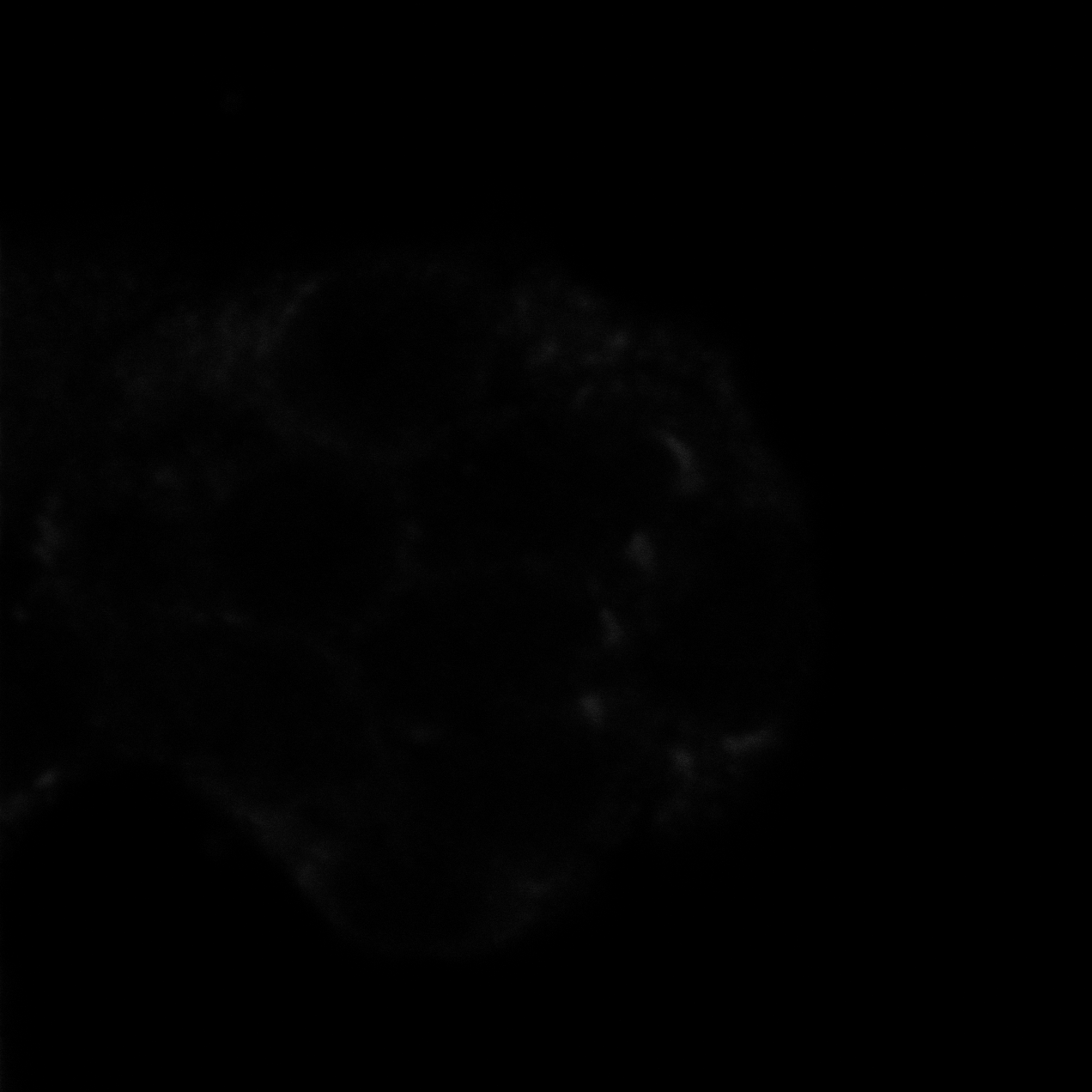

Supplement: Supplementary file 6 — Source data Fig. 1 [file 44318_2025_448_MOESM6_ESM.zip › Figure 1/Fig 1E/lacZ mRNA 2.8/composite.tif]

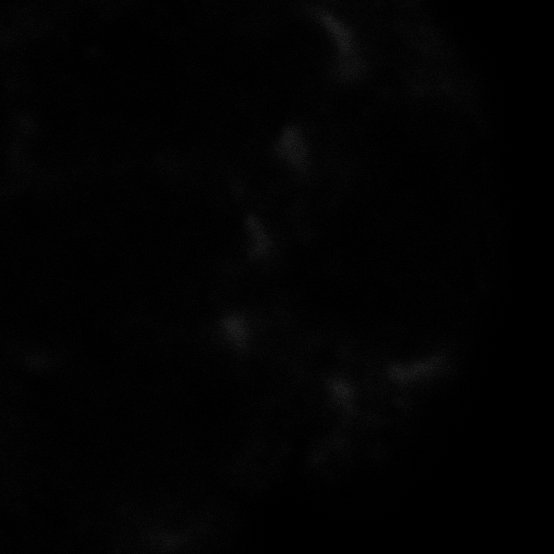

Supplement: Supplementary file 6 — Source data Fig. 1 [file 44318_2025_448_MOESM6_ESM.zip › Figure 1/Fig 1E/lacZ mRNA 2.8/composite crop.tif]

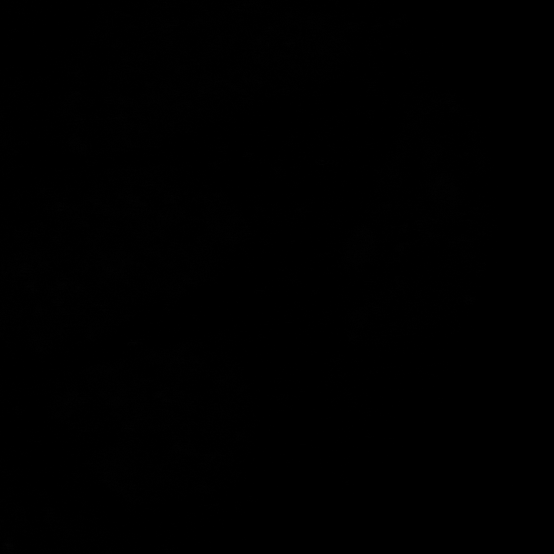

Supplement: Supplementary file 6 — Source data Fig. 1 [file 44318_2025_448_MOESM6_ESM.zip › Figure 1/Fig 1E/lacZ mRNA 2.8/lacz mrna.tif]

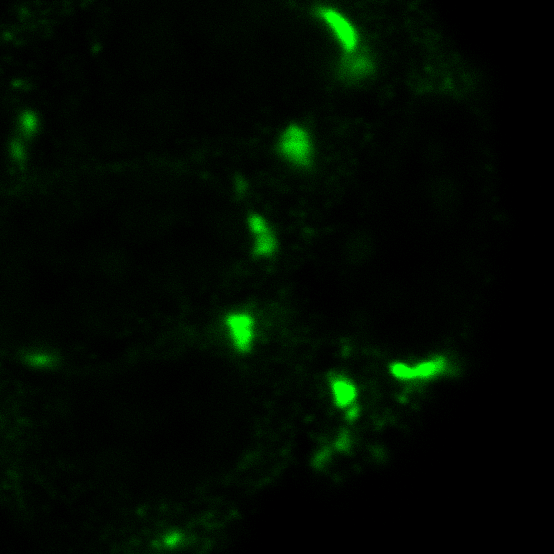

Supplement: Supplementary file 6 — Source data Fig. 1 [file 44318_2025_448_MOESM6_ESM.zip › Figure 1/Fig 1E/lacZ mRNA 2.8/G3BP1.tif]

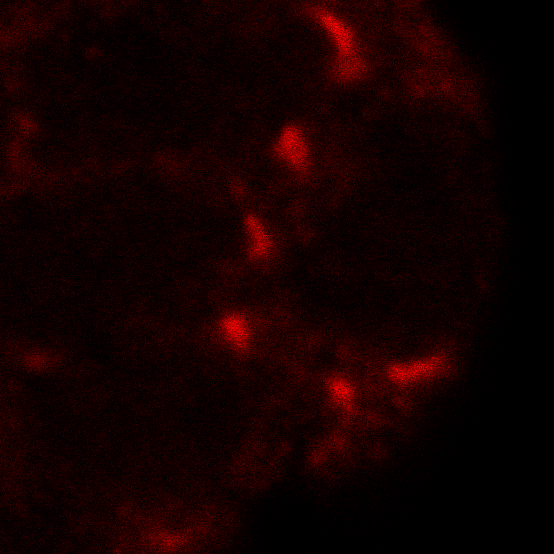

Supplement: Supplementary file 6 — Source data Fig. 1 [file 44318_2025_448_MOESM6_ESM.zip › Figure 1/Fig 1E/lacZ mRNA 2.8/EIF3B.tif]

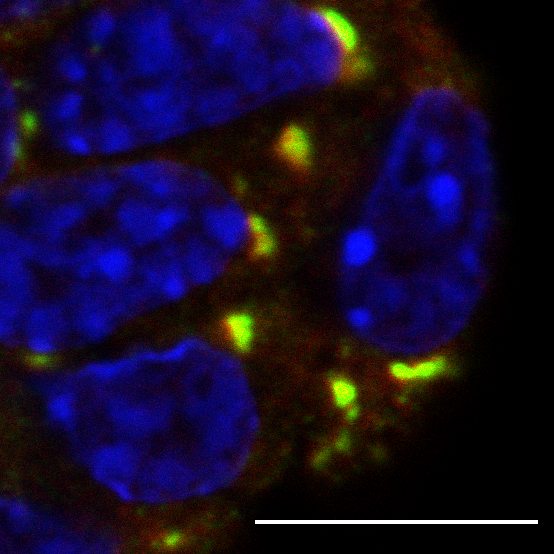

Supplement: Supplementary file 6 — Source data Fig. 1 [file 44318_2025_448_MOESM6_ESM.zip › Figure 1/Fig 1E/lacZ mRNA 2.8/composite crop.tif (RGB).tif]

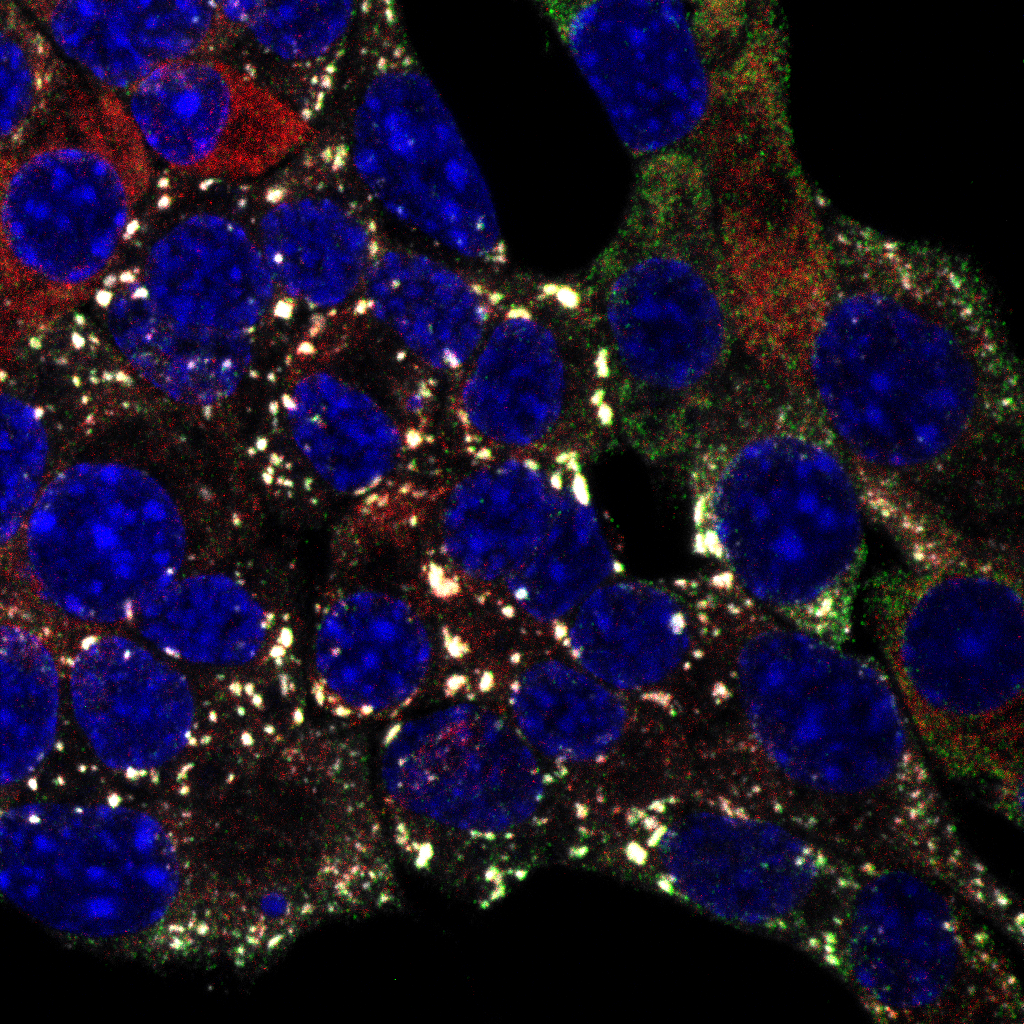

Supplement: Supplementary file 6 — Source data Fig. 1 [file 44318_2025_448_MOESM6_ESM.zip › Figure 1/Fig 1E/G3BP2-INS1-2 mRNA 2.8/Composite-1.tif (RGB).tif]

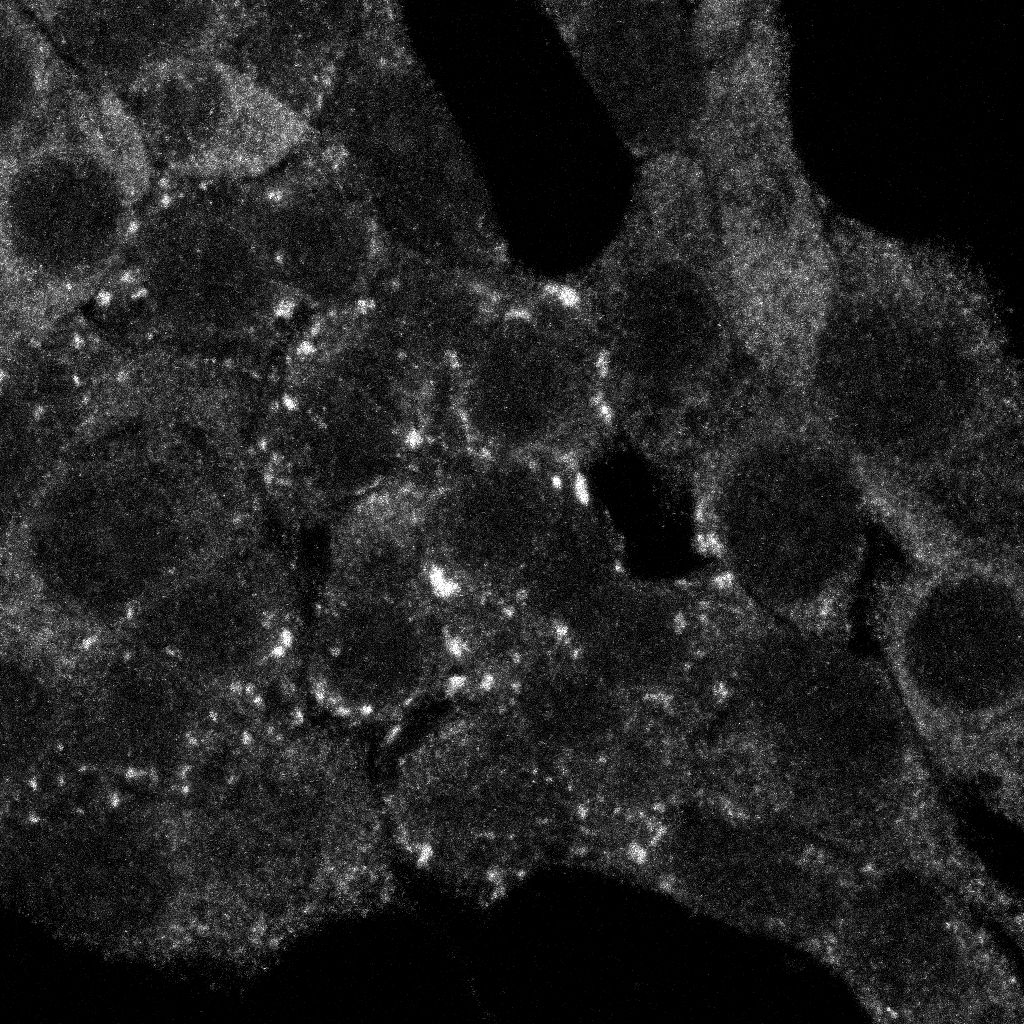

Supplement: Supplementary file 6 — Source data Fig. 1 [file 44318_2025_448_MOESM6_ESM.zip › Figure 1/Fig 1E/G3BP2-INS1-2 mRNA 2.8/Fig 1E G3BP2-eIf3b-Ins1-2mRNA complete image.tif]

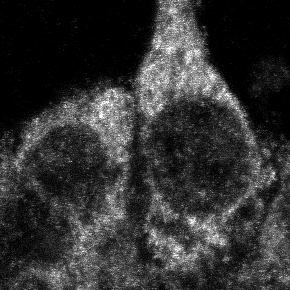

Supplement: Supplementary file 6 — Source data Fig. 1 [file 44318_2025_448_MOESM6_ESM.zip › Figure 1/Fig 1F/G3BP1-INS1-2 mRNA 16-7/crop of Fig 1F G3BP1-eIf3b-Ins1-2mRNA complete image/INS1 MRNA.tif]

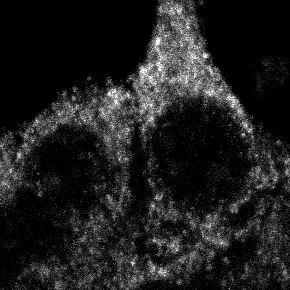

Supplement: Supplementary file 6 — Source data Fig. 1 [file 44318_2025_448_MOESM6_ESM.zip › Figure 1/Fig 1F/G3BP1-INS1-2 mRNA 16-7/crop of Fig 1F G3BP1-eIf3b-Ins1-2mRNA complete image/Composite.tif]

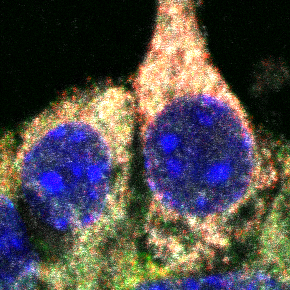

Supplement: Supplementary file 6 — Source data Fig. 1 [file 44318_2025_448_MOESM6_ESM.zip › Figure 1/Fig 1F/G3BP1-INS1-2 mRNA 16-7/crop of Fig 1F G3BP1-eIf3b-Ins1-2mRNA complete image/Composite.tif (RGB).tif]

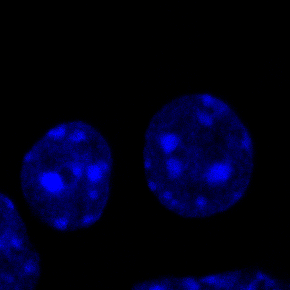

Supplement: Supplementary file 6 — Source data Fig. 1 [file 44318_2025_448_MOESM6_ESM.zip › Figure 1/Fig 1F/G3BP1-INS1-2 mRNA 16-7/crop of Fig 1F G3BP1-eIf3b-Ins1-2mRNA complete image/DAPI.tif]

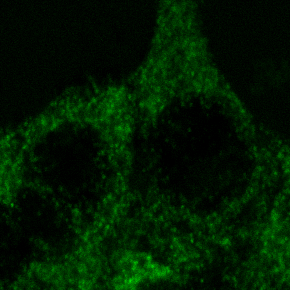

Supplement: Supplementary file 6 — Source data Fig. 1 [file 44318_2025_448_MOESM6_ESM.zip › Figure 1/Fig 1F/G3BP1-INS1-2 mRNA 16-7/crop of Fig 1F G3BP1-eIf3b-Ins1-2mRNA complete image/G3BP1.tif]

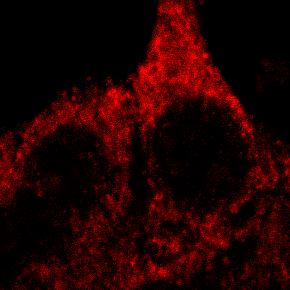

Supplement: Supplementary file 6 — Source data Fig. 1 [file 44318_2025_448_MOESM6_ESM.zip › Figure 1/Fig 1F/G3BP1-INS1-2 mRNA 16-7/crop of Fig 1F G3BP1-eIf3b-Ins1-2mRNA complete image/EIF3B.tif]

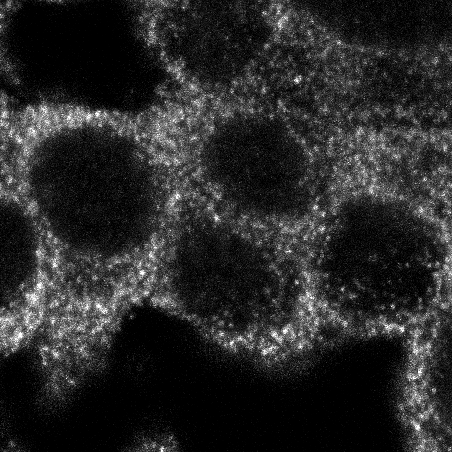

Supplement: Supplementary file 6 — Source data Fig. 1 [file 44318_2025_448_MOESM6_ESM.zip › Figure 1/Fig 1F/G3BP2-INS1-2 mRNA 16.7/Crop of Fig 1F G3BP2-eIf3b-Ins1-2mRNA complete image/Composite2.tif]

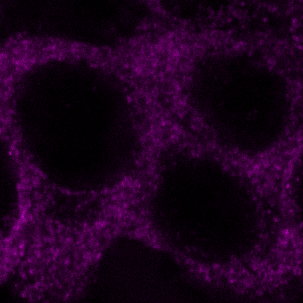

Supplement: Supplementary file 6 — Source data Fig. 1 [file 44318_2025_448_MOESM6_ESM.zip › Figure 1/Fig 1F/G3BP2-INS1-2 mRNA 16.7/Crop of Fig 1F G3BP2-eIf3b-Ins1-2mRNA complete image/G3BP2.tif]

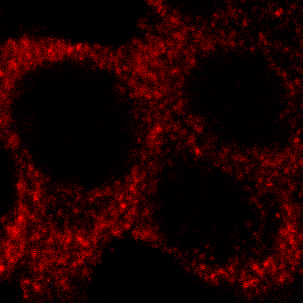

Supplement: Supplementary file 6 — Source data Fig. 1 [file 44318_2025_448_MOESM6_ESM.zip › Figure 1/Fig 1F/G3BP2-INS1-2 mRNA 16.7/Crop of Fig 1F G3BP2-eIf3b-Ins1-2mRNA complete image/EIF3B.tif]

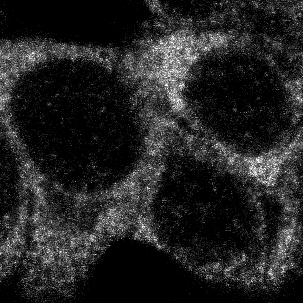

Supplement: Supplementary file 6 — Source data Fig. 1 [file 44318_2025_448_MOESM6_ESM.zip › Figure 1/Fig 1F/G3BP2-INS1-2 mRNA 16.7/Crop of Fig 1F G3BP2-eIf3b-Ins1-2mRNA complete image/INS MRNA.tif]

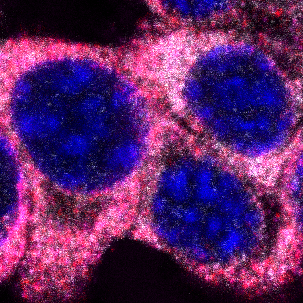

Supplement: Supplementary file 6 — Source data Fig. 1 [file 44318_2025_448_MOESM6_ESM.zip › Figure 1/Fig 1F/G3BP2-INS1-2 mRNA 16.7/Crop of Fig 1F G3BP2-eIf3b-Ins1-2mRNA complete image/Composite2.tif (RGB).tif]

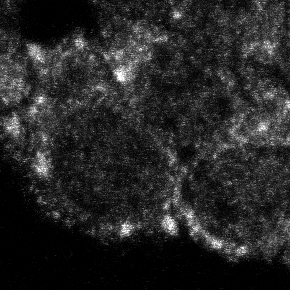

Supplement: Supplementary file 6 — Source data Fig. 1 [file 44318_2025_448_MOESM6_ESM.zip › Figure 1/Fig 1E/G3BP1-INS1-2 mRNA 2.8/crop of Fig 1E G3BP1-eIf3b-Ins1-2mRNA complete image/INS1 MRNA.tif]

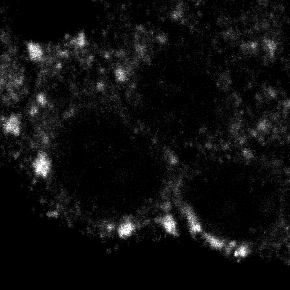

Supplement: Supplementary file 6 — Source data Fig. 1 [file 44318_2025_448_MOESM6_ESM.zip › Figure 1/Fig 1E/G3BP1-INS1-2 mRNA 2.8/crop of Fig 1E G3BP1-eIf3b-Ins1-2mRNA complete image/Composite CROP 20,01 .tif]

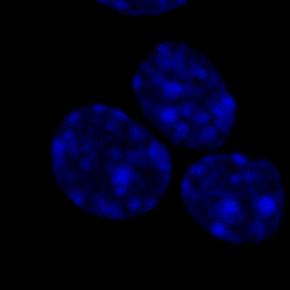

Supplement: Supplementary file 6 — Source data Fig. 1 [file 44318_2025_448_MOESM6_ESM.zip › Figure 1/Fig 1E/G3BP1-INS1-2 mRNA 2.8/crop of Fig 1E G3BP1-eIf3b-Ins1-2mRNA complete image/dapi.tif]

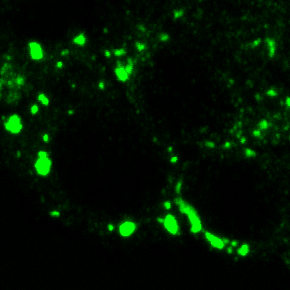

Supplement: Supplementary file 6 — Source data Fig. 1 [file 44318_2025_448_MOESM6_ESM.zip › Figure 1/Fig 1E/G3BP1-INS1-2 mRNA 2.8/crop of Fig 1E G3BP1-eIf3b-Ins1-2mRNA complete image/g3bp1.tif]

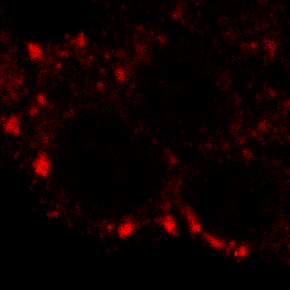

Supplement: Supplementary file 6 — Source data Fig. 1 [file 44318_2025_448_MOESM6_ESM.zip › Figure 1/Fig 1E/G3BP1-INS1-2 mRNA 2.8/crop of Fig 1E G3BP1-eIf3b-Ins1-2mRNA complete image/eif3b.tif]

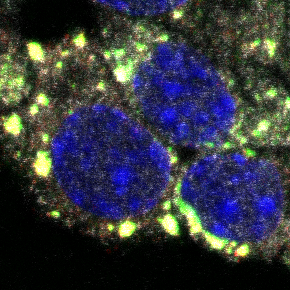

Supplement: Supplementary file 6 — Source data Fig. 1 [file 44318_2025_448_MOESM6_ESM.zip › Figure 1/Fig 1E/G3BP1-INS1-2 mRNA 2.8/crop of Fig 1E G3BP1-eIf3b-Ins1-2mRNA complete image/Composite (RGB).tif]

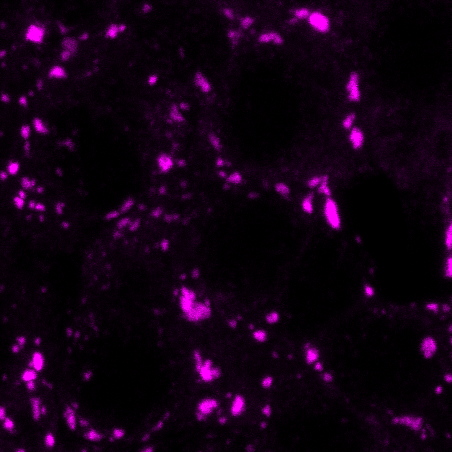

Supplement: Supplementary file 6 — Source data Fig. 1 [file 44318_2025_448_MOESM6_ESM.zip › Figure 1/Fig 1E/G3BP2-INS1-2 mRNA 2.8/Crop of Fig 1E G3BP2-eIf3b-Ins1-2mRNA complete image/G3BP2.tif]

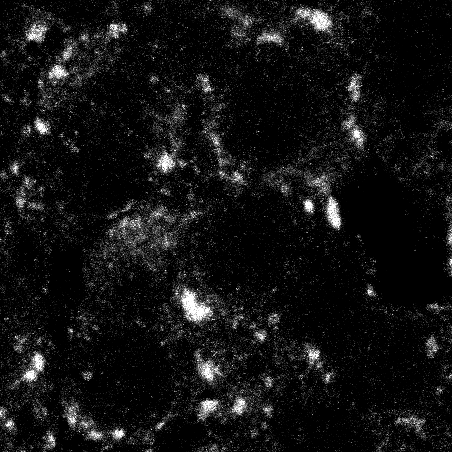

Supplement: Supplementary file 6 — Source data Fig. 1 [file 44318_2025_448_MOESM6_ESM.zip › Figure 1/Fig 1E/G3BP2-INS1-2 mRNA 2.8/Crop of Fig 1E G3BP2-eIf3b-Ins1-2mRNA complete image/INS2 MRNA.tif]

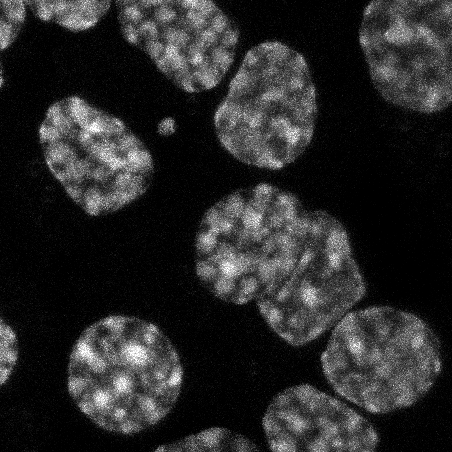

Supplement: Supplementary file 6 — Source data Fig. 1 [file 44318_2025_448_MOESM6_ESM.zip › Figure 1/Fig 1E/G3BP2-INS1-2 mRNA 2.8/Crop of Fig 1E G3BP2-eIf3b-Ins1-2mRNA complete image/DAPI.tif]

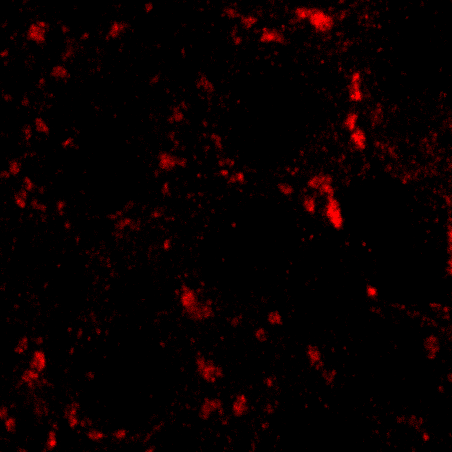

Supplement: Supplementary file 6 — Source data Fig. 1 [file 44318_2025_448_MOESM6_ESM.zip › Figure 1/Fig 1E/G3BP2-INS1-2 mRNA 2.8/Crop of Fig 1E G3BP2-eIf3b-Ins1-2mRNA complete image/EIF3B.tif]

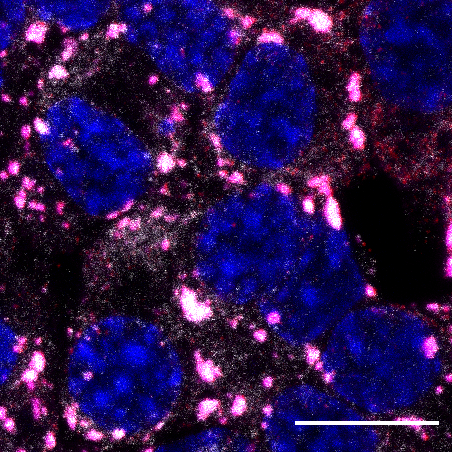

Supplement: Supplementary file 6 — Source data Fig. 1 [file 44318_2025_448_MOESM6_ESM.zip › Figure 1/Fig 1E/G3BP2-INS1-2 mRNA 2.8/Crop of Fig 1E G3BP2-eIf3b-Ins1-2mRNA complete image/Composite-1.tif (RGB).tif]

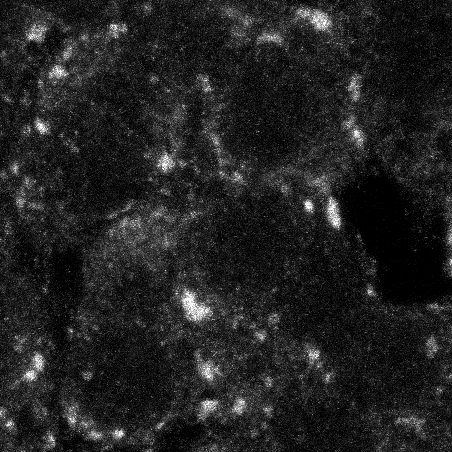

Supplement: Supplementary file 6 — Source data Fig. 1 [file 44318_2025_448_MOESM6_ESM.zip › Figure 1/Fig 1E/G3BP2-INS1-2 mRNA 2.8/Crop of Fig 1E G3BP2-eIf3b-Ins1-2mRNA complete image/Composite-1.tif]

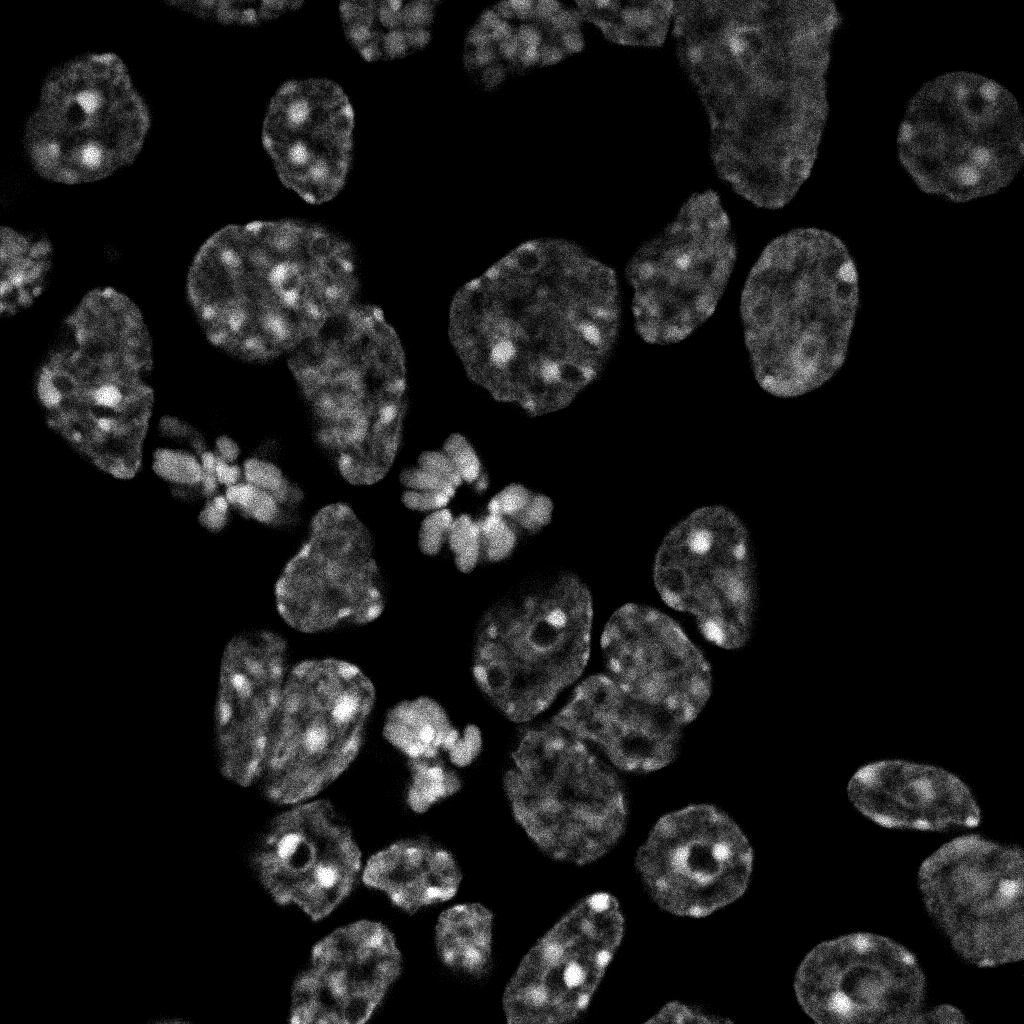

Supplement: Supplementary file 7 — Source data Fig. 2 [file 44318_2025_448_MOESM7_ESM.zip › Figure 2/Fig 2A/16.7/Fig 2A complete Image 16.7.tif]

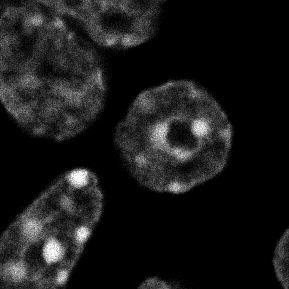

Supplement: Supplementary file 7 — Source data Fig. 2 [file 44318_2025_448_MOESM7_ESM.zip › Figure 2/Fig 2A/16.7/crop of Fig 2A complete Image 16.7/composite-1.tif]

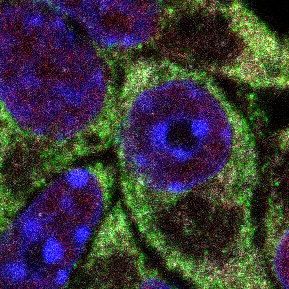

Supplement: Supplementary file 7 — Source data Fig. 2 [file 44318_2025_448_MOESM7_ESM.zip › Figure 2/Fig 2A/16.7/crop of Fig 2A complete Image 16.7/composite-1.tif (RGB).tif]

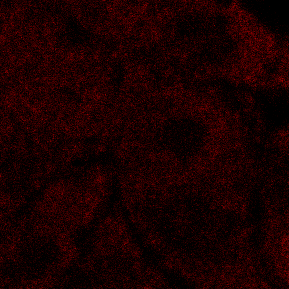

Supplement: Supplementary file 7 — Source data Fig. 2 [file 44318_2025_448_MOESM7_ESM.zip › Figure 2/Fig 2A/16.7/crop of Fig 2A complete Image 16.7/eif3b.tif]

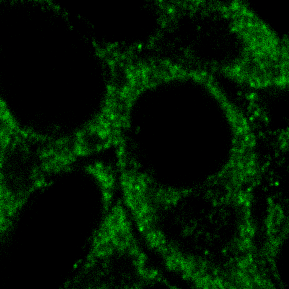

Supplement: Supplementary file 7 — Source data Fig. 2 [file 44318_2025_448_MOESM7_ESM.zip › Figure 2/Fig 2A/16.7/crop of Fig 2A complete Image 16.7/g3bp1.tif]

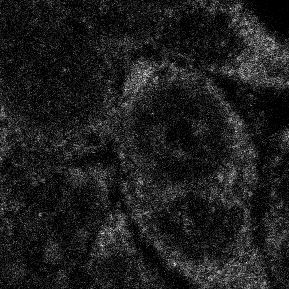

Supplement: Supplementary file 7 — Source data Fig. 2 [file 44318_2025_448_MOESM7_ESM.zip › Figure 2/Fig 2A/16.7/crop of Fig 2A complete Image 16.7/ins mrna.tif]

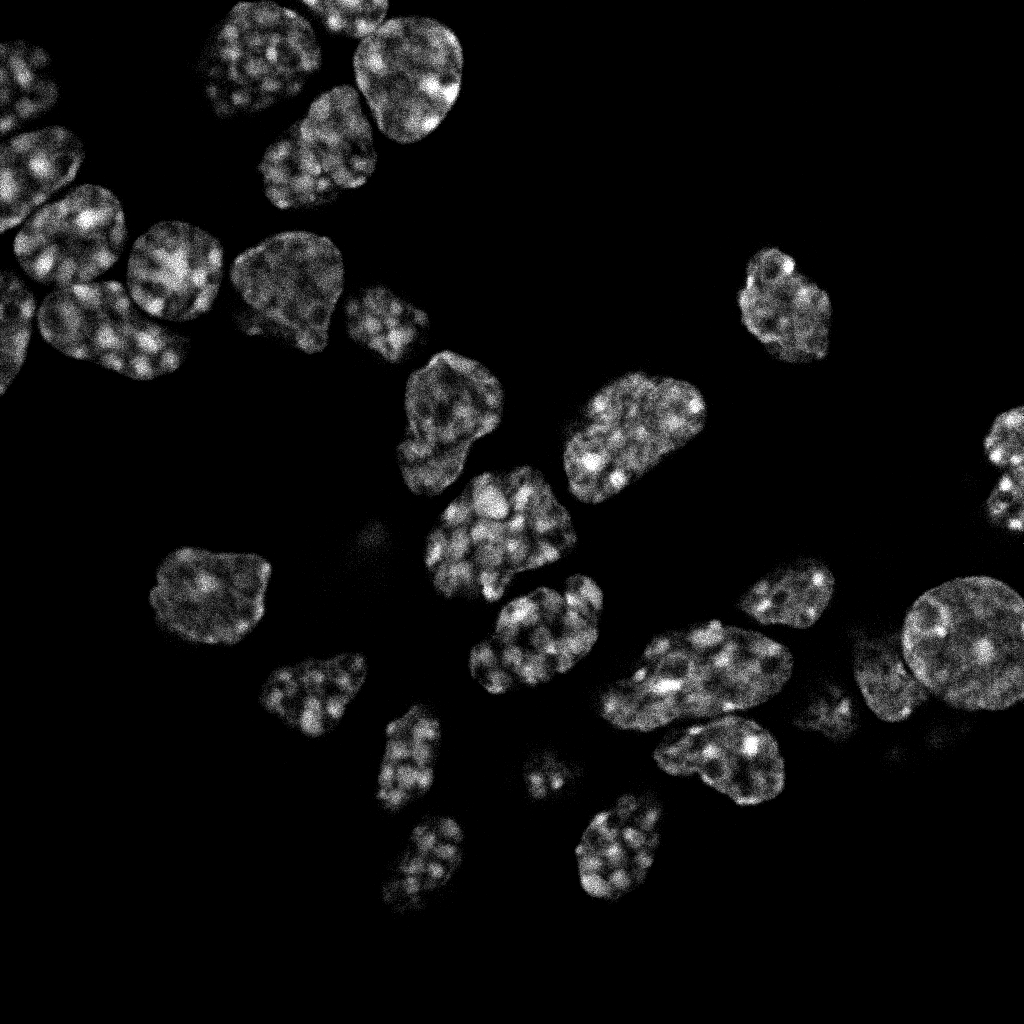

Supplement: Supplementary file 7 — Source data Fig. 2 [file 44318_2025_448_MOESM7_ESM.zip › Figure 2/Fig 2A/16.7 + ars/Fig 2A complete Image 16.7 ars.tif]

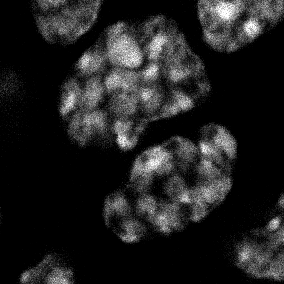

Supplement: Supplementary file 7 — Source data Fig. 2 [file 44318_2025_448_MOESM7_ESM.zip › Figure 2/Fig 2A/16.7 + ars/crop of Fig 2A complete Image 16.7 ars/composite-3.tif]

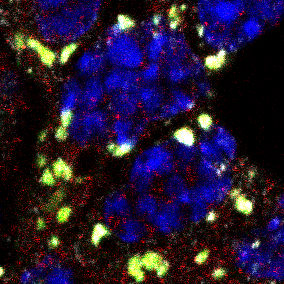

Supplement: Supplementary file 7 — Source data Fig. 2 [file 44318_2025_448_MOESM7_ESM.zip › Figure 2/Fig 2A/16.7 + ars/crop of Fig 2A complete Image 16.7 ars/composite-3.tif (RGB).tif]

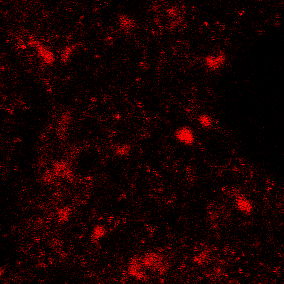

Supplement: Supplementary file 7 — Source data Fig. 2 [file 44318_2025_448_MOESM7_ESM.zip › Figure 2/Fig 2A/16.7 + ars/crop of Fig 2A complete Image 16.7 ars/eif3b.tif]

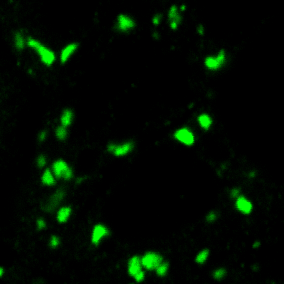

Supplement: Supplementary file 7 — Source data Fig. 2 [file 44318_2025_448_MOESM7_ESM.zip › Figure 2/Fig 2A/16.7 + ars/crop of Fig 2A complete Image 16.7 ars/g3bp1.tif]

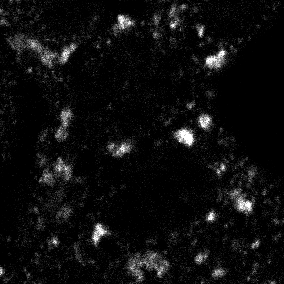

Supplement: Supplementary file 7 — Source data Fig. 2 [file 44318_2025_448_MOESM7_ESM.zip › Figure 2/Fig 2A/16.7 + ars/crop of Fig 2A complete Image 16.7 ars/ins mrna.tif]

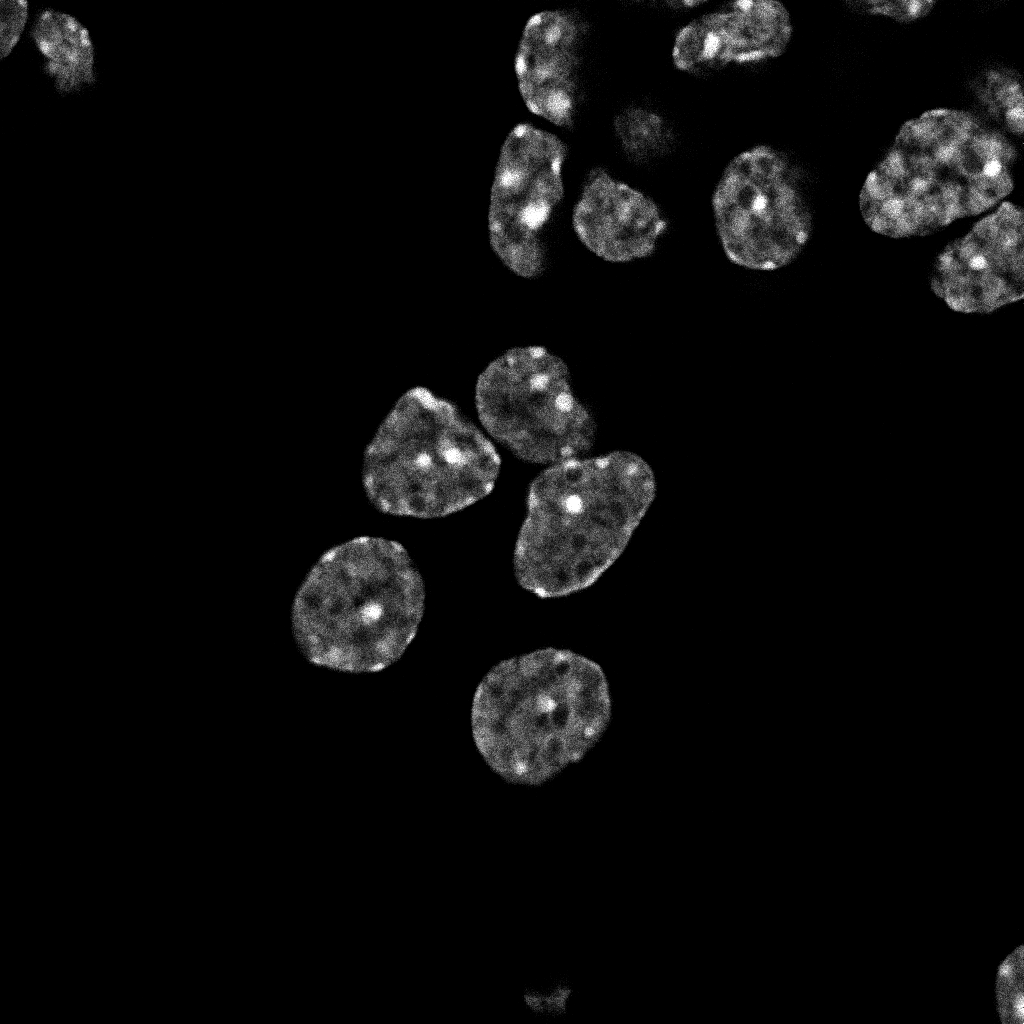

Supplement: Supplementary file 7 — Source data Fig. 2 [file 44318_2025_448_MOESM7_ESM.zip › Figure 2/Fig 2A/2.8/Fig 2A complete Image 2.8.tif]

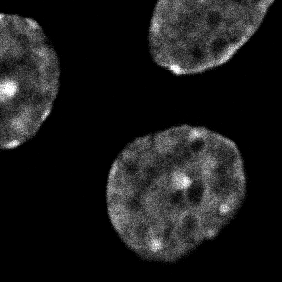

Supplement: Supplementary file 7 — Source data Fig. 2 [file 44318_2025_448_MOESM7_ESM.zip › Figure 2/Fig 2A/2.8/crop of. Fig 2A complete Image 2.8/composite.tif]

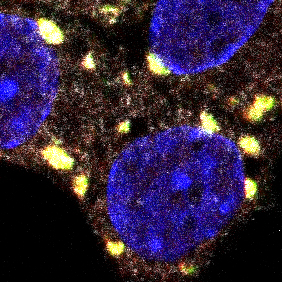

Supplement: Supplementary file 7 — Source data Fig. 2 [file 44318_2025_448_MOESM7_ESM.zip › Figure 2/Fig 2A/2.8/crop of. Fig 2A complete Image 2.8/composite.tif (RGB).tif]

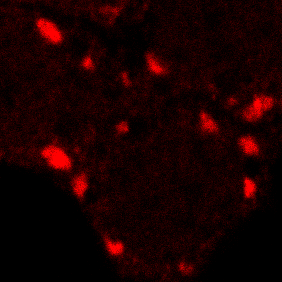

Supplement: Supplementary file 7 — Source data Fig. 2 [file 44318_2025_448_MOESM7_ESM.zip › Figure 2/Fig 2A/2.8/crop of. Fig 2A complete Image 2.8/eif3b.tif]

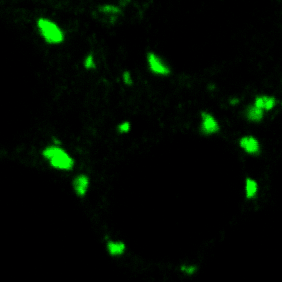

Supplement: Supplementary file 7 — Source data Fig. 2 [file 44318_2025_448_MOESM7_ESM.zip › Figure 2/Fig 2A/2.8/crop of. Fig 2A complete Image 2.8/g3bp1.tif]

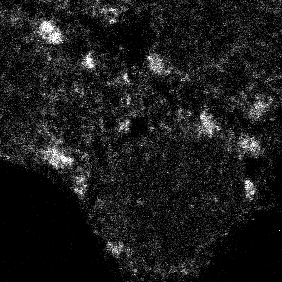

Supplement: Supplementary file 7 — Source data Fig. 2 [file 44318_2025_448_MOESM7_ESM.zip › Figure 2/Fig 2A/2.8/crop of. Fig 2A complete Image 2.8/ins mrna.tif]

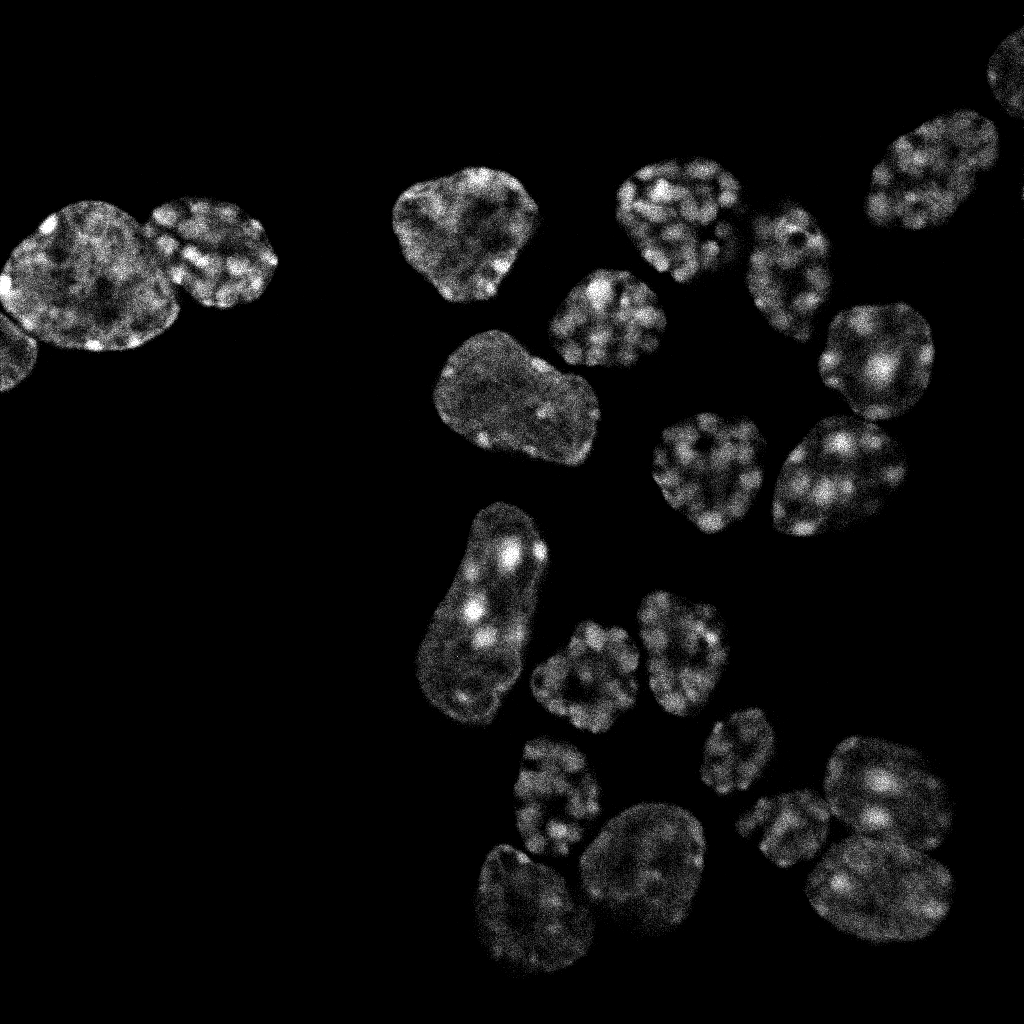

Supplement: Supplementary file 7 — Source data Fig. 2 [file 44318_2025_448_MOESM7_ESM.zip › Figure 2/Fig 2A/2.8 + ars/Fig 2A complete Image 2.8 ars.tif]

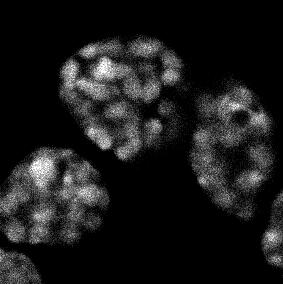

Supplement: Supplementary file 7 — Source data Fig. 2 [file 44318_2025_448_MOESM7_ESM.zip › Figure 2/Fig 2A/2.8 + ars/crop of Fig 2A complete Image 2.8 ars/composite.tif]

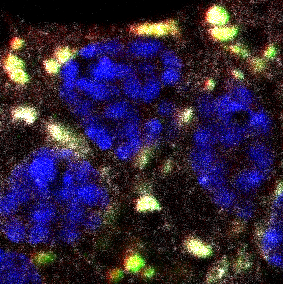

Supplement: Supplementary file 7 — Source data Fig. 2 [file 44318_2025_448_MOESM7_ESM.zip › Figure 2/Fig 2A/2.8 + ars/crop of Fig 2A complete Image 2.8 ars/composite.tif (RGB).tif]

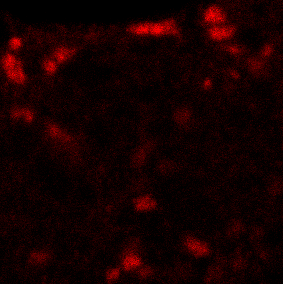

Supplement: Supplementary file 7 — Source data Fig. 2 [file 44318_2025_448_MOESM7_ESM.zip › Figure 2/Fig 2A/2.8 + ars/crop of Fig 2A complete Image 2.8 ars/eIF3b.tif]

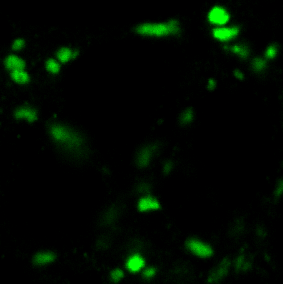

Supplement: Supplementary file 7 — Source data Fig. 2 [file 44318_2025_448_MOESM7_ESM.zip › Figure 2/Fig 2A/2.8 + ars/crop of Fig 2A complete Image 2.8 ars/g3bp1.tif]

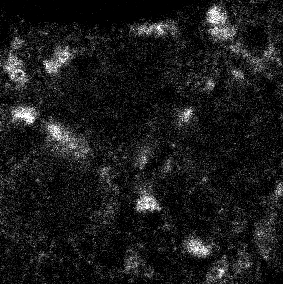

Supplement: Supplementary file 7 — Source data Fig. 2 [file 44318_2025_448_MOESM7_ESM.zip › Figure 2/Fig 2A/2.8 + ars/crop of Fig 2A complete Image 2.8 ars/ins mrna.tif]

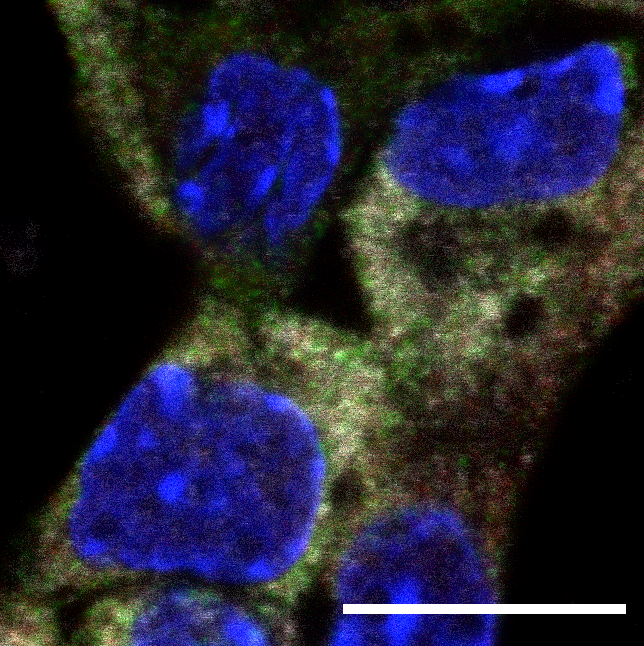

Supplement: Supplementary file 7 — Source data Fig. 2 [file 44318_2025_448_MOESM7_ESM.zip › Figure 2/Fig 2A/CHX/Composite crop.tif (RGB).tif]

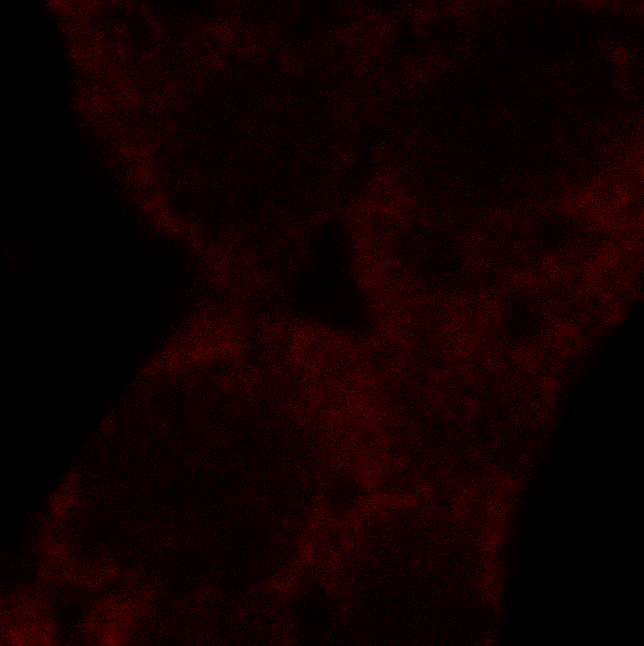

Supplement: Supplementary file 7 — Source data Fig. 2 [file 44318_2025_448_MOESM7_ESM.zip › Figure 2/Fig 2A/CHX/eif3b.tif]

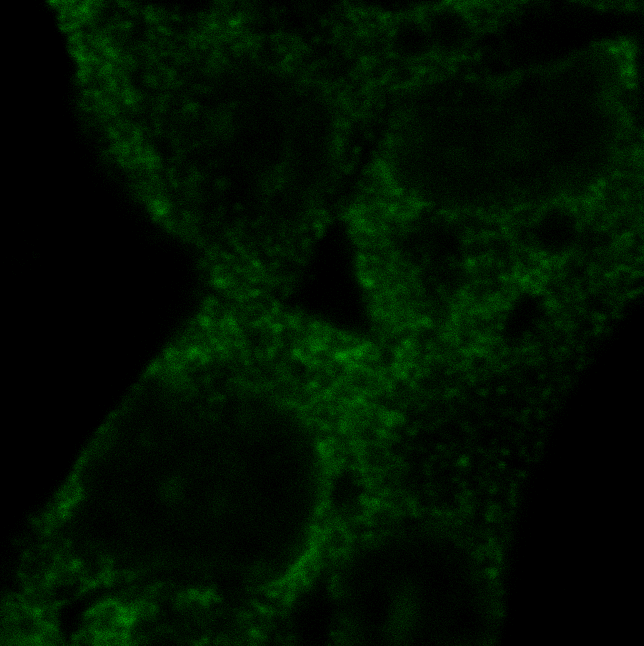

Supplement: Supplementary file 7 — Source data Fig. 2 [file 44318_2025_448_MOESM7_ESM.zip › Figure 2/Fig 2A/CHX/g3bp1.tif]

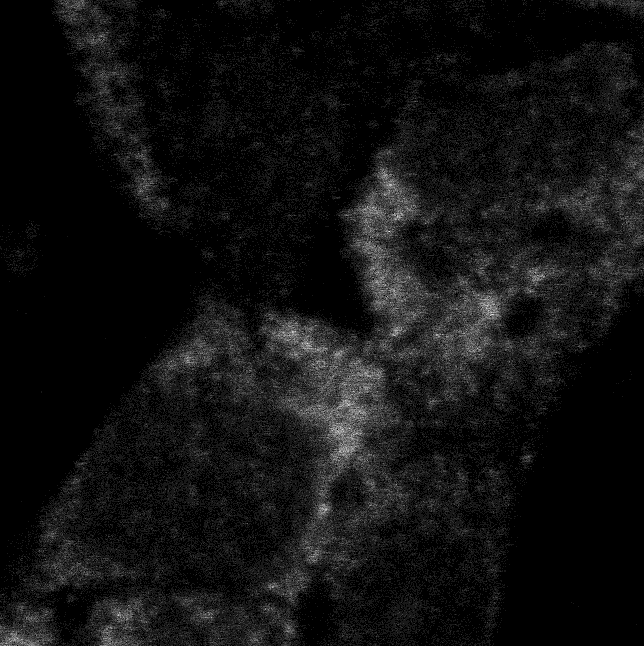

Supplement: Supplementary file 7 — Source data Fig. 2 [file 44318_2025_448_MOESM7_ESM.zip › Figure 2/Fig 2A/CHX/ins mrna.tif]

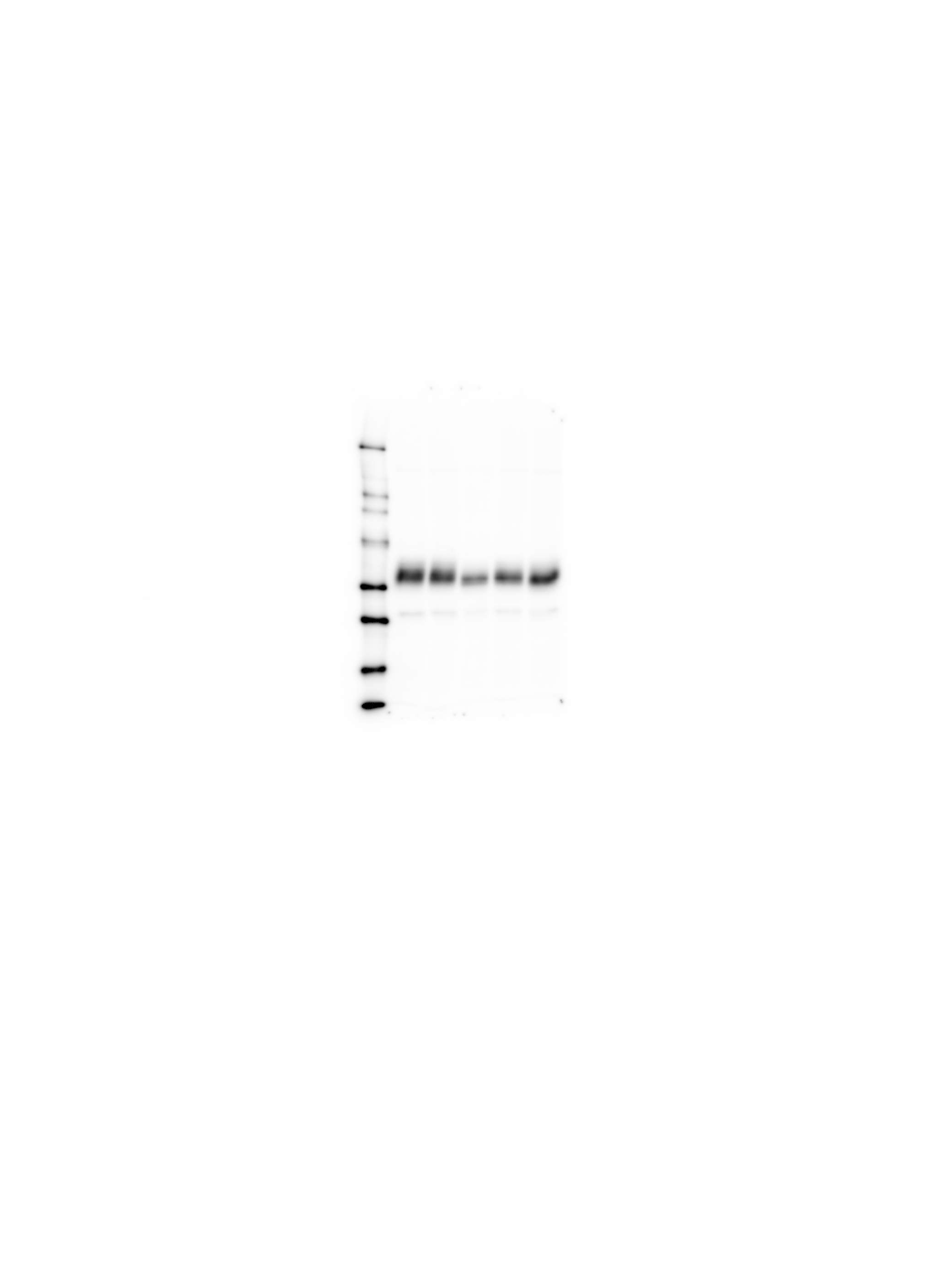

Supplement: Supplementary file 7 — Source data Fig. 2 [file 44318_2025_448_MOESM7_ESM.zip › Figure 2/Fig 2E-F/AMPKA/AMPKA.tif]

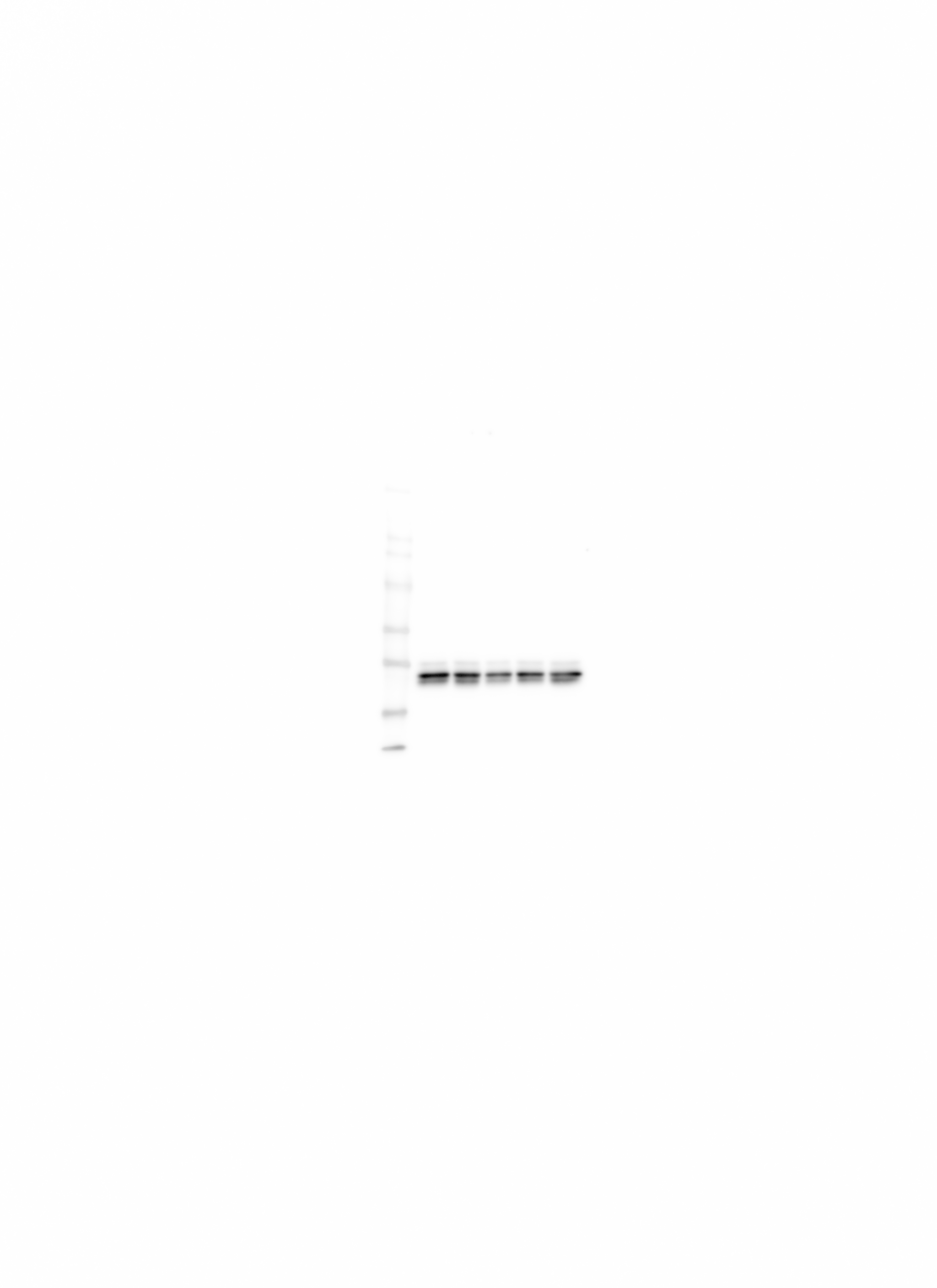

Supplement: Supplementary file 7 — Source data Fig. 2 [file 44318_2025_448_MOESM7_ESM.zip › Figure 2/Fig 2E-F/AMPKA/YTUB.tif]

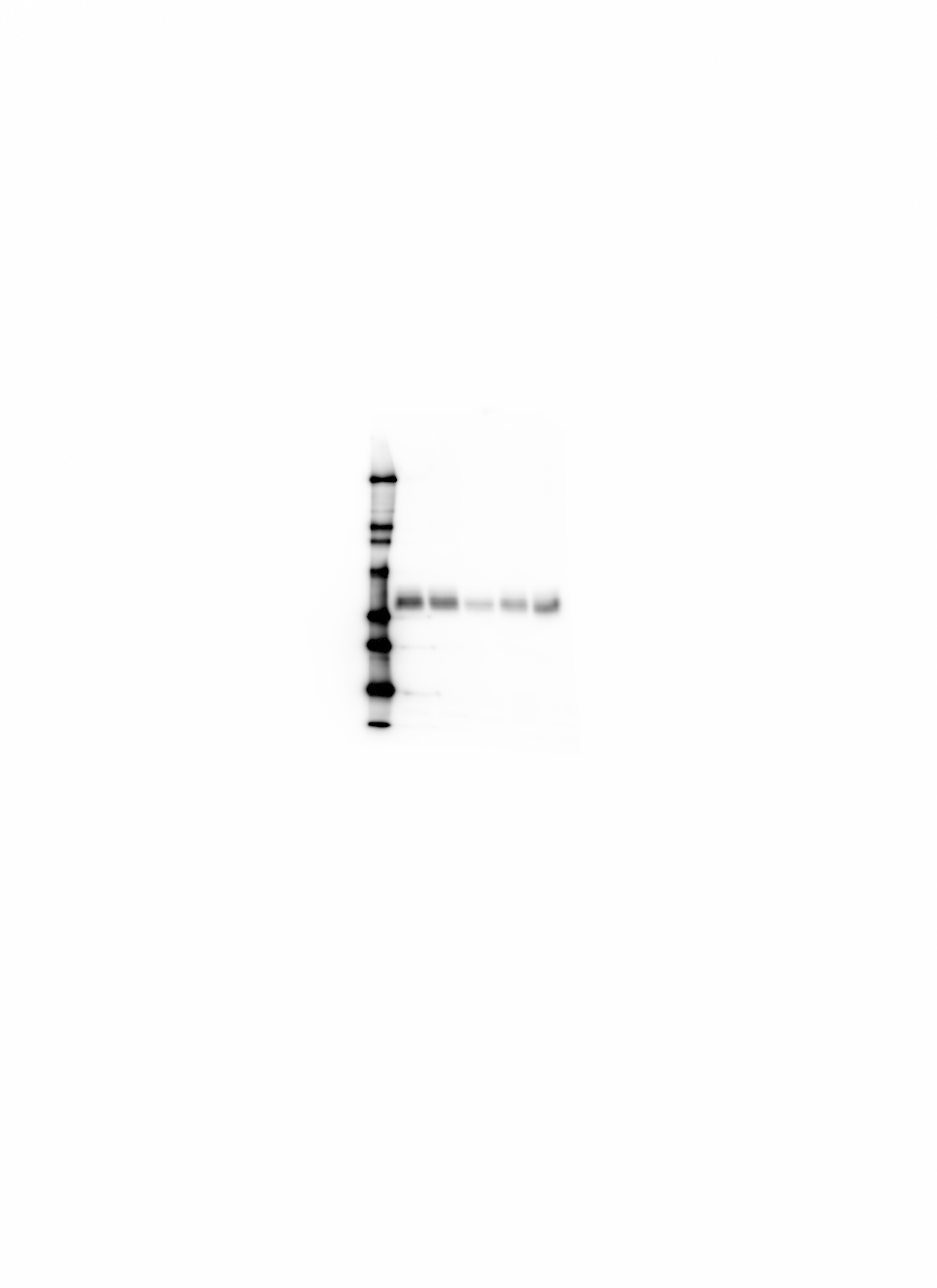

Supplement: Supplementary file 7 — Source data Fig. 2 [file 44318_2025_448_MOESM7_ESM.zip › Figure 2/Fig 2E-F/AMPKA-P/AMPKA-P.tif]

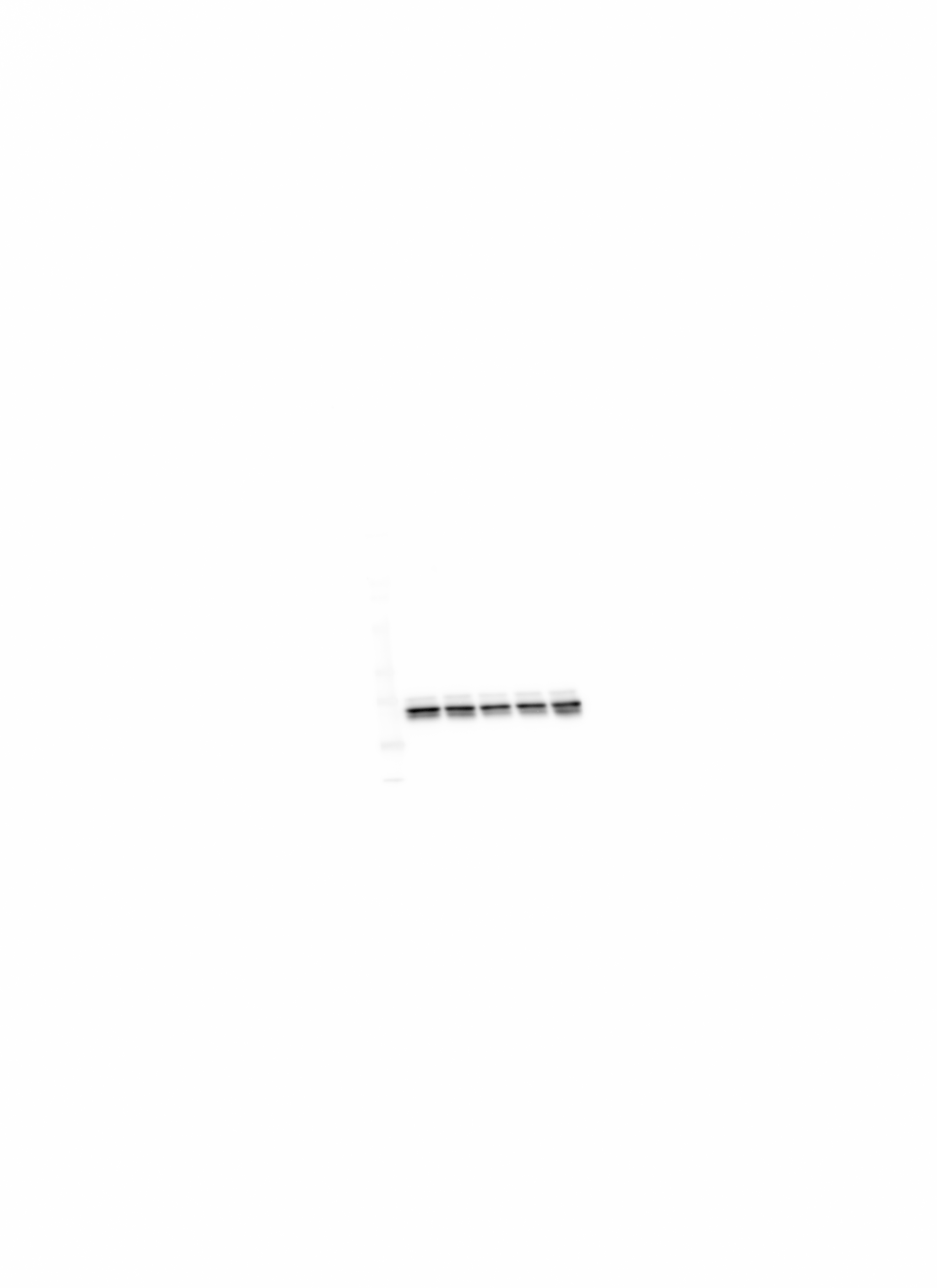

Supplement: Supplementary file 7 — Source data Fig. 2 [file 44318_2025_448_MOESM7_ESM.zip › Figure 2/Fig 2E-F/AMPKA-P/YTUB.tif]

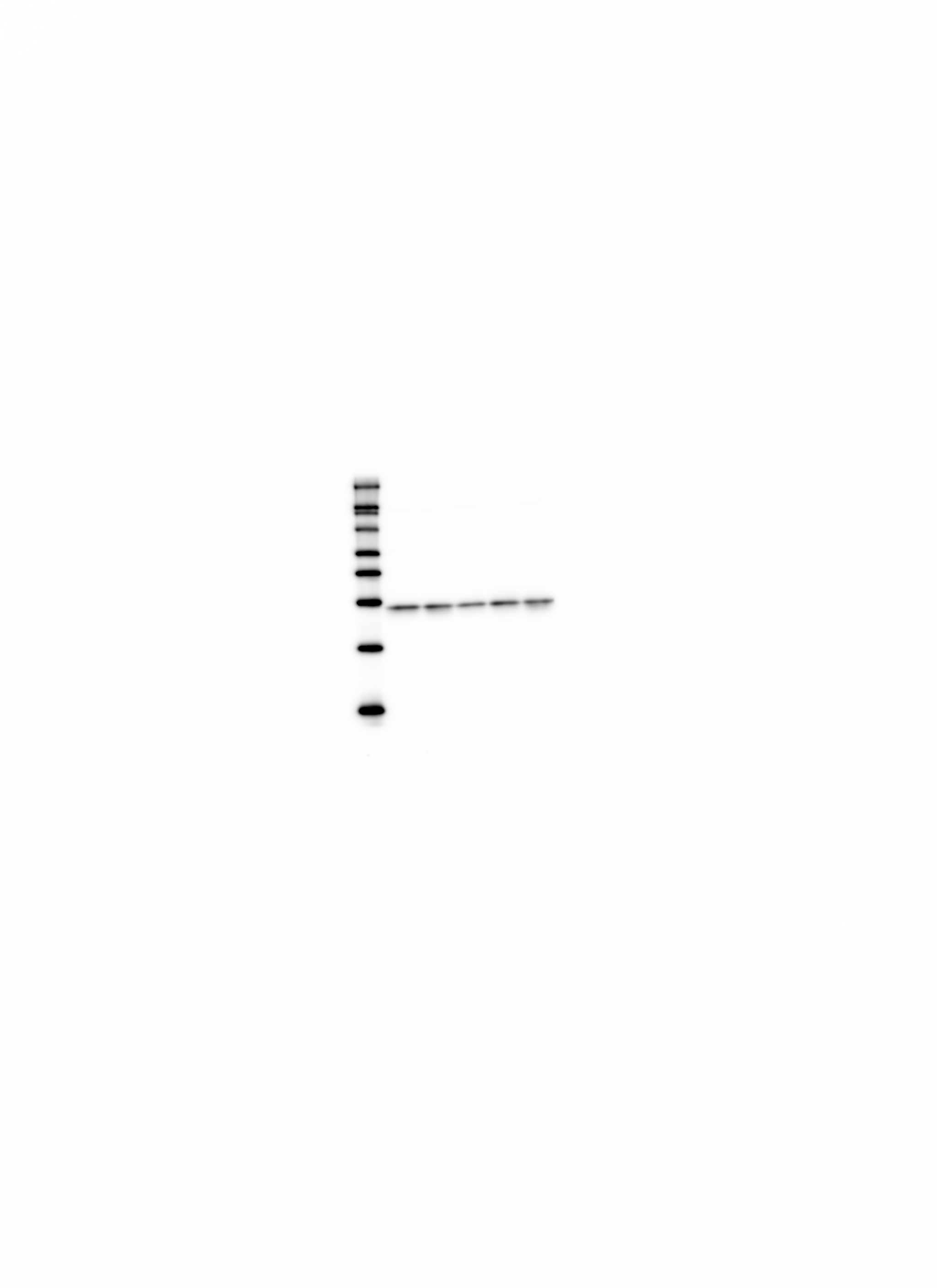

Supplement: Supplementary file 7 — Source data Fig. 2 [file 44318_2025_448_MOESM7_ESM.zip › Figure 2/Fig 2E-F/EIF2A/EIF2A.tif]

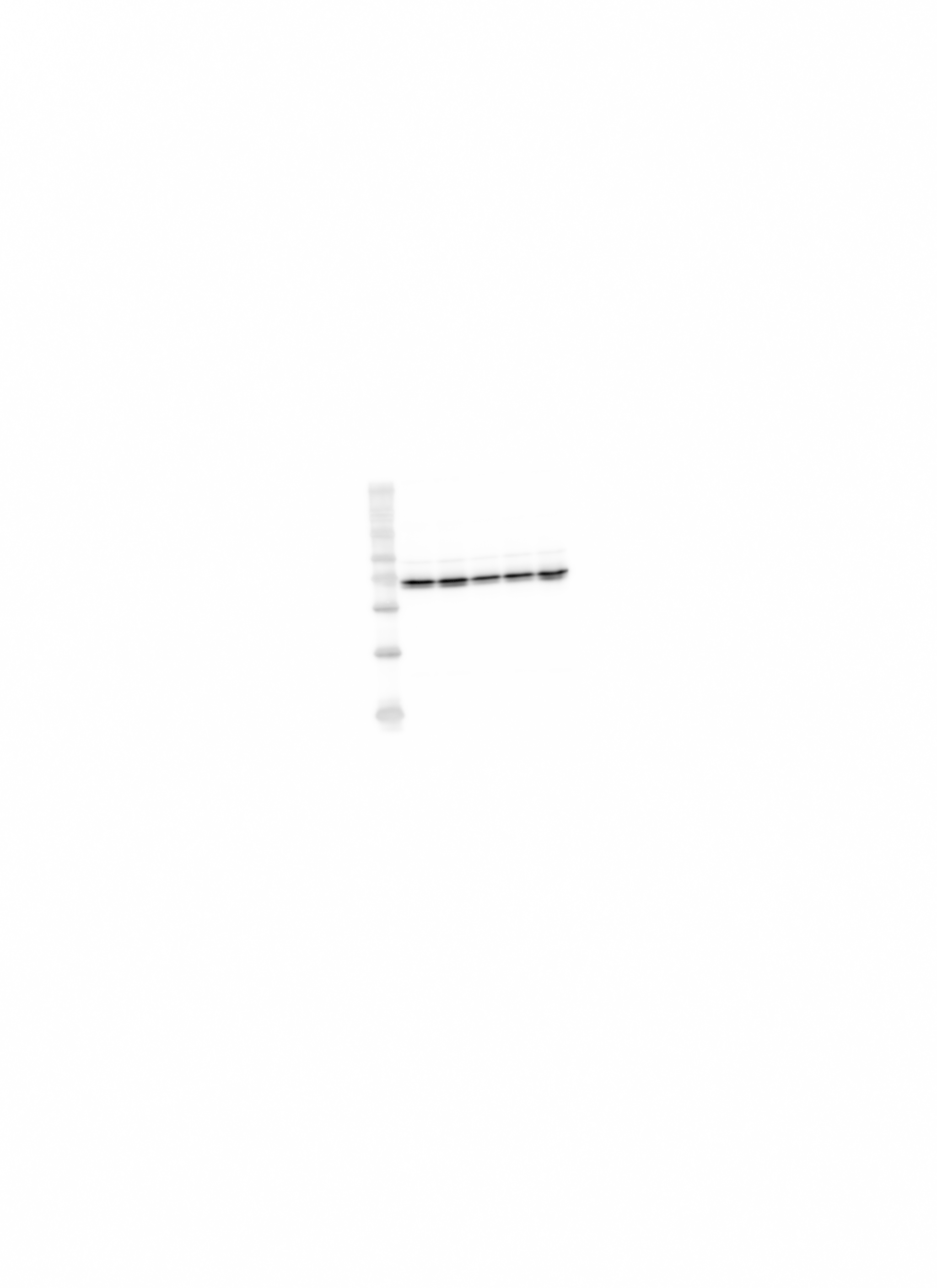

Supplement: Supplementary file 7 — Source data Fig. 2 [file 44318_2025_448_MOESM7_ESM.zip › Figure 2/Fig 2E-F/EIF2A/ytub.tif]

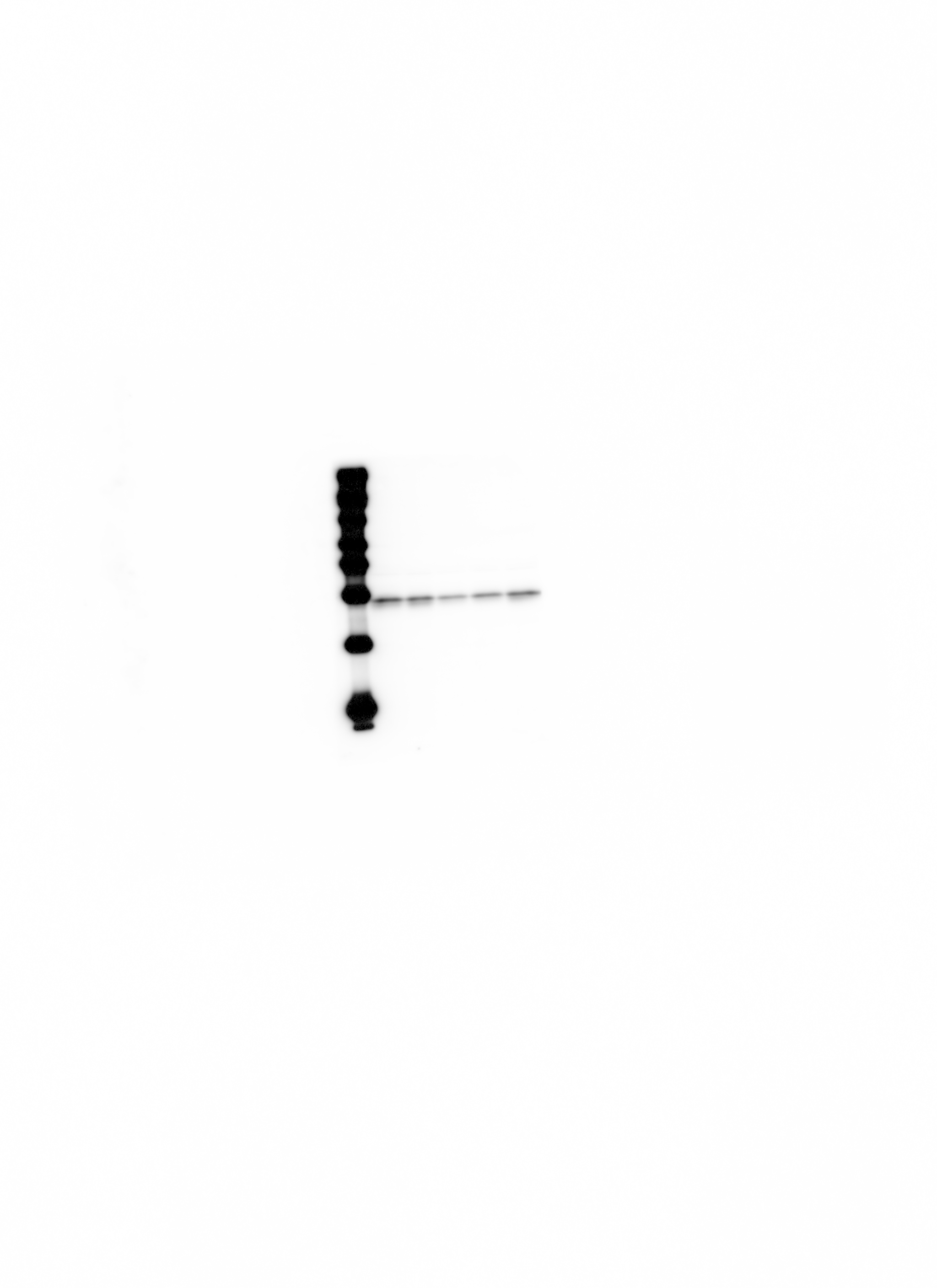

Supplement: Supplementary file 7 — Source data Fig. 2 [file 44318_2025_448_MOESM7_ESM.zip › Figure 2/Fig 2E-F/EIF2A-P/EIF2A-P.tif]

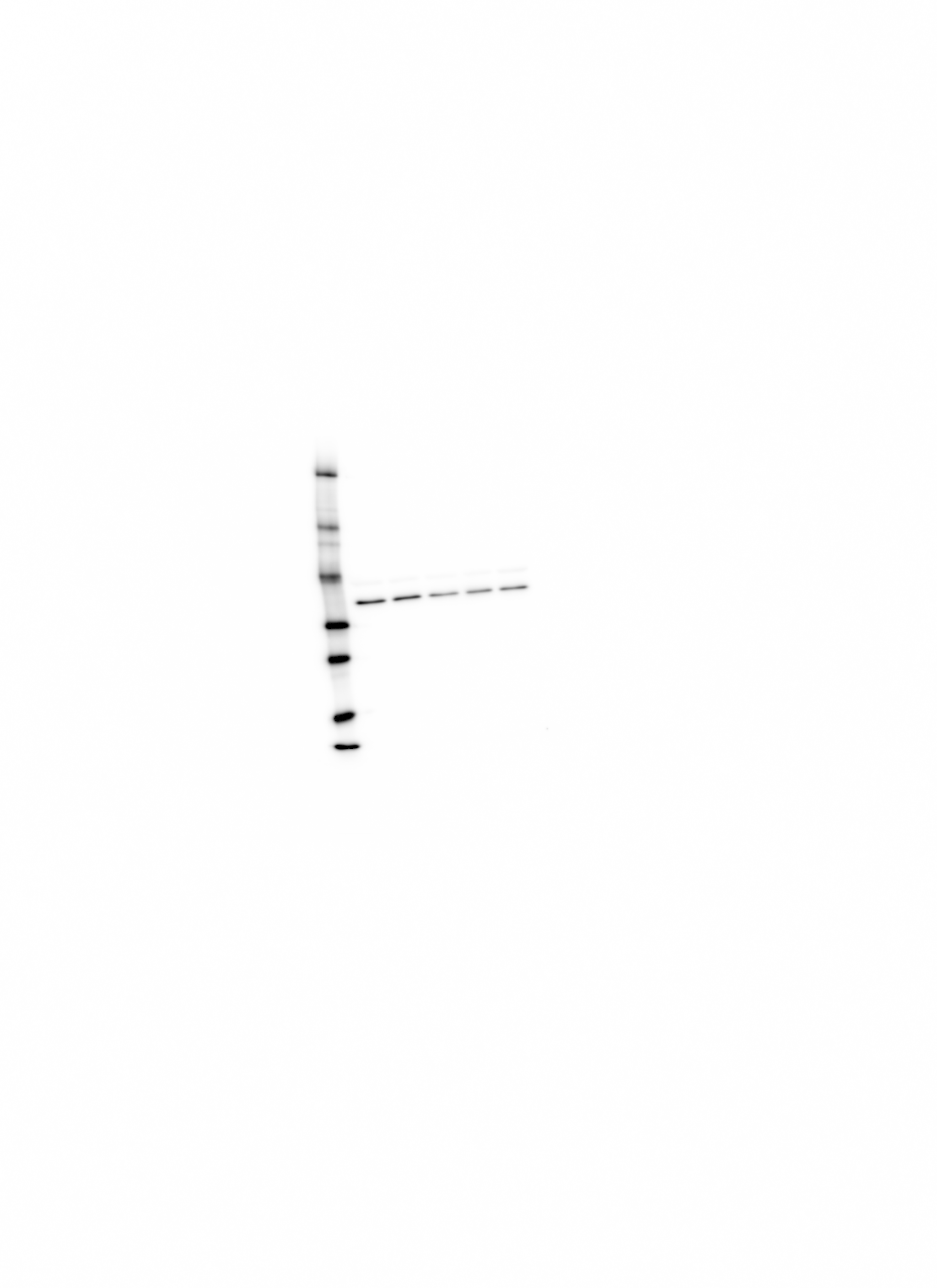

Supplement: Supplementary file 7 — Source data Fig. 2 [file 44318_2025_448_MOESM7_ESM.zip › Figure 2/Fig 2E-F/G3BP1/G3BP1.tif]

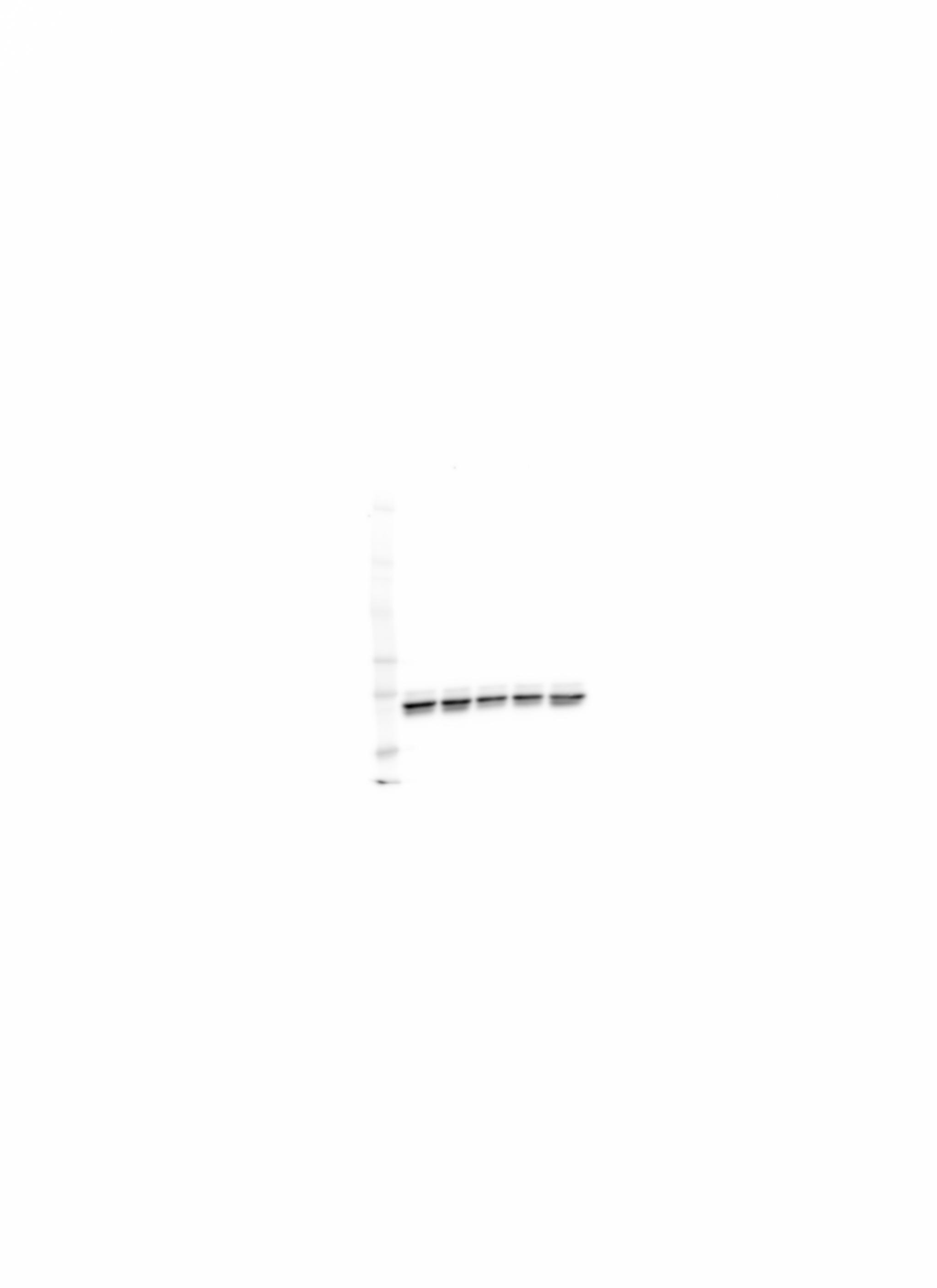

Supplement: Supplementary file 7 — Source data Fig. 2 [file 44318_2025_448_MOESM7_ESM.zip › Figure 2/Fig 2E-F/G3BP1/ytub.tif]

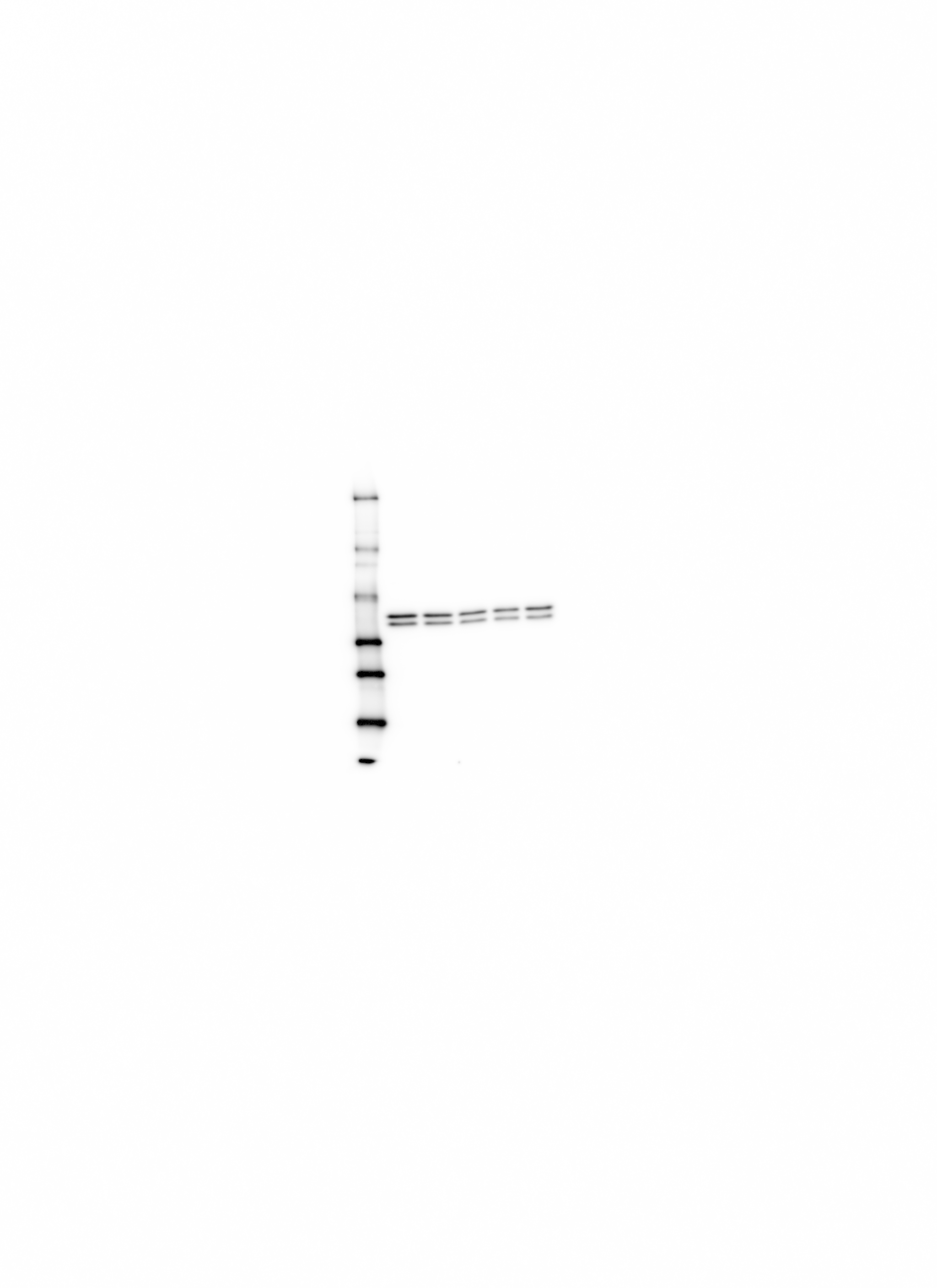

Supplement: Supplementary file 7 — Source data Fig. 2 [file 44318_2025_448_MOESM7_ESM.zip › Figure 2/Fig 2E-F/G3BP2/G3BP2.tif]

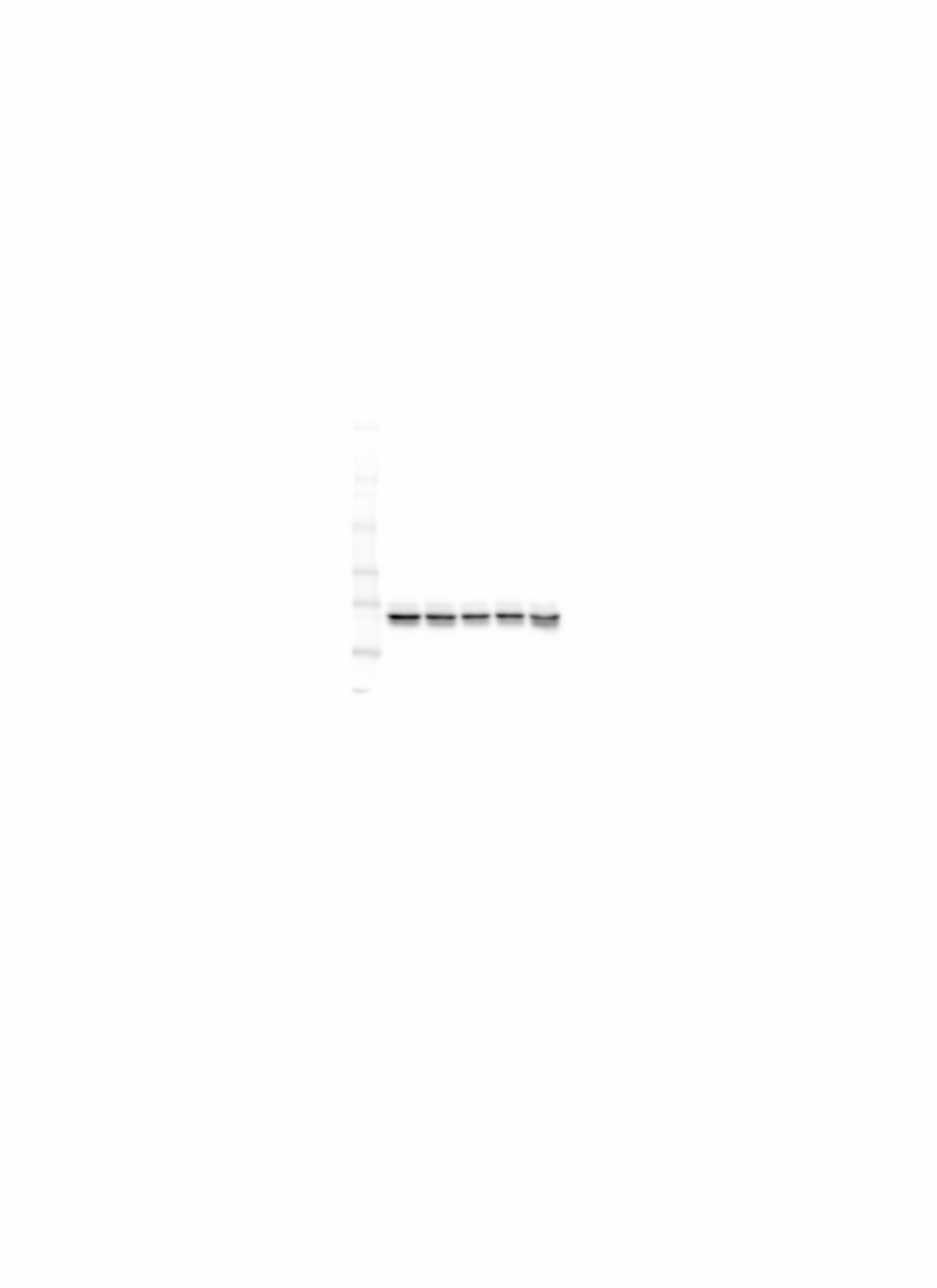

Supplement: Supplementary file 7 — Source data Fig. 2 [file 44318_2025_448_MOESM7_ESM.zip › Figure 2/Fig 2E-F/G3BP2/YTUB.tif]

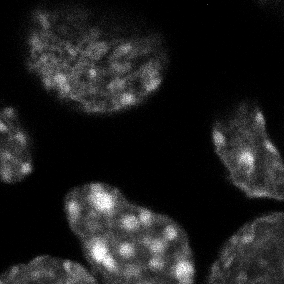

Supplement: Supplementary file 7 — Source data Fig. 2 [file 44318_2025_448_MOESM7_ESM.zip › Figure 2/Fig 2G/16.7/ crop of Fig 2G 16.7 complete image/composiute crop.tif]

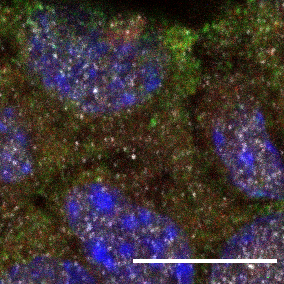

Supplement: Supplementary file 7 — Source data Fig. 2 [file 44318_2025_448_MOESM7_ESM.zip › Figure 2/Fig 2G/16.7/ crop of Fig 2G 16.7 complete image/composiute crop.tif (RGB).tif]

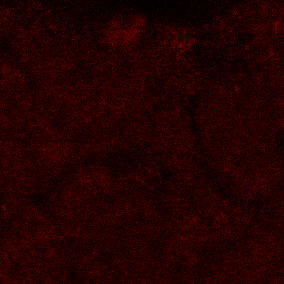

Supplement: Supplementary file 7 — Source data Fig. 2 [file 44318_2025_448_MOESM7_ESM.zip › Figure 2/Fig 2G/16.7/ crop of Fig 2G 16.7 complete image/eif3b.tif]

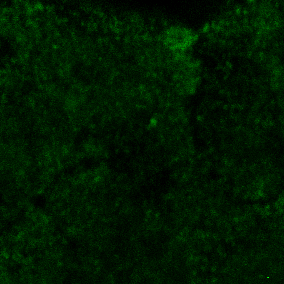

Supplement: Supplementary file 7 — Source data Fig. 2 [file 44318_2025_448_MOESM7_ESM.zip › Figure 2/Fig 2G/16.7/ crop of Fig 2G 16.7 complete image/g3bp1.tif]

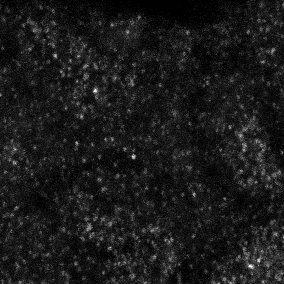

Supplement: Supplementary file 7 — Source data Fig. 2 [file 44318_2025_448_MOESM7_ESM.zip › Figure 2/Fig 2G/16.7/ crop of Fig 2G 16.7 complete image/phosphoampk.tif]

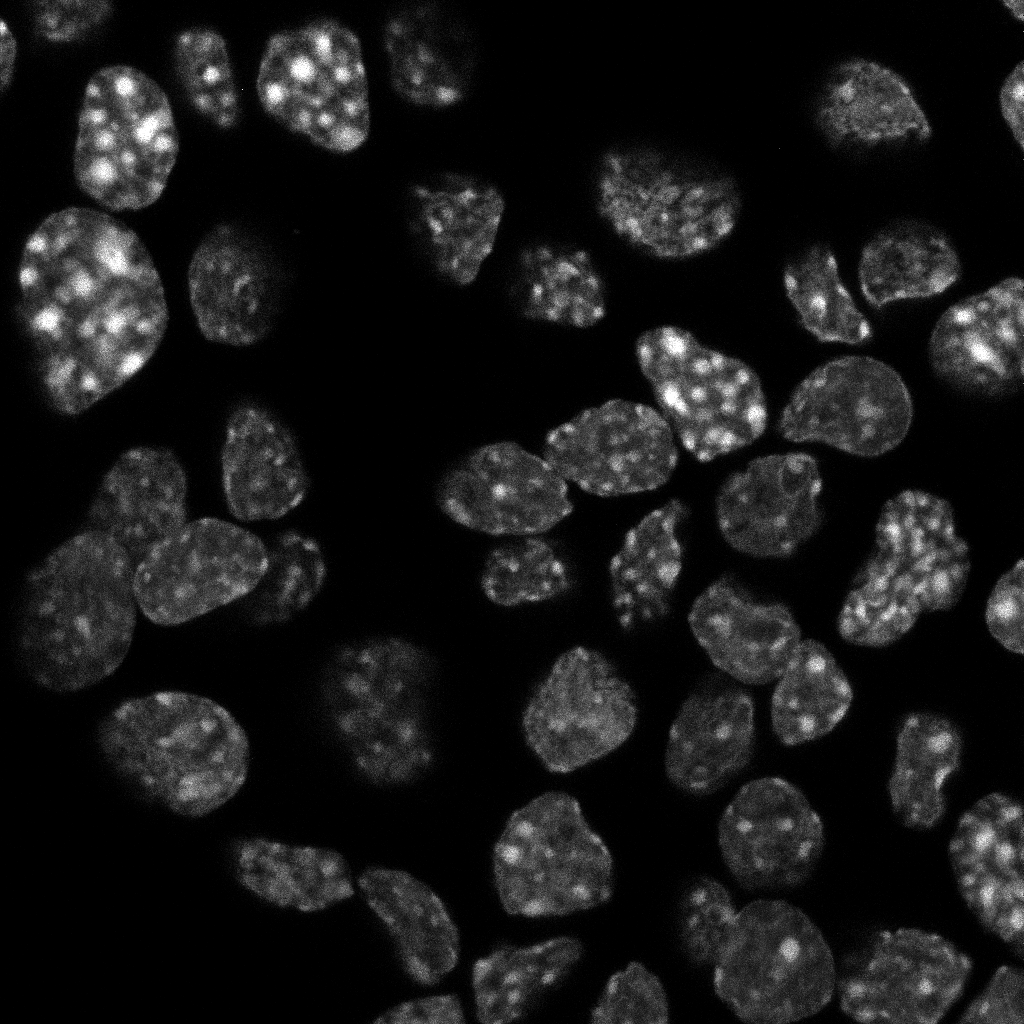

Supplement: Supplementary file 7 — Source data Fig. 2 [file 44318_2025_448_MOESM7_ESM.zip › Figure 2/Fig 2G/16.7/Fig 2G 16.7 complete image.tif]
