# Supplementary figures and images for: Aldolase-regulated G3BP1/2+ condensates control insulin mRNA storage in beta cells (part 2 of 4)
Source: EMBO J. 2025 May 12;44(13):3669–96. doi: 10.1038/s44318-025-00448-7 (PMC12216156; doi:10.1038/s44318-025-00448-7)

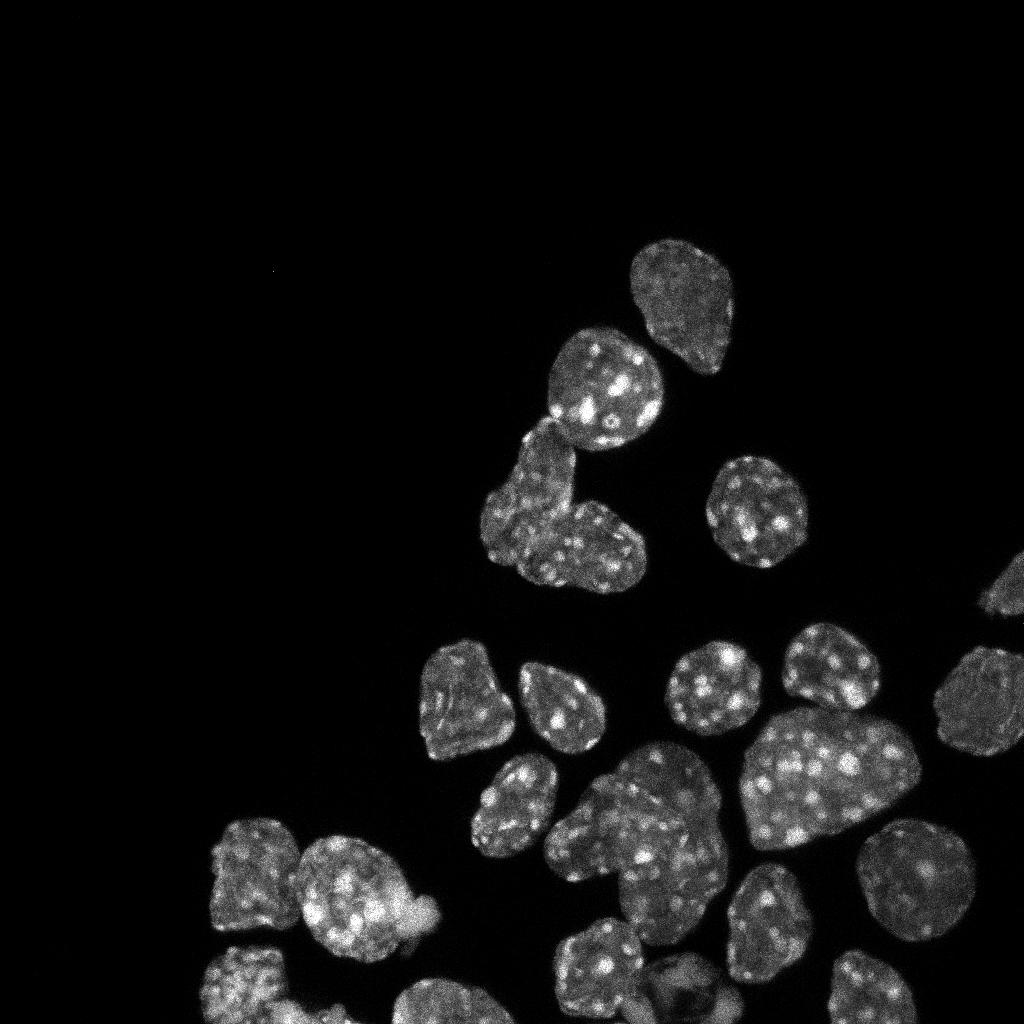

Supplement: Supplementary file 7 — Source data Fig. 2 [file 44318_2025_448_MOESM7_ESM.zip › Figure 2/Fig 2G/2.8/Fig 2G 2.8 complete image.tif]

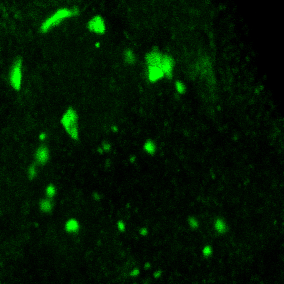

Supplement: Supplementary file 7 — Source data Fig. 2 [file 44318_2025_448_MOESM7_ESM.zip › Figure 2/Fig 2G/2.8/crop of Fig 2G 2.8 complete image/G3BP1.tif]

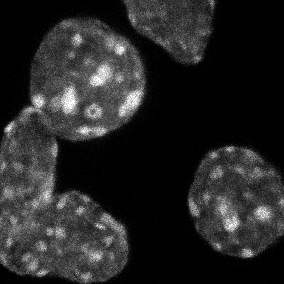

Supplement: Supplementary file 7 — Source data Fig. 2 [file 44318_2025_448_MOESM7_ESM.zip › Figure 2/Fig 2G/2.8/crop of Fig 2G 2.8 complete image/composite crop.tif]

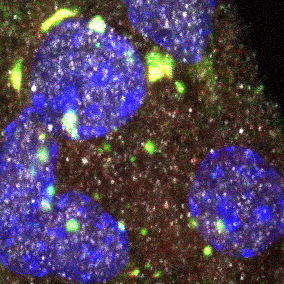

Supplement: Supplementary file 7 — Source data Fig. 2 [file 44318_2025_448_MOESM7_ESM.zip › Figure 2/Fig 2G/2.8/crop of Fig 2G 2.8 complete image/composite crop.tif (RGB).tif]

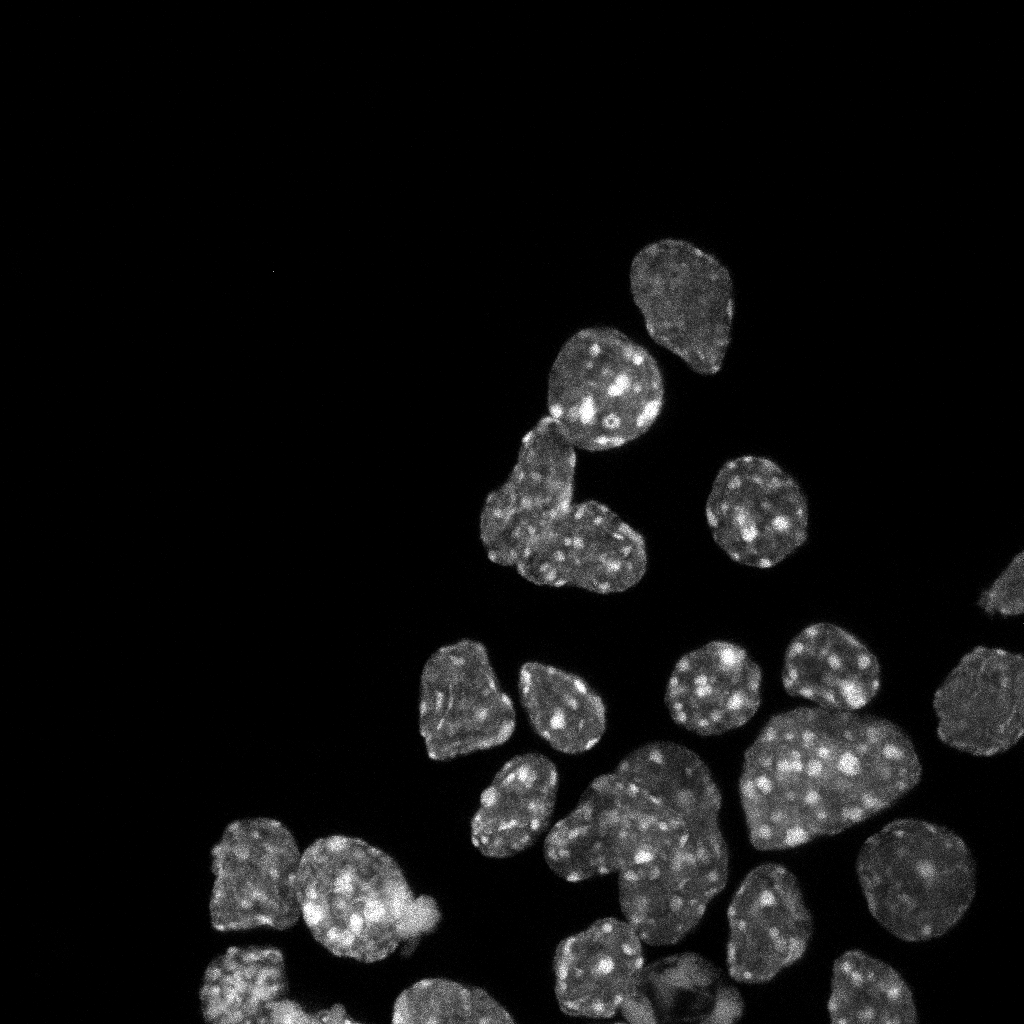

Supplement: Supplementary file 7 — Source data Fig. 2 [file 44318_2025_448_MOESM7_ESM.zip › Figure 2/Fig 2G/2.8/crop of Fig 2G 2.8 complete image/composite.tif]

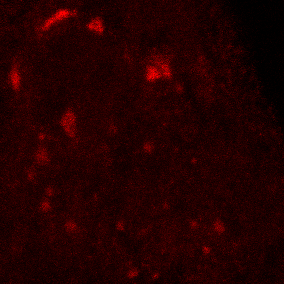

Supplement: Supplementary file 7 — Source data Fig. 2 [file 44318_2025_448_MOESM7_ESM.zip › Figure 2/Fig 2G/2.8/crop of Fig 2G 2.8 complete image/eif3b.tif]

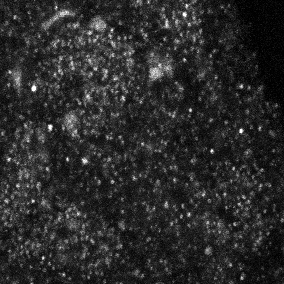

Supplement: Supplementary file 7 — Source data Fig. 2 [file 44318_2025_448_MOESM7_ESM.zip › Figure 2/Fig 2G/2.8/crop of Fig 2G 2.8 complete image/pAMPKa.tif]

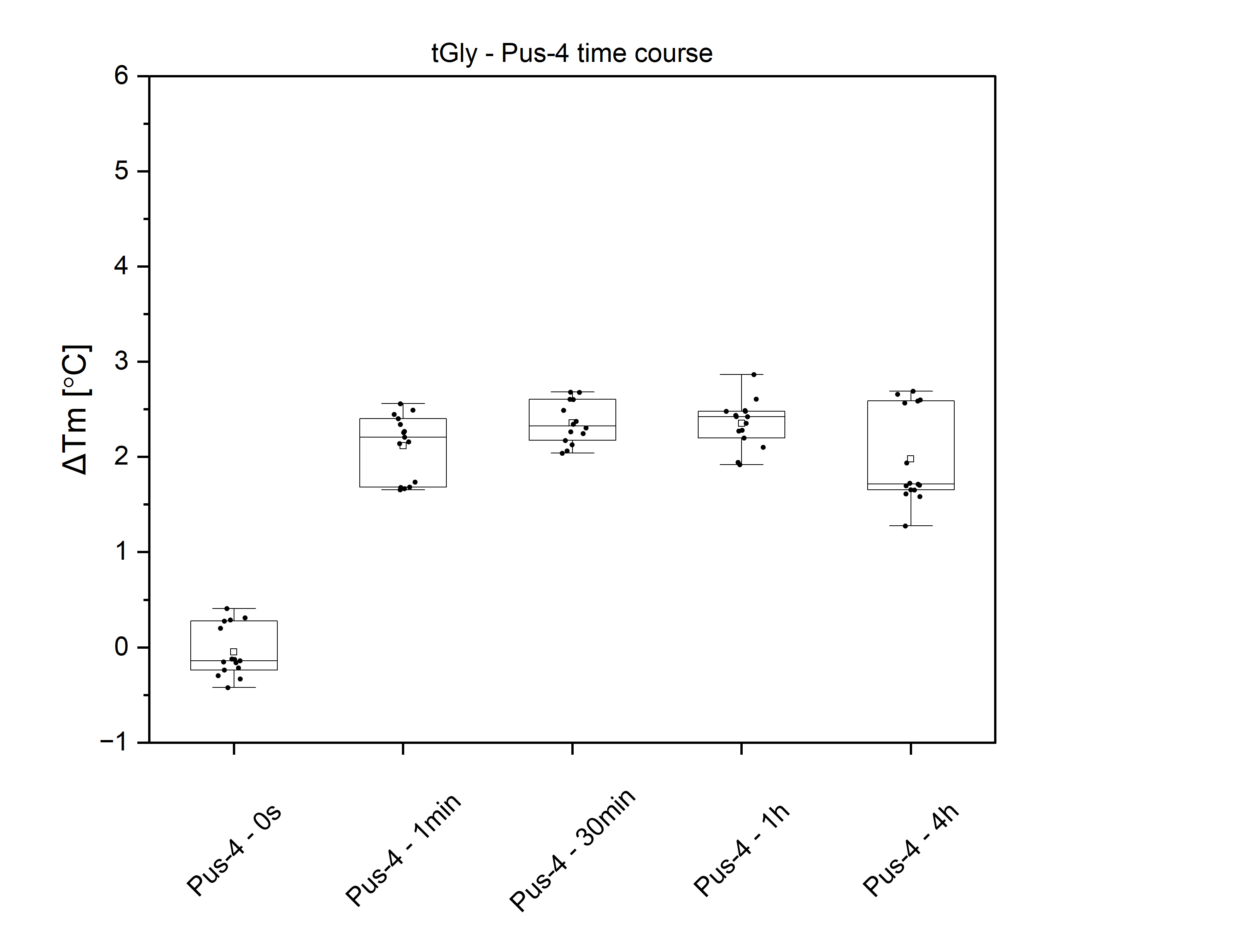

Supplement: Supplementary file 7 — Source data Fig. 2 [file 44318_2025_448_MOESM7_ESM.zip › Figure 2/Fig 2G/Corrected files for 2G/Figure2G_tGly_PUS4_TimeCourse_corrected.jpg]

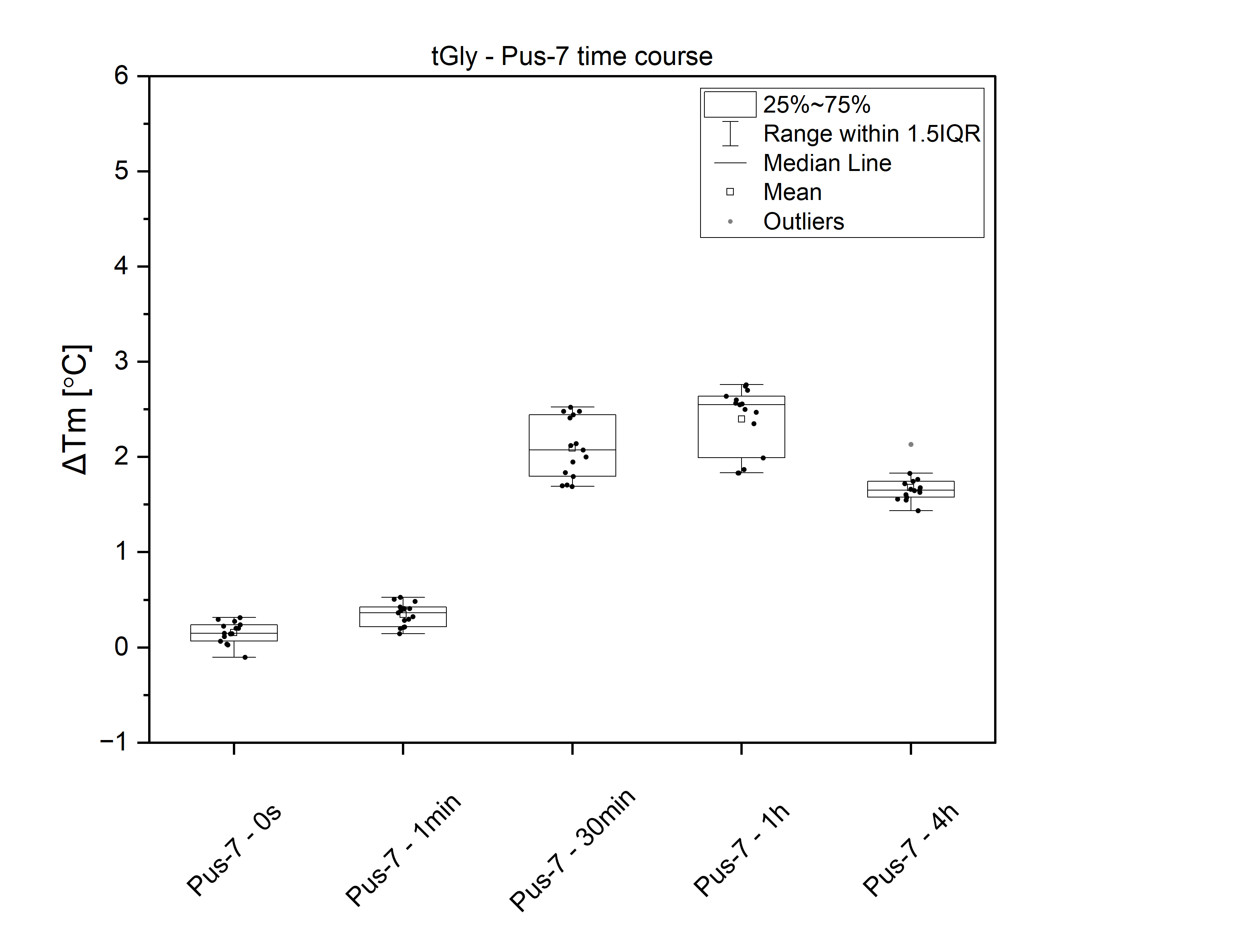

Supplement: Supplementary file 7 — Source data Fig. 2 [file 44318_2025_448_MOESM7_ESM.zip › Figure 2/Fig 2G/Corrected files for 2G/Figure2G_tGly_PUS7_TimeCourse_corrected.jpg]

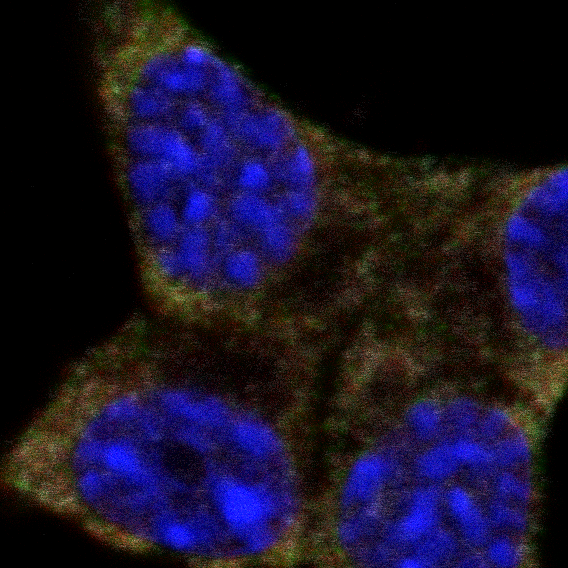

Supplement: Supplementary file 8 — Source data Fig. 3 [file 44318_2025_448_MOESM8_ESM.zip › Figure 3/Fig 3B/16.7 + DMSO/Crop of Figure 3B 16.7 complete image (RGB).tif]

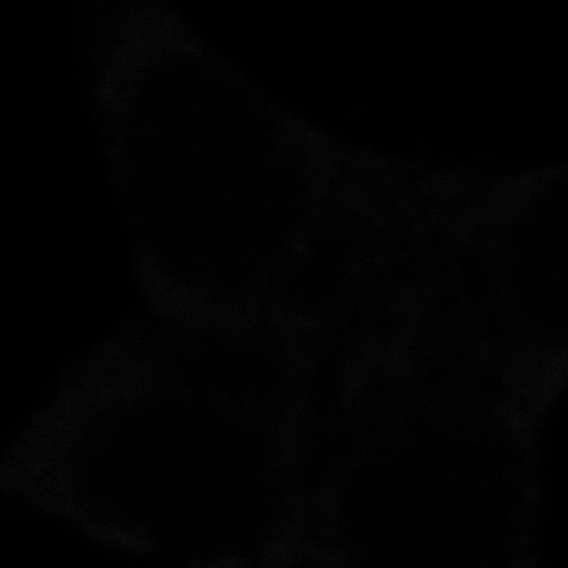

Supplement: Supplementary file 8 — Source data Fig. 3 [file 44318_2025_448_MOESM8_ESM.zip › Figure 3/Fig 3B/16.7 + DMSO/Crop of Figure 3B 16.7 complete image.tif]

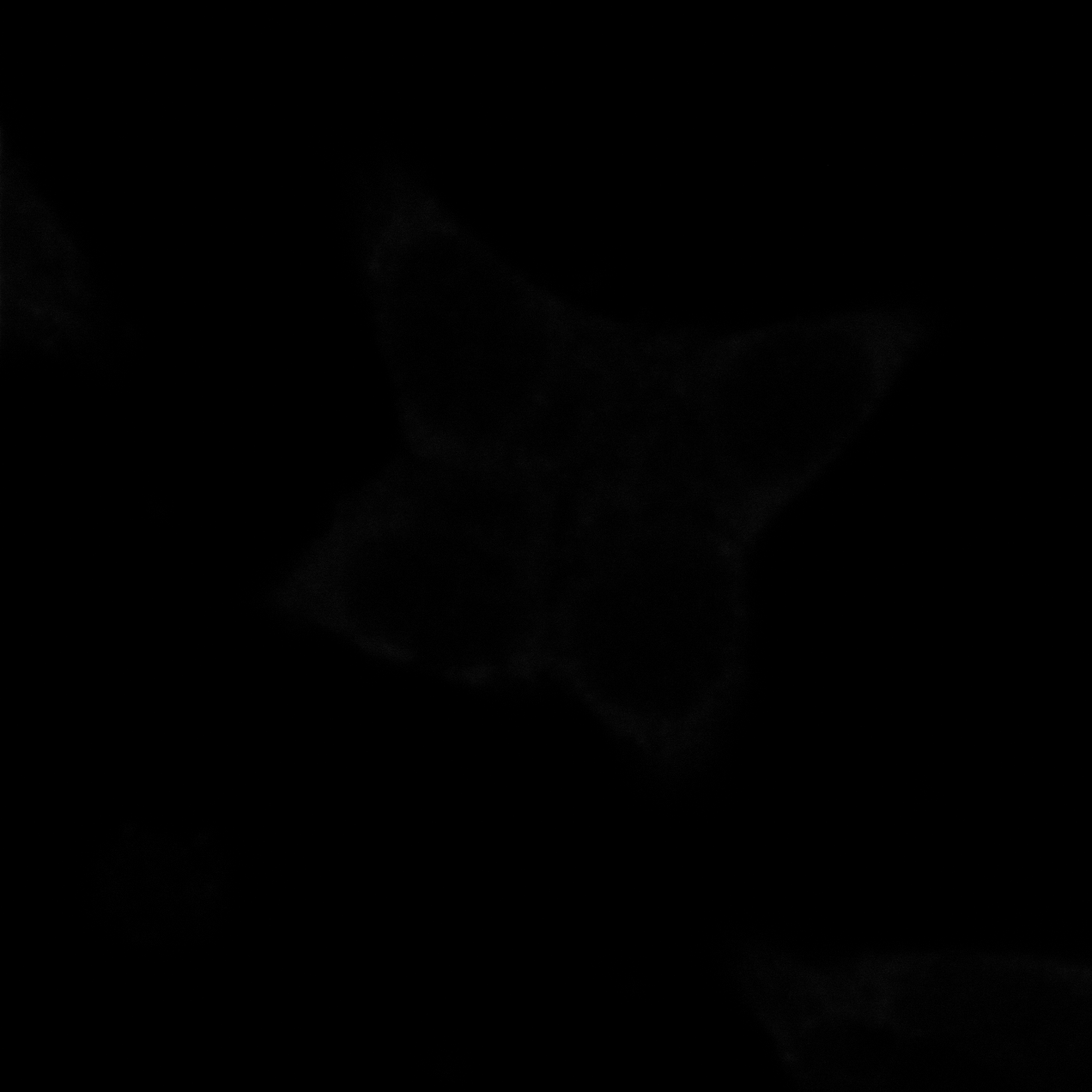

Supplement: Supplementary file 8 — Source data Fig. 3 [file 44318_2025_448_MOESM8_ESM.zip › Figure 3/Fig 3B/16.7 + DMSO/Figure 3B 16.7 complete image.tif]

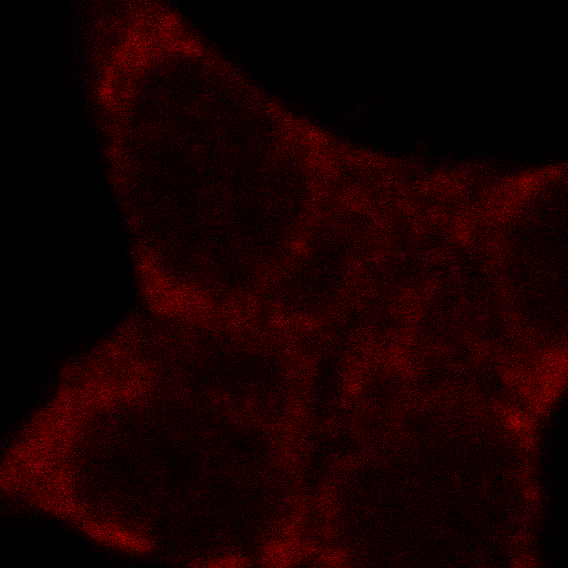

Supplement: Supplementary file 8 — Source data Fig. 3 [file 44318_2025_448_MOESM8_ESM.zip › Figure 3/Fig 3B/16.7 + DMSO/eif3b.tif]

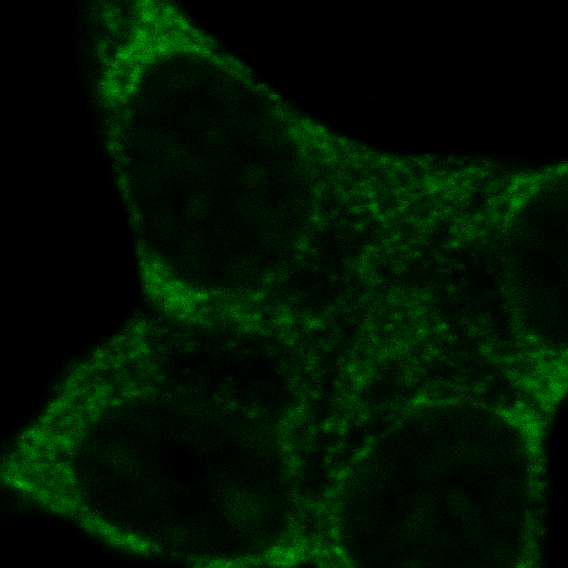

Supplement: Supplementary file 8 — Source data Fig. 3 [file 44318_2025_448_MOESM8_ESM.zip › Figure 3/Fig 3B/16.7 + DMSO/g3bp1.tif]

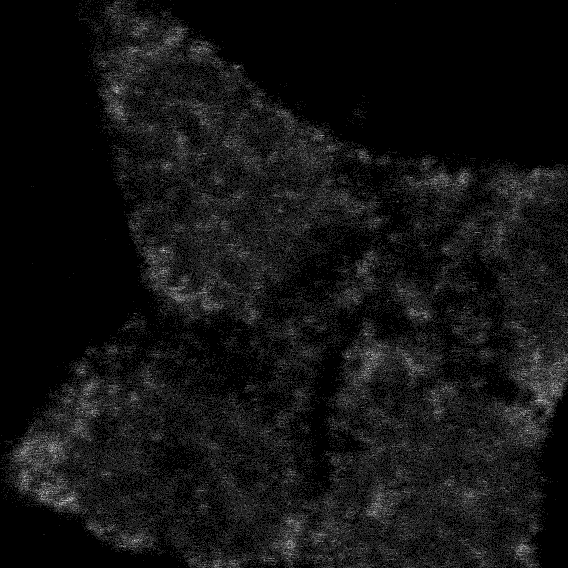

Supplement: Supplementary file 8 — Source data Fig. 3 [file 44318_2025_448_MOESM8_ESM.zip › Figure 3/Fig 3B/16.7 + DMSO/ins mrna.tif]

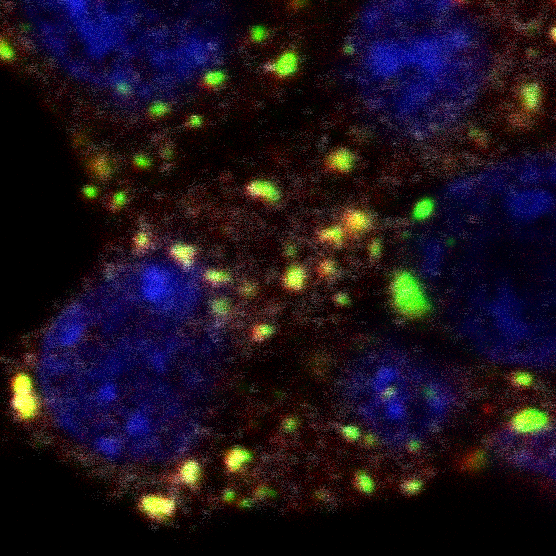

Supplement: Supplementary file 8 — Source data Fig. 3 [file 44318_2025_448_MOESM8_ESM.zip › Figure 3/Fig 3B/16.7 + aldometanib/Crop of Figure 3B 16.7 + aldometanib complete image (RGB).tif]

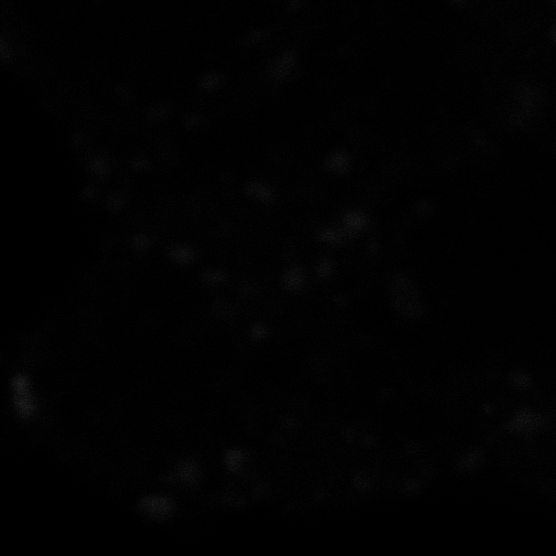

Supplement: Supplementary file 8 — Source data Fig. 3 [file 44318_2025_448_MOESM8_ESM.zip › Figure 3/Fig 3B/16.7 + aldometanib/Crop of Figure 3B 16.7 + aldometanib complete image.tif]

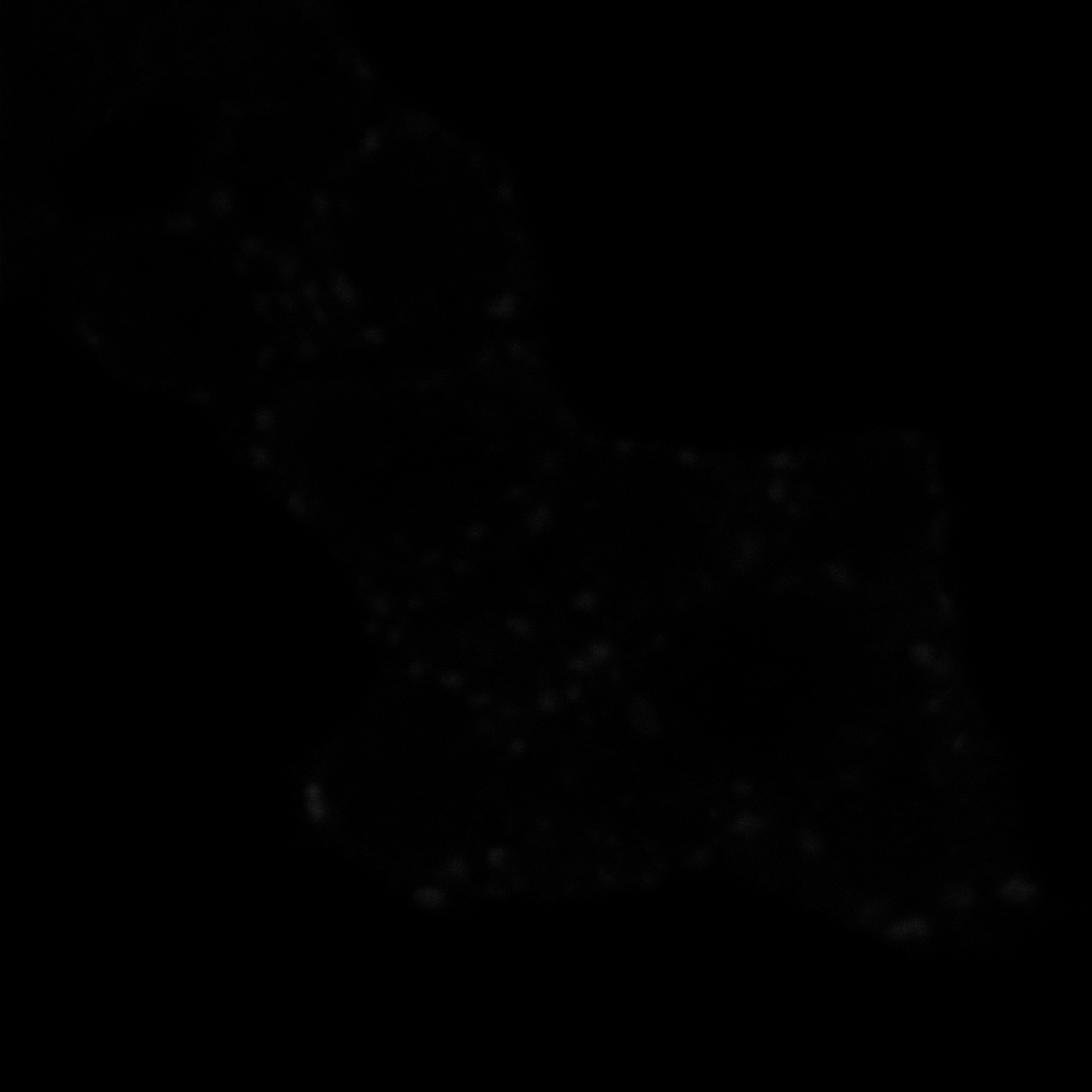

Supplement: Supplementary file 8 — Source data Fig. 3 [file 44318_2025_448_MOESM8_ESM.zip › Figure 3/Fig 3B/16.7 + aldometanib/Figure 3B 16.7 + aldometanib complete image.tif]

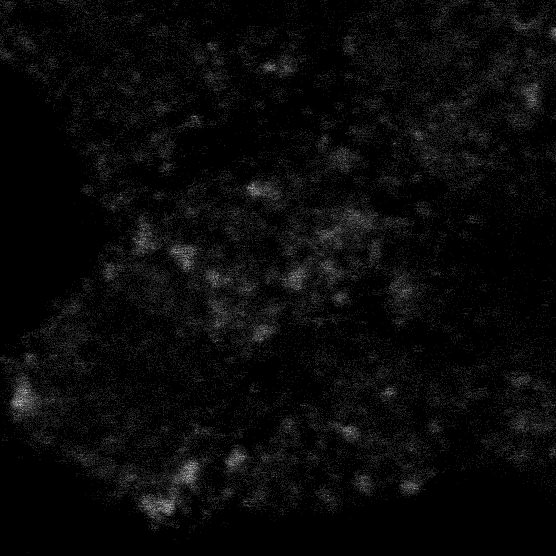

Supplement: Supplementary file 8 — Source data Fig. 3 [file 44318_2025_448_MOESM8_ESM.zip › Figure 3/Fig 3B/16.7 + aldometanib/Ins mrna.tif]

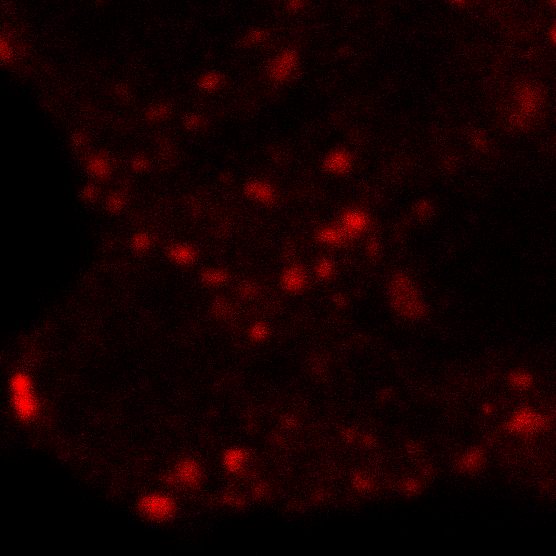

Supplement: Supplementary file 8 — Source data Fig. 3 [file 44318_2025_448_MOESM8_ESM.zip › Figure 3/Fig 3B/16.7 + aldometanib/eif3b.tif]

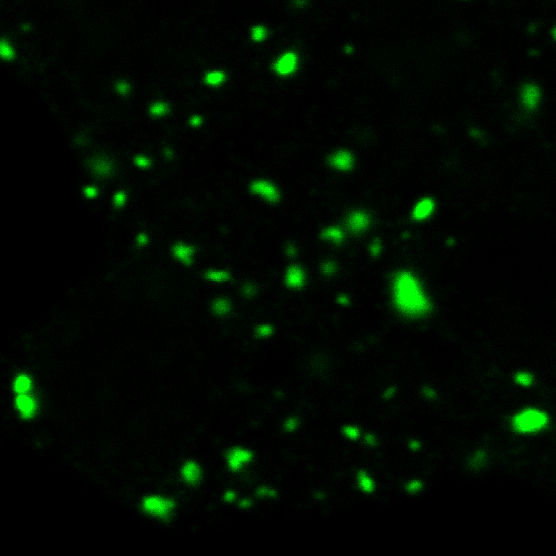

Supplement: Supplementary file 8 — Source data Fig. 3 [file 44318_2025_448_MOESM8_ESM.zip › Figure 3/Fig 3B/16.7 + aldometanib/g3bp1.tif]

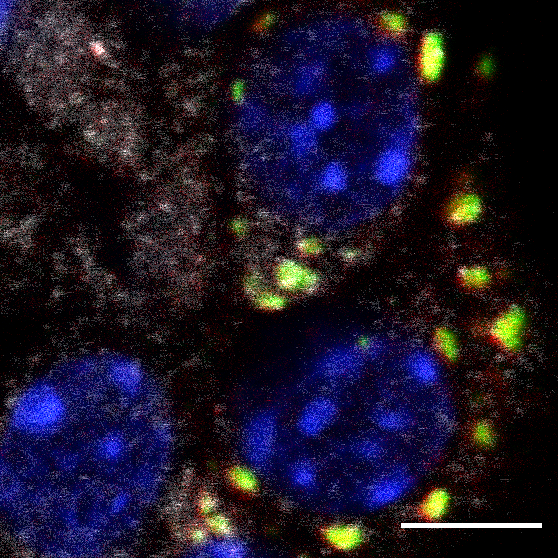

Supplement: Supplementary file 8 — Source data Fig. 3 [file 44318_2025_448_MOESM8_ESM.zip › Figure 3/Fig 3B/16.7 + aldometanib + pyruvate/Crop of Figure 3B 16.7 + aldometanib + pyruvate (RGB).tif]

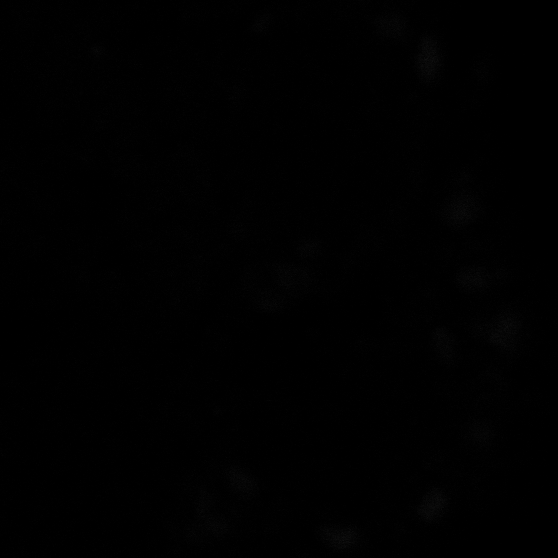

Supplement: Supplementary file 8 — Source data Fig. 3 [file 44318_2025_448_MOESM8_ESM.zip › Figure 3/Fig 3B/16.7 + aldometanib + pyruvate/Crop of Figure 3B 16.7 + aldometanib + pyruvate.tif]

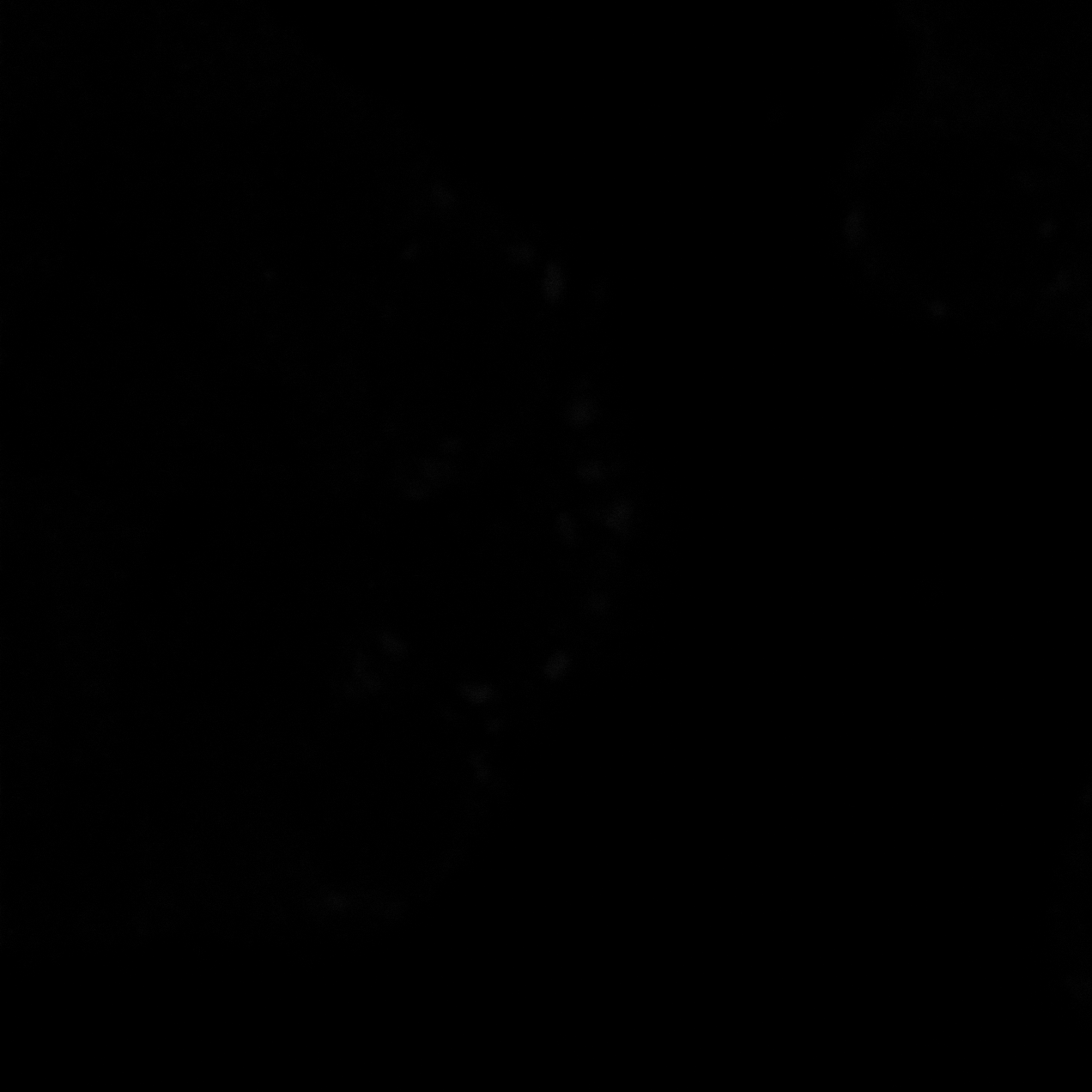

Supplement: Supplementary file 8 — Source data Fig. 3 [file 44318_2025_448_MOESM8_ESM.zip › Figure 3/Fig 3B/16.7 + aldometanib + pyruvate/Figure 3B 16.7 + aldometanib + pyruvate.tif]

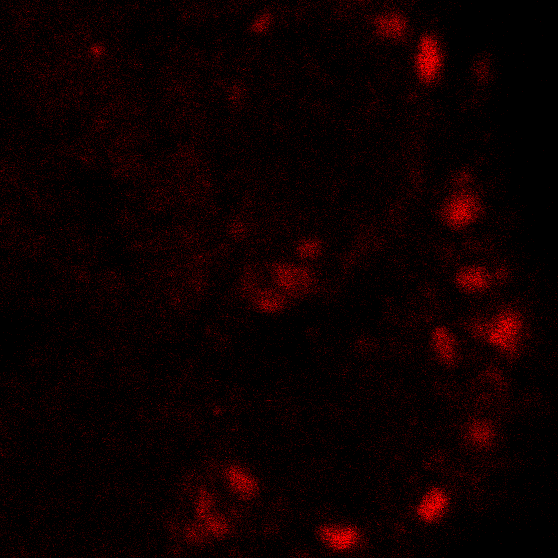

Supplement: Supplementary file 8 — Source data Fig. 3 [file 44318_2025_448_MOESM8_ESM.zip › Figure 3/Fig 3B/16.7 + aldometanib + pyruvate/eif3b.tif]

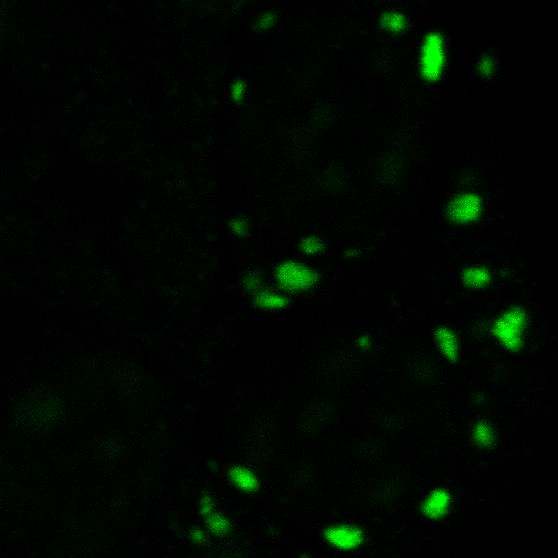

Supplement: Supplementary file 8 — Source data Fig. 3 [file 44318_2025_448_MOESM8_ESM.zip › Figure 3/Fig 3B/16.7 + aldometanib + pyruvate/g3bp1.tif]

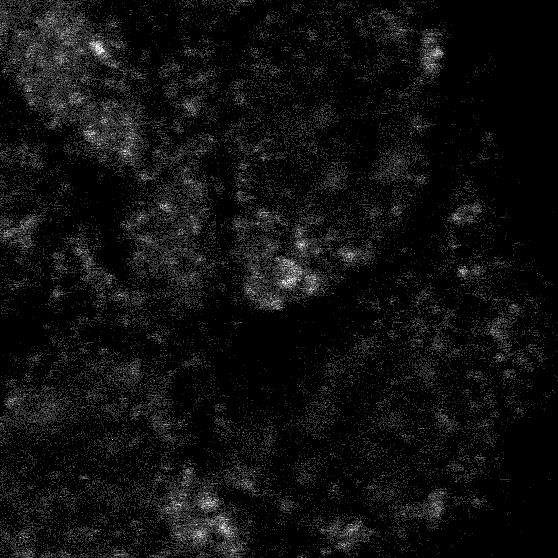

Supplement: Supplementary file 8 — Source data Fig. 3 [file 44318_2025_448_MOESM8_ESM.zip › Figure 3/Fig 3B/16.7 + aldometanib + pyruvate/ins mrna.tif]

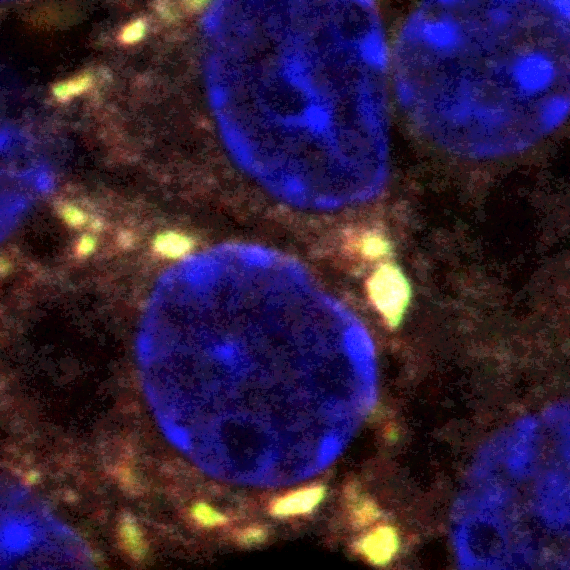

Supplement: Supplementary file 8 — Source data Fig. 3 [file 44318_2025_448_MOESM8_ESM.zip › Figure 3/Fig 3B/2.8/Crop of Figure 3B complete image.tif]

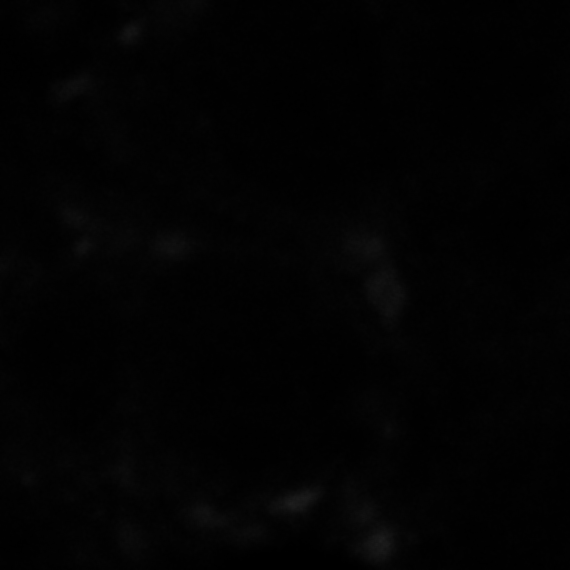

Supplement: Supplementary file 8 — Source data Fig. 3 [file 44318_2025_448_MOESM8_ESM.zip › Figure 3/Fig 3B/2.8/Figure 3B 2.8 complete image.tif]

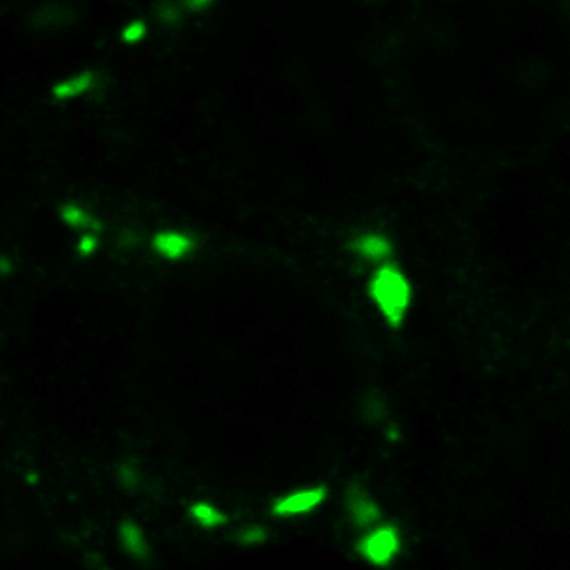

Supplement: Supplementary file 8 — Source data Fig. 3 [file 44318_2025_448_MOESM8_ESM.zip › Figure 3/Fig 3B/2.8/g3bp1.tif]

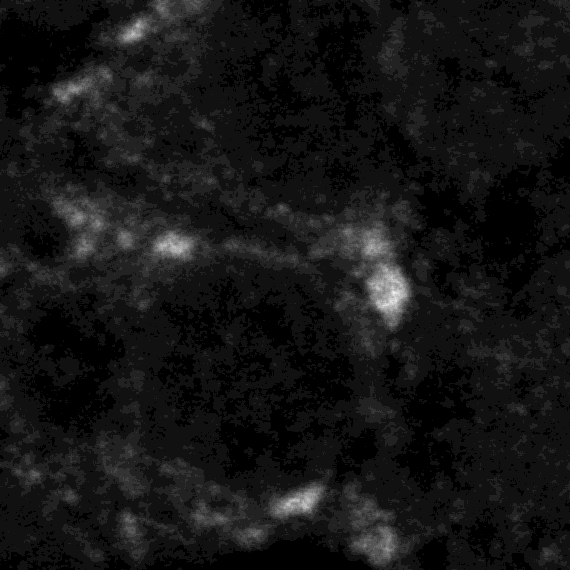

Supplement: Supplementary file 8 — Source data Fig. 3 [file 44318_2025_448_MOESM8_ESM.zip › Figure 3/Fig 3B/2.8/ins mrna.tif]

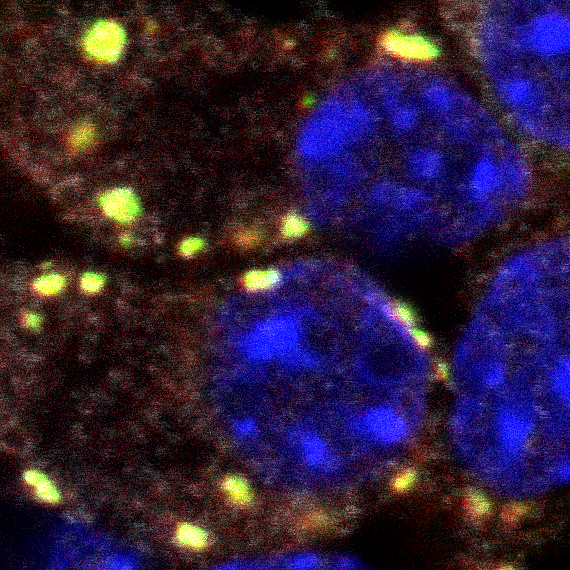

Supplement: Supplementary file 8 — Source data Fig. 3 [file 44318_2025_448_MOESM8_ESM.zip › Figure 3/Fig 3B/2.8 + DMSO/Crop of Figure 3B 2.8 DMSO complete image (RGB).tif]

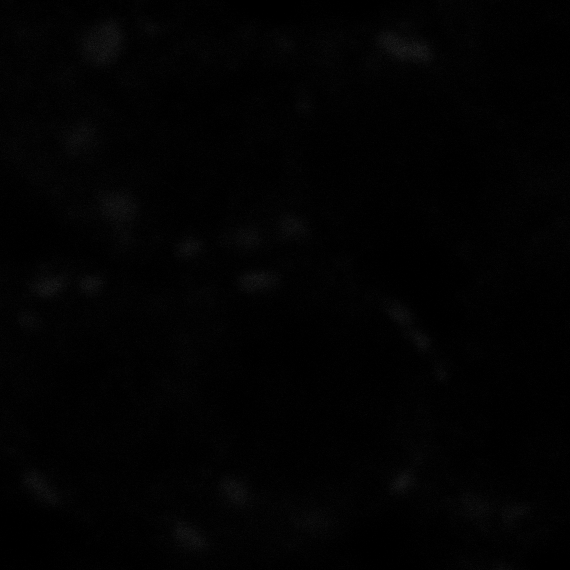

Supplement: Supplementary file 8 — Source data Fig. 3 [file 44318_2025_448_MOESM8_ESM.zip › Figure 3/Fig 3B/2.8 + DMSO/Crop of Figure 3B 2.8 DMSO complete image.tif]

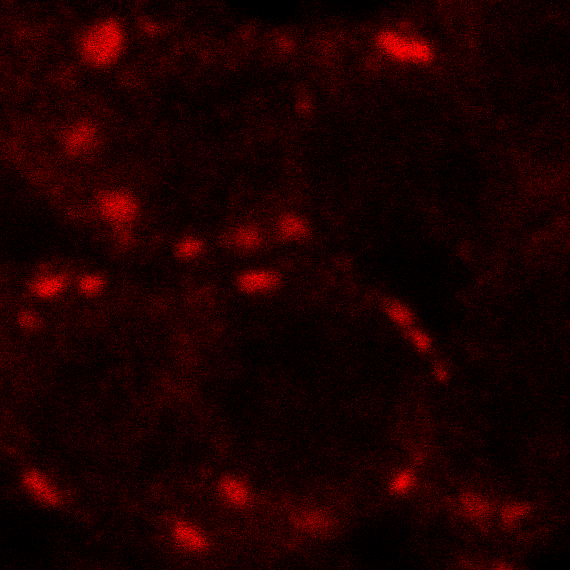

Supplement: Supplementary file 8 — Source data Fig. 3 [file 44318_2025_448_MOESM8_ESM.zip › Figure 3/Fig 3B/2.8 + DMSO/eif3b.tif]

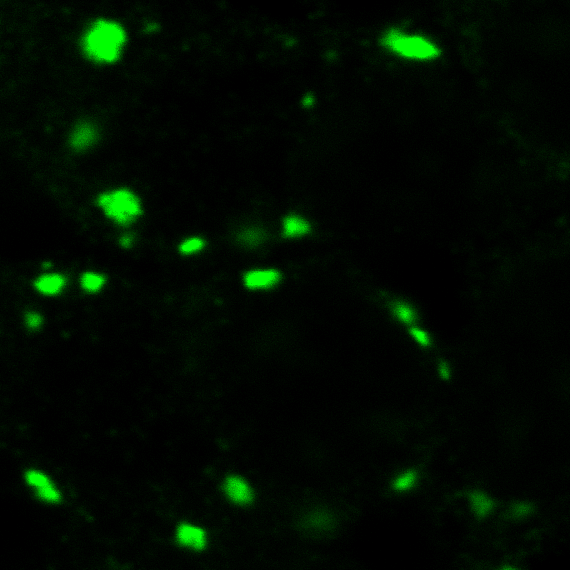

Supplement: Supplementary file 8 — Source data Fig. 3 [file 44318_2025_448_MOESM8_ESM.zip › Figure 3/Fig 3B/2.8 + DMSO/g3bp1.tif]

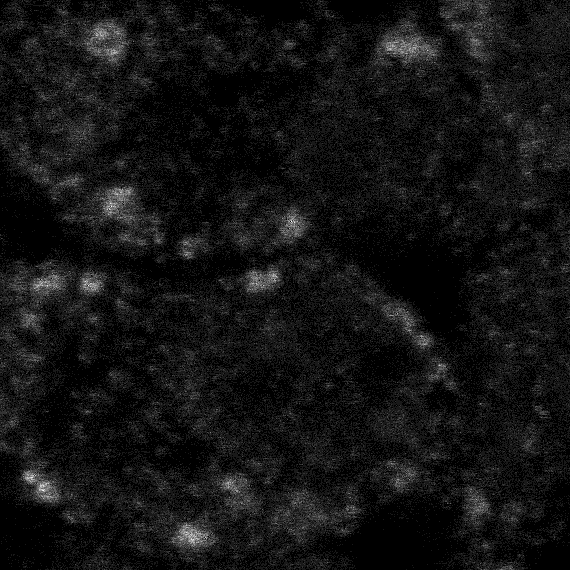

Supplement: Supplementary file 8 — Source data Fig. 3 [file 44318_2025_448_MOESM8_ESM.zip › Figure 3/Fig 3B/2.8 + DMSO/ins mrna.tif]

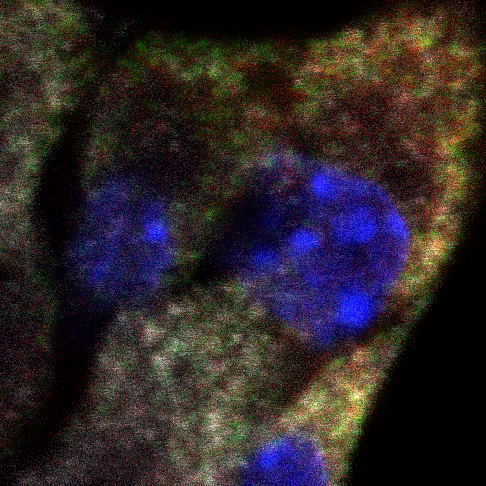

Supplement: Supplementary file 8 — Source data Fig. 3 [file 44318_2025_448_MOESM8_ESM.zip › Figure 3/Fig 3B/2.8 + Pyruvate/Crop of Figure 3B 2.8 + Pyruvate complete image (RGB).tif]

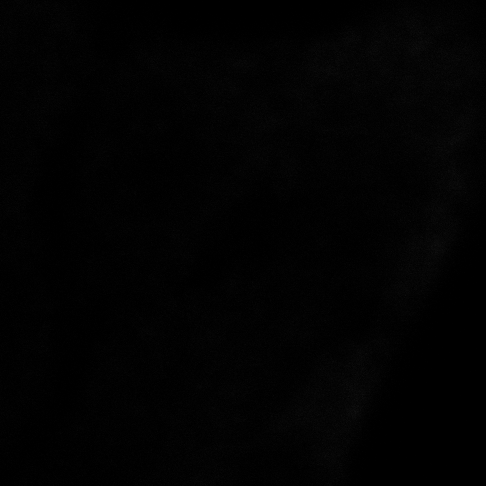

Supplement: Supplementary file 8 — Source data Fig. 3 [file 44318_2025_448_MOESM8_ESM.zip › Figure 3/Fig 3B/2.8 + Pyruvate/Crop of Figure 3B 2.8 + Pyruvate complete image.tif]

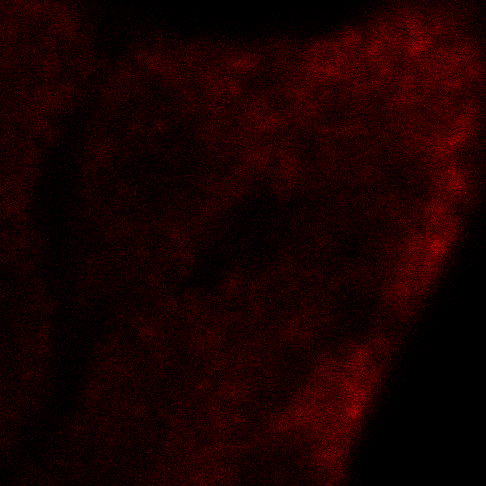

Supplement: Supplementary file 8 — Source data Fig. 3 [file 44318_2025_448_MOESM8_ESM.zip › Figure 3/Fig 3B/2.8 + Pyruvate/eif3b.tif]

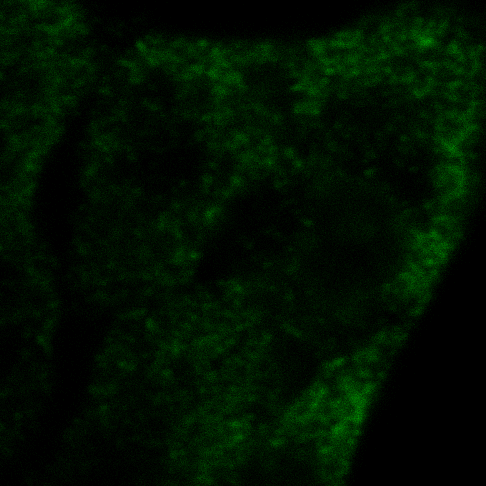

Supplement: Supplementary file 8 — Source data Fig. 3 [file 44318_2025_448_MOESM8_ESM.zip › Figure 3/Fig 3B/2.8 + Pyruvate/g3bp1.tif]

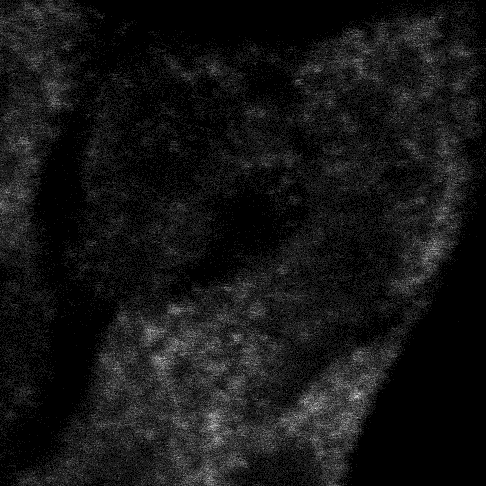

Supplement: Supplementary file 8 — Source data Fig. 3 [file 44318_2025_448_MOESM8_ESM.zip › Figure 3/Fig 3B/2.8 + Pyruvate/ins mrna.tif]

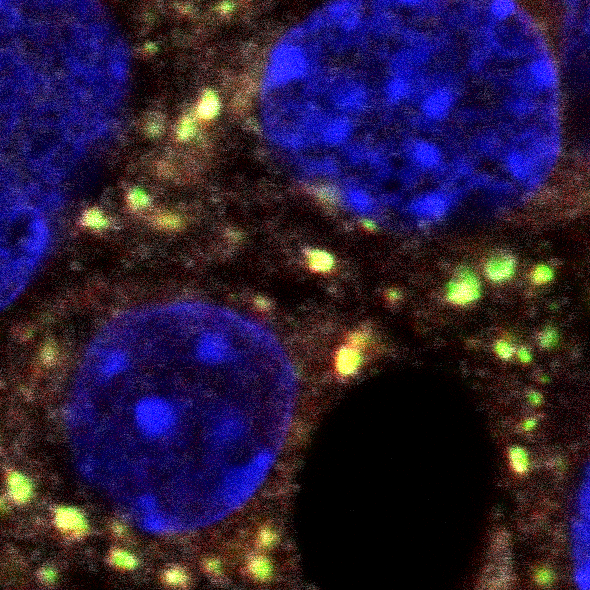

Supplement: Supplementary file 8 — Source data Fig. 3 [file 44318_2025_448_MOESM8_ESM.zip › Figure 3/Fig 3B/2.8 + aldometanib/Crop of Figure 3B 2.8 + aldometanib (RGB).tif]

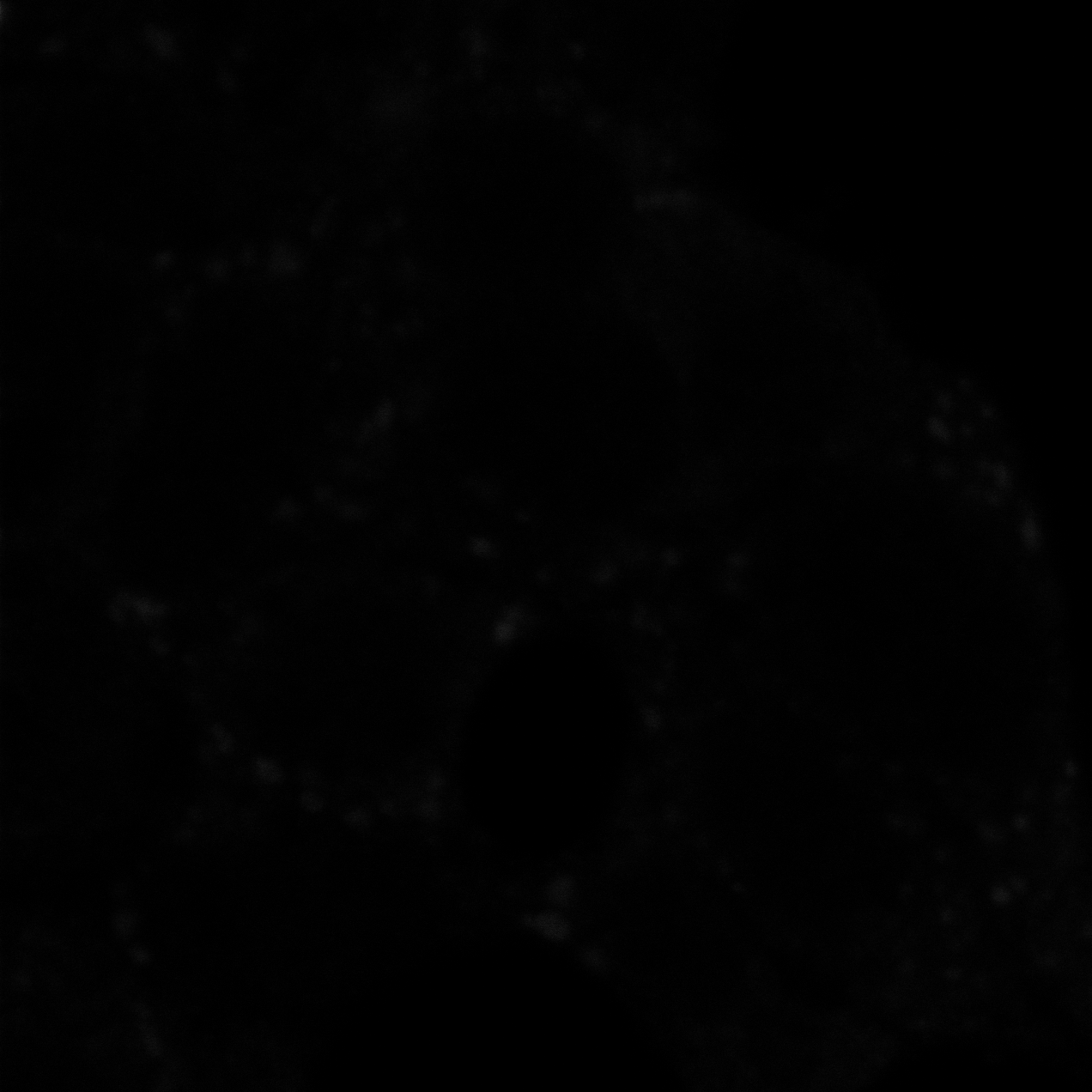

Supplement: Supplementary file 8 — Source data Fig. 3 [file 44318_2025_448_MOESM8_ESM.zip › Figure 3/Fig 3B/2.8 + aldometanib/Figure 3B 2.8 + aldometanib.tif]

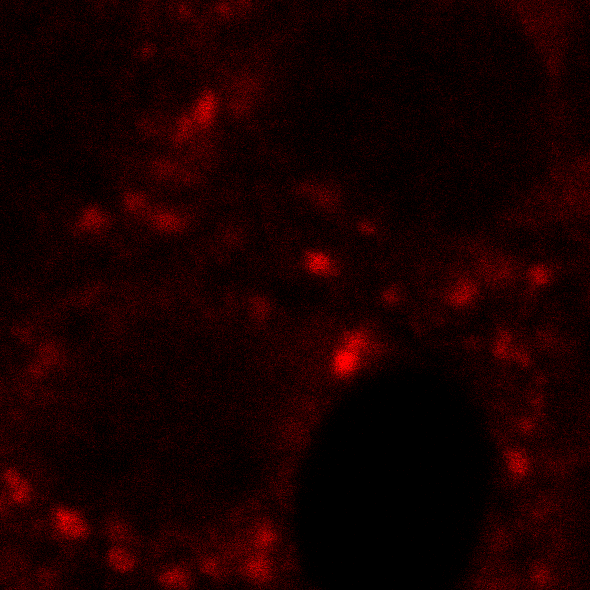

Supplement: Supplementary file 8 — Source data Fig. 3 [file 44318_2025_448_MOESM8_ESM.zip › Figure 3/Fig 3B/2.8 + aldometanib/eif3b.tif]

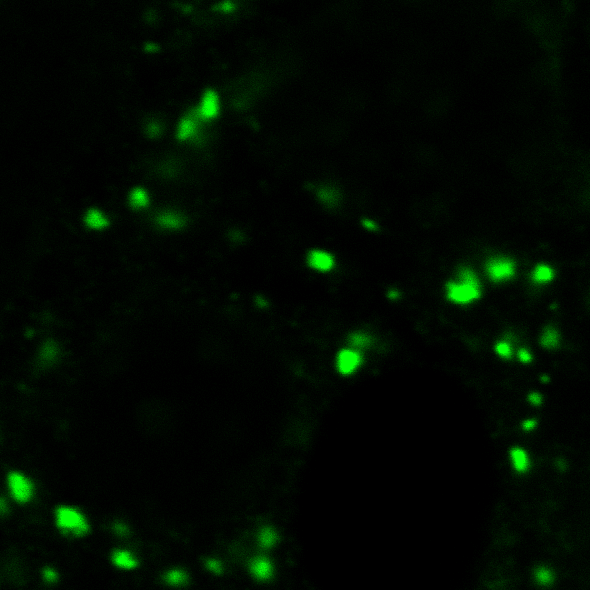

Supplement: Supplementary file 8 — Source data Fig. 3 [file 44318_2025_448_MOESM8_ESM.zip › Figure 3/Fig 3B/2.8 + aldometanib/g3bp1.tif]

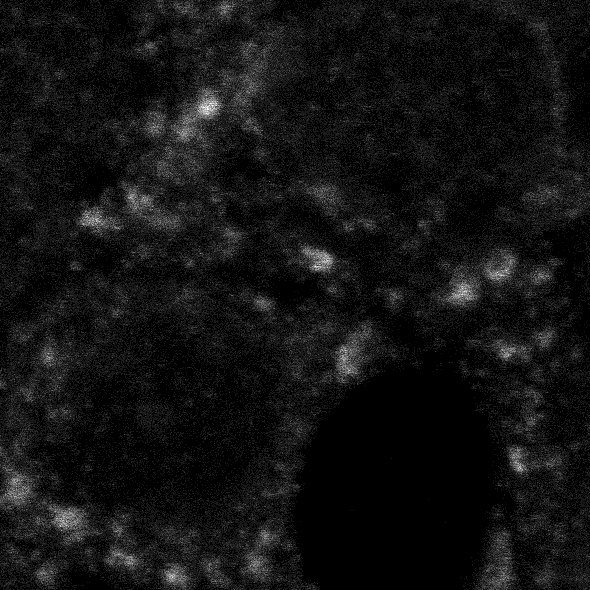

Supplement: Supplementary file 8 — Source data Fig. 3 [file 44318_2025_448_MOESM8_ESM.zip › Figure 3/Fig 3B/2.8 + aldometanib/ins mrna.tif]

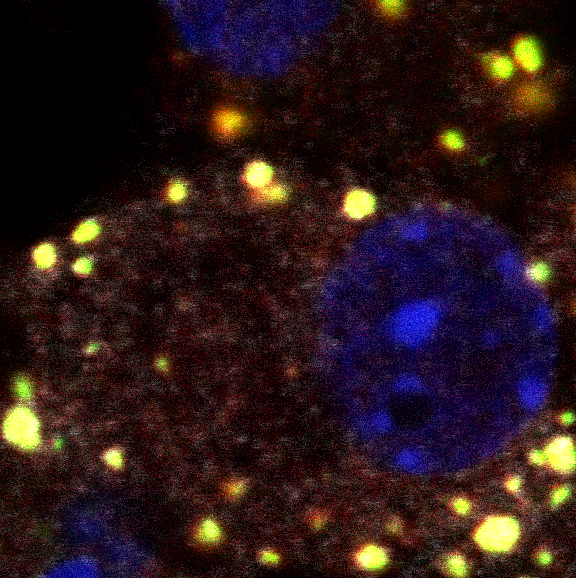

Supplement: Supplementary file 8 — Source data Fig. 3 [file 44318_2025_448_MOESM8_ESM.zip › Figure 3/Fig 3B/2.8 + aldometanib + pyruvate/Crop of Figure 3B 2.8 + aldometanib + pyruvate complete image (RGB).tif]

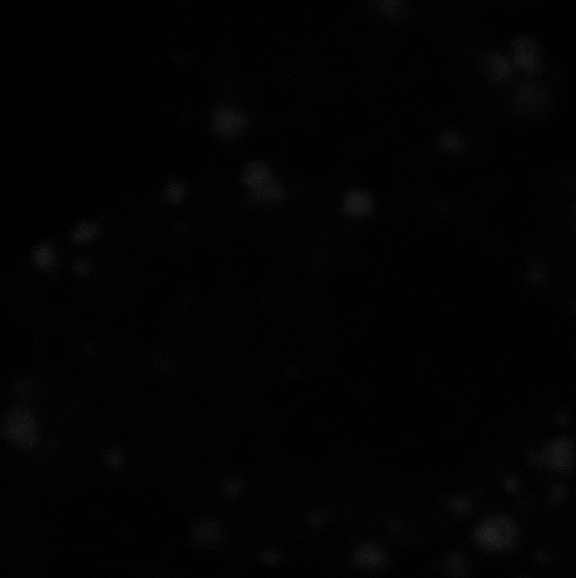

Supplement: Supplementary file 8 — Source data Fig. 3 [file 44318_2025_448_MOESM8_ESM.zip › Figure 3/Fig 3B/2.8 + aldometanib + pyruvate/Crop of Figure 3B 2.8 + aldometanib + pyruvate complete image.tif]

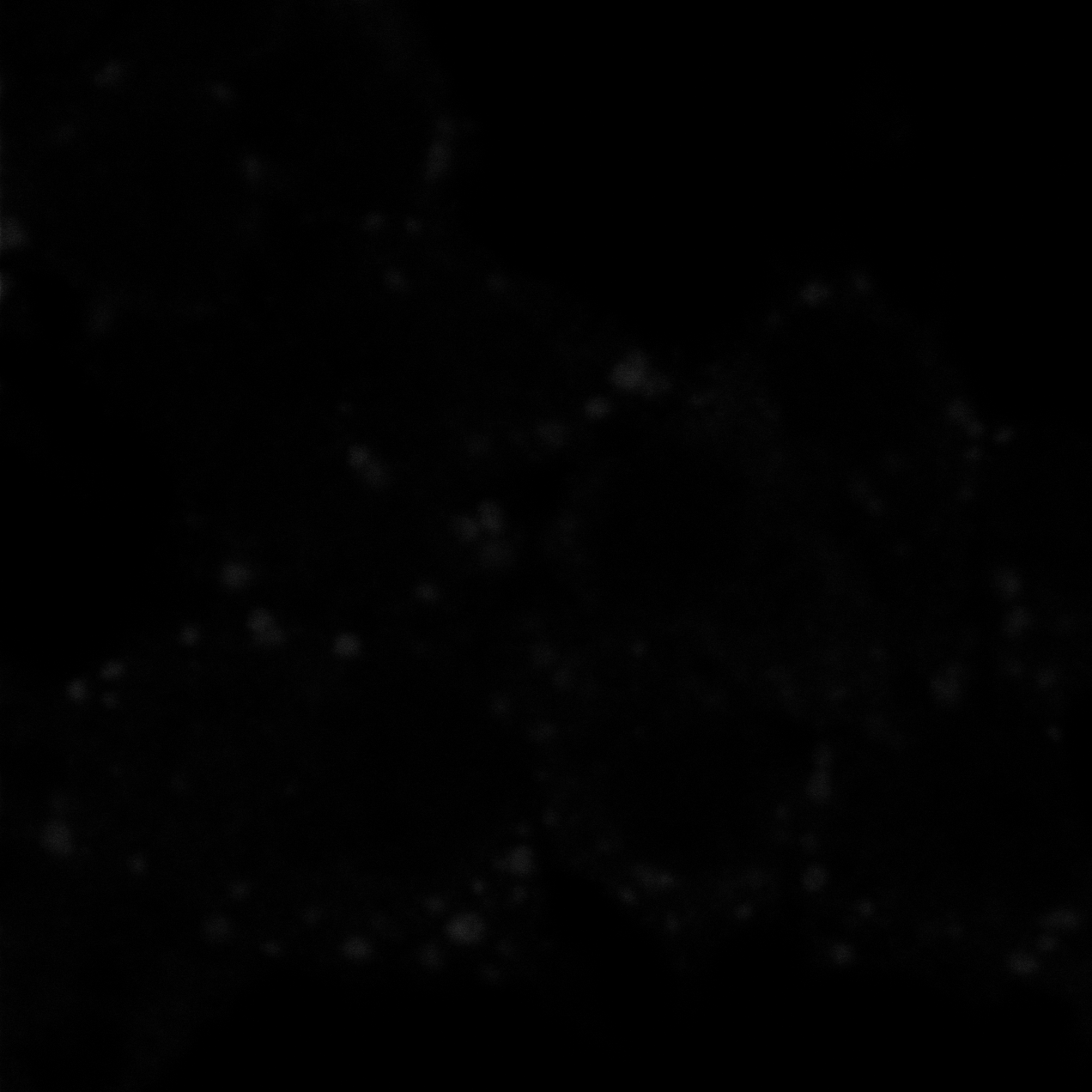

Supplement: Supplementary file 8 — Source data Fig. 3 [file 44318_2025_448_MOESM8_ESM.zip › Figure 3/Fig 3B/2.8 + aldometanib + pyruvate/Figure 3B 2.8 + aldometanib + pyruvate complete image.tif]

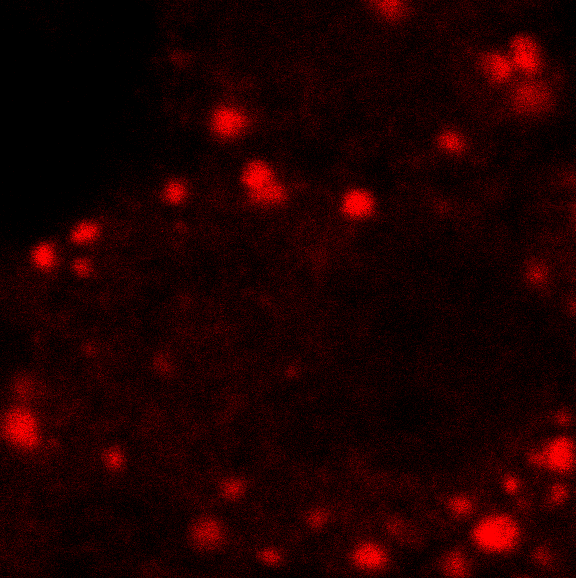

Supplement: Supplementary file 8 — Source data Fig. 3 [file 44318_2025_448_MOESM8_ESM.zip › Figure 3/Fig 3B/2.8 + aldometanib + pyruvate/eif3b.tif]

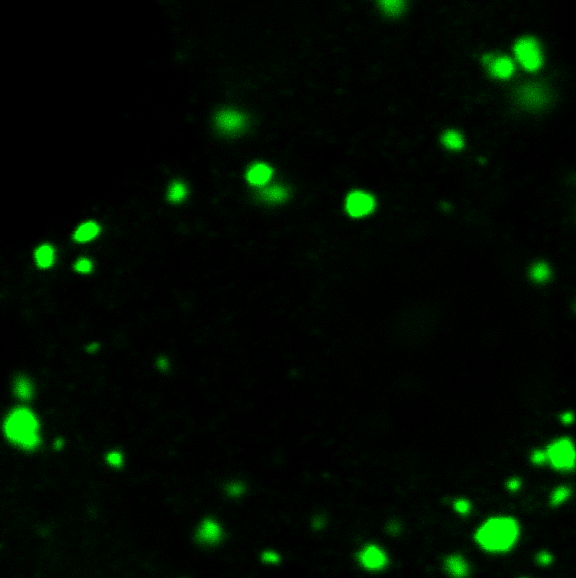

Supplement: Supplementary file 8 — Source data Fig. 3 [file 44318_2025_448_MOESM8_ESM.zip › Figure 3/Fig 3B/2.8 + aldometanib + pyruvate/g3bp1.tif]

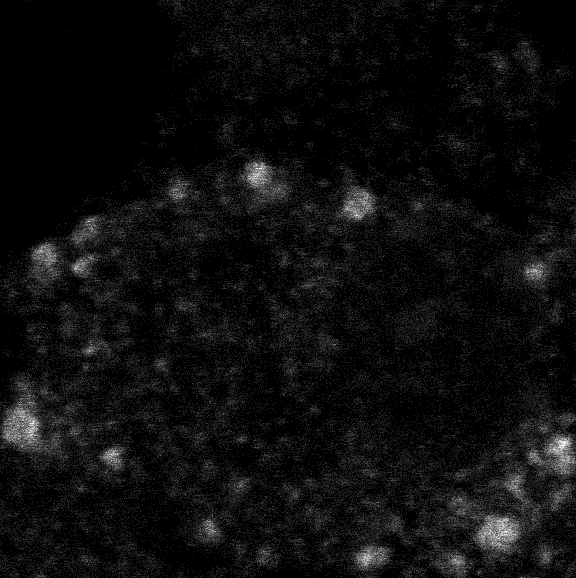

Supplement: Supplementary file 8 — Source data Fig. 3 [file 44318_2025_448_MOESM8_ESM.zip › Figure 3/Fig 3B/2.8 + aldometanib + pyruvate/ins mrna.tif]

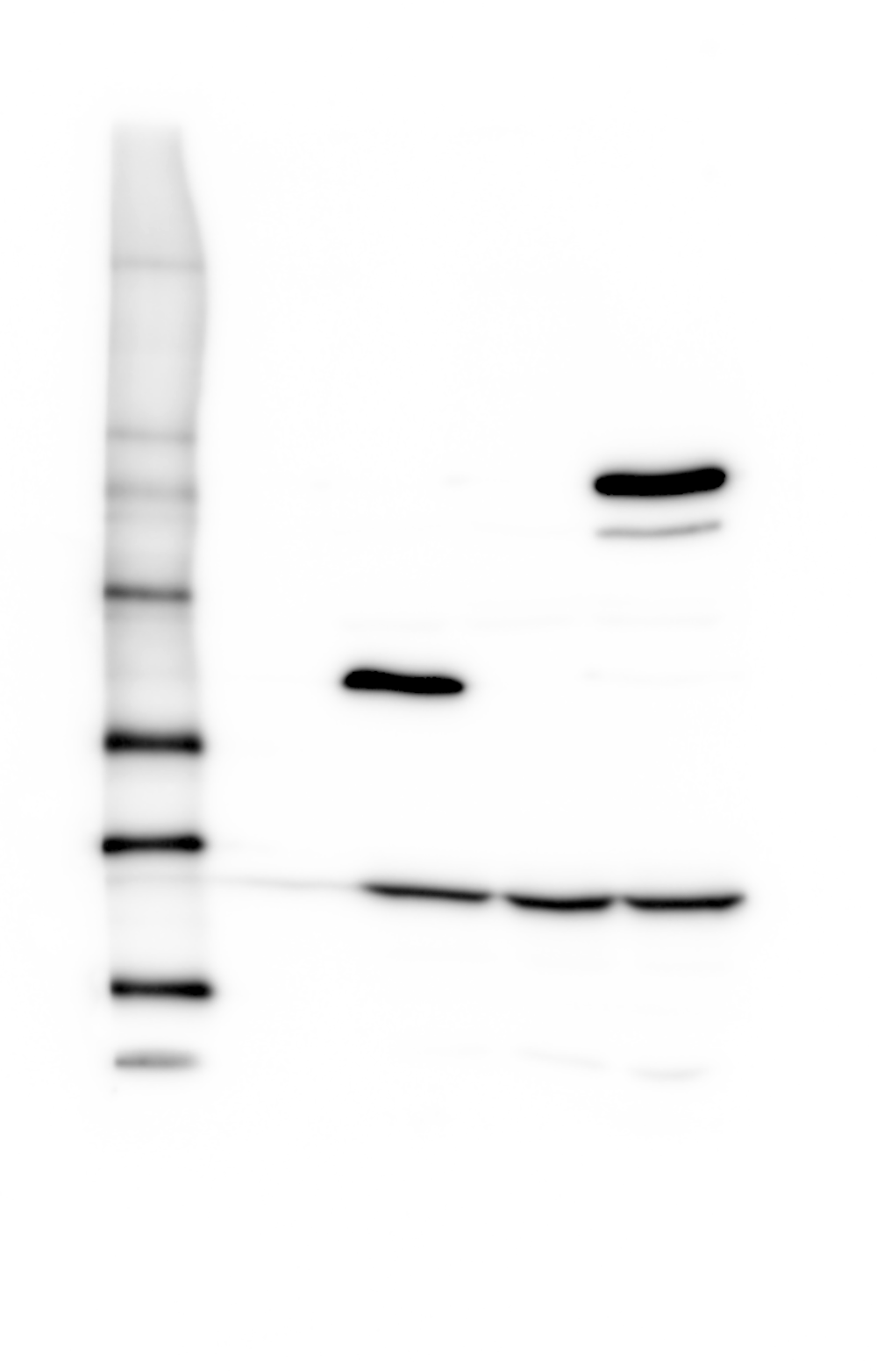

Supplement: Supplementary file 9 — Source data Fig. 4 [file 44318_2025_448_MOESM9_ESM.zip › Figure 4/Fig 4J/mCherry-hG3BP1, G3BP1 and g tubulin signal for Fig 4J.tiff]

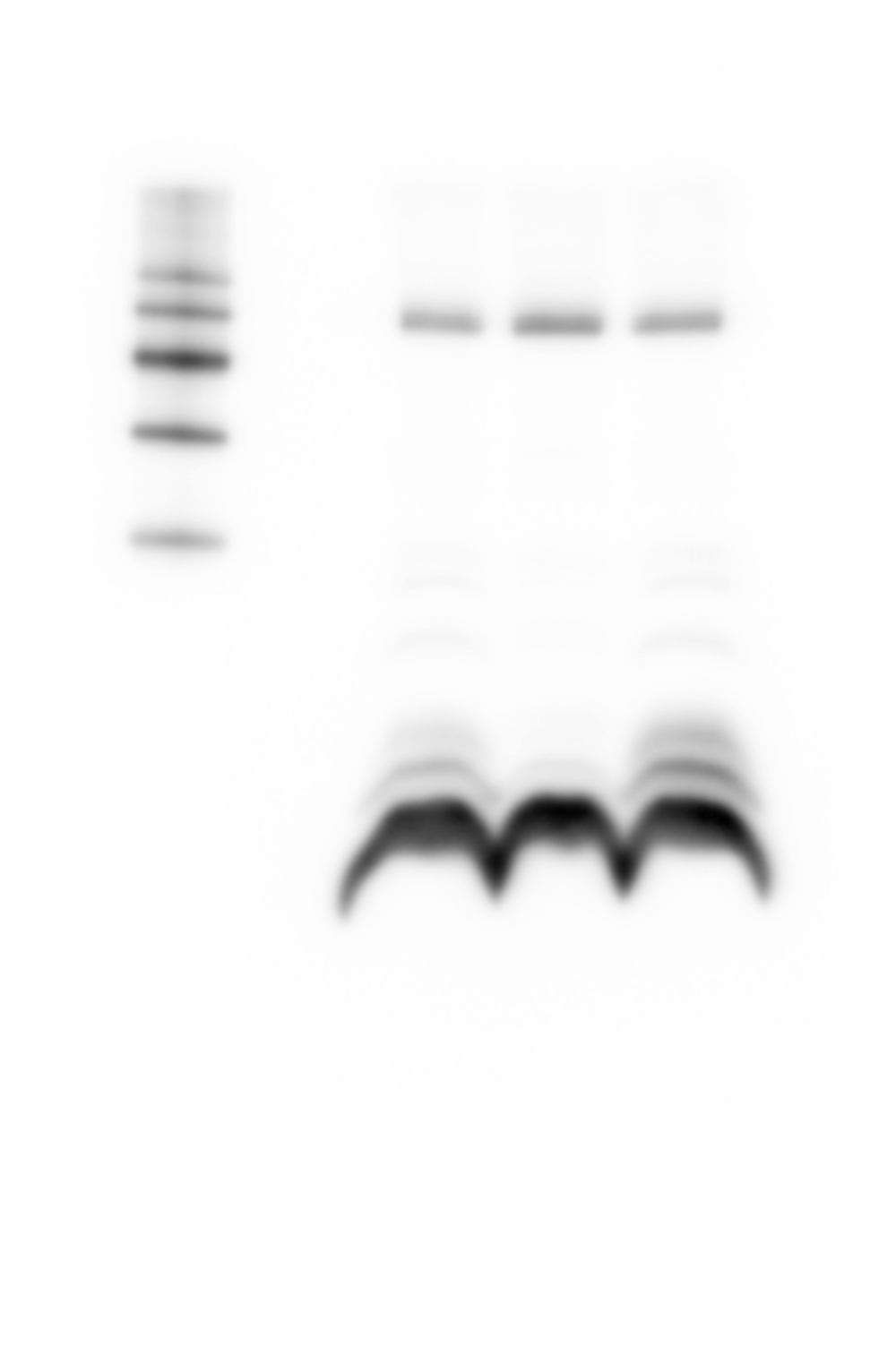

Supplement: Supplementary file 9 — Source data Fig. 4 [file 44318_2025_448_MOESM9_ESM.zip › Figure 4/Fig 4J/Insulin, Proinsulin signal for Figure 4J.tiff]

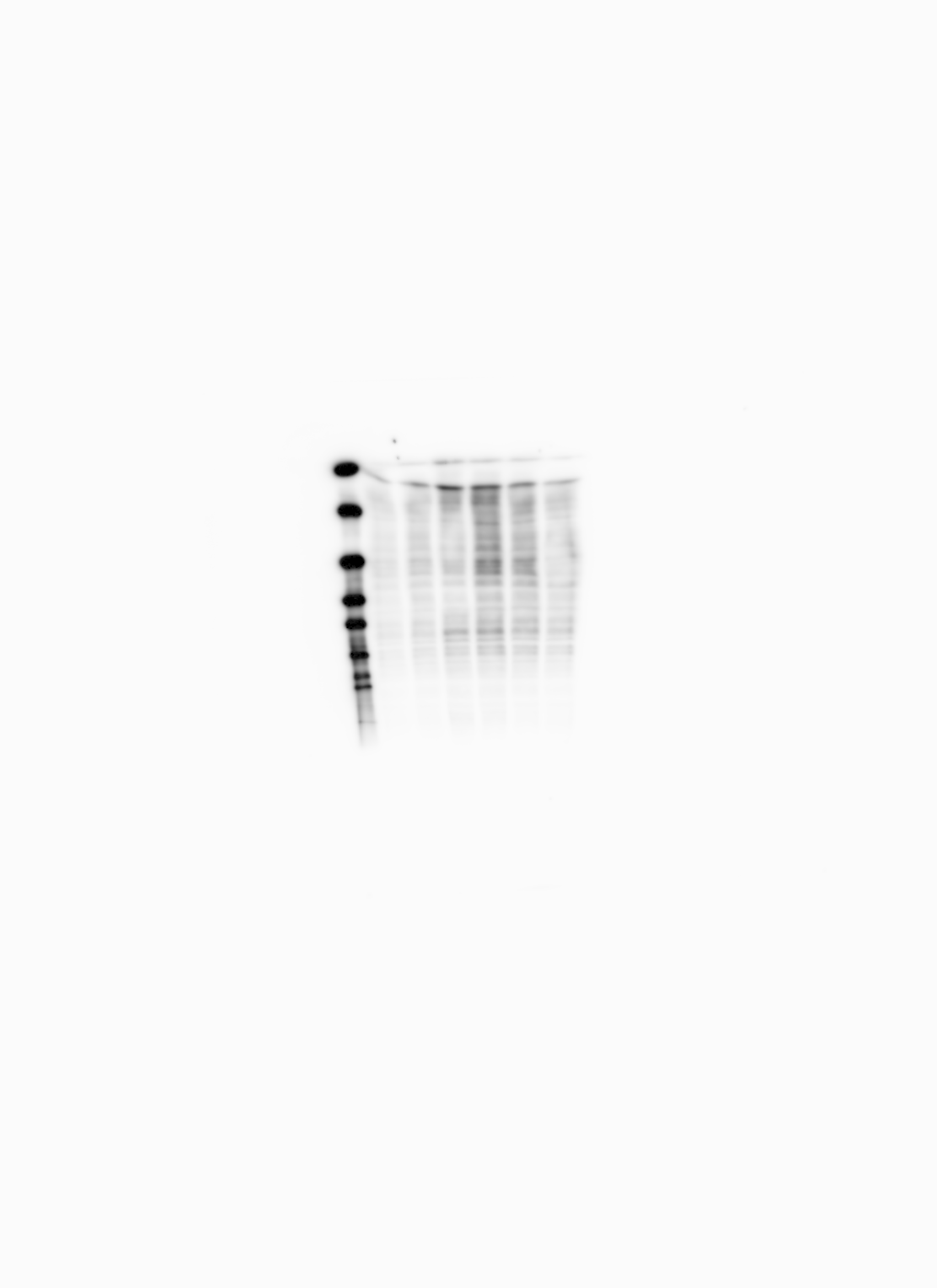

Supplement: Supplementary file 9 — Source data Fig. 4 [file 44318_2025_448_MOESM9_ESM.zip › Figure 4/Fig 4L/Puromycin exp Fig 4L.tif]

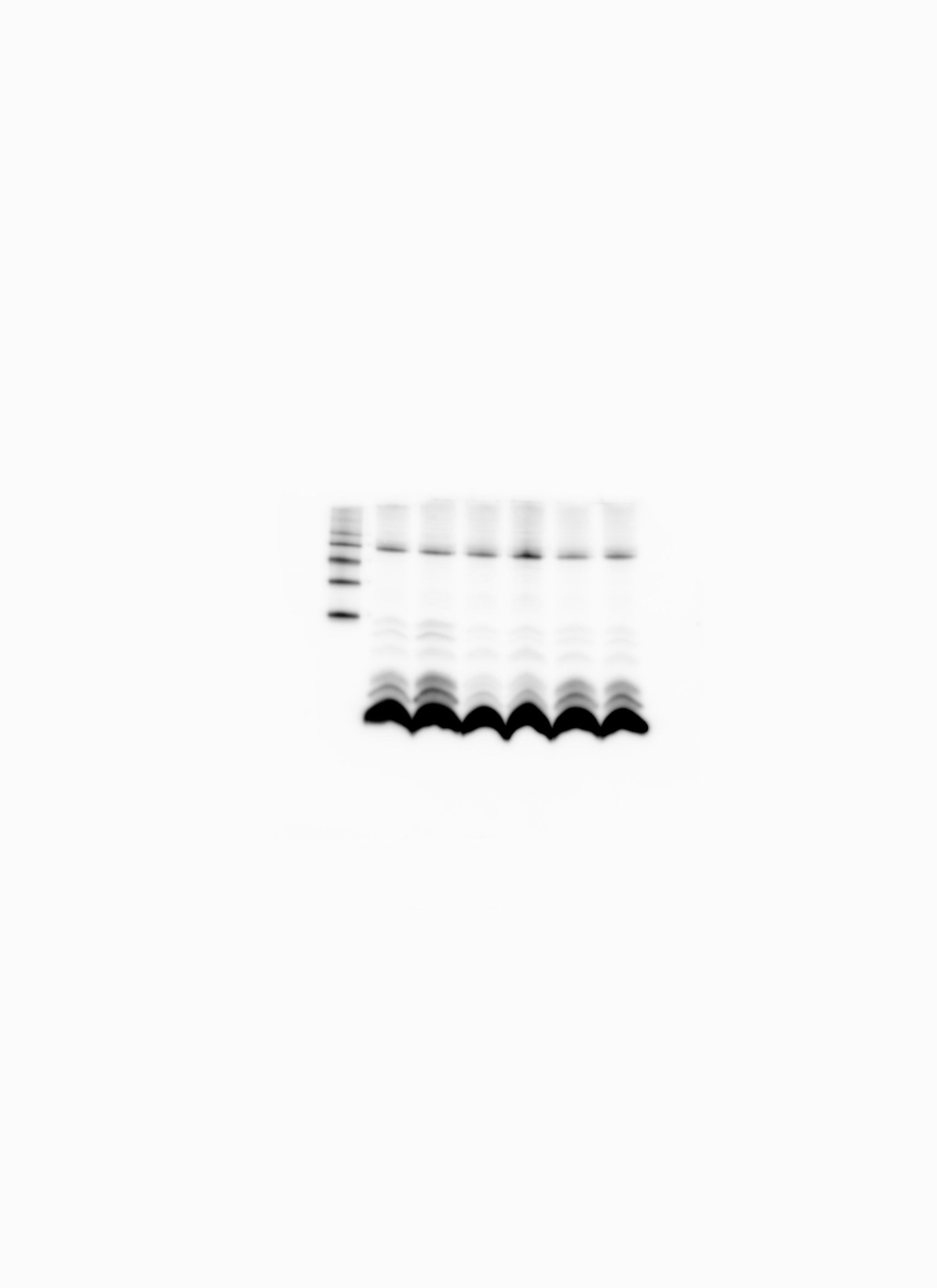

Supplement: Supplementary file 9 — Source data Fig. 4 [file 44318_2025_448_MOESM9_ESM.zip › Figure 4/Fig 4I/Proinsulin and g tub signal Figure 4I.tif]

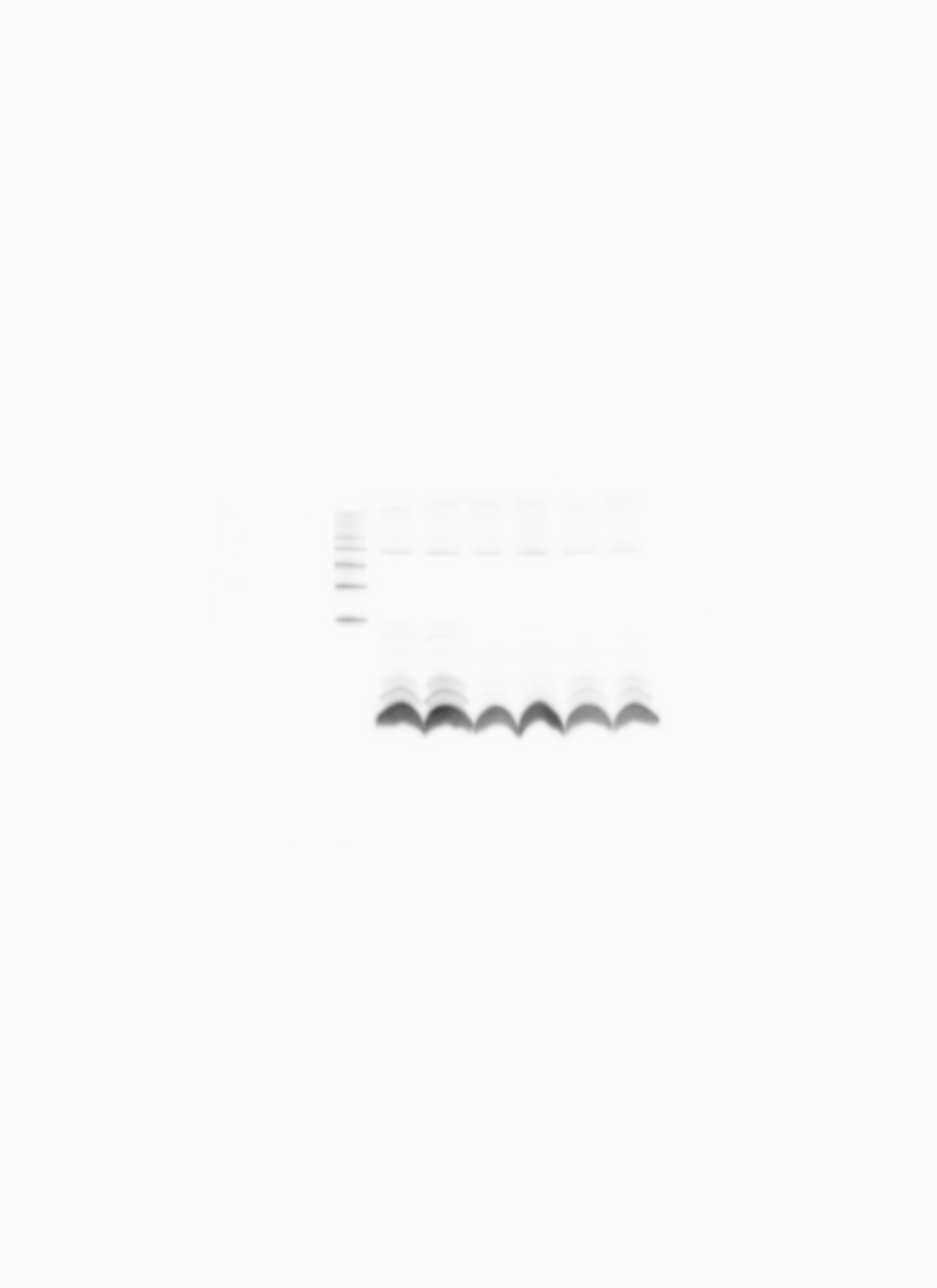

Supplement: Supplementary file 9 — Source data Fig. 4 [file 44318_2025_448_MOESM9_ESM.zip › Figure 4/Fig 4I/Insulin signal in Figure 4I.tif]

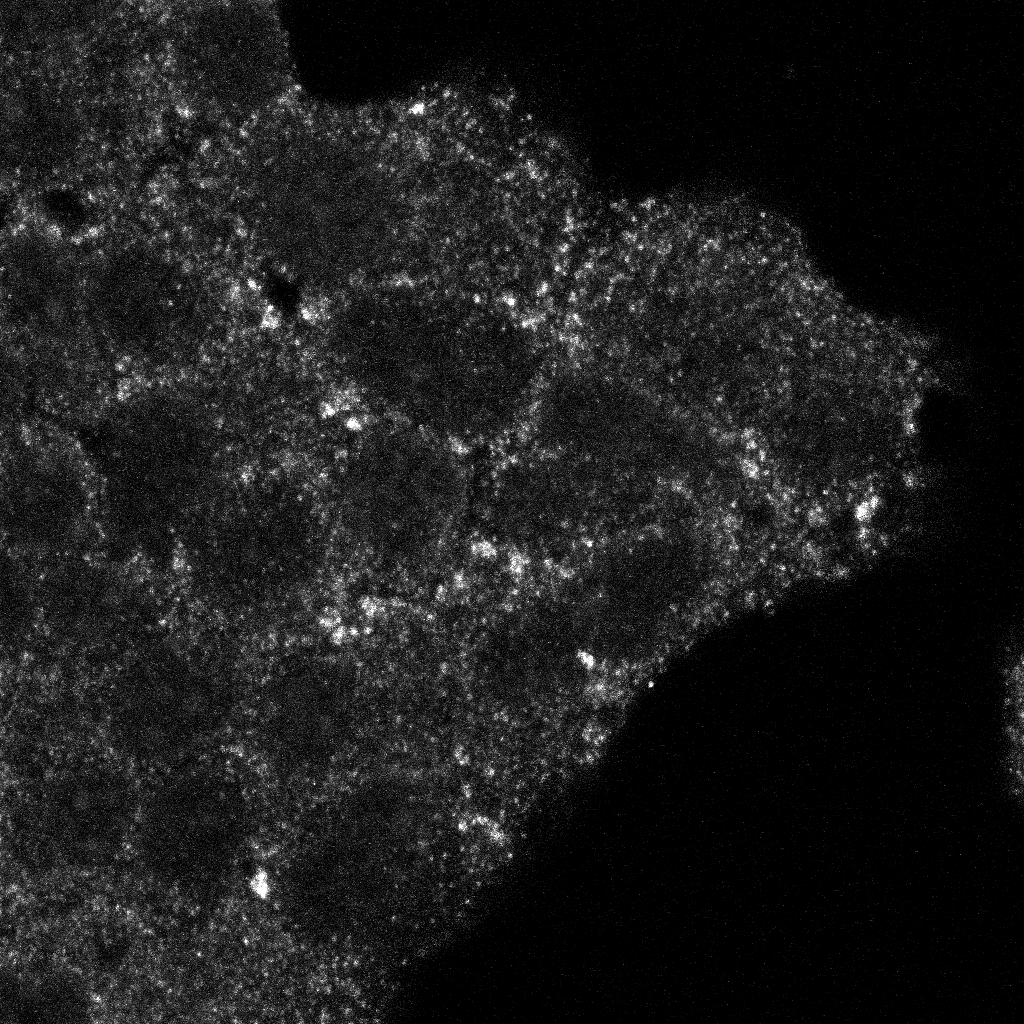

Supplement: Supplementary file 9 — Source data Fig. 4 [file 44318_2025_448_MOESM9_ESM.zip › Figure 4/Fig 4C/WT 2.8 (G3BP1 signal)/Composite2.tif]

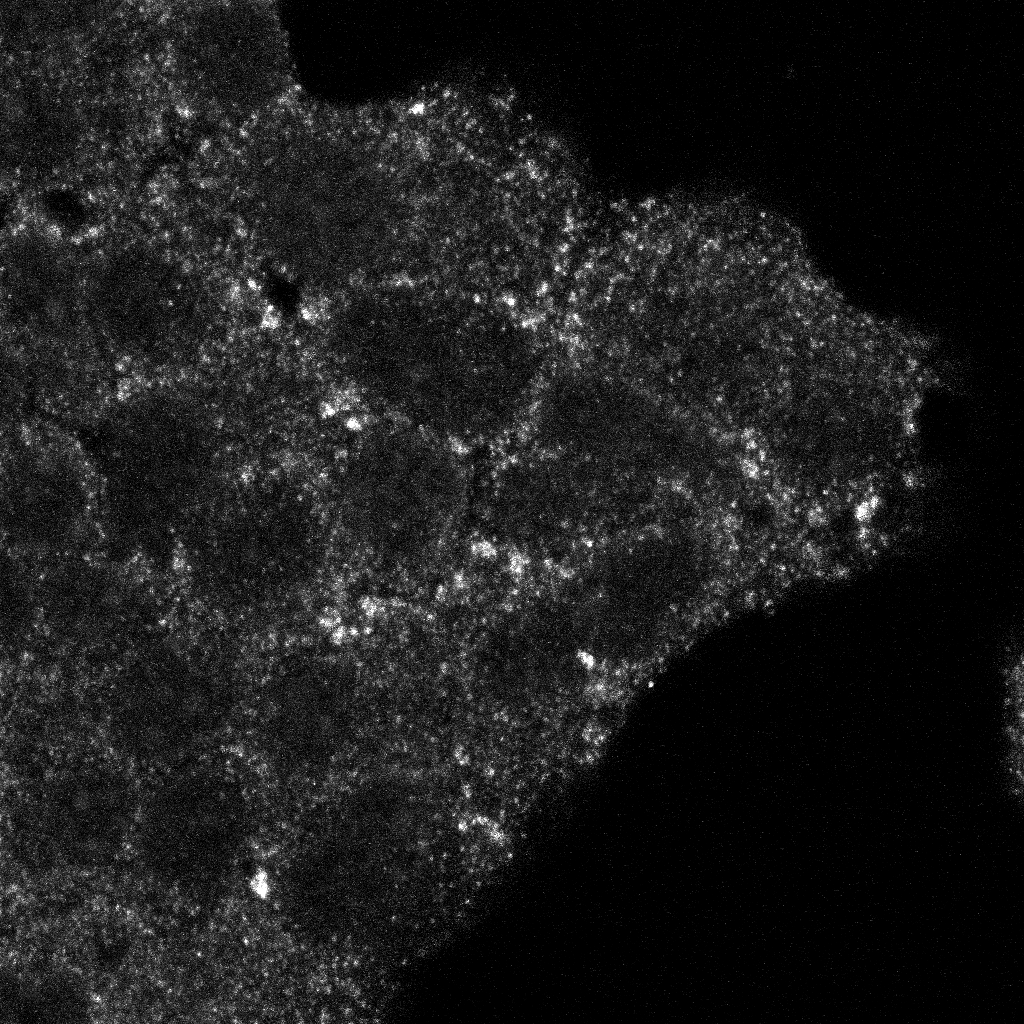

Supplement: Supplementary file 9 — Source data Fig. 4 [file 44318_2025_448_MOESM9_ESM.zip › Figure 4/Fig 4C/WT 2.8 (G3BP1 signal)/Composite.tif]

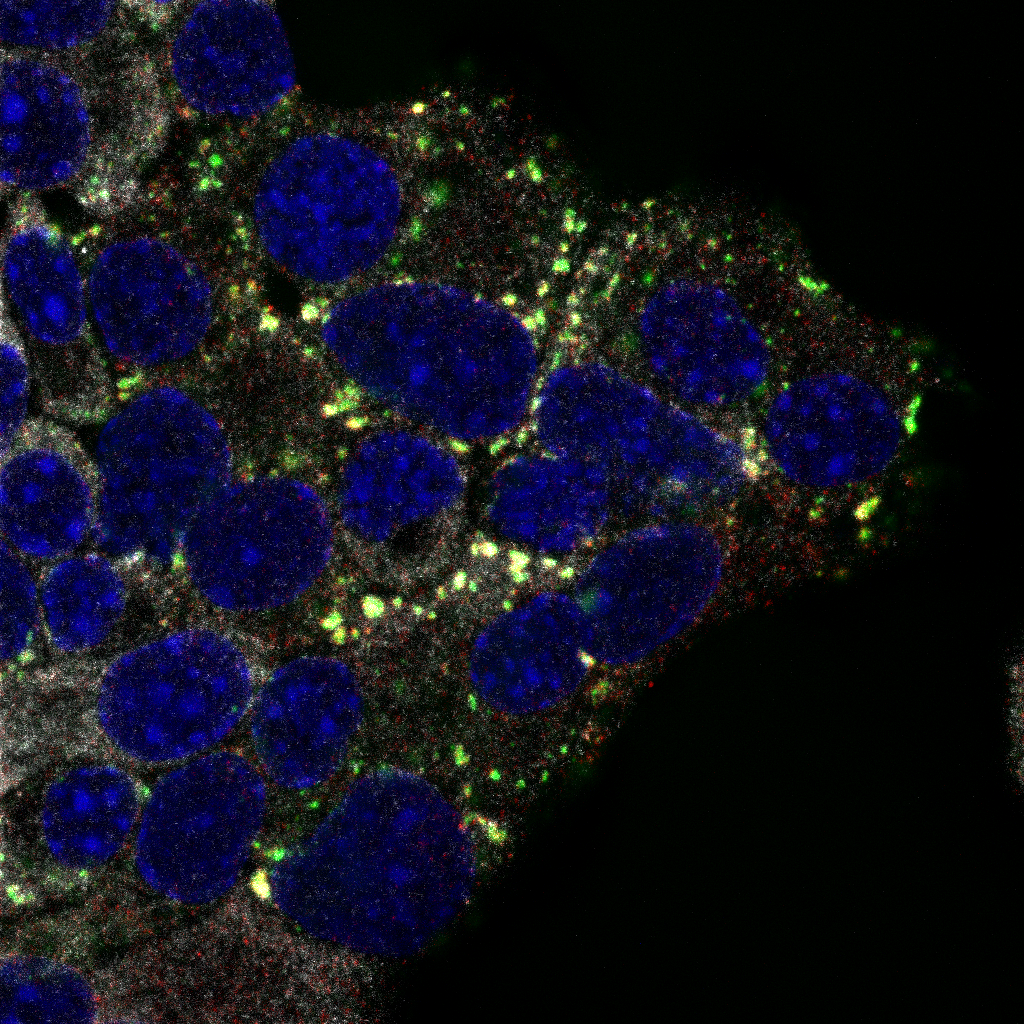

Supplement: Supplementary file 9 — Source data Fig. 4 [file 44318_2025_448_MOESM9_ESM.zip › Figure 4/Fig 4C/WT 2.8 (G3BP1 signal)/Composite2.tif (RGB).tif]

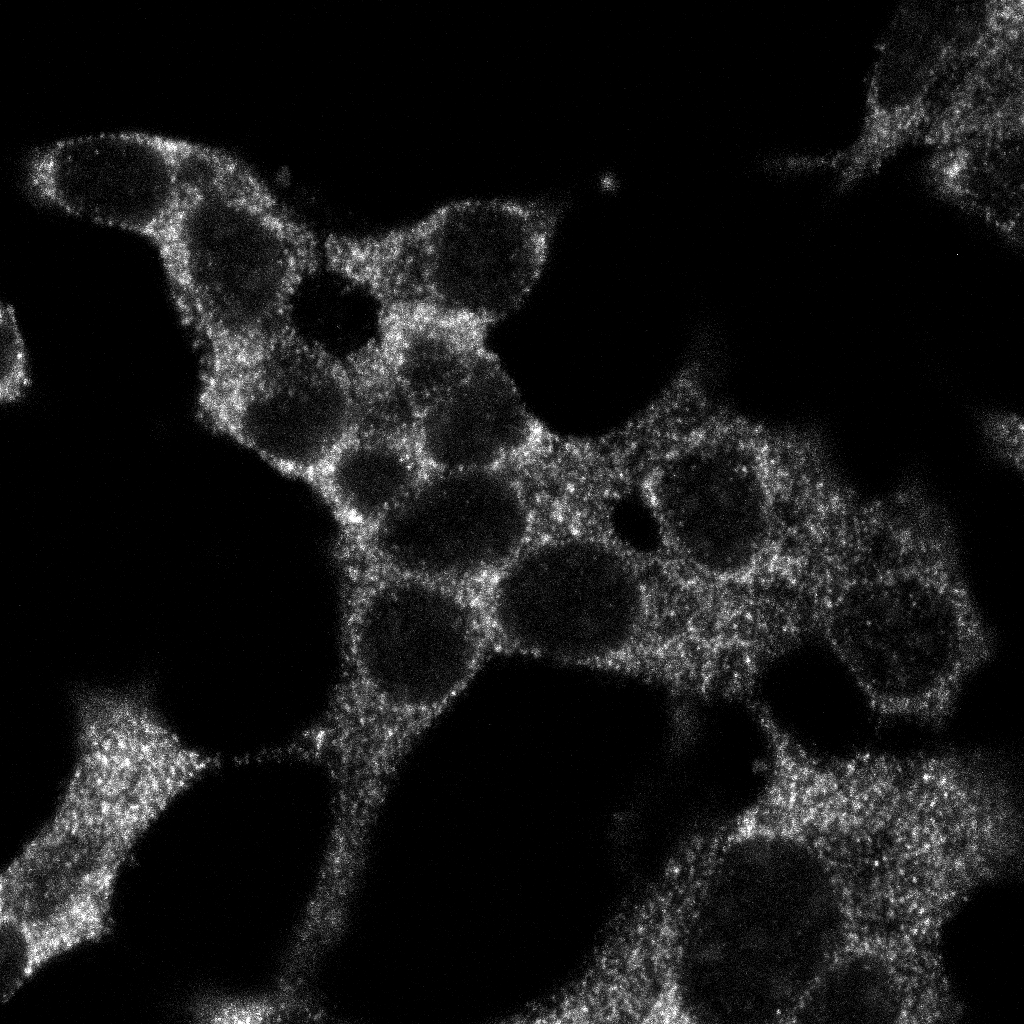

Supplement: Supplementary file 9 — Source data Fig. 4 [file 44318_2025_448_MOESM9_ESM.zip › Figure 4/Fig 4C/G3BP1 KO 2.8 (G3BP1 signal)/Composite.tif]

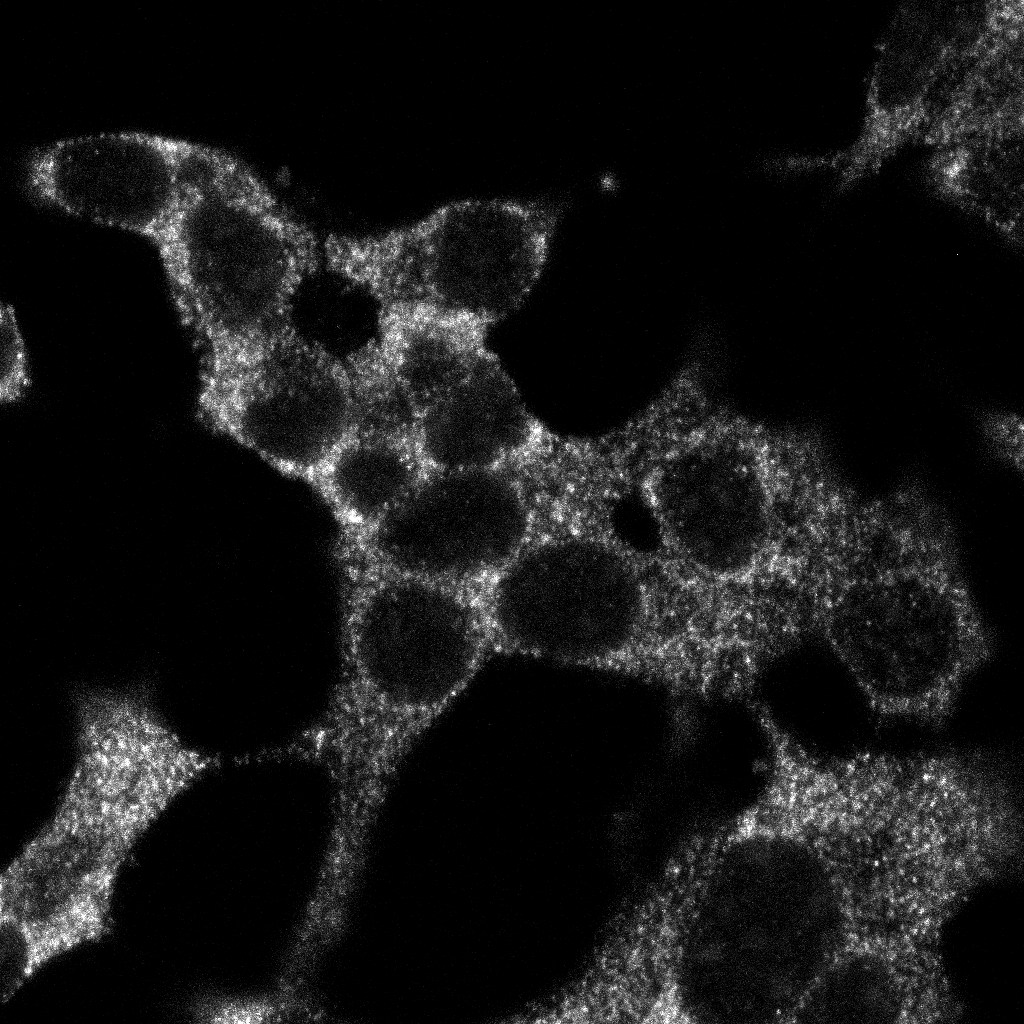

Supplement: Supplementary file 9 — Source data Fig. 4 [file 44318_2025_448_MOESM9_ESM.zip › Figure 4/Fig 4C/G3BP1 KO 2.8 (G3BP1 signal)/Composite-1.tif]

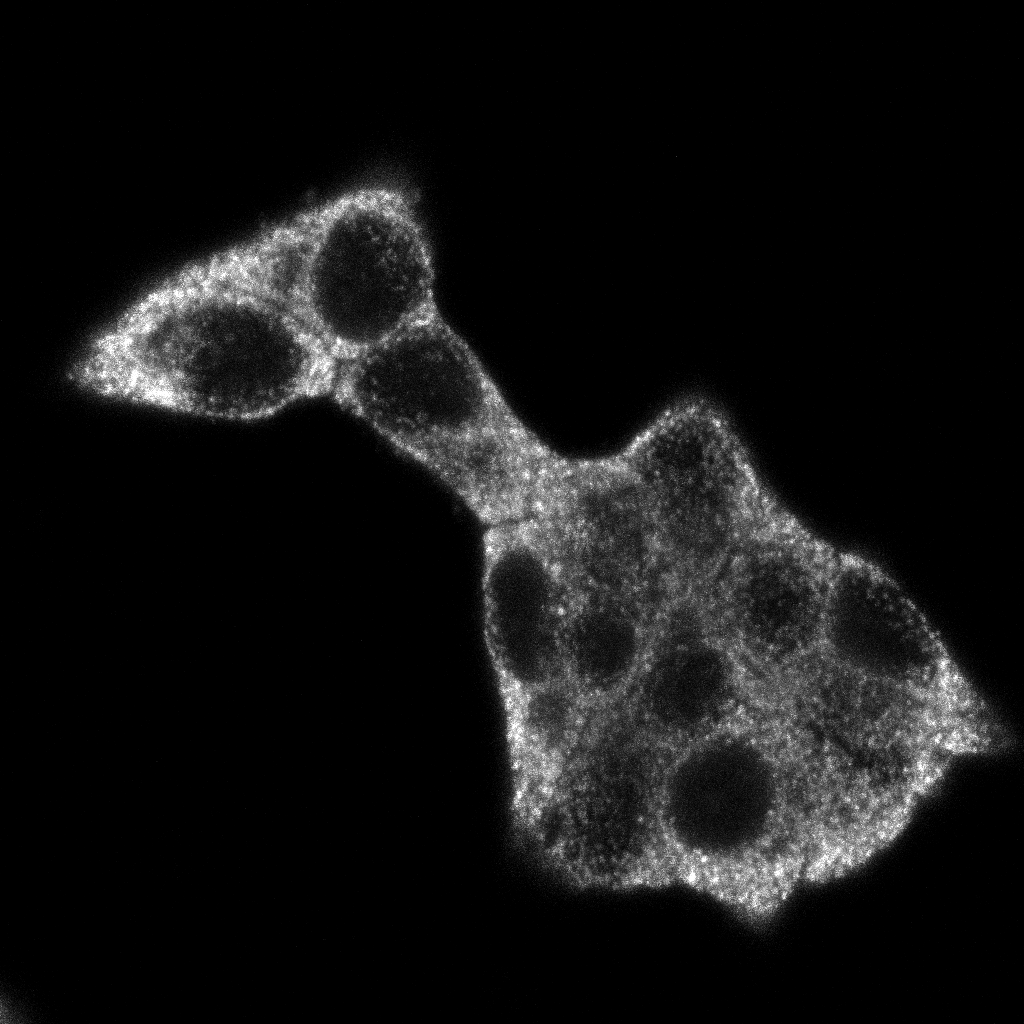

Supplement: Supplementary file 9 — Source data Fig. 4 [file 44318_2025_448_MOESM9_ESM.zip › Figure 4/Fig 4C/G3BP2 KO 2.8 (G3BP1 signal)/Composite.tif]

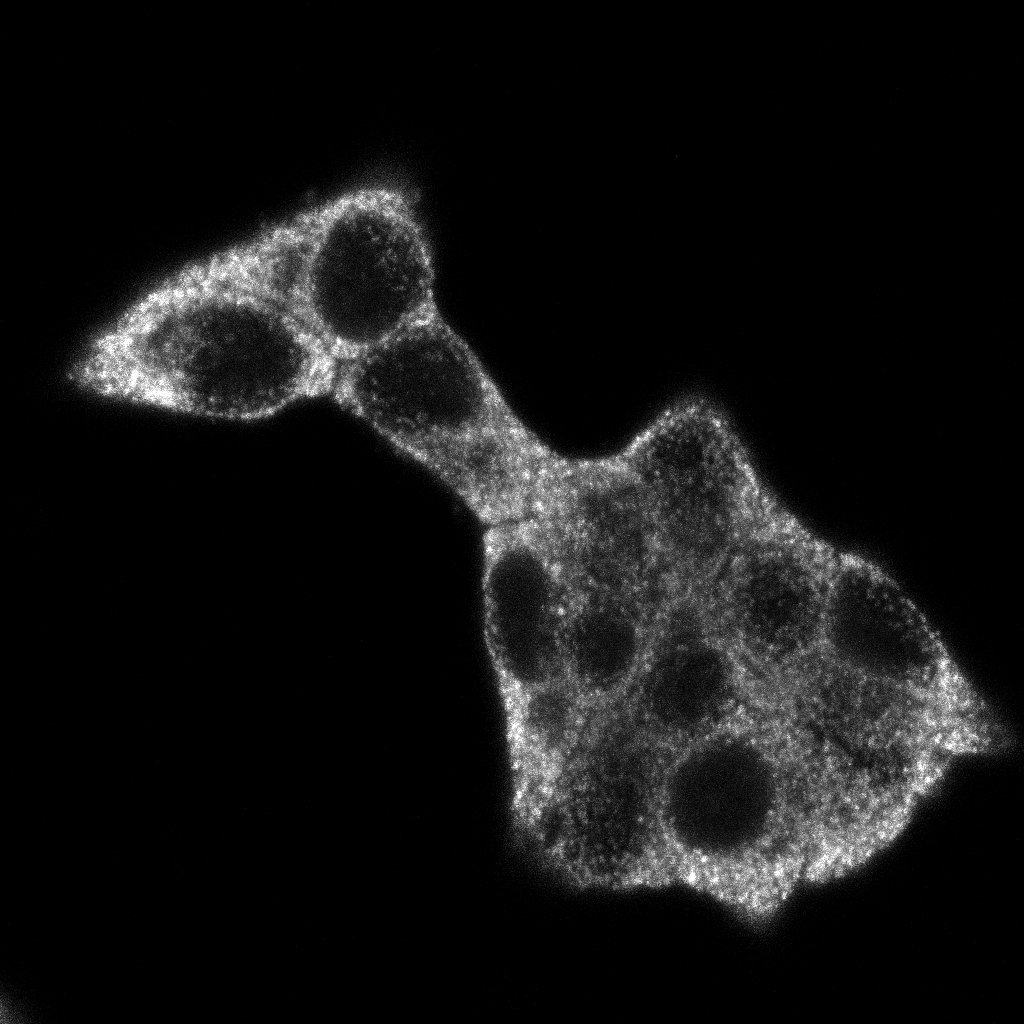

Supplement: Supplementary file 9 — Source data Fig. 4 [file 44318_2025_448_MOESM9_ESM.zip › Figure 4/Fig 4C/G3BP2 KO 2.8 (G3BP1 signal)/Composite-1.tif]

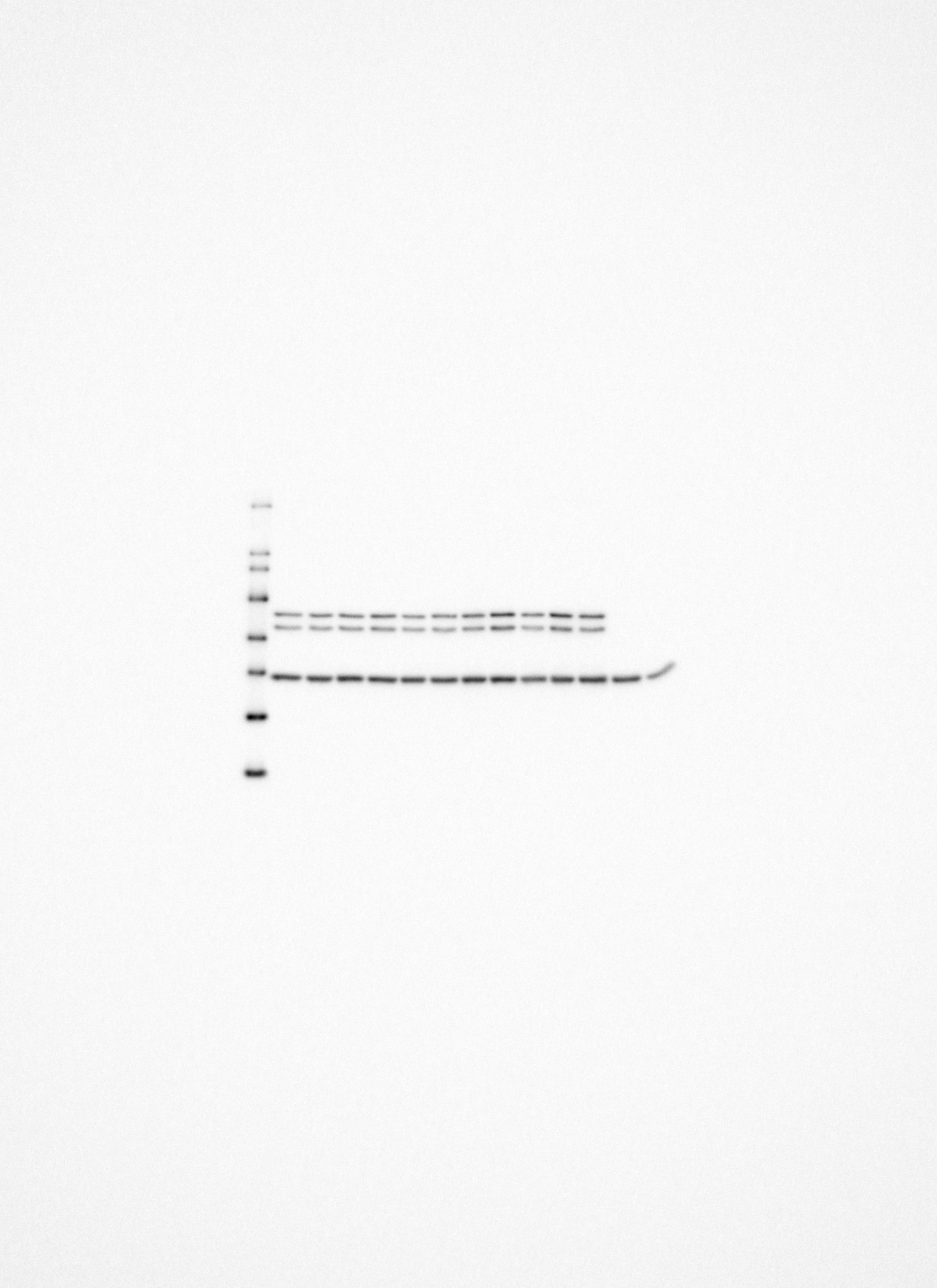

Supplement: Supplementary file 9 — Source data Fig. 4 [file 44318_2025_448_MOESM9_ESM.zip › Figure 4/Fig 4B/G3BP2 KO G3BP2 SIGNAL/19.05.21 PA50-500 R-S n1 G3BP2 1 2021.05.19_09.07.15_Ch.tif]

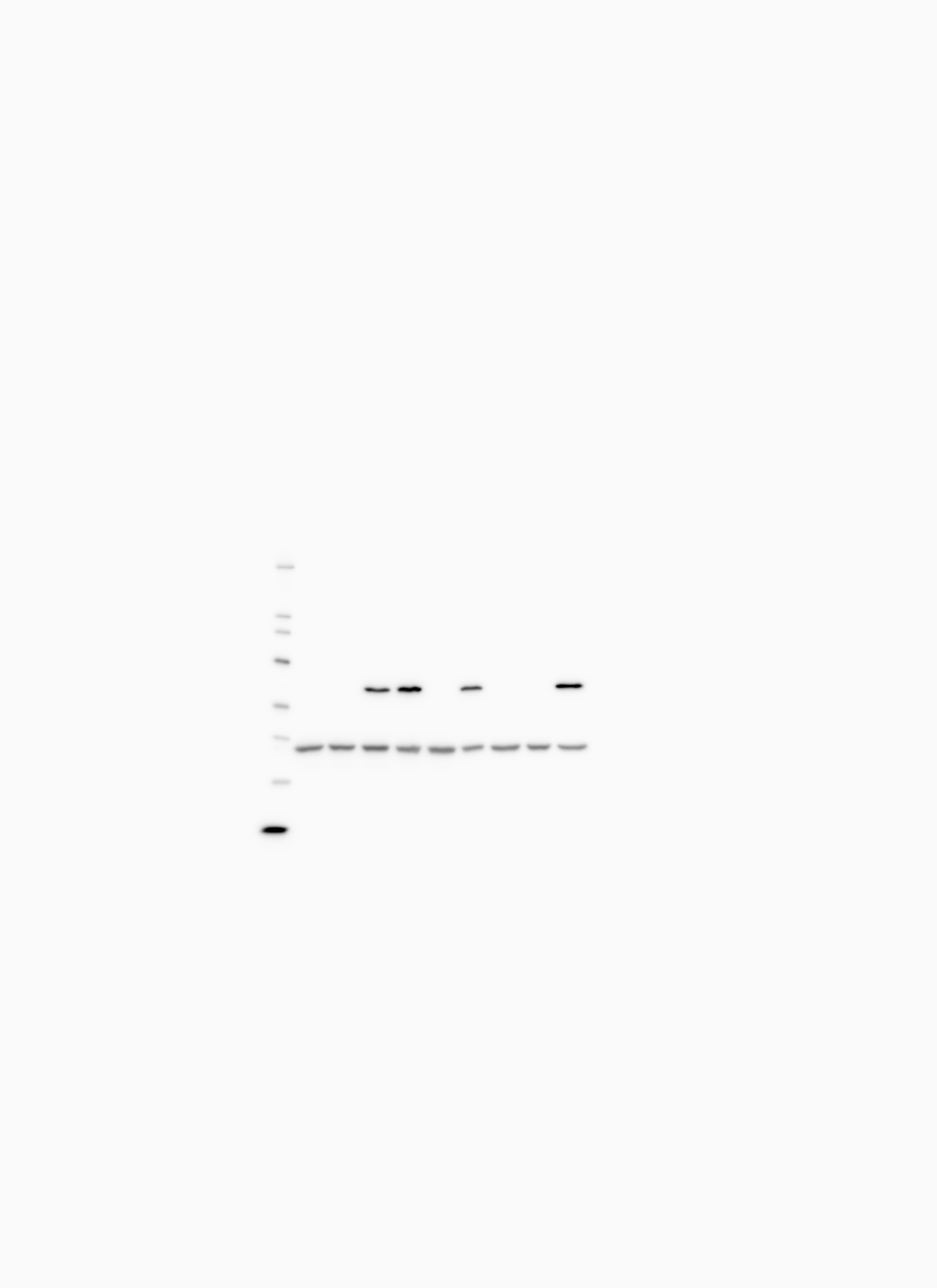

Supplement: Supplementary file 9 — Source data Fig. 4 [file 44318_2025_448_MOESM9_ESM.zip › Figure 4/Fig 4B/G3BP1 KO G3BP1 SIGNAL/sgRNA 3 G3BP1 KO 09.08.20 MIN6K8 2020.09.09_15.01.08_Ch.tif]

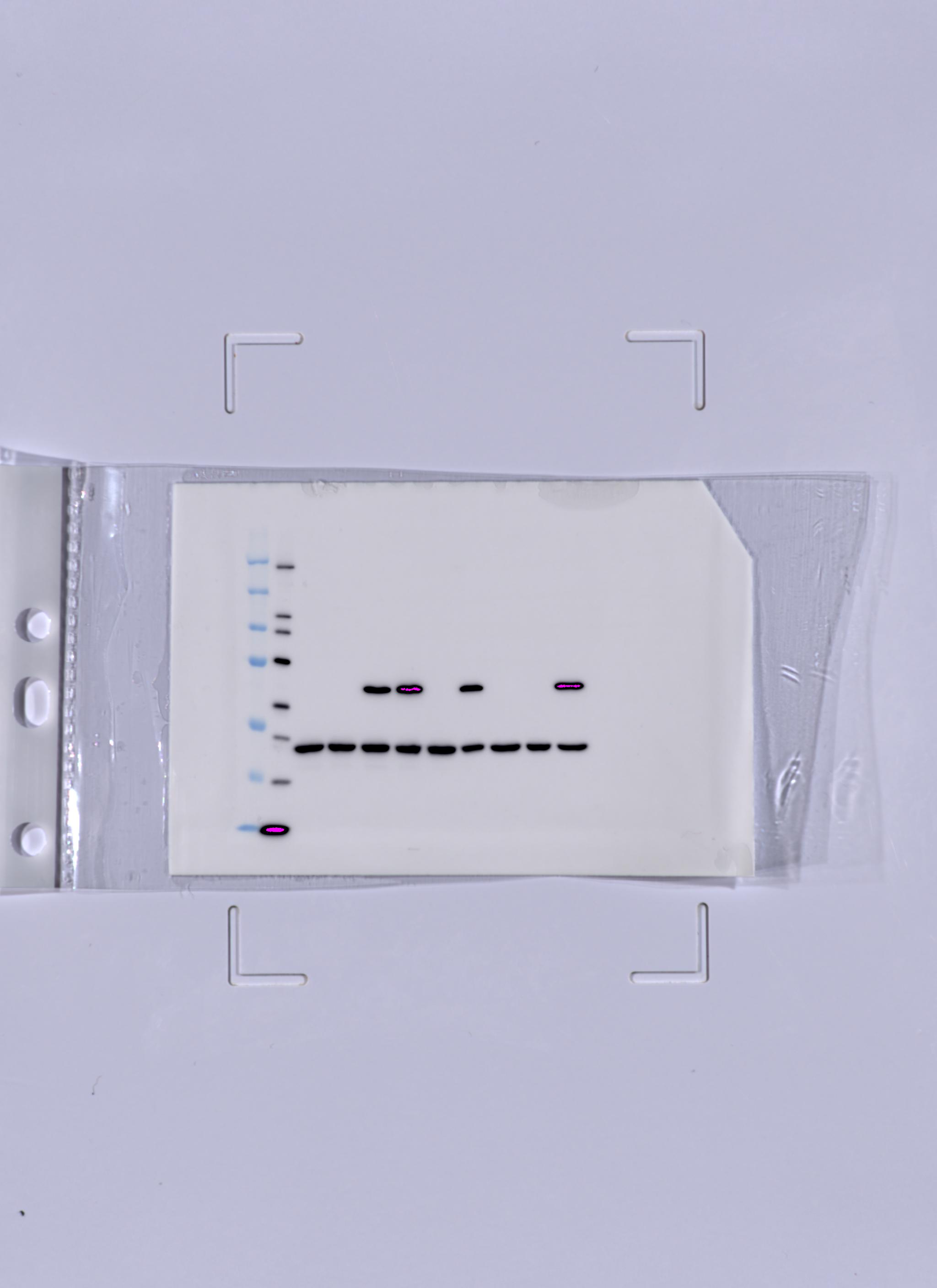

Supplement: Supplementary file 9 — Source data Fig. 4 [file 44318_2025_448_MOESM9_ESM.zip › Figure 4/Fig 4B/G3BP1 KO G3BP1 SIGNAL/sgRNA 3 G3BP1 KO 09.08.20 MIN6K8 2020.09.09_15.01.08_Ch+Marker.jpg]

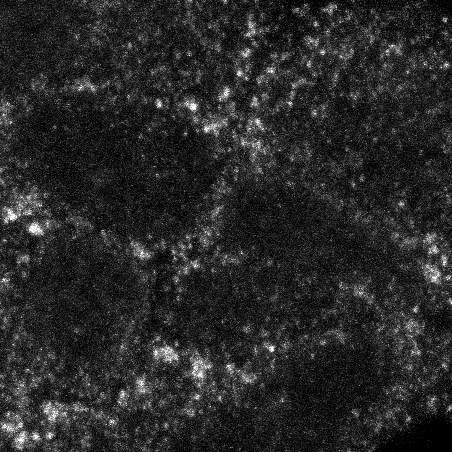

Supplement: Supplementary file 9 — Source data Fig. 4 [file 44318_2025_448_MOESM9_ESM.zip › Figure 4/Fig 4C/WT 2.8 (G3BP1 signal)/crop/Composite2.tif]

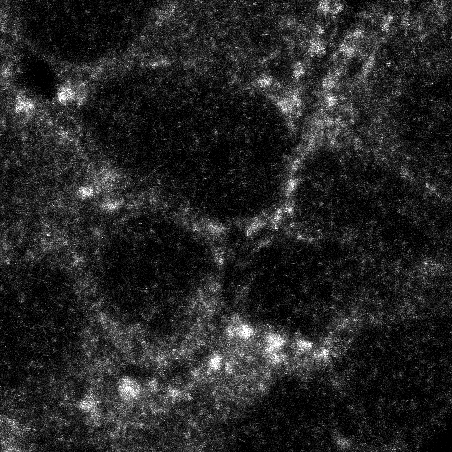

Supplement: Supplementary file 9 — Source data Fig. 4 [file 44318_2025_448_MOESM9_ESM.zip › Figure 4/Fig 4C/WT 2.8 (G3BP1 signal)/crop/INS1 MRNA.tif]

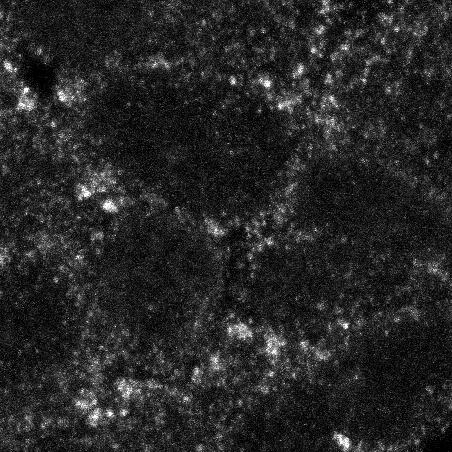

Supplement: Supplementary file 9 — Source data Fig. 4 [file 44318_2025_448_MOESM9_ESM.zip › Figure 4/Fig 4C/WT 2.8 (G3BP1 signal)/crop/Composite.tif]

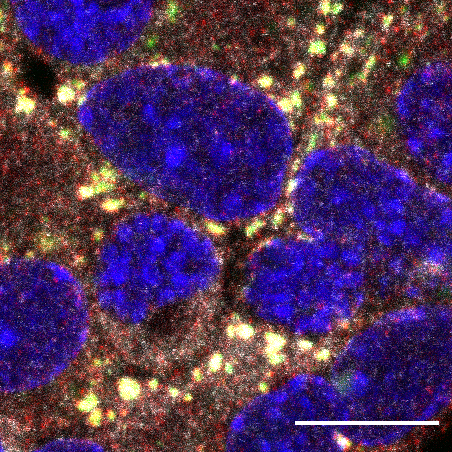

Supplement: Supplementary file 9 — Source data Fig. 4 [file 44318_2025_448_MOESM9_ESM.zip › Figure 4/Fig 4C/WT 2.8 (G3BP1 signal)/crop/Composite.tif (RGB).tif]

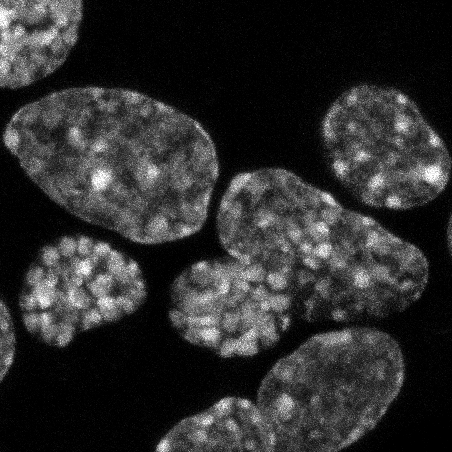

Supplement: Supplementary file 9 — Source data Fig. 4 [file 44318_2025_448_MOESM9_ESM.zip › Figure 4/Fig 4C/WT 2.8 (G3BP1 signal)/crop/DAPI.tif]

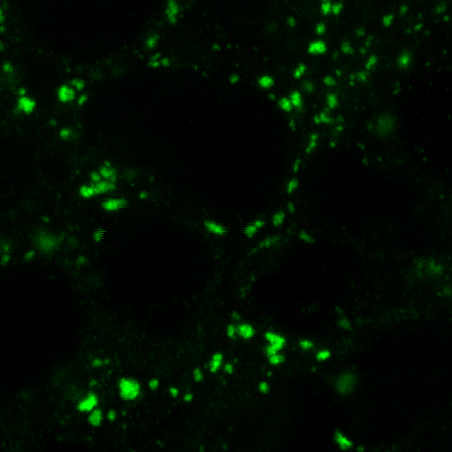

Supplement: Supplementary file 9 — Source data Fig. 4 [file 44318_2025_448_MOESM9_ESM.zip › Figure 4/Fig 4C/WT 2.8 (G3BP1 signal)/crop/G3BP1.tif]

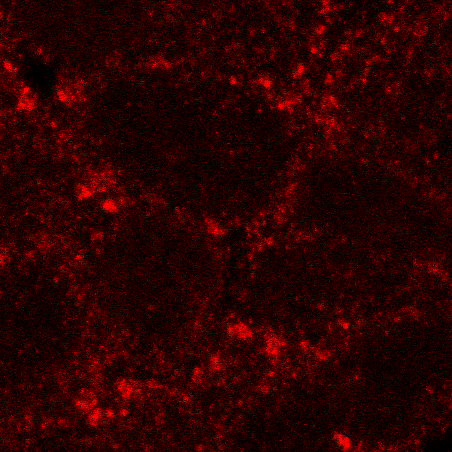

Supplement: Supplementary file 9 — Source data Fig. 4 [file 44318_2025_448_MOESM9_ESM.zip › Figure 4/Fig 4C/WT 2.8 (G3BP1 signal)/crop/EIF3B.tif]

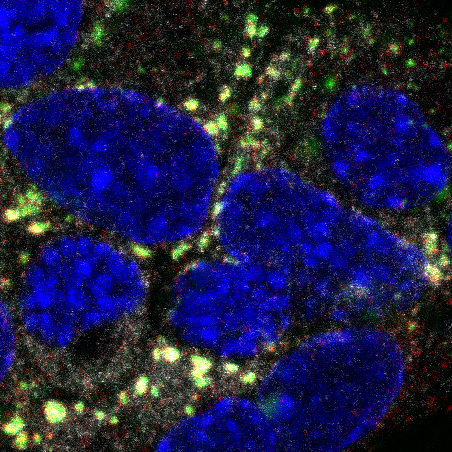

Supplement: Supplementary file 9 — Source data Fig. 4 [file 44318_2025_448_MOESM9_ESM.zip › Figure 4/Fig 4C/WT 2.8 (G3BP1 signal)/crop/Composite2.tif (RGB).tif]

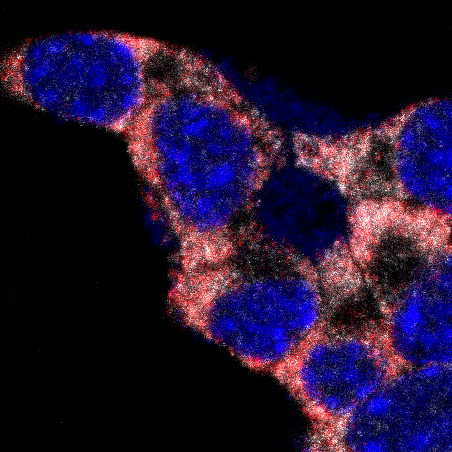

Supplement: Supplementary file 9 — Source data Fig. 4 [file 44318_2025_448_MOESM9_ESM.zip › Figure 4/Fig 4C/G3BP1 KO 2.8 (G3BP1 signal)/crop/Composite-1.tif2 (RGB).tif]

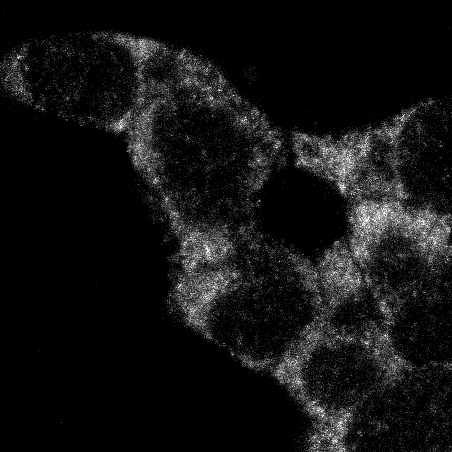

Supplement: Supplementary file 9 — Source data Fig. 4 [file 44318_2025_448_MOESM9_ESM.zip › Figure 4/Fig 4C/G3BP1 KO 2.8 (G3BP1 signal)/crop/INS1 MRNA.tif]

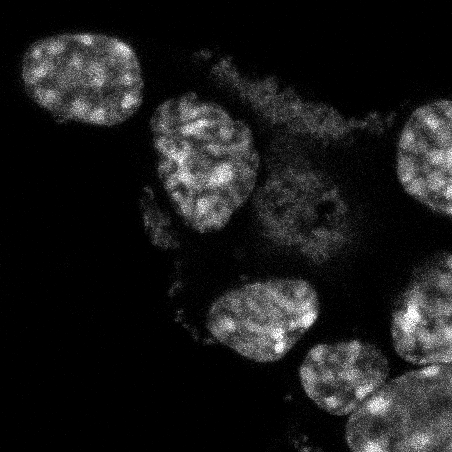

Supplement: Supplementary file 9 — Source data Fig. 4 [file 44318_2025_448_MOESM9_ESM.zip › Figure 4/Fig 4C/G3BP1 KO 2.8 (G3BP1 signal)/crop/DAPI.tif]

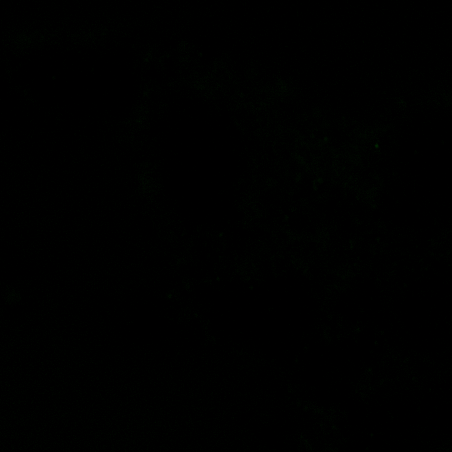

Supplement: Supplementary file 9 — Source data Fig. 4 [file 44318_2025_448_MOESM9_ESM.zip › Figure 4/Fig 4C/G3BP1 KO 2.8 (G3BP1 signal)/crop/G3BP1.tif]

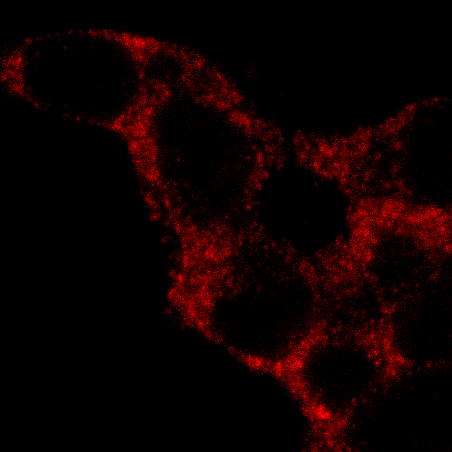

Supplement: Supplementary file 9 — Source data Fig. 4 [file 44318_2025_448_MOESM9_ESM.zip › Figure 4/Fig 4C/G3BP1 KO 2.8 (G3BP1 signal)/crop/EIF3B.tif]

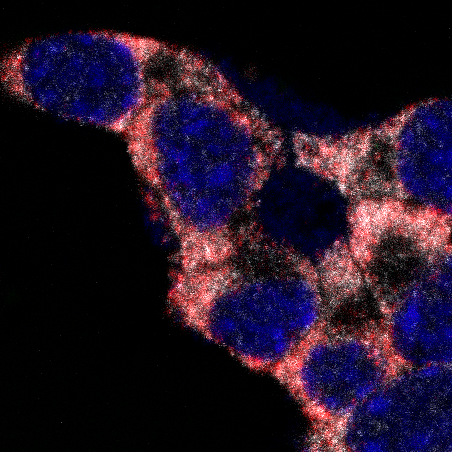

Supplement: Supplementary file 9 — Source data Fig. 4 [file 44318_2025_448_MOESM9_ESM.zip › Figure 4/Fig 4C/G3BP1 KO 2.8 (G3BP1 signal)/crop/Composite-1.tif (RGB).tif]

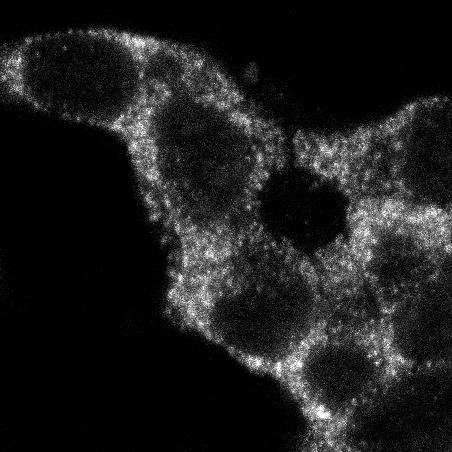

Supplement: Supplementary file 9 — Source data Fig. 4 [file 44318_2025_448_MOESM9_ESM.zip › Figure 4/Fig 4C/G3BP1 KO 2.8 (G3BP1 signal)/crop/Composite-1.tif]

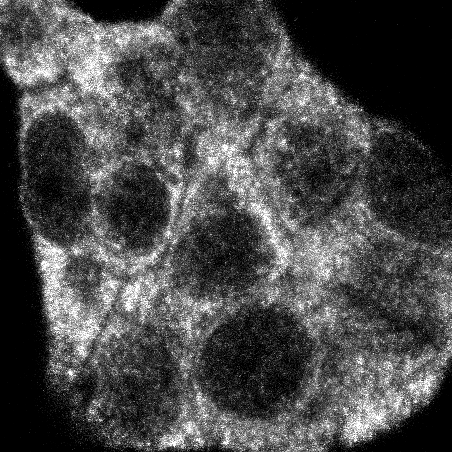

Supplement: Supplementary file 9 — Source data Fig. 4 [file 44318_2025_448_MOESM9_ESM.zip › Figure 4/Fig 4C/G3BP2 KO 2.8 (G3BP1 signal)/crop/INS1 MRNA.tif]

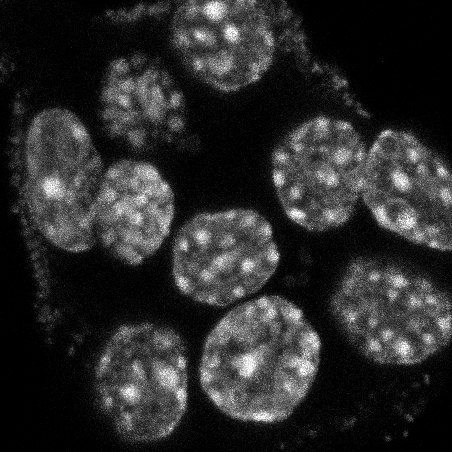

Supplement: Supplementary file 9 — Source data Fig. 4 [file 44318_2025_448_MOESM9_ESM.zip › Figure 4/Fig 4C/G3BP2 KO 2.8 (G3BP1 signal)/crop/DAPI.tif]

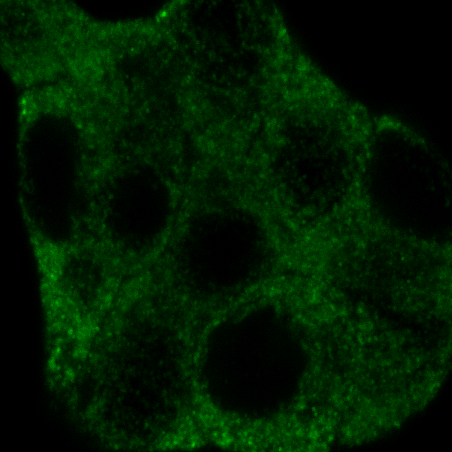

Supplement: Supplementary file 9 — Source data Fig. 4 [file 44318_2025_448_MOESM9_ESM.zip › Figure 4/Fig 4C/G3BP2 KO 2.8 (G3BP1 signal)/crop/G3BP1.tif]

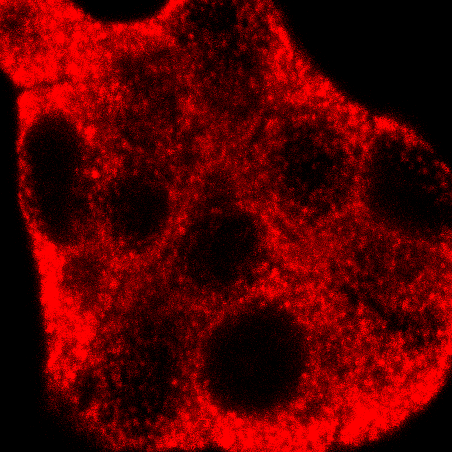

Supplement: Supplementary file 9 — Source data Fig. 4 [file 44318_2025_448_MOESM9_ESM.zip › Figure 4/Fig 4C/G3BP2 KO 2.8 (G3BP1 signal)/crop/EIF3B.tif]

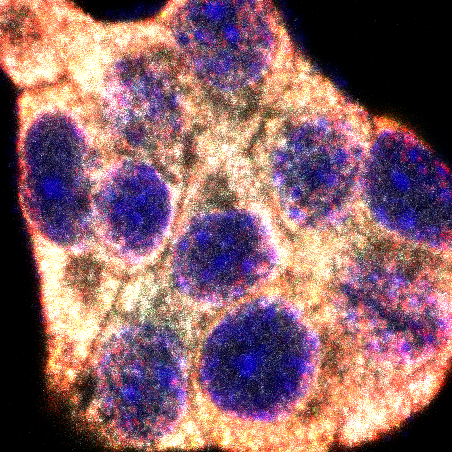

Supplement: Supplementary file 9 — Source data Fig. 4 [file 44318_2025_448_MOESM9_ESM.zip › Figure 4/Fig 4C/G3BP2 KO 2.8 (G3BP1 signal)/crop/Composite-1.tif (RGB).tif]

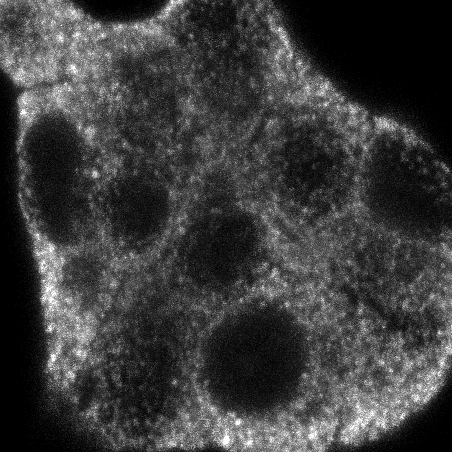

Supplement: Supplementary file 9 — Source data Fig. 4 [file 44318_2025_448_MOESM9_ESM.zip › Figure 4/Fig 4C/G3BP2 KO 2.8 (G3BP1 signal)/crop/Composite-1.tif]

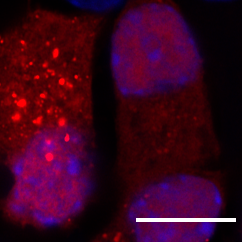

Supplement: Supplementary file 9 — Source data Fig. 4 [file 44318_2025_448_MOESM9_ESM.zip › Figure 4/Fig 4D/stim/cl8 stim mcherry-hG3BP1/KO-cl.8-mCherry-hG3BP1-Stim-04.tif]

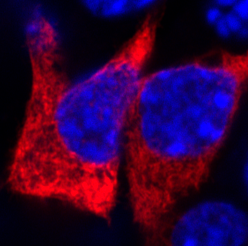

Supplement: Supplementary file 9 — Source data Fig. 4 [file 44318_2025_448_MOESM9_ESM.zip › Figure 4/Fig 4D/stim/cl8 stim mcherry/KO-cl.8-wt-pmCherry-Stim-02.tif]

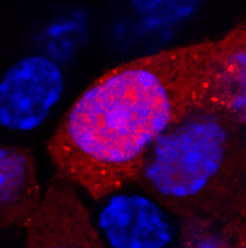

Supplement: Supplementary file 9 — Source data Fig. 4 [file 44318_2025_448_MOESM9_ESM.zip › Figure 4/Fig 4D/stim/wt stim mcherry-hg3bp1/K8-wt-pmCherry-hG3BP1-Stim-01.tif]

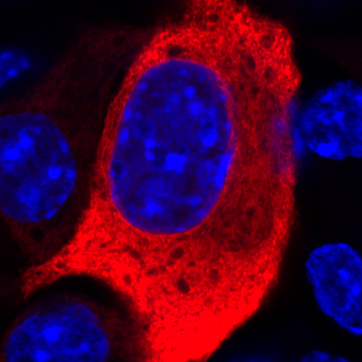

Supplement: Supplementary file 9 — Source data Fig. 4 [file 44318_2025_448_MOESM9_ESM.zip › Figure 4/Fig 4D/stim/wt stim mcherry/K8-wt-pmCherry-Stim-03.tif]

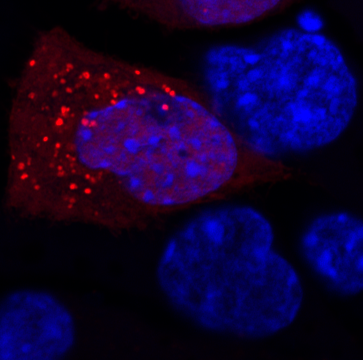

Supplement: Supplementary file 9 — Source data Fig. 4 [file 44318_2025_448_MOESM9_ESM.zip › Figure 4/Fig 4D/rest/WT res mcherry-G3BP1/K8-wt-mCherry-hG3BP1-Rest-01.tif]

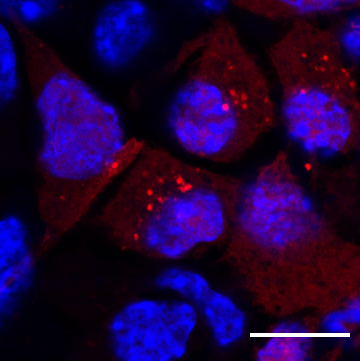

Supplement: Supplementary file 9 — Source data Fig. 4 [file 44318_2025_448_MOESM9_ESM.zip › Figure 4/Fig 4D/rest/G3BP1 KO CL8 rest mcherry-hG3BP1/KO-cl.8-mCherry-hG3BP1-Rest-02.tif]

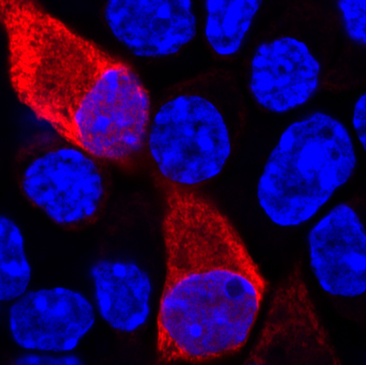

Supplement: Supplementary file 9 — Source data Fig. 4 [file 44318_2025_448_MOESM9_ESM.zip › Figure 4/Fig 4D/rest/G3BP1 KO CL8 rest mcherry/K8-wt-pmCherry-Rest-01.tif]

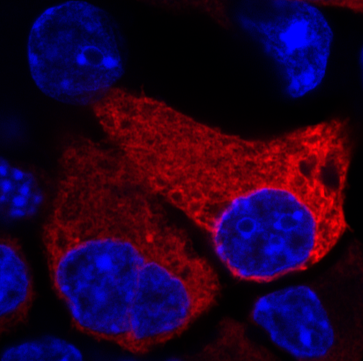

Supplement: Supplementary file 9 — Source data Fig. 4 [file 44318_2025_448_MOESM9_ESM.zip › Figure 4/Fig 4D/rest/WT rest mcherry/K8-wt-pmCherry-Rest-01.tif]

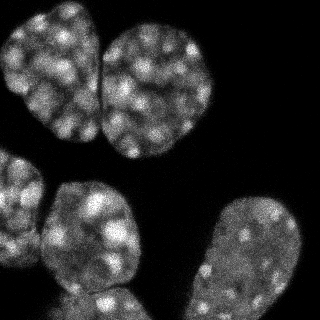

Supplement: Supplementary file 10 — Source data Fig. 5 [file 44318_2025_448_MOESM10_ESM.zip › Figure 5/Fig 5A/2.8 + PA/composite.tif]

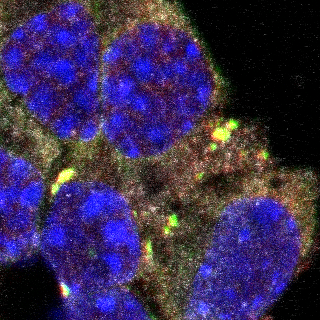

Supplement: Supplementary file 10 — Source data Fig. 5 [file 44318_2025_448_MOESM10_ESM.zip › Figure 5/Fig 5A/2.8 + PA/composite(RGB).tif]

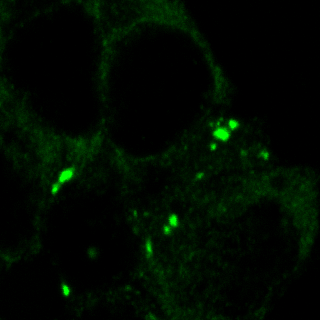

Supplement: Supplementary file 10 — Source data Fig. 5 [file 44318_2025_448_MOESM10_ESM.zip › Figure 5/Fig 5A/2.8 + PA/g3bp1.tif]

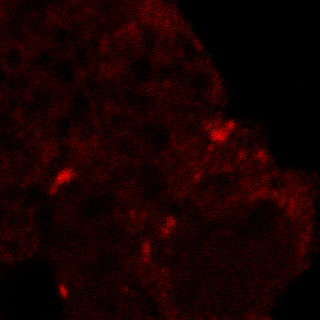

Supplement: Supplementary file 10 — Source data Fig. 5 [file 44318_2025_448_MOESM10_ESM.zip › Figure 5/Fig 5A/2.8 + PA/eif3b.tif]
