# Supplementary figures and images for: Aldolase-regulated G3BP1/2+ condensates control insulin mRNA storage in beta cells (part 3 of 4)
Source: EMBO J. 2025 May 12;44(13):3669–96. doi: 10.1038/s44318-025-00448-7 (PMC12216156; doi:10.1038/s44318-025-00448-7)

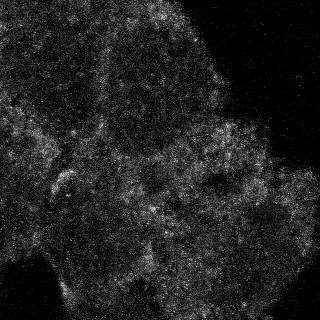

Supplement: Supplementary file 10 — Source data Fig. 5 [file 44318_2025_448_MOESM10_ESM.zip › Figure 5/Fig 5A/2.8 + PA/ins mrna.tif]

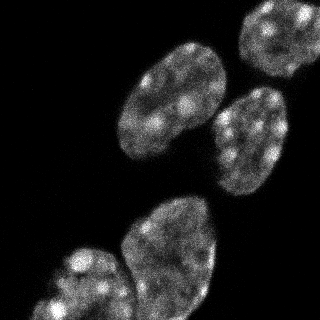

Supplement: Supplementary file 10 — Source data Fig. 5 [file 44318_2025_448_MOESM10_ESM.zip › Figure 5/Fig 5A/2.8 + DMSO/composite.tif]

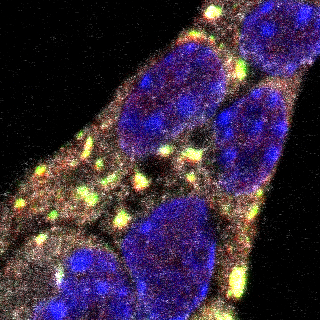

Supplement: Supplementary file 10 — Source data Fig. 5 [file 44318_2025_448_MOESM10_ESM.zip › Figure 5/Fig 5A/2.8 + DMSO/composite.tif (RGB).tif]

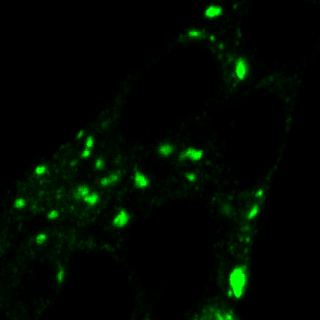

Supplement: Supplementary file 10 — Source data Fig. 5 [file 44318_2025_448_MOESM10_ESM.zip › Figure 5/Fig 5A/2.8 + DMSO/g3bp1.tif]

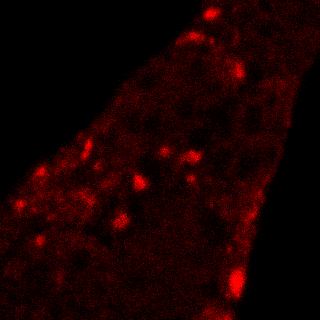

Supplement: Supplementary file 10 — Source data Fig. 5 [file 44318_2025_448_MOESM10_ESM.zip › Figure 5/Fig 5A/2.8 + DMSO/eif3b.tif]

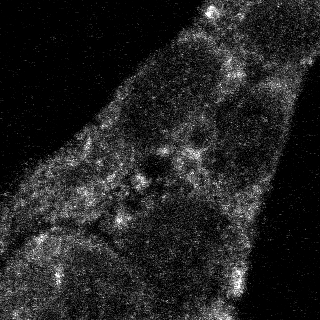

Supplement: Supplementary file 10 — Source data Fig. 5 [file 44318_2025_448_MOESM10_ESM.zip › Figure 5/Fig 5A/2.8 + DMSO/ins mrna.tif]

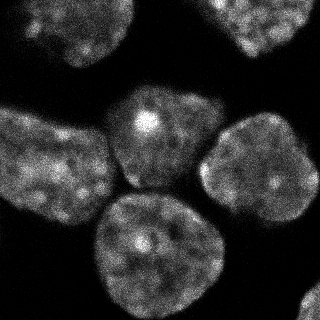

Supplement: Supplementary file 10 — Source data Fig. 5 [file 44318_2025_448_MOESM10_ESM.zip › Figure 5/Fig 5A/2.8 + exendin/composite.tif]

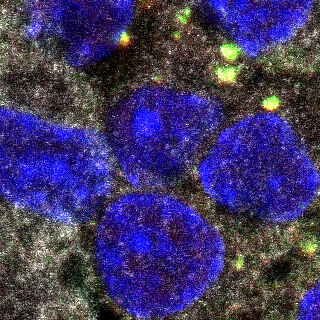

Supplement: Supplementary file 10 — Source data Fig. 5 [file 44318_2025_448_MOESM10_ESM.zip › Figure 5/Fig 5A/2.8 + exendin/composite(RGB).tif]

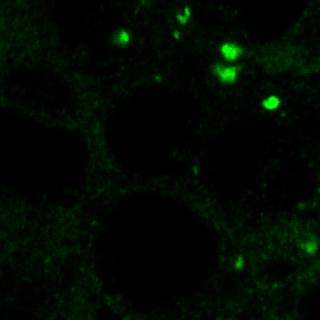

Supplement: Supplementary file 10 — Source data Fig. 5 [file 44318_2025_448_MOESM10_ESM.zip › Figure 5/Fig 5A/2.8 + exendin/g3bp1.tif]

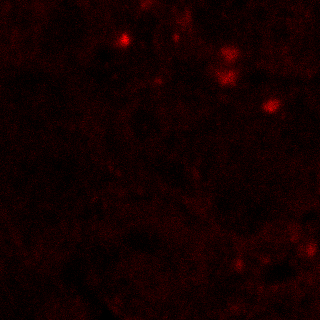

Supplement: Supplementary file 10 — Source data Fig. 5 [file 44318_2025_448_MOESM10_ESM.zip › Figure 5/Fig 5A/2.8 + exendin/eif3b.tif]

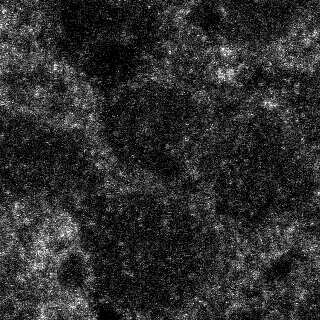

Supplement: Supplementary file 10 — Source data Fig. 5 [file 44318_2025_448_MOESM10_ESM.zip › Figure 5/Fig 5A/2.8 + exendin/ins mrna.tif]

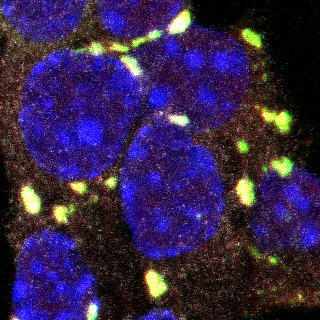

Supplement: Supplementary file 10 — Source data Fig. 5 [file 44318_2025_448_MOESM10_ESM.zip › Figure 5/Fig 5A/2.8/composite crop 22,08.tif (RGB).tif]

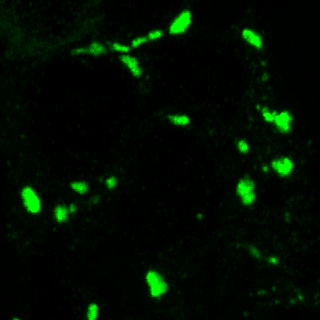

Supplement: Supplementary file 10 — Source data Fig. 5 [file 44318_2025_448_MOESM10_ESM.zip › Figure 5/Fig 5A/2.8/G3BP1.tif]

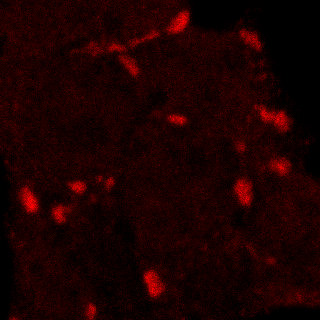

Supplement: Supplementary file 10 — Source data Fig. 5 [file 44318_2025_448_MOESM10_ESM.zip › Figure 5/Fig 5A/2.8/EIF3B.tif]

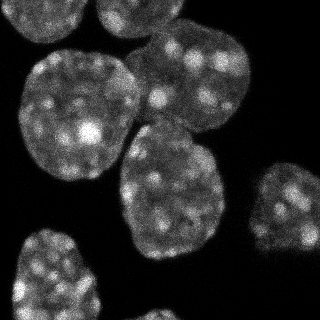

Supplement: Supplementary file 10 — Source data Fig. 5 [file 44318_2025_448_MOESM10_ESM.zip › Figure 5/Fig 5A/2.8/composite crop 22,08.tif]

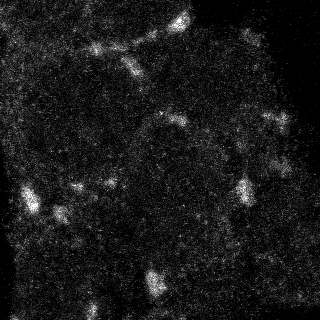

Supplement: Supplementary file 10 — Source data Fig. 5 [file 44318_2025_448_MOESM10_ESM.zip › Figure 5/Fig 5A/2.8/INS MRNA.tif]

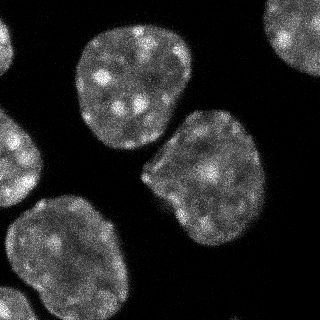

Supplement: Supplementary file 10 — Source data Fig. 5 [file 44318_2025_448_MOESM10_ESM.zip › Figure 5/Fig 5A/16.7/composite.tif]

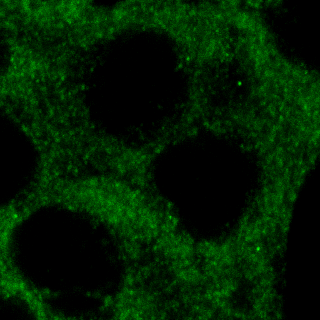

Supplement: Supplementary file 10 — Source data Fig. 5 [file 44318_2025_448_MOESM10_ESM.zip › Figure 5/Fig 5A/16.7/g3p1.tif]

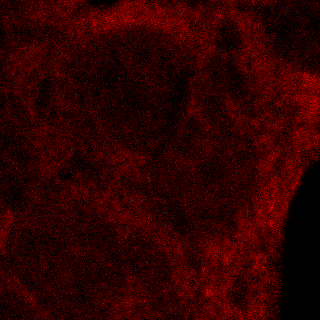

Supplement: Supplementary file 10 — Source data Fig. 5 [file 44318_2025_448_MOESM10_ESM.zip › Figure 5/Fig 5A/16.7/eif3b.tif]

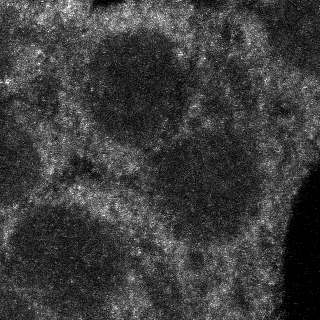

Supplement: Supplementary file 10 — Source data Fig. 5 [file 44318_2025_448_MOESM10_ESM.zip › Figure 5/Fig 5A/16.7/ins mrna.tif]

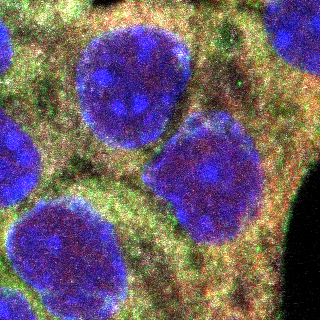

Supplement: Supplementary file 10 — Source data Fig. 5 [file 44318_2025_448_MOESM10_ESM.zip › Figure 5/Fig 5A/16.7/composite (RGB).tif]

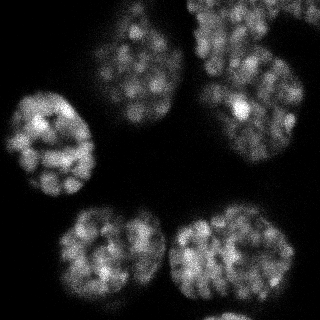

Supplement: Supplementary file 10 — Source data Fig. 5 [file 44318_2025_448_MOESM10_ESM.zip › Figure 5/Fig 5A/2.8 + KCl/composite.tif]

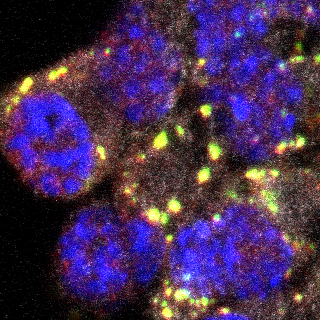

Supplement: Supplementary file 10 — Source data Fig. 5 [file 44318_2025_448_MOESM10_ESM.zip › Figure 5/Fig 5A/2.8 + KCl/composite(RGB).tif]

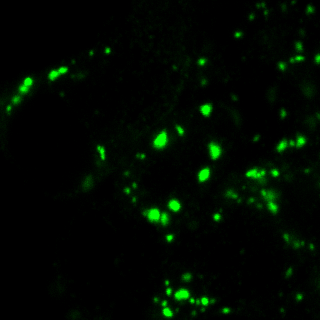

Supplement: Supplementary file 10 — Source data Fig. 5 [file 44318_2025_448_MOESM10_ESM.zip › Figure 5/Fig 5A/2.8 + KCl/g3bp1.tif]

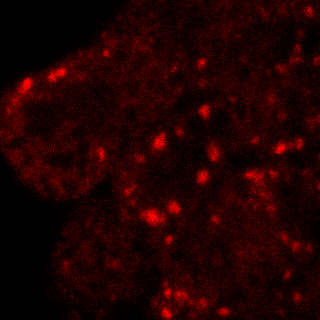

Supplement: Supplementary file 10 — Source data Fig. 5 [file 44318_2025_448_MOESM10_ESM.zip › Figure 5/Fig 5A/2.8 + KCl/eif3b.tif]

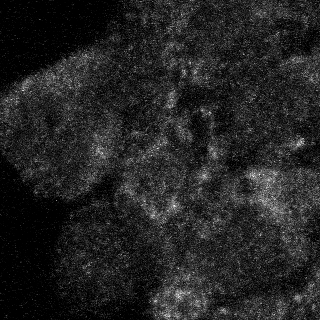

Supplement: Supplementary file 10 — Source data Fig. 5 [file 44318_2025_448_MOESM10_ESM.zip › Figure 5/Fig 5A/2.8 + KCl/ins mrna.tif]

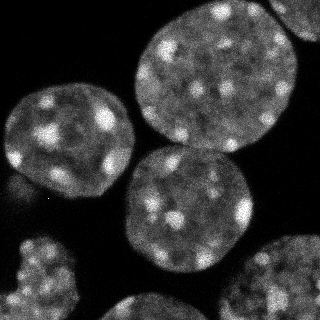

Supplement: Supplementary file 10 — Source data Fig. 5 [file 44318_2025_448_MOESM10_ESM.zip › Figure 5/Fig 5A/2.8 + Ro-28-1675/composite.tif]

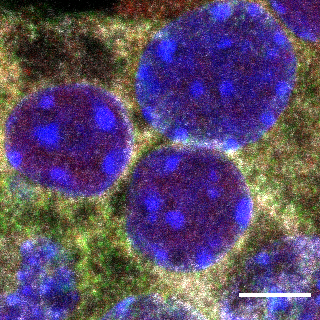

Supplement: Supplementary file 10 — Source data Fig. 5 [file 44318_2025_448_MOESM10_ESM.zip › Figure 5/Fig 5A/2.8 + Ro-28-1675/composite.tif (RGB).tif]

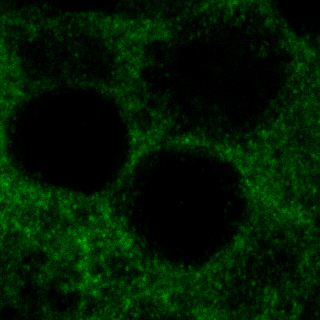

Supplement: Supplementary file 10 — Source data Fig. 5 [file 44318_2025_448_MOESM10_ESM.zip › Figure 5/Fig 5A/2.8 + Ro-28-1675/g3bp1.tif]

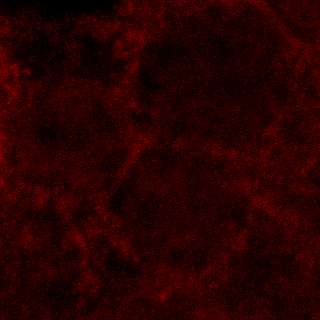

Supplement: Supplementary file 10 — Source data Fig. 5 [file 44318_2025_448_MOESM10_ESM.zip › Figure 5/Fig 5A/2.8 + Ro-28-1675/eif3b.tif]

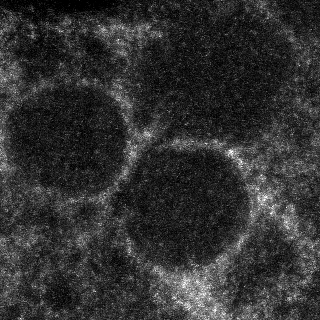

Supplement: Supplementary file 10 — Source data Fig. 5 [file 44318_2025_448_MOESM10_ESM.zip › Figure 5/Fig 5A/2.8 + Ro-28-1675/ins mrna.tif]

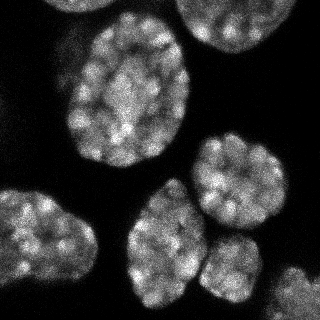

Supplement: Supplementary file 10 — Source data Fig. 5 [file 44318_2025_448_MOESM10_ESM.zip › Figure 5/Fig 5A/2.8 + EtOH/composite.tif]

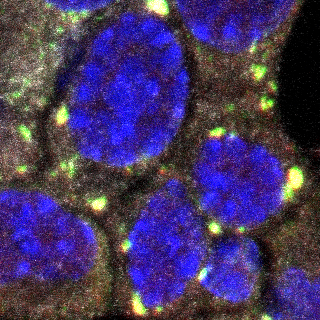

Supplement: Supplementary file 10 — Source data Fig. 5 [file 44318_2025_448_MOESM10_ESM.zip › Figure 5/Fig 5A/2.8 + EtOH/composite(RGB).tif]

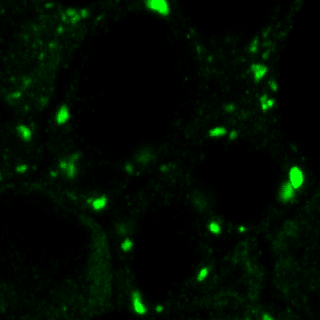

Supplement: Supplementary file 10 — Source data Fig. 5 [file 44318_2025_448_MOESM10_ESM.zip › Figure 5/Fig 5A/2.8 + EtOH/g3bp1.tif]

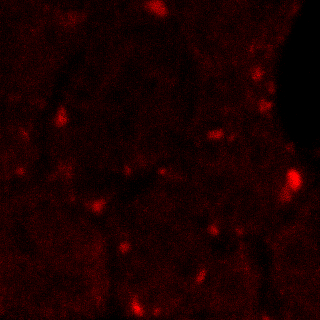

Supplement: Supplementary file 10 — Source data Fig. 5 [file 44318_2025_448_MOESM10_ESM.zip › Figure 5/Fig 5A/2.8 + EtOH/eif3b.tif]

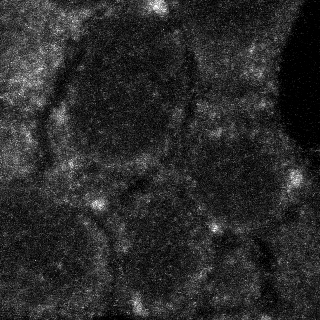

Supplement: Supplementary file 10 — Source data Fig. 5 [file 44318_2025_448_MOESM10_ESM.zip › Figure 5/Fig 5A/2.8 + EtOH/ins mrna.tif]

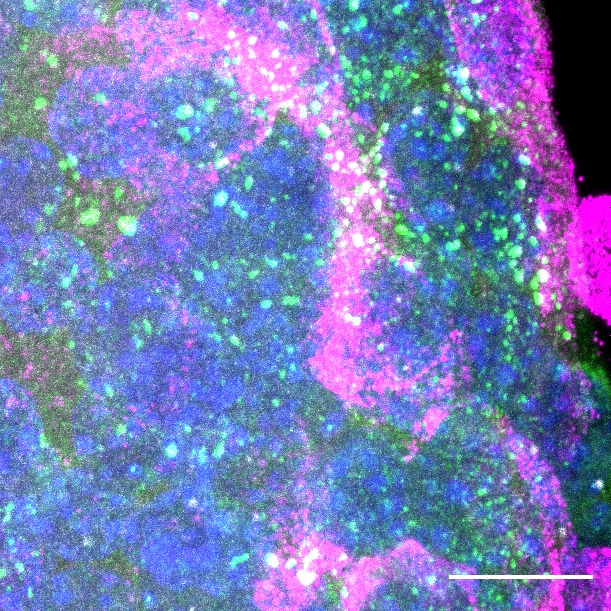

Supplement: Supplementary file 11 — Source data Fig. 6 [file 44318_2025_448_MOESM11_ESM.zip › Figure 6/Fig 6D/Composite CROP (RGB).tif]

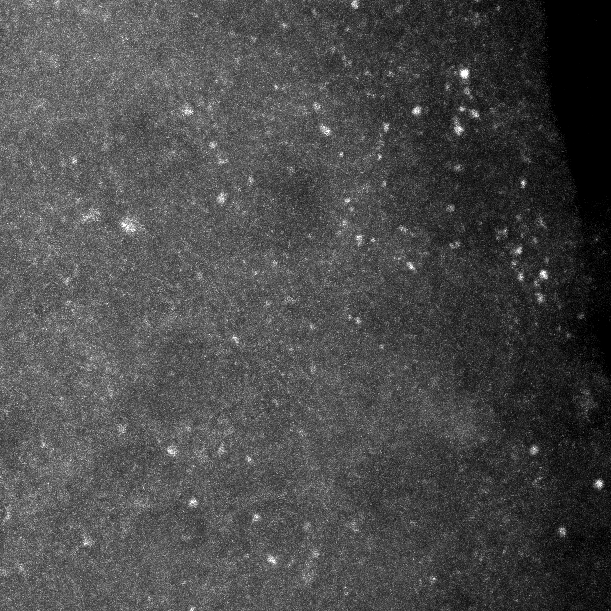

Supplement: Supplementary file 11 — Source data Fig. 6 [file 44318_2025_448_MOESM11_ESM.zip › Figure 6/Fig 6D/ins2mrna.tif]

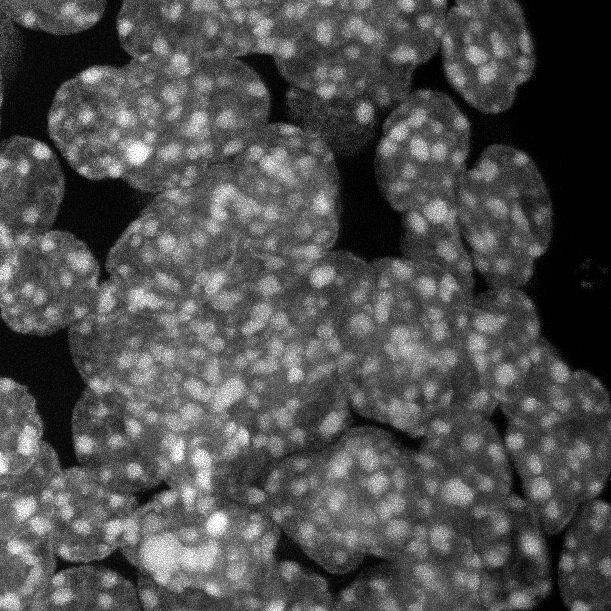

Supplement: Supplementary file 11 — Source data Fig. 6 [file 44318_2025_448_MOESM11_ESM.zip › Figure 6/Fig 6D/dapi.tif]

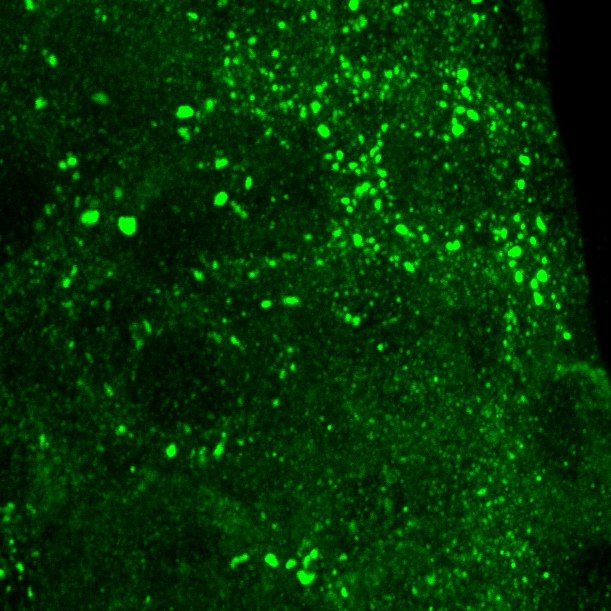

Supplement: Supplementary file 11 — Source data Fig. 6 [file 44318_2025_448_MOESM11_ESM.zip › Figure 6/Fig 6D/g3bp1.tif]

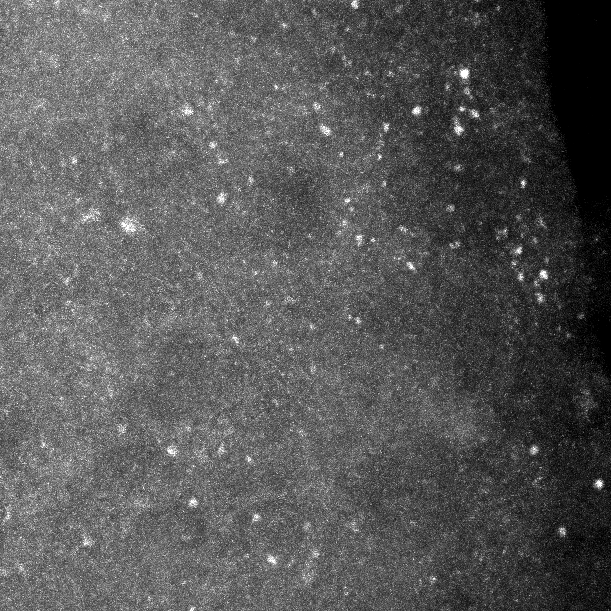

Supplement: Supplementary file 11 — Source data Fig. 6 [file 44318_2025_448_MOESM11_ESM.zip › Figure 6/Fig 6D/Composite CROP 42.15.tif]

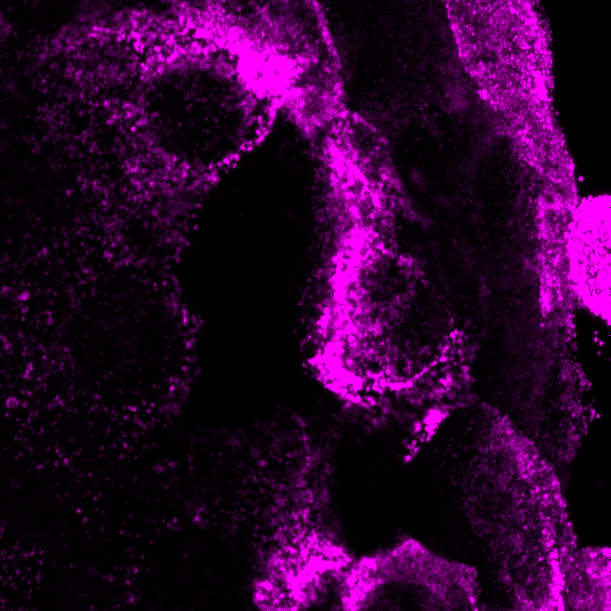

Supplement: Supplementary file 11 — Source data Fig. 6 [file 44318_2025_448_MOESM11_ESM.zip › Figure 6/Fig 6D/insulin.tif]

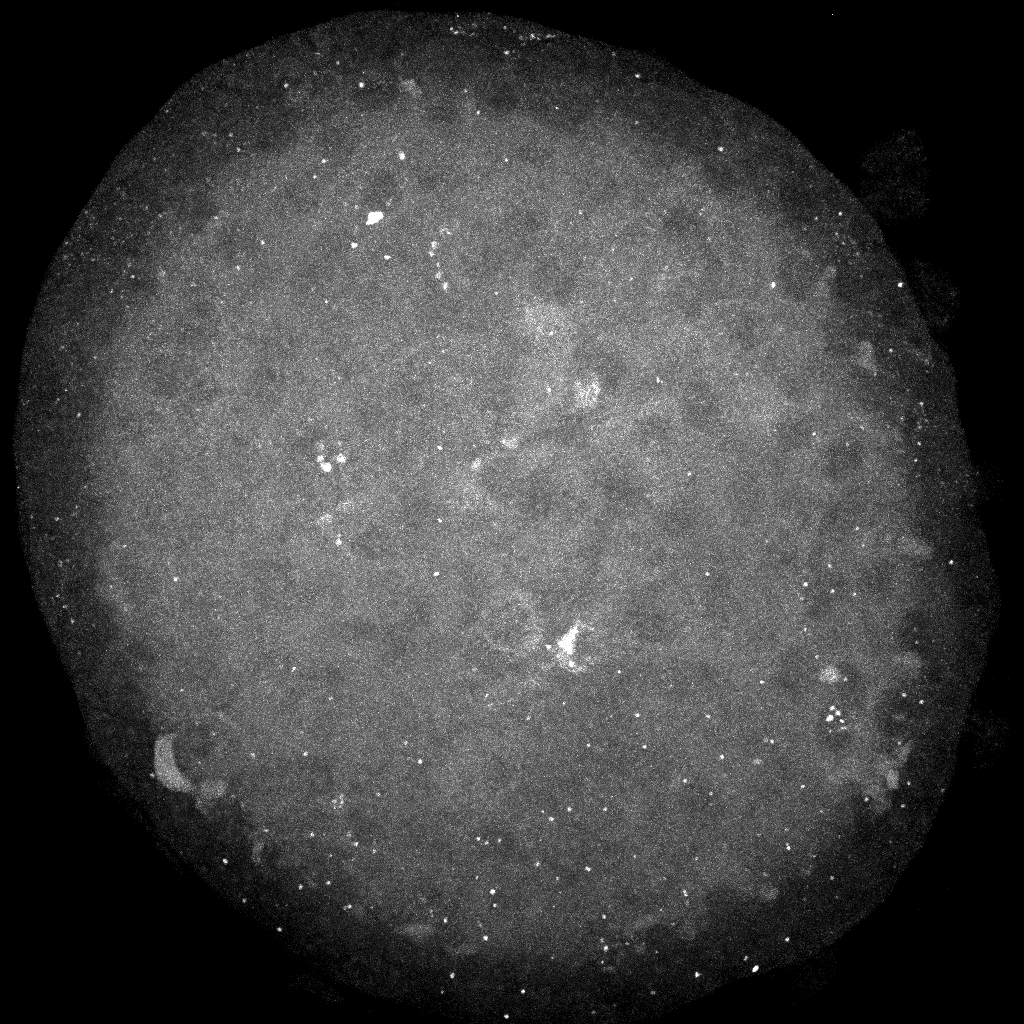

Supplement: Supplementary file 11 — Source data Fig. 6 [file 44318_2025_448_MOESM11_ESM.zip › Figure 6/Fig 6C/Composite.tif]

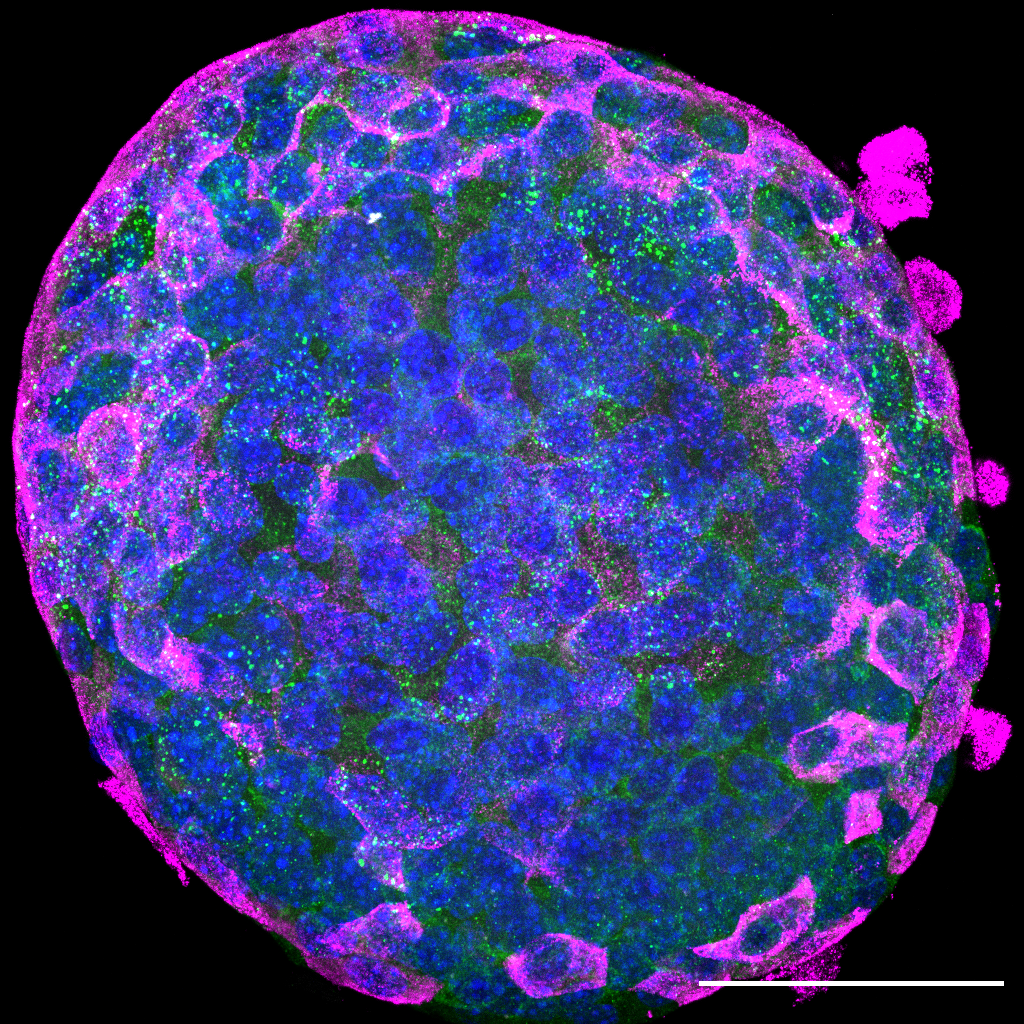

Supplement: Supplementary file 11 — Source data Fig. 6 [file 44318_2025_448_MOESM11_ESM.zip › Figure 6/Fig 6C/Composite.tif (RGB).tif]

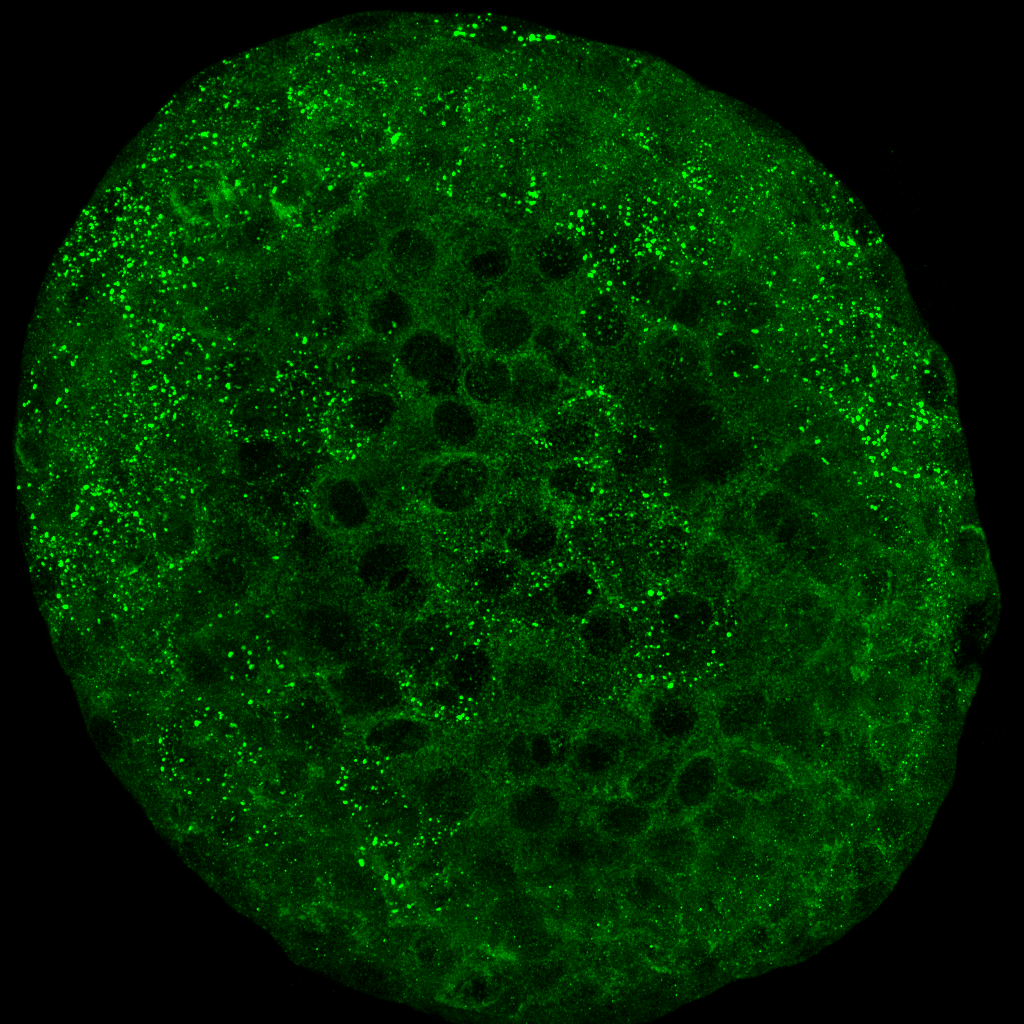

Supplement: Supplementary file 11 — Source data Fig. 6 [file 44318_2025_448_MOESM11_ESM.zip › Figure 6/Fig 6C/G3BP1.tif]

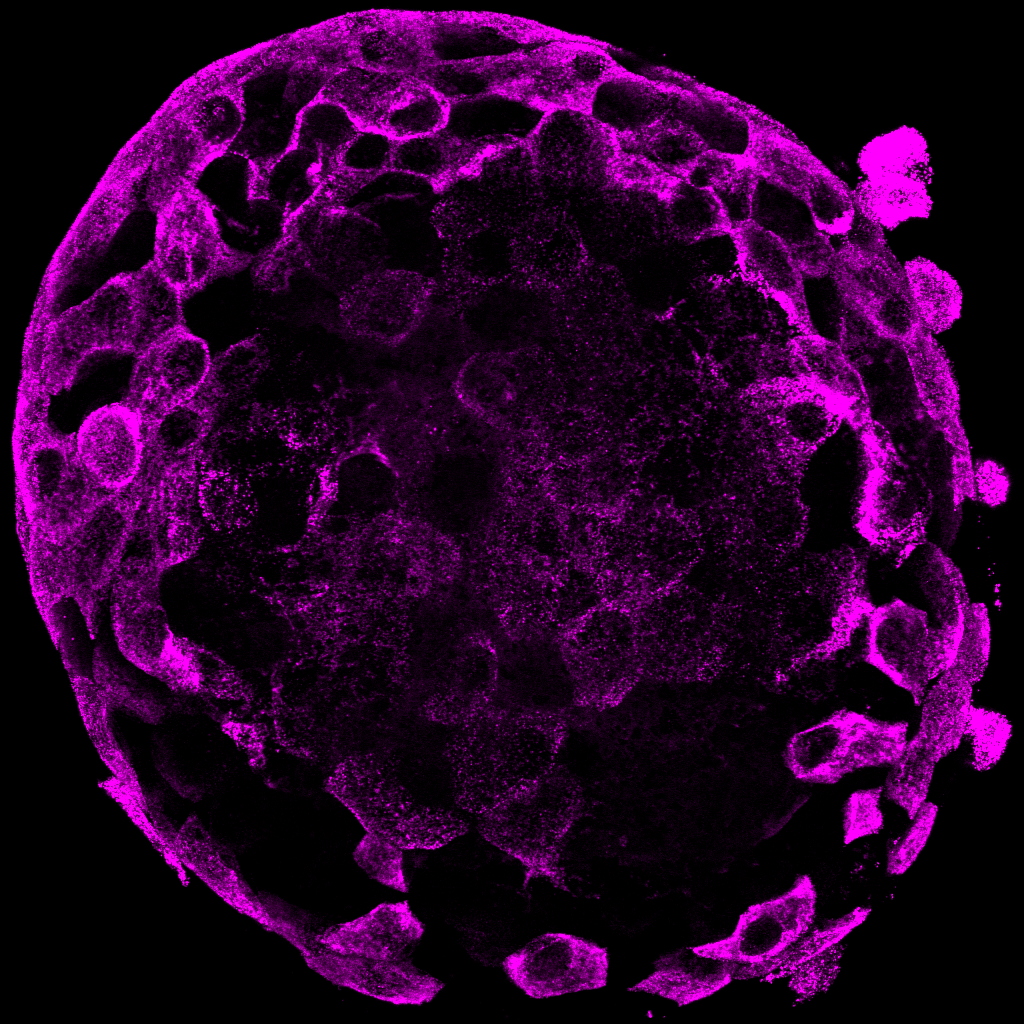

Supplement: Supplementary file 11 — Source data Fig. 6 [file 44318_2025_448_MOESM11_ESM.zip › Figure 6/Fig 6C/INSULIN.tif]

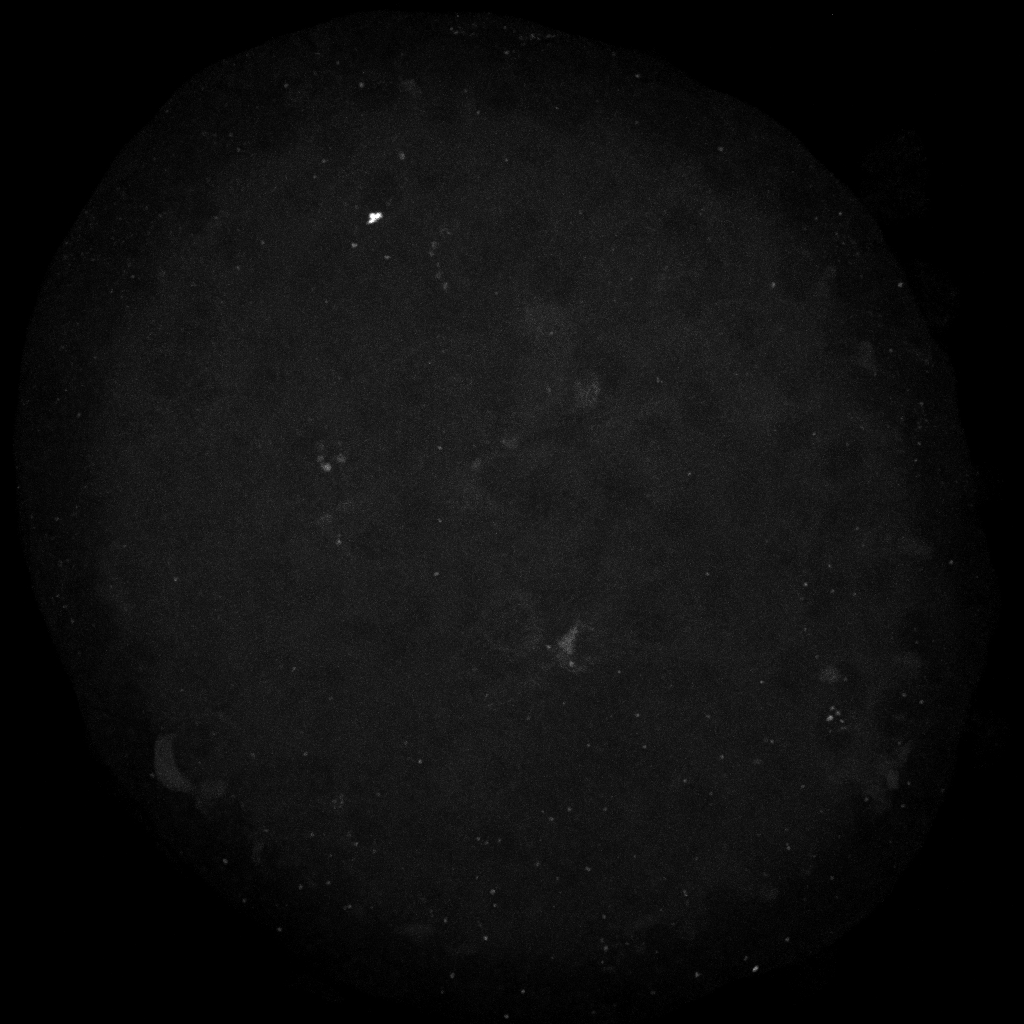

Supplement: Supplementary file 11 — Source data Fig. 6 [file 44318_2025_448_MOESM11_ESM.zip › Figure 6/Fig 6C/INS RNA.tif]

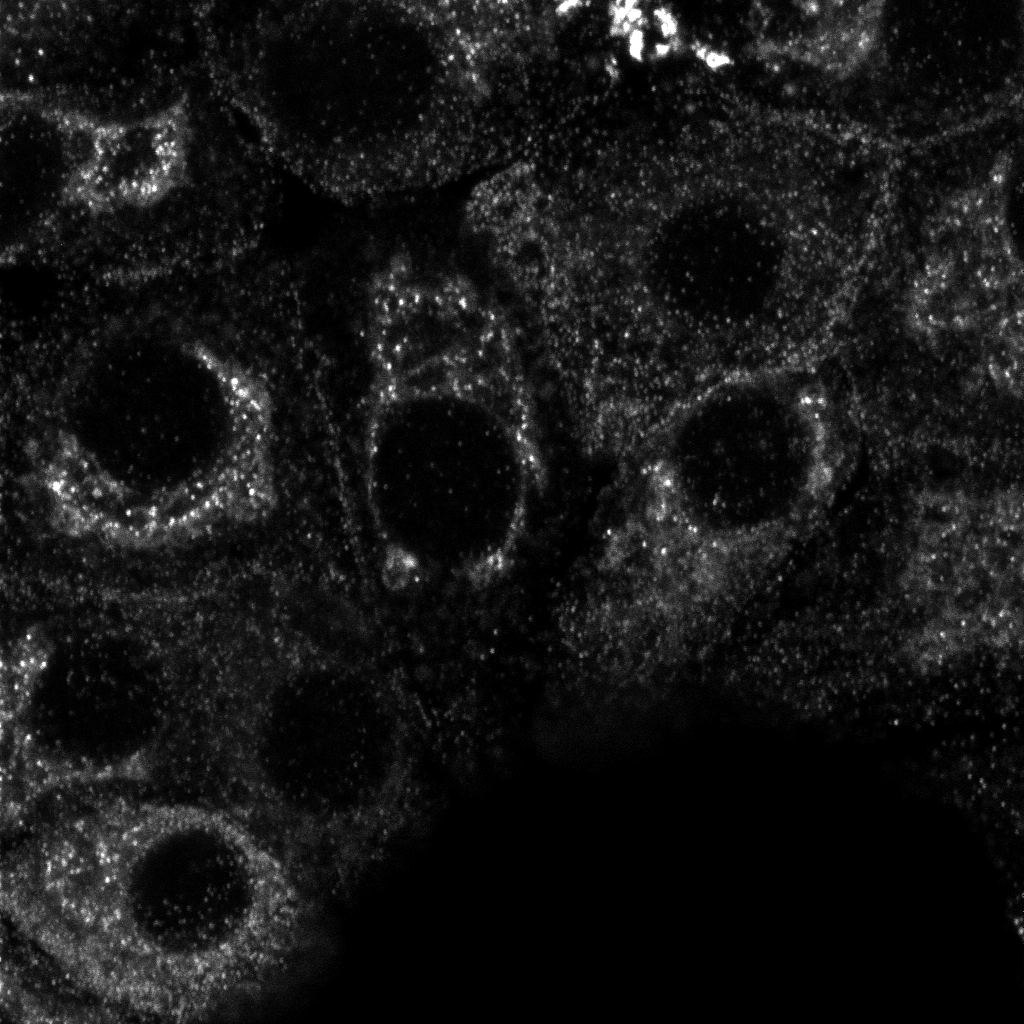

Supplement: Supplementary file 11 — Source data Fig. 6 [file 44318_2025_448_MOESM11_ESM.zip › Figure 6/Fig 6A/2.5 mM/Complete image 2.5 mM Fig 6A.tif]

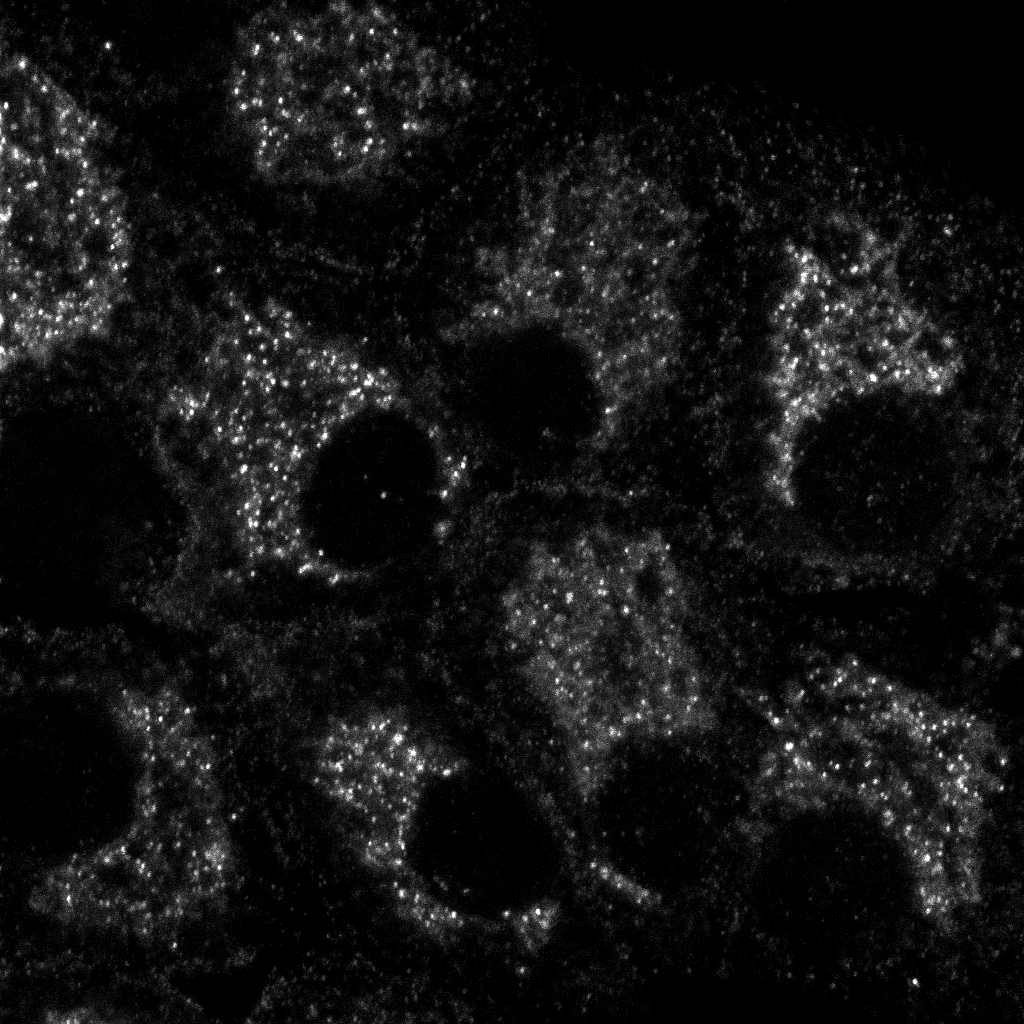

Supplement: Supplementary file 11 — Source data Fig. 6 [file 44318_2025_448_MOESM11_ESM.zip › Figure 6/Fig 6A/16.7 mM/Complete image of 16.7 mM Fig 6A.tif]

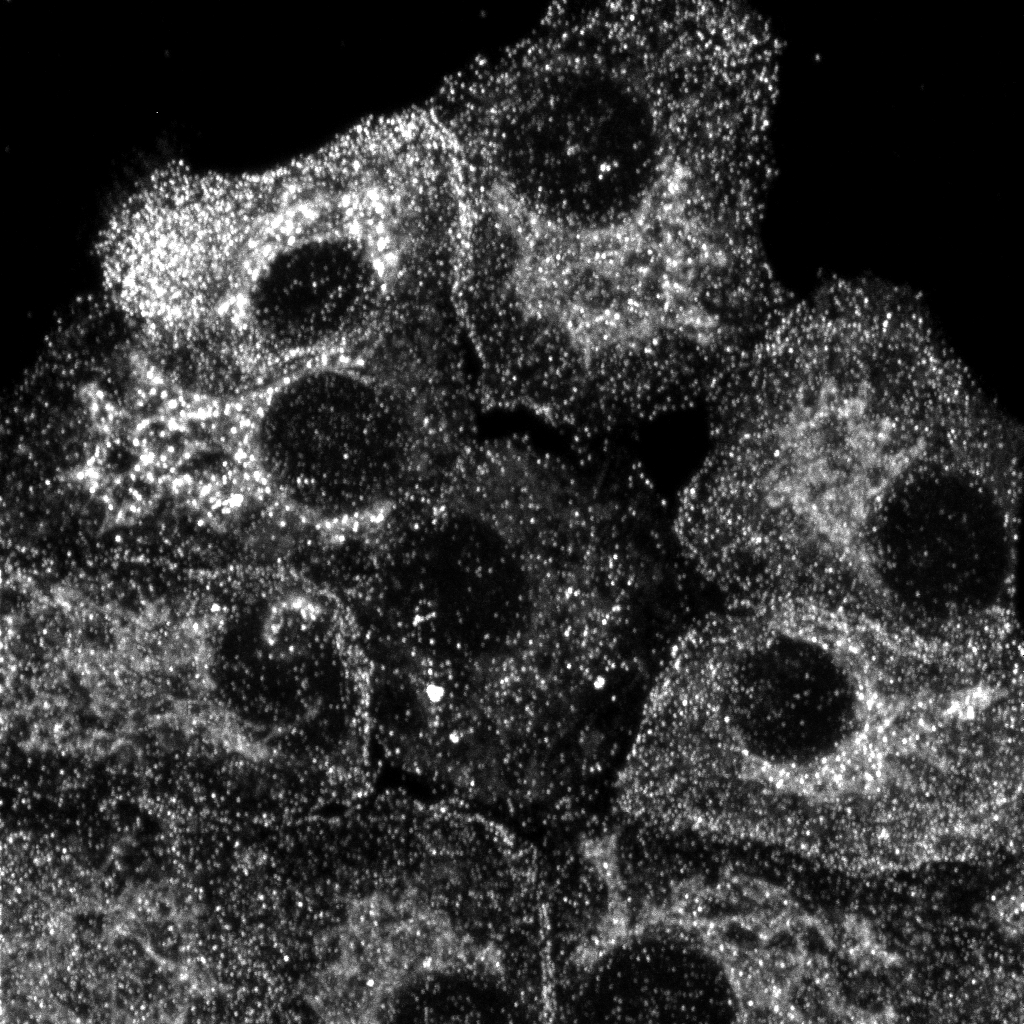

Supplement: Supplementary file 11 — Source data Fig. 6 [file 44318_2025_448_MOESM11_ESM.zip › Figure 6/Fig 6A/3.3 mM/Complete image 3.3 mM Fig 6A.tif]

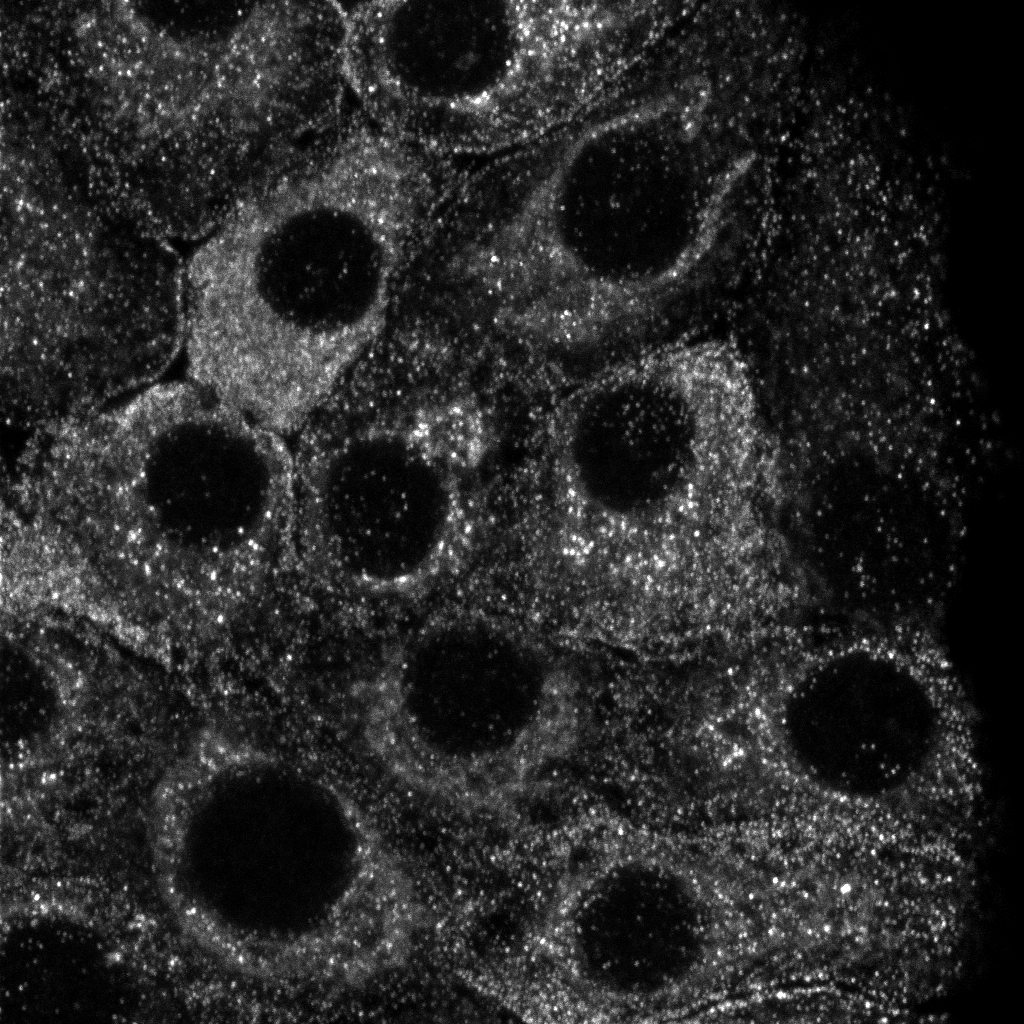

Supplement: Supplementary file 11 — Source data Fig. 6 [file 44318_2025_448_MOESM11_ESM.zip › Figure 6/Fig 6B/2.5 mM/Complete image 2.5 mM Fig 6B.tif]

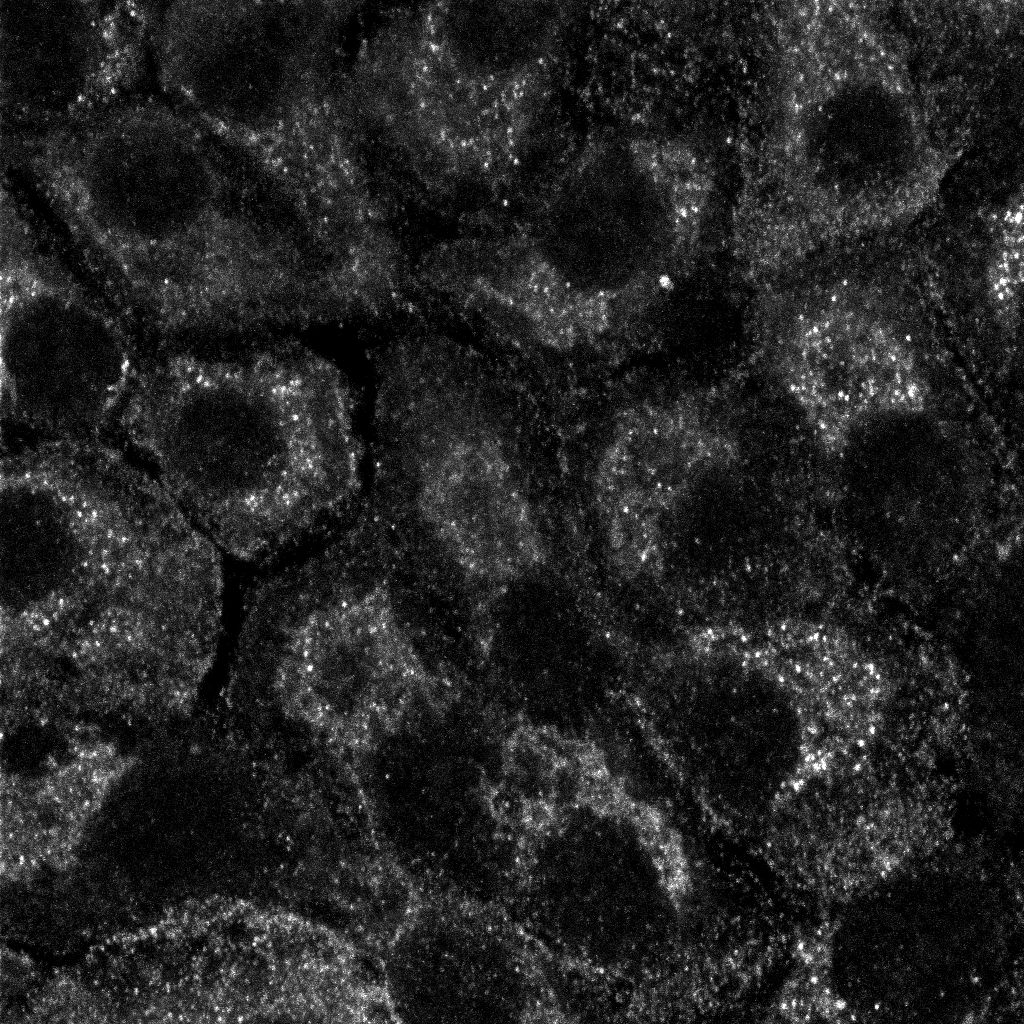

Supplement: Supplementary file 11 — Source data Fig. 6 [file 44318_2025_448_MOESM11_ESM.zip › Figure 6/Fig 6B/16.7 mM/Complete image of 16.7 mM Fig 6B.tif]

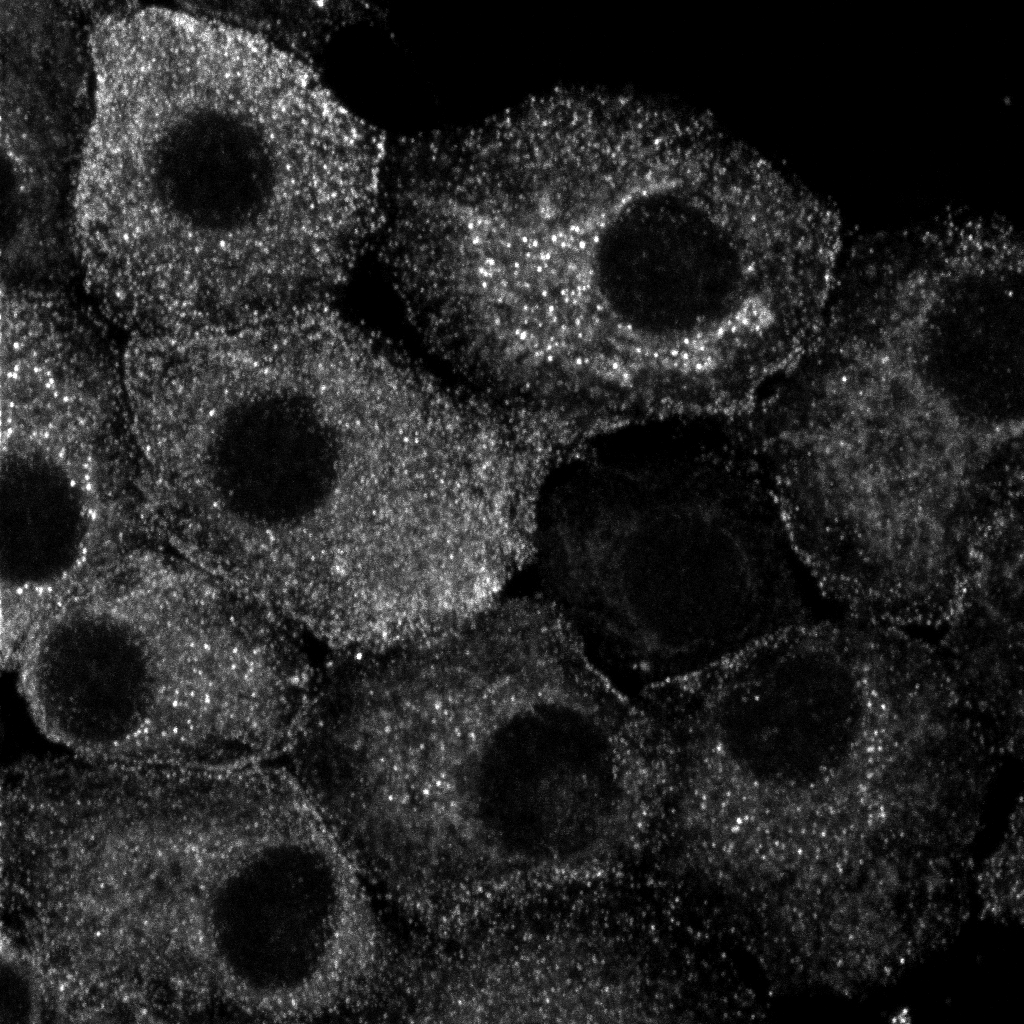

Supplement: Supplementary file 11 — Source data Fig. 6 [file 44318_2025_448_MOESM11_ESM.zip › Figure 6/Fig 6B/3.3 mM/Complete image of 3.3 mM Fig 6B.tif]

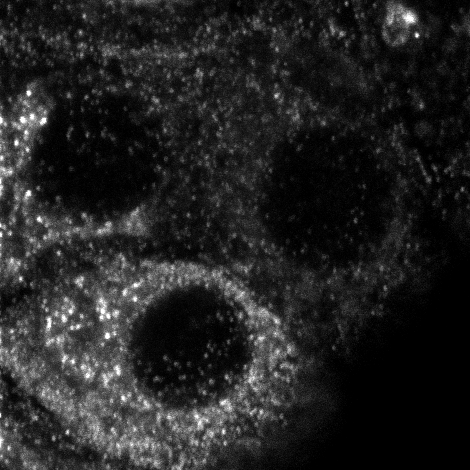

Supplement: Supplementary file 11 — Source data Fig. 6 [file 44318_2025_448_MOESM11_ESM.zip › Figure 6/Fig 6A/2.5 mM/Crop of Complete image 2.5 mM Fig 6A/Composite2.tif]

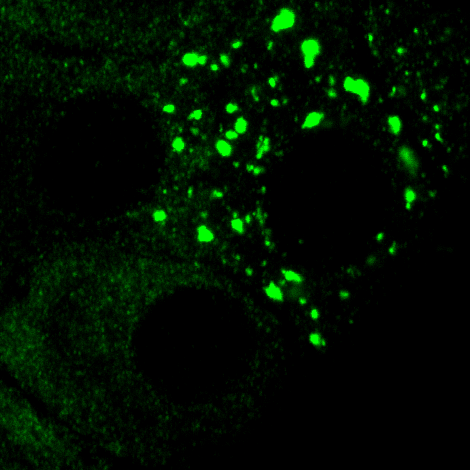

Supplement: Supplementary file 11 — Source data Fig. 6 [file 44318_2025_448_MOESM11_ESM.zip › Figure 6/Fig 6A/2.5 mM/Crop of Complete image 2.5 mM Fig 6A/g3bp1.tif]

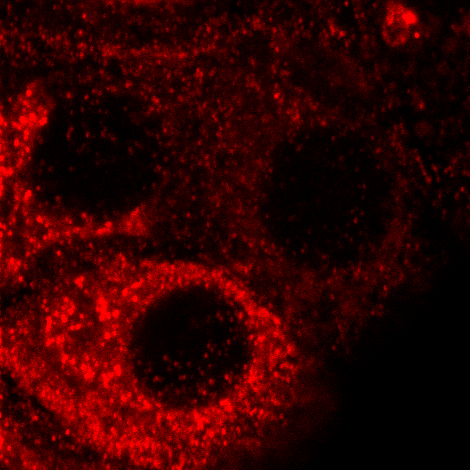

Supplement: Supplementary file 11 — Source data Fig. 6 [file 44318_2025_448_MOESM11_ESM.zip › Figure 6/Fig 6A/2.5 mM/Crop of Complete image 2.5 mM Fig 6A/insulin.tif]

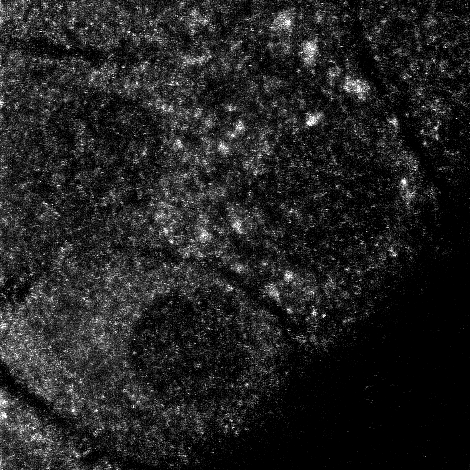

Supplement: Supplementary file 11 — Source data Fig. 6 [file 44318_2025_448_MOESM11_ESM.zip › Figure 6/Fig 6A/2.5 mM/Crop of Complete image 2.5 mM Fig 6A/ins mrna.tif]

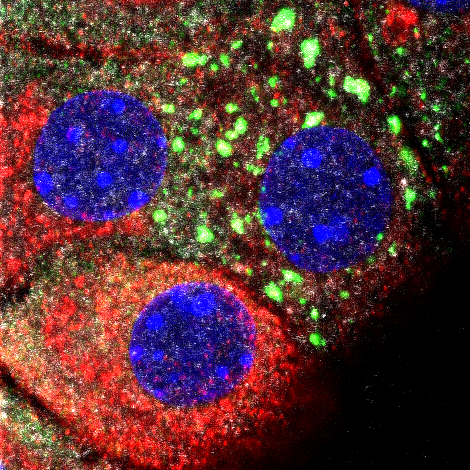

Supplement: Supplementary file 11 — Source data Fig. 6 [file 44318_2025_448_MOESM11_ESM.zip › Figure 6/Fig 6A/2.5 mM/Crop of Complete image 2.5 mM Fig 6A/Composite2.tif (RGB).tif]

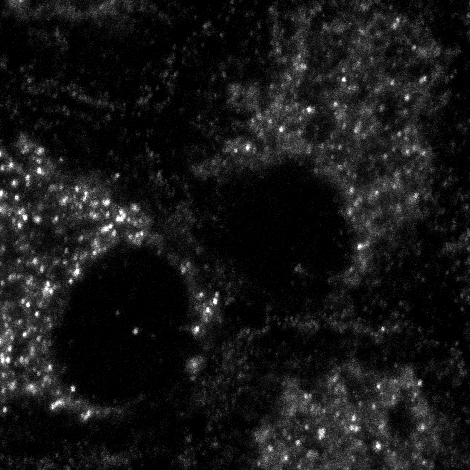

Supplement: Supplementary file 11 — Source data Fig. 6 [file 44318_2025_448_MOESM11_ESM.zip › Figure 6/Fig 6A/16.7 mM/Crop of Complete image of 16.7 mM Fig 6A/Composite2.tif]

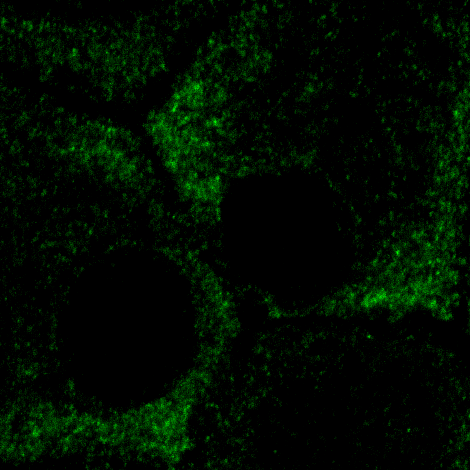

Supplement: Supplementary file 11 — Source data Fig. 6 [file 44318_2025_448_MOESM11_ESM.zip › Figure 6/Fig 6A/16.7 mM/Crop of Complete image of 16.7 mM Fig 6A/g3bop1.tif]

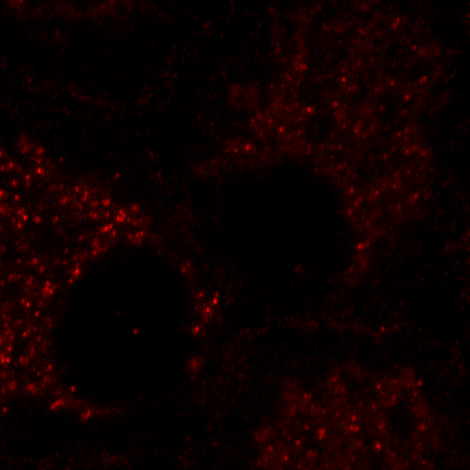

Supplement: Supplementary file 11 — Source data Fig. 6 [file 44318_2025_448_MOESM11_ESM.zip › Figure 6/Fig 6A/16.7 mM/Crop of Complete image of 16.7 mM Fig 6A/insulin.tif]

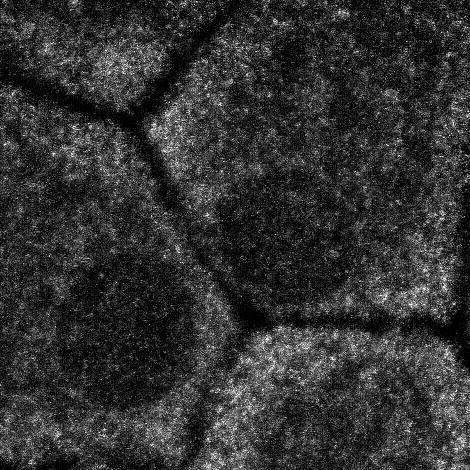

Supplement: Supplementary file 11 — Source data Fig. 6 [file 44318_2025_448_MOESM11_ESM.zip › Figure 6/Fig 6A/16.7 mM/Crop of Complete image of 16.7 mM Fig 6A/ins mrna.tif]

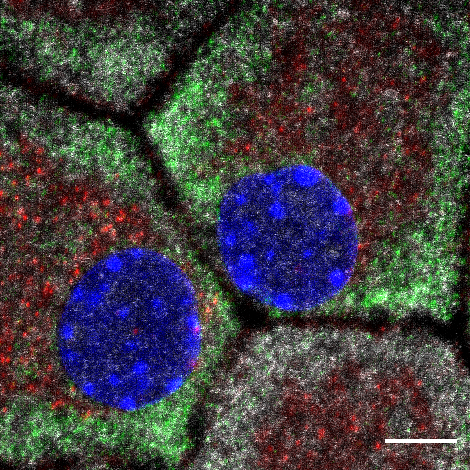

Supplement: Supplementary file 11 — Source data Fig. 6 [file 44318_2025_448_MOESM11_ESM.zip › Figure 6/Fig 6A/16.7 mM/Crop of Complete image of 16.7 mM Fig 6A/Composite2.tif (RGB).tif]

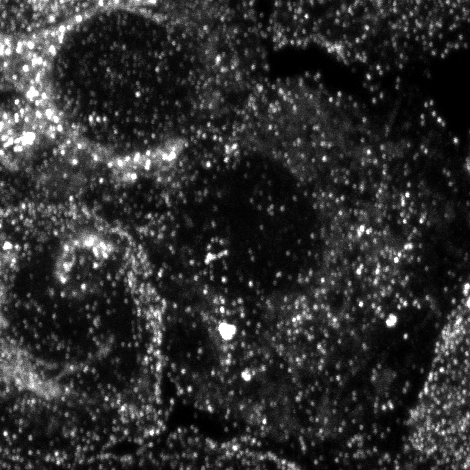

Supplement: Supplementary file 11 — Source data Fig. 6 [file 44318_2025_448_MOESM11_ESM.zip › Figure 6/Fig 6A/3.3 mM/Crop of Complete image 3.3 mM Fig 6A/Composite2.tif]

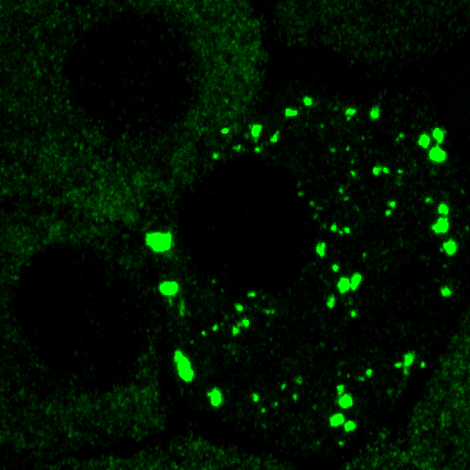

Supplement: Supplementary file 11 — Source data Fig. 6 [file 44318_2025_448_MOESM11_ESM.zip › Figure 6/Fig 6A/3.3 mM/Crop of Complete image 3.3 mM Fig 6A/g3bp1.tif]

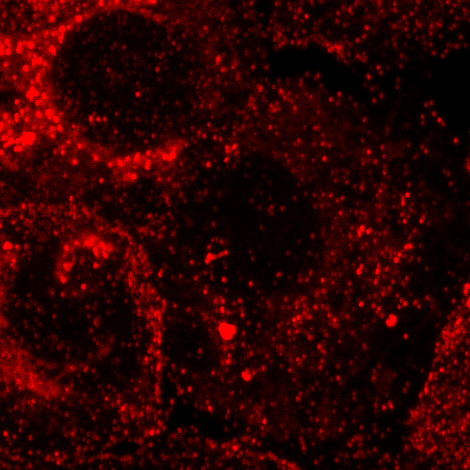

Supplement: Supplementary file 11 — Source data Fig. 6 [file 44318_2025_448_MOESM11_ESM.zip › Figure 6/Fig 6A/3.3 mM/Crop of Complete image 3.3 mM Fig 6A/insulin.tif]

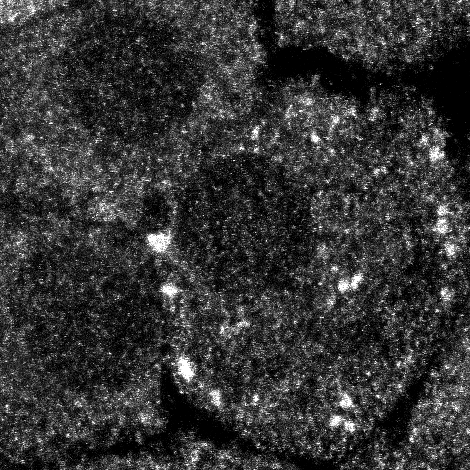

Supplement: Supplementary file 11 — Source data Fig. 6 [file 44318_2025_448_MOESM11_ESM.zip › Figure 6/Fig 6A/3.3 mM/Crop of Complete image 3.3 mM Fig 6A/ins mrna.tif]

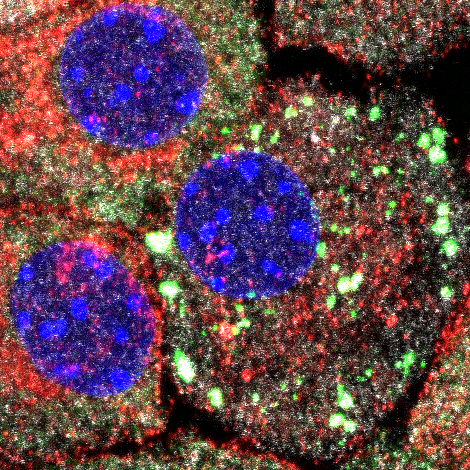

Supplement: Supplementary file 11 — Source data Fig. 6 [file 44318_2025_448_MOESM11_ESM.zip › Figure 6/Fig 6A/3.3 mM/Crop of Complete image 3.3 mM Fig 6A/Composite2.tif (RGB).tif]

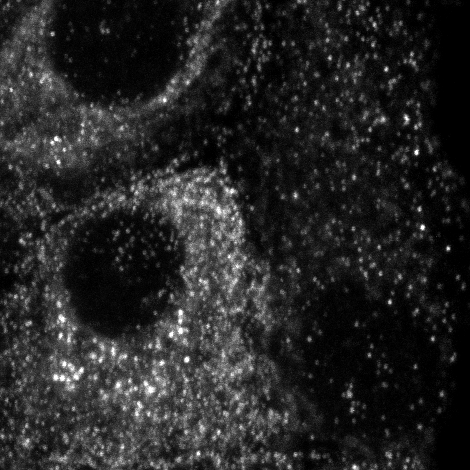

Supplement: Supplementary file 11 — Source data Fig. 6 [file 44318_2025_448_MOESM11_ESM.zip › Figure 6/Fig 6B/2.5 mM/Crop of Complete image 2.5 mM Fig 6B/Composite2.tif]

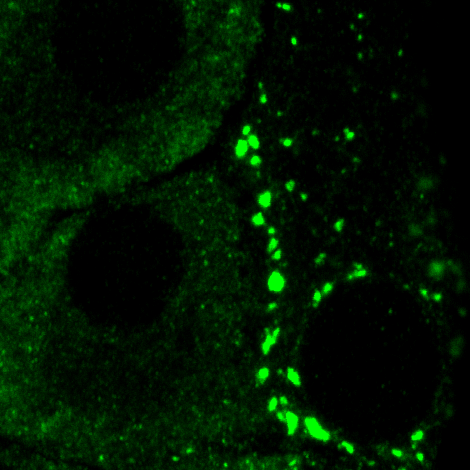

Supplement: Supplementary file 11 — Source data Fig. 6 [file 44318_2025_448_MOESM11_ESM.zip › Figure 6/Fig 6B/2.5 mM/Crop of Complete image 2.5 mM Fig 6B/g3bp2.tif]

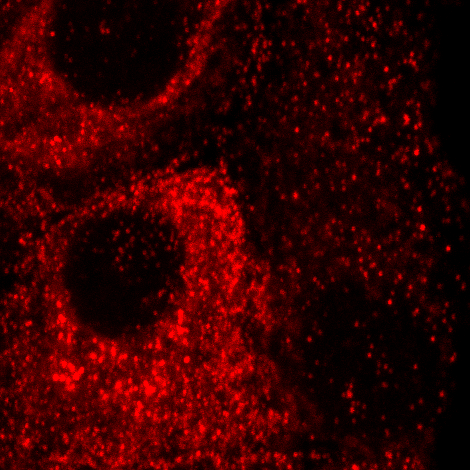

Supplement: Supplementary file 11 — Source data Fig. 6 [file 44318_2025_448_MOESM11_ESM.zip › Figure 6/Fig 6B/2.5 mM/Crop of Complete image 2.5 mM Fig 6B/insulin.tif]

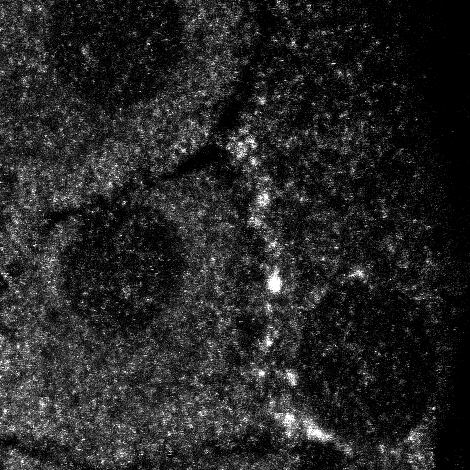

Supplement: Supplementary file 11 — Source data Fig. 6 [file 44318_2025_448_MOESM11_ESM.zip › Figure 6/Fig 6B/2.5 mM/Crop of Complete image 2.5 mM Fig 6B/ins mrna.tif]

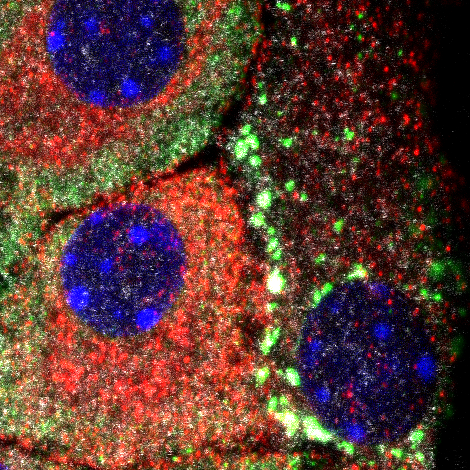

Supplement: Supplementary file 11 — Source data Fig. 6 [file 44318_2025_448_MOESM11_ESM.zip › Figure 6/Fig 6B/2.5 mM/Crop of Complete image 2.5 mM Fig 6B/Composite2.tif (RGB).tif]

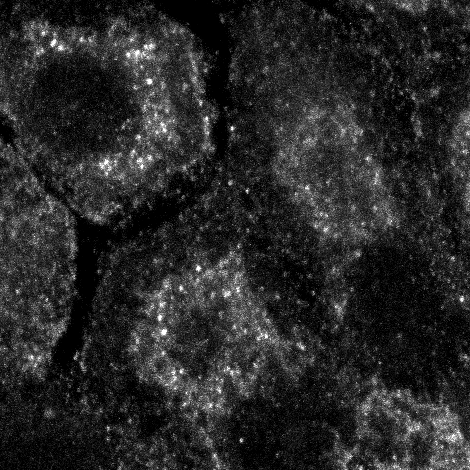

Supplement: Supplementary file 11 — Source data Fig. 6 [file 44318_2025_448_MOESM11_ESM.zip › Figure 6/Fig 6B/16.7 mM/Crop of Complete image of 16.7 mM Fig 6B/Composite2.tif]

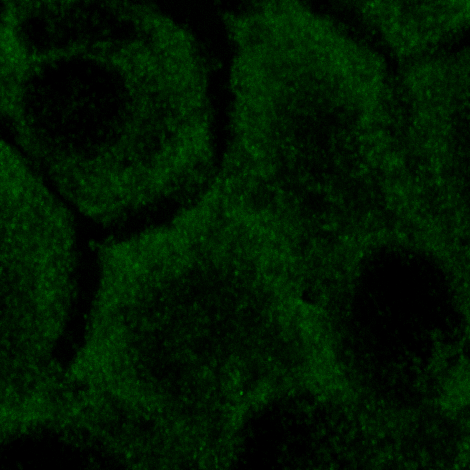

Supplement: Supplementary file 11 — Source data Fig. 6 [file 44318_2025_448_MOESM11_ESM.zip › Figure 6/Fig 6B/16.7 mM/Crop of Complete image of 16.7 mM Fig 6B/g3bp2.tif]

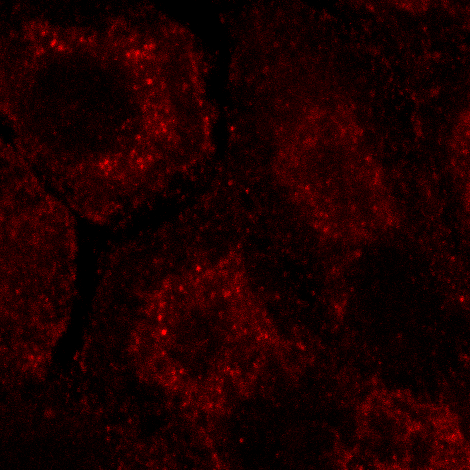

Supplement: Supplementary file 11 — Source data Fig. 6 [file 44318_2025_448_MOESM11_ESM.zip › Figure 6/Fig 6B/16.7 mM/Crop of Complete image of 16.7 mM Fig 6B/insulin.tif]

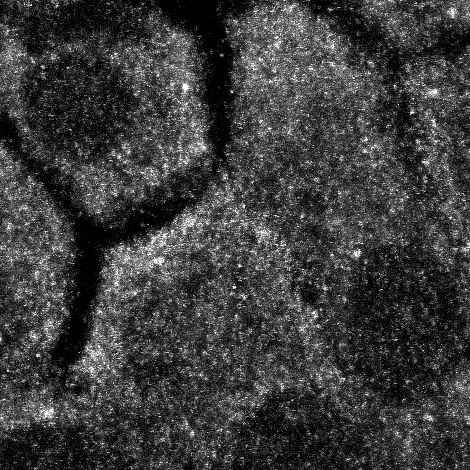

Supplement: Supplementary file 11 — Source data Fig. 6 [file 44318_2025_448_MOESM11_ESM.zip › Figure 6/Fig 6B/16.7 mM/Crop of Complete image of 16.7 mM Fig 6B/ins mrna.tif]

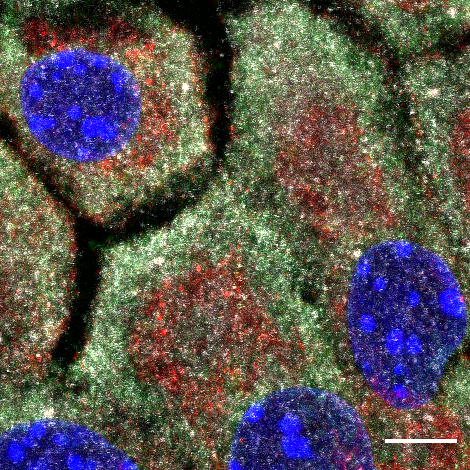

Supplement: Supplementary file 11 — Source data Fig. 6 [file 44318_2025_448_MOESM11_ESM.zip › Figure 6/Fig 6B/16.7 mM/Crop of Complete image of 16.7 mM Fig 6B/Composite2.tif (RGB).tif]

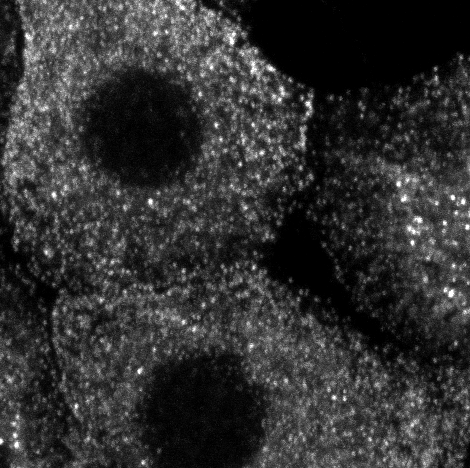

Supplement: Supplementary file 11 — Source data Fig. 6 [file 44318_2025_448_MOESM11_ESM.zip › Figure 6/Fig 6B/3.3 mM/Crop of Complete image of 3.3 mM Fig 6B/Composite2.tif]

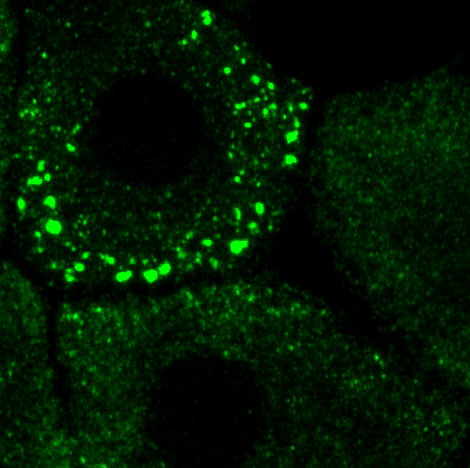

Supplement: Supplementary file 11 — Source data Fig. 6 [file 44318_2025_448_MOESM11_ESM.zip › Figure 6/Fig 6B/3.3 mM/Crop of Complete image of 3.3 mM Fig 6B/g3bp2.tif]

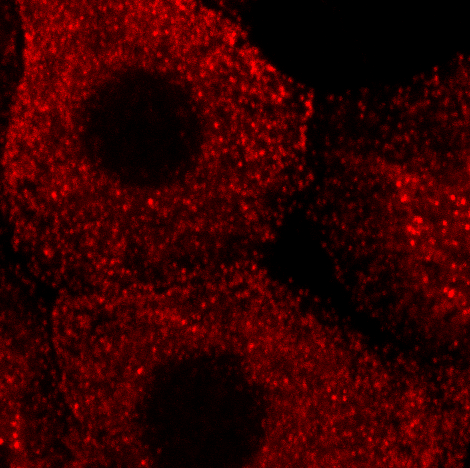

Supplement: Supplementary file 11 — Source data Fig. 6 [file 44318_2025_448_MOESM11_ESM.zip › Figure 6/Fig 6B/3.3 mM/Crop of Complete image of 3.3 mM Fig 6B/insulin.tif]

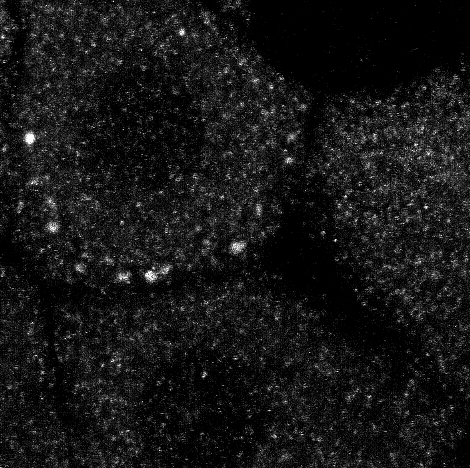

Supplement: Supplementary file 11 — Source data Fig. 6 [file 44318_2025_448_MOESM11_ESM.zip › Figure 6/Fig 6B/3.3 mM/Crop of Complete image of 3.3 mM Fig 6B/ins mrna.tif]

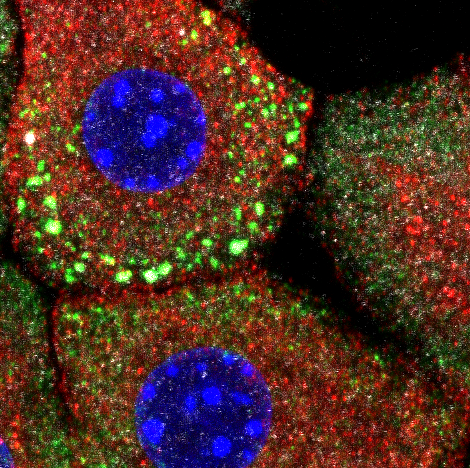

Supplement: Supplementary file 11 — Source data Fig. 6 [file 44318_2025_448_MOESM11_ESM.zip › Figure 6/Fig 6B/3.3 mM/Crop of Complete image of 3.3 mM Fig 6B/Composite2.tif (RGB).tif]

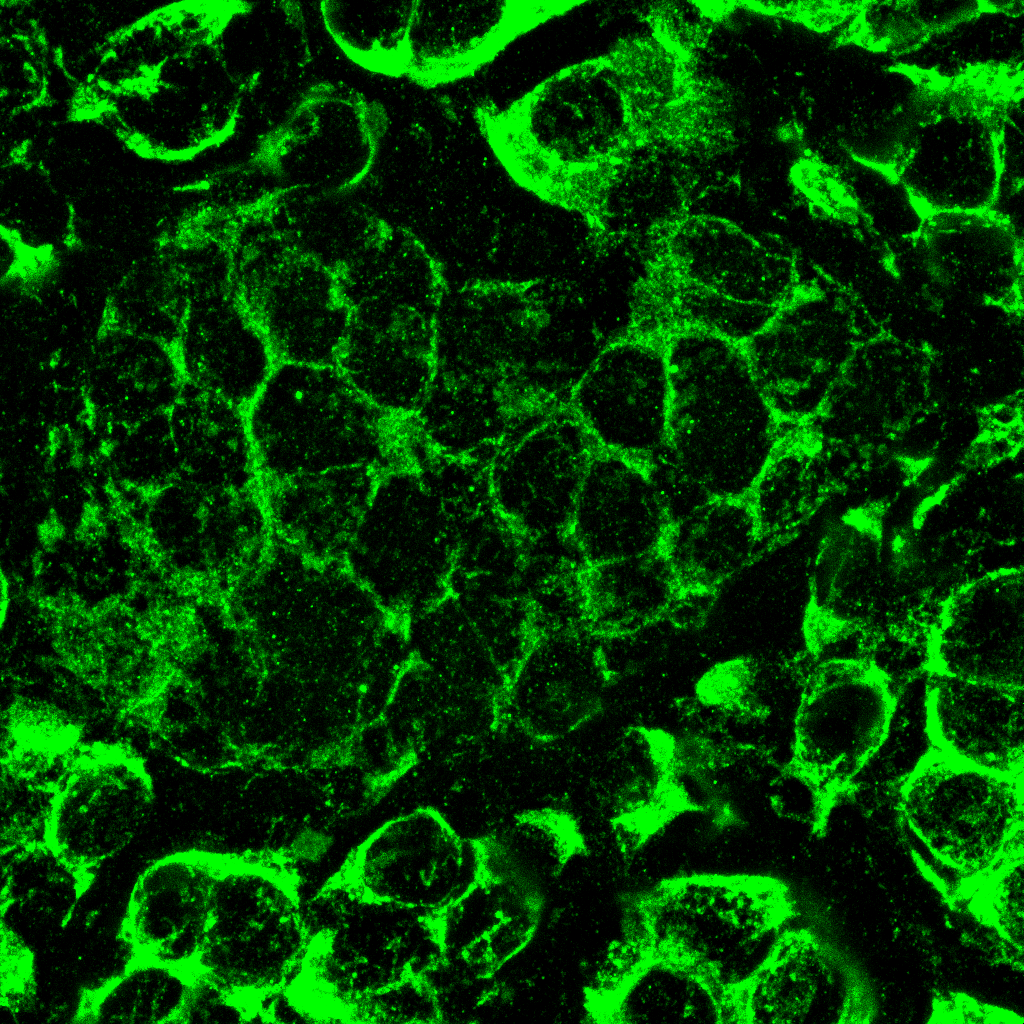

Supplement: Supplementary file 12 — Source data Fig. 7 [file 44318_2025_448_MOESM12_ESM.zip › Figure 7/Fig 7A/NLD1/g3bp1.tif]

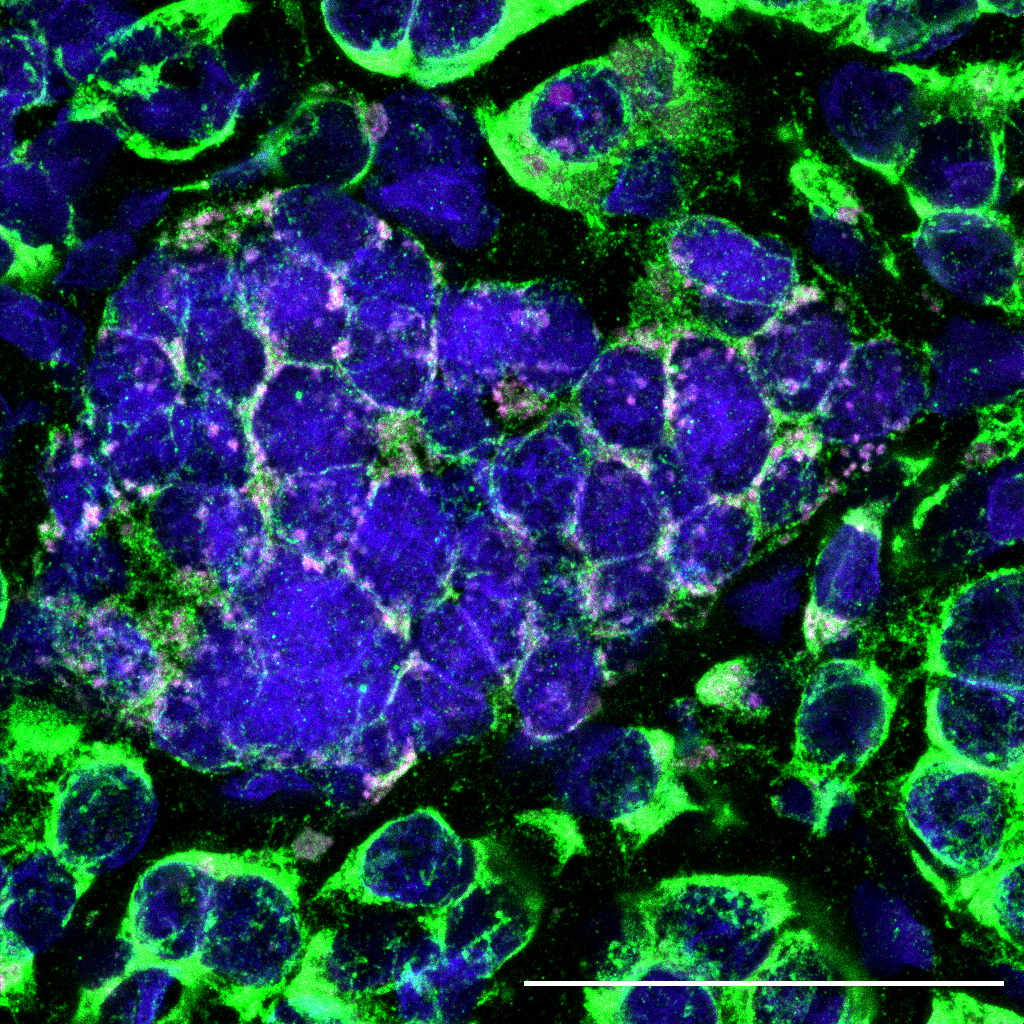

Supplement: Supplementary file 12 — Source data Fig. 7 [file 44318_2025_448_MOESM12_ESM.zip › Figure 7/Fig 7A/NLD1/Composite-1.tif (RGB).tif]

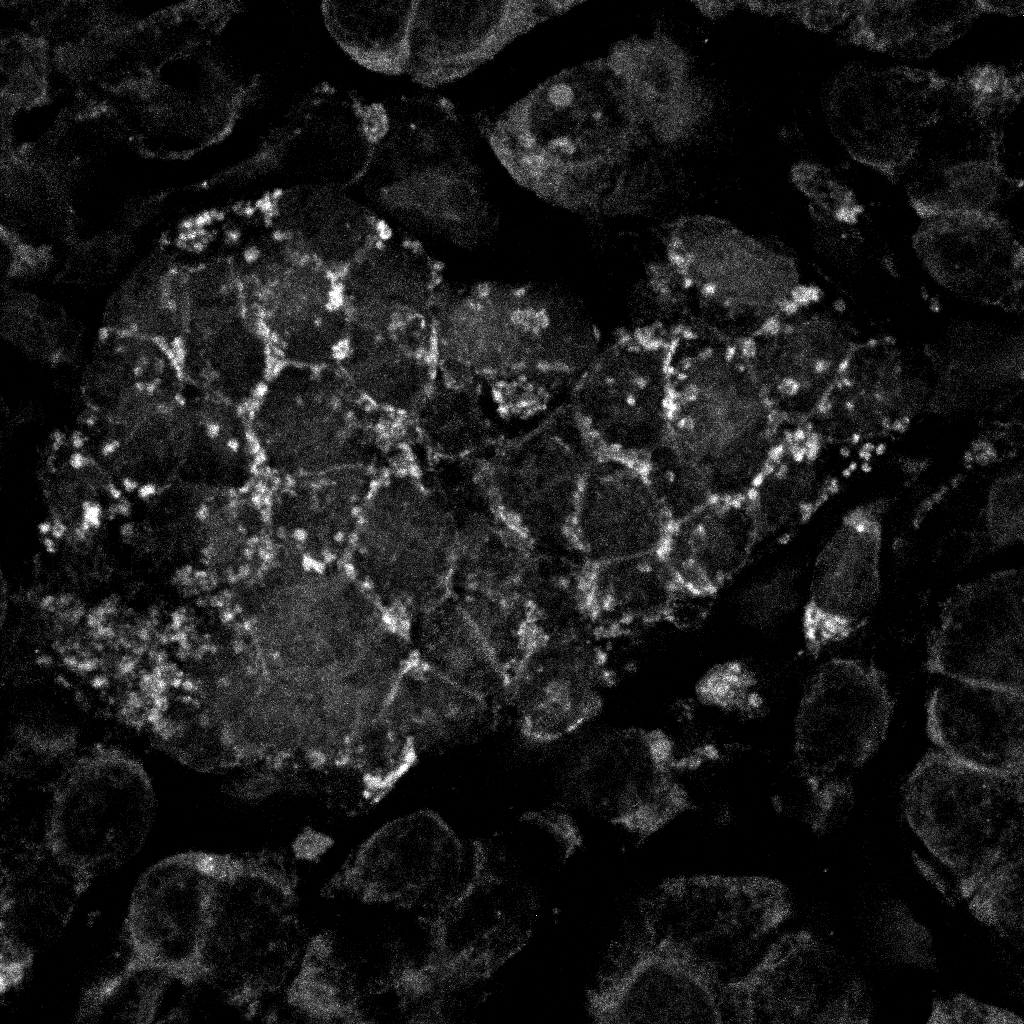

Supplement: Supplementary file 12 — Source data Fig. 7 [file 44318_2025_448_MOESM12_ESM.zip › Figure 7/Fig 7A/NLD1/Composite-1.tif]

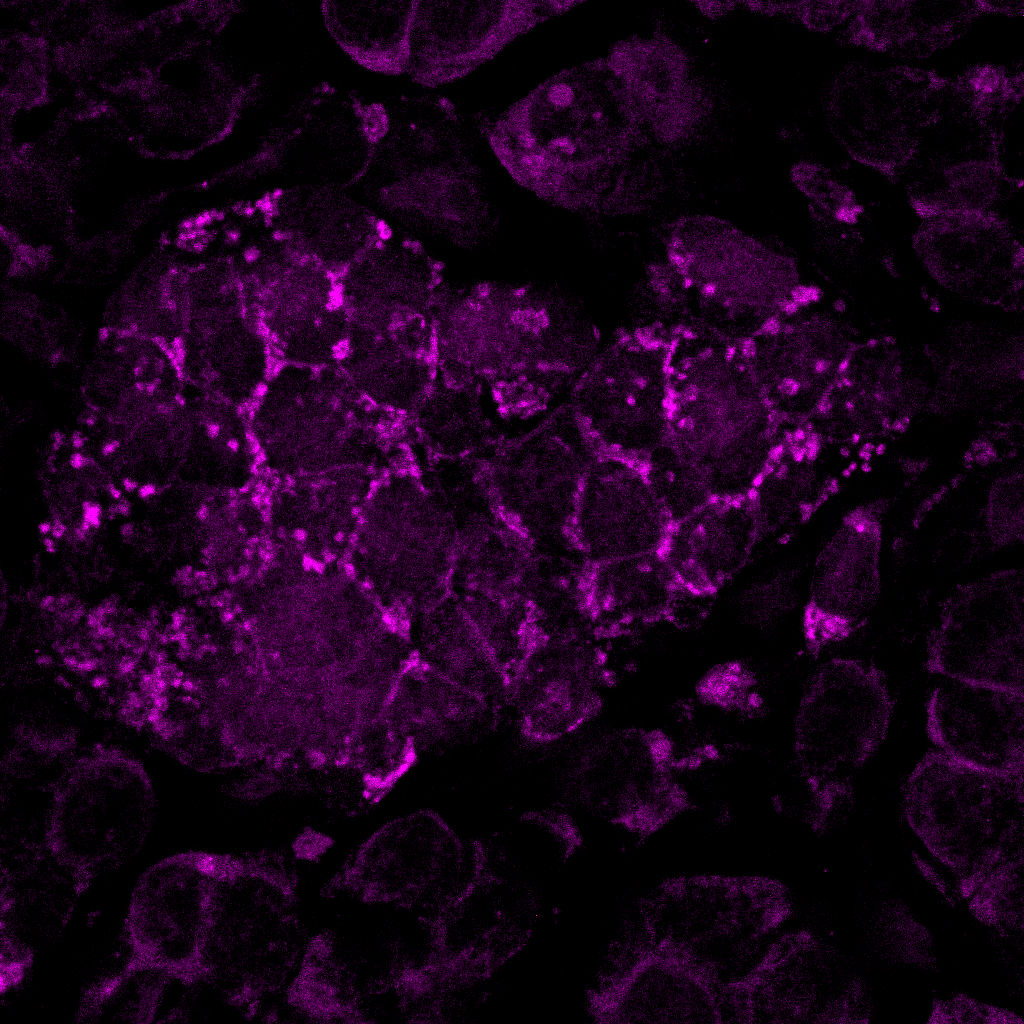

Supplement: Supplementary file 12 — Source data Fig. 7 [file 44318_2025_448_MOESM12_ESM.zip › Figure 7/Fig 7A/NLD1/ins rna.tif]

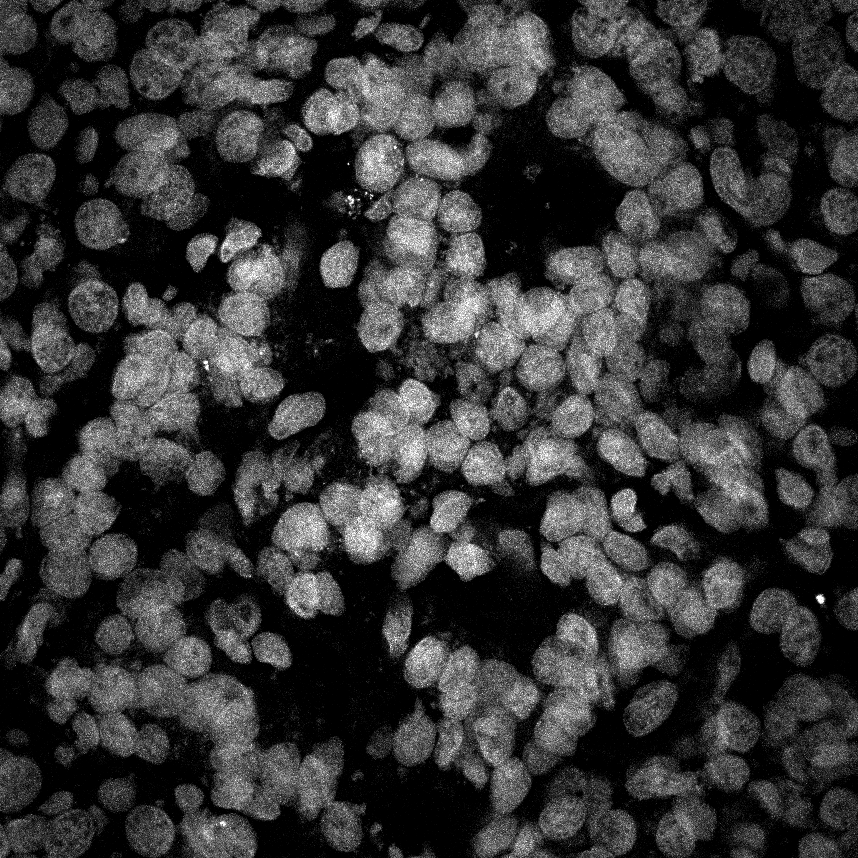

Supplement: Supplementary file 12 — Source data Fig. 7 [file 44318_2025_448_MOESM12_ESM.zip › Figure 7/Fig 7A/NLD3/Composite.tif]

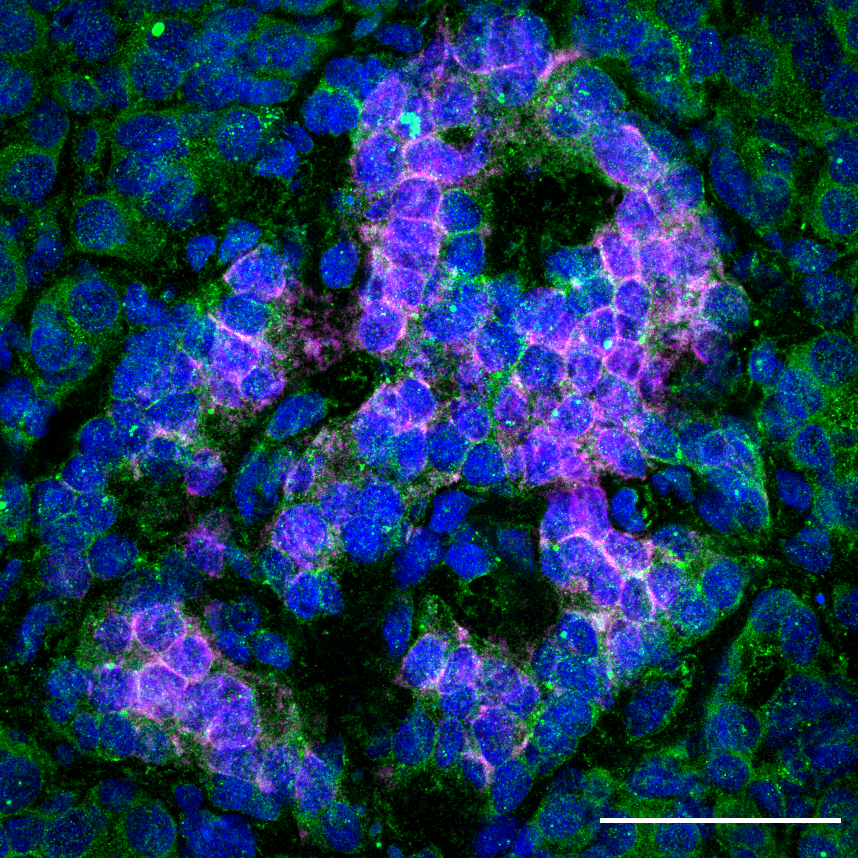

Supplement: Supplementary file 12 — Source data Fig. 7 [file 44318_2025_448_MOESM12_ESM.zip › Figure 7/Fig 7A/NLD3/Composite.tif (RGB).tif]

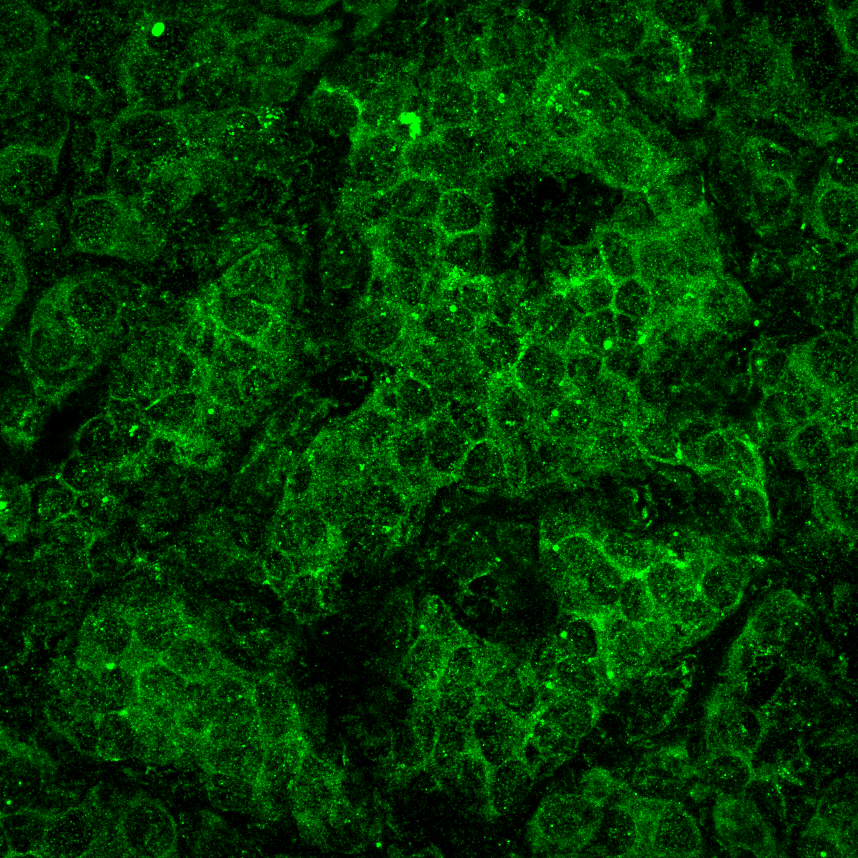

Supplement: Supplementary file 12 — Source data Fig. 7 [file 44318_2025_448_MOESM12_ESM.zip › Figure 7/Fig 7A/NLD3/g3bp1.tif]

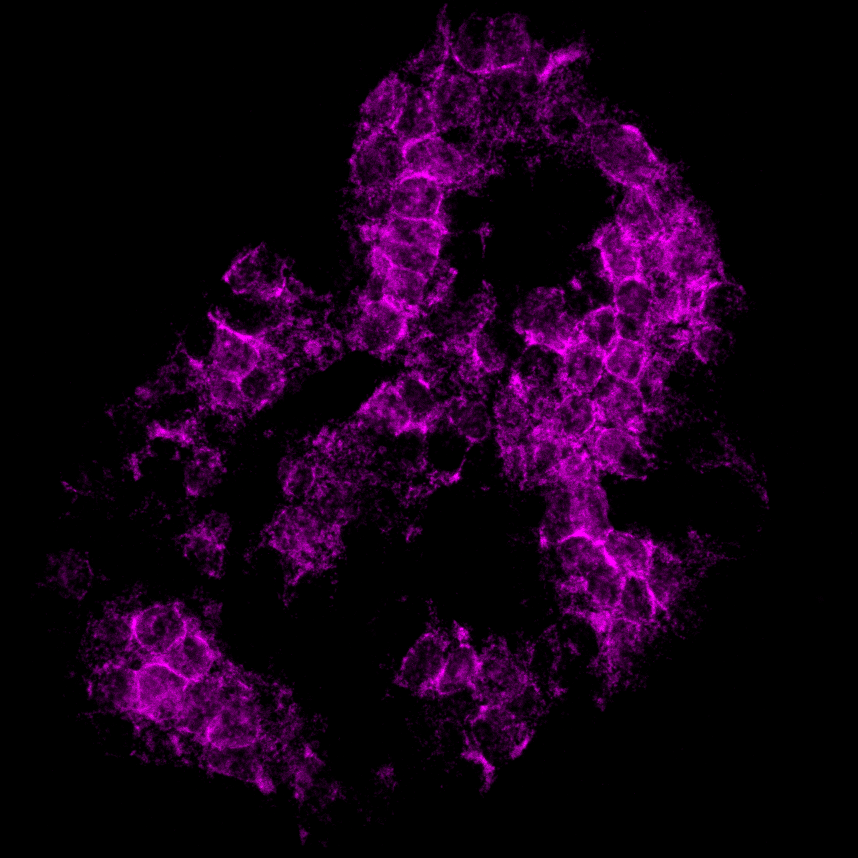

Supplement: Supplementary file 12 — Source data Fig. 7 [file 44318_2025_448_MOESM12_ESM.zip › Figure 7/Fig 7A/NLD3/ins rna.tif]

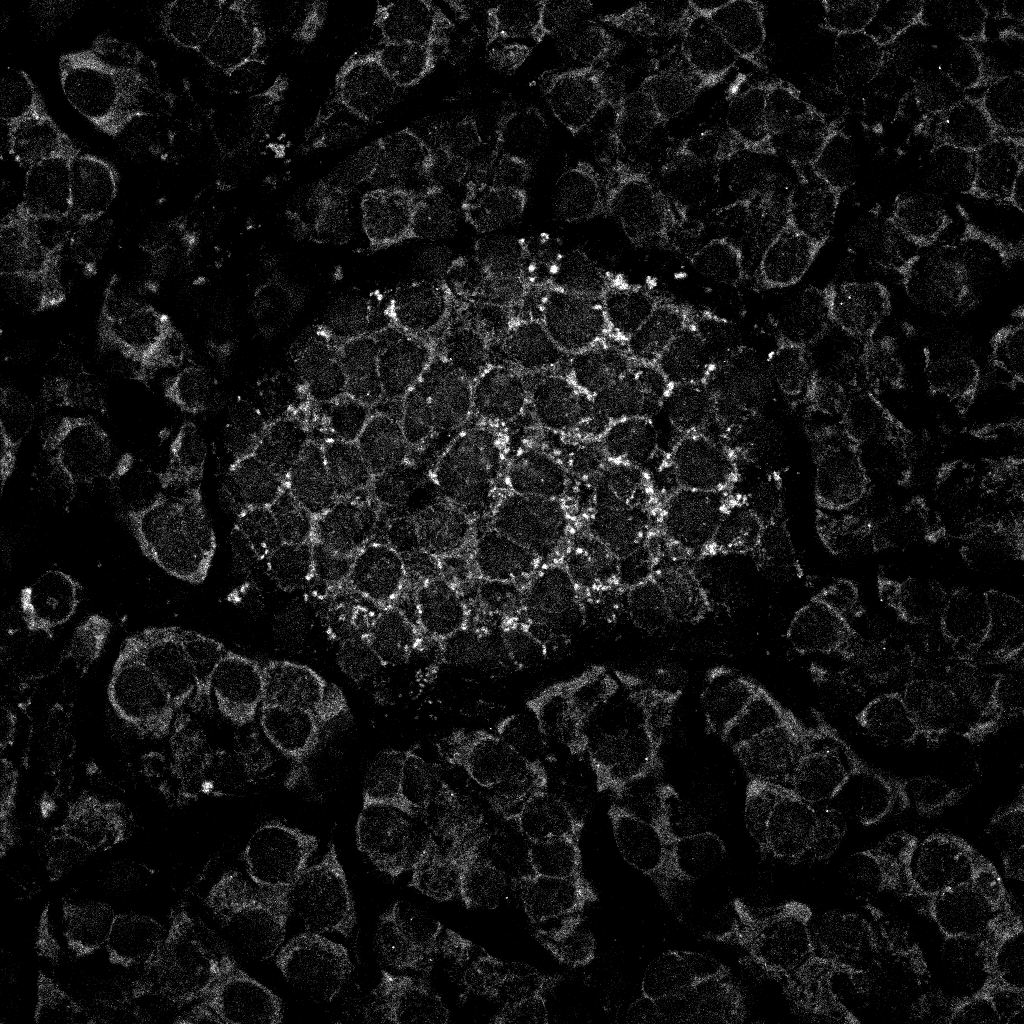

Supplement: Supplementary file 12 — Source data Fig. 7 [file 44318_2025_448_MOESM12_ESM.zip › Figure 7/Fig 7A/NLD2/Composite.tif]

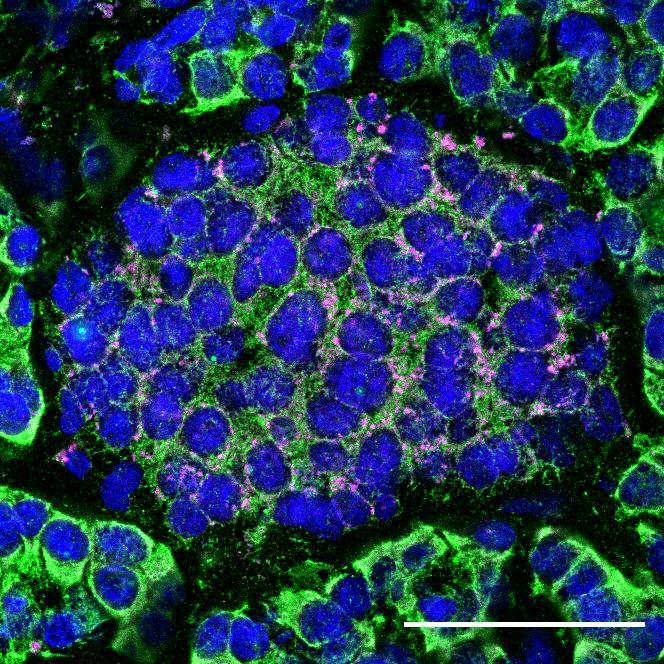

Supplement: Supplementary file 12 — Source data Fig. 7 [file 44318_2025_448_MOESM12_ESM.zip › Figure 7/Fig 7A/NLD2/Composite.tif (RGB).tif]

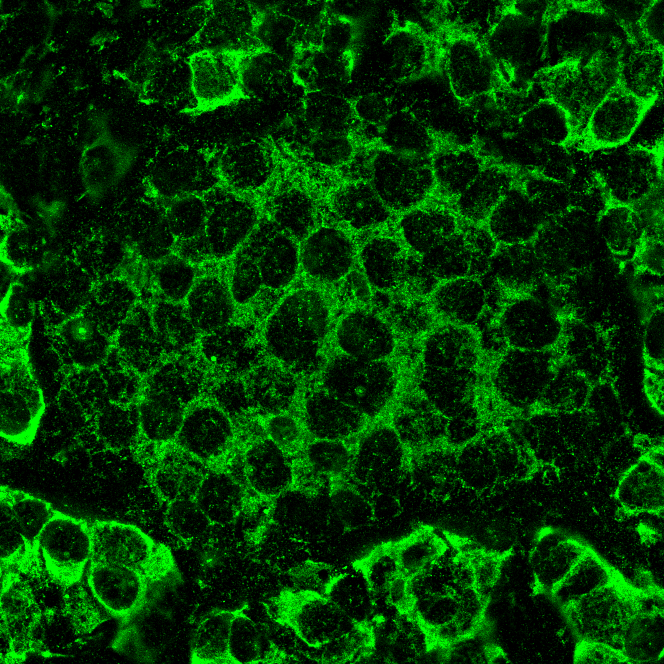

Supplement: Supplementary file 12 — Source data Fig. 7 [file 44318_2025_448_MOESM12_ESM.zip › Figure 7/Fig 7A/NLD2/g3bp1.tif]

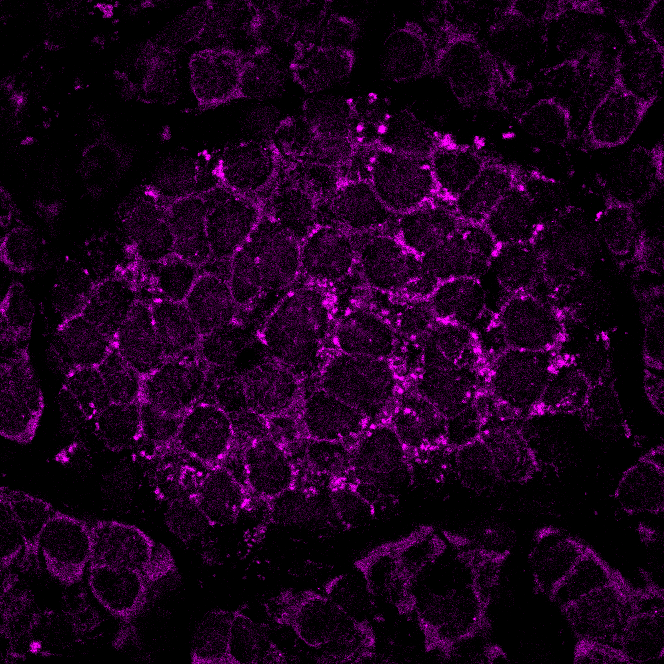

Supplement: Supplementary file 12 — Source data Fig. 7 [file 44318_2025_448_MOESM12_ESM.zip › Figure 7/Fig 7A/NLD2/ins rna.tif]

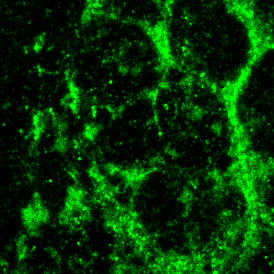

Supplement: Supplementary file 12 — Source data Fig. 7 [file 44318_2025_448_MOESM12_ESM.zip › Figure 7/Fig 7A/NLD1/1/G3BP1.tif]

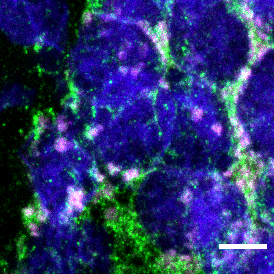

Supplement: Supplementary file 12 — Source data Fig. 7 [file 44318_2025_448_MOESM12_ESM.zip › Figure 7/Fig 7A/NLD1/1/Composite-1.tif (RGB).tif]

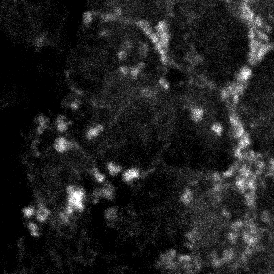

Supplement: Supplementary file 12 — Source data Fig. 7 [file 44318_2025_448_MOESM12_ESM.zip › Figure 7/Fig 7A/NLD1/1/Composite-1.tif]

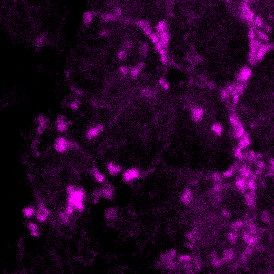

Supplement: Supplementary file 12 — Source data Fig. 7 [file 44318_2025_448_MOESM12_ESM.zip › Figure 7/Fig 7A/NLD1/1/INS RNA.tif]

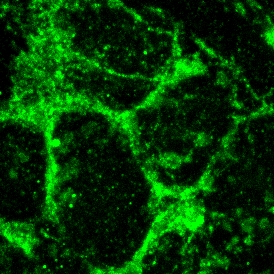

Supplement: Supplementary file 12 — Source data Fig. 7 [file 44318_2025_448_MOESM12_ESM.zip › Figure 7/Fig 7A/NLD1/2/g3bp1.tif]
