# Supplementary figures and images for: Aldolase-regulated G3BP1/2+ condensates control insulin mRNA storage in beta cells (part 4 of 4)
Source: EMBO J. 2025 May 12;44(13):3669–96. doi: 10.1038/s44318-025-00448-7 (PMC12216156; doi:10.1038/s44318-025-00448-7)

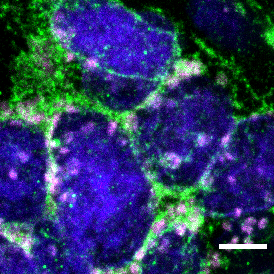

Supplement: Supplementary file 12 — Source data Fig. 7 [file 44318_2025_448_MOESM12_ESM.zip › Figure 7/Fig 7A/NLD1/2/Composite-1.tif (RGB).tif]

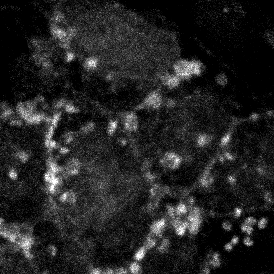

Supplement: Supplementary file 12 — Source data Fig. 7 [file 44318_2025_448_MOESM12_ESM.zip › Figure 7/Fig 7A/NLD1/2/Composite-1.tif]

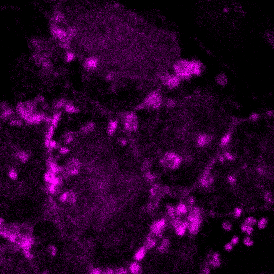

Supplement: Supplementary file 12 — Source data Fig. 7 [file 44318_2025_448_MOESM12_ESM.zip › Figure 7/Fig 7A/NLD1/2/ins rna.tif]

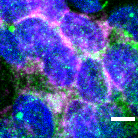

Supplement: Supplementary file 12 — Source data Fig. 7 [file 44318_2025_448_MOESM12_ESM.zip › Figure 7/Fig 7A/NLD3/crop/1/Composite.tif (RGB).tif]

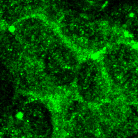

Supplement: Supplementary file 12 — Source data Fig. 7 [file 44318_2025_448_MOESM12_ESM.zip › Figure 7/Fig 7A/NLD3/crop/1/g3bp1.tif]

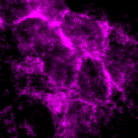

Supplement: Supplementary file 12 — Source data Fig. 7 [file 44318_2025_448_MOESM12_ESM.zip › Figure 7/Fig 7A/NLD3/crop/1/ins rna.tif]

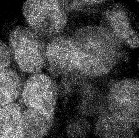

Supplement: Supplementary file 12 — Source data Fig. 7 [file 44318_2025_448_MOESM12_ESM.zip › Figure 7/Fig 7A/NLD3/crop/2/Composite.tif]

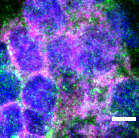

Supplement: Supplementary file 12 — Source data Fig. 7 [file 44318_2025_448_MOESM12_ESM.zip › Figure 7/Fig 7A/NLD3/crop/2/Composite.tif (RGB).tif]

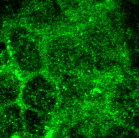

Supplement: Supplementary file 12 — Source data Fig. 7 [file 44318_2025_448_MOESM12_ESM.zip › Figure 7/Fig 7A/NLD3/crop/2/g3bp1.tif]

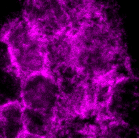

Supplement: Supplementary file 12 — Source data Fig. 7 [file 44318_2025_448_MOESM12_ESM.zip › Figure 7/Fig 7A/NLD3/crop/2/ins rna.tif]

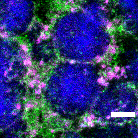

Supplement: Supplementary file 12 — Source data Fig. 7 [file 44318_2025_448_MOESM12_ESM.zip › Figure 7/Fig 7A/NLD2/crops/1/Composite crop 1(RGB).tif]

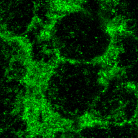

Supplement: Supplementary file 12 — Source data Fig. 7 [file 44318_2025_448_MOESM12_ESM.zip › Figure 7/Fig 7A/NLD2/crops/1/g3bp1.tif]

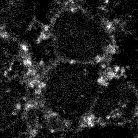

Supplement: Supplementary file 12 — Source data Fig. 7 [file 44318_2025_448_MOESM12_ESM.zip › Figure 7/Fig 7A/NLD2/crops/1/Composite crop 1.tif]

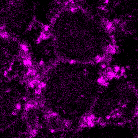

Supplement: Supplementary file 12 — Source data Fig. 7 [file 44318_2025_448_MOESM12_ESM.zip › Figure 7/Fig 7A/NLD2/crops/1/ins rna.tif]

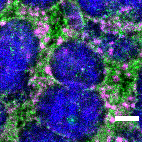

Supplement: Supplementary file 12 — Source data Fig. 7 [file 44318_2025_448_MOESM12_ESM.zip › Figure 7/Fig 7A/NLD2/crops/2/Composite.tif (RGB).tif]

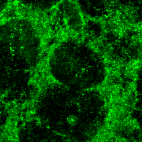

Supplement: Supplementary file 12 — Source data Fig. 7 [file 44318_2025_448_MOESM12_ESM.zip › Figure 7/Fig 7A/NLD2/crops/2/g3bp1.tif]

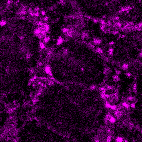

Supplement: Supplementary file 12 — Source data Fig. 7 [file 44318_2025_448_MOESM12_ESM.zip › Figure 7/Fig 7A/NLD2/crops/2/ins rna.tif]
