# Supplementary material for: Aldolase-regulated G3BP1/2+ condensates control insulin mRNA storage in beta cells
Source: EMBO J. 2025 May 12;44(13):3669–96. doi: 10.1038/s44318-025-00448-7 (PMC12216156; doi:10.1038/s44318-025-00448-7)
Supplement: Supplementary file 13 — Expanded View Figures [file 44318_2025_448_MOESM13_ESM.pdf]

Expanded View Figures

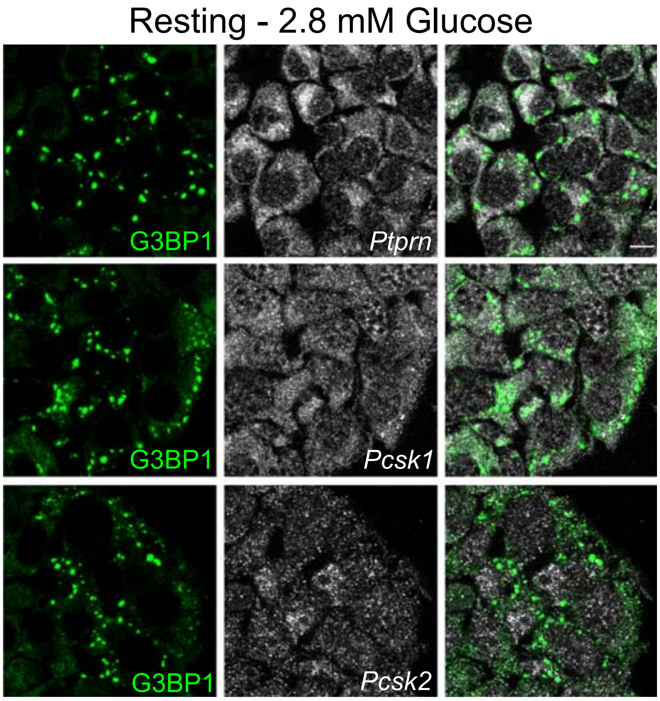

Figure EV1. mRNA detection of other insulin secretory granule cargoes.

(**A**) Immunostainings for G3BP1 (green) and smRNA FISH for *Ptprn*, *Pcsk1* and *Pcsk2* (gray) in MIN6-K8 cells in resting glucose conditions.

© The Author(s)

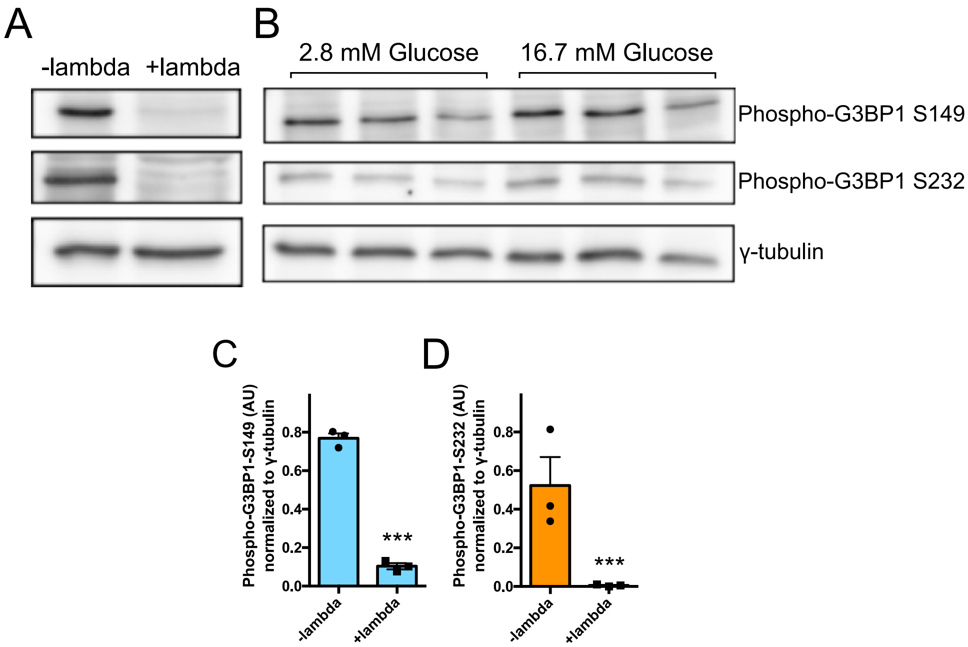

Figure EV2. Western blots for phospho-G3BP1 S149 and phospho-G3BP1 S232.

(**A**) Western blot for Phospho-G3BP1 S149, Phospho-G3BP1 S232 and gamma-tubulin on extracts of MIN6-K8 cells in growth media with 25 mM glucose and treated with or without lambda phosphatase. (**B**) Western blot for Phospho-G3BP1 S149, Phospho-G3BP1 S232 and gamma-tubulin on extracts of glucose resting or stimulated MIN6-K8 cells. (**C**, **D**) Quantification of the western blots shown in panel (**A**). (**C**, **D**): Every comparison $p < 0.0001$. Data information: Presented values denote the mean ± SD derived from three independent experiments, analyzed via paired *t*-test with Mann–Whitney correction. Values with $^{***}p < 0.001$ were considered statistically significant relative to the -lambda condition. The results for the western blot are from 1 technical replicate of each condition per independent experiment.

© The Author(s)

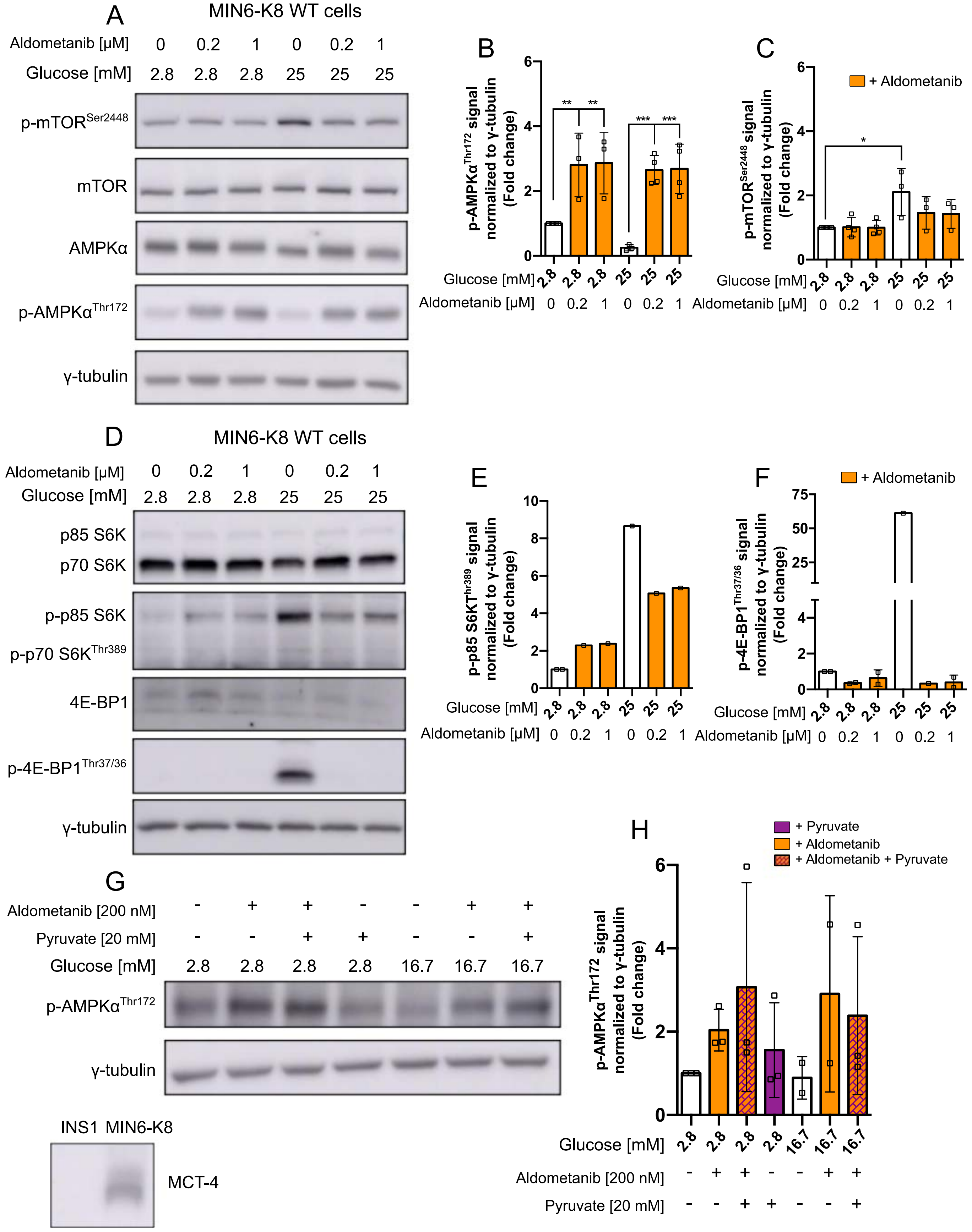
A
MIN6-K8 WT cells
Aldometanib [μM] 0 0.2 1 0 0.2 1
Glucose [mM] 2.8 2.8 2.8 25 25 25
p-mTORSer2448
mTOR
AMPKα
p-AMPKαThr172
γ-tubulin
B
p-AMPKαThr172 signal normalized to γ-tubulin (Fold change)
Glucose [mM] 2.8 2.8 2.8 25 25 25
Aldometanib [μM] 0 0.2 1 0 0.2 1
C
+ Aldometanib
p-mTORSer2448 signal normalized to γ-tubulin (Fold change)
Glucose [mM] 2.8 2.8 2.8 25 25 25
Aldometanib [μM] 0 0.2 1 0 0.2 1
D
MIN6-K8 WT cells
Aldometanib [μM] 0 0.2 1 0 0.2 1
Glucose [mM] 2.8 2.8 2.8 25 25 25
p85 S6K
p70 S6K
p-p85 S6K
p-p70 S6KThr389
4E-BP1
p-4E-BP1Thr37/36
γ-tubulin
E
p-p85 S6KThr389 signal normalized to γ-tubulin (Fold change)
Glucose [mM] 2.8 2.8 2.8 25 25 25
Aldometanib [μM] 0 0.2 1 0 0.2 1
F
+ Aldometanib
p-4E-BP1Thr37/36 signal normalized to γ-tubulin (Fold change)
Glucose [mM] 2.8 2.8 2.8 25 25 25
Aldometanib [μM] 0 0.2 1 0 0.2 1
G
Aldometanib [200 nM] - + + - - + +
Pyruvate [20 mM] - - + + - - +
Glucose [mM] 2.8 2.8 2.8 2.8 16.7 16.7 16.7
p-AMPKαThr172
γ-tubulin
INS1 MIN6-K8
MCT-4
H
+ Pyruvate
+ Aldometanib
+ Aldometanib + Pyruvate
p-AMPKαThr172 signal normalized to γ-tubulin (Fold change)
Glucose [mM] 2.8 2.8 2.8 2.8 16.7 16.7 16.7
Aldometanib [200 nM] - + + - - + +
Pyruvate [20 mM] - - + + - - +

 © The Author(s)

Figure EV3. Aldolase control of AMPKα and mTOR signaling in MIN6-K8 cells.

(**A**) Western blot (**A**) and quantifications (**B**) p-AMPKα^{Thr172}, (**C**) p-mTOR^{Ser2448} and gamma-tubulin across varying conditions: resting and stimulating glucose concentrations, with and without aldometanib treatment as shown in the figure. (**D**) Western blot (**D**) and quantifications of (**E**) p-p85 S6K^{Thr389}, (**F**) p-4E-BP1$^{Thr37/36}$ and gamma-tubulin across varying conditions: resting and stimulating glucose concentrations, with and without aldometanib treatment as shown in the figure. (**G**) Western blot (**G**) and quantifications (**H**) p-AMPKα^{Thr172} and gamma-tubulin across varying conditions: resting and stimulating glucose concentrations, with and without aldometanib treatment and/or pyruvate as shown in the figure. Data information: Presented values denote the mean ± SD derived from three independent experiments (**B**, **C**, **H**) or two independent experiments (**E**, **F**), analyzed via paired *t*-test with Mann–Whitney correction. Values with $^{**}p < 0.01$ and $^{***}p < 0.001$ were considered statistically significant relative to the conditions as shown in each graph. The results of the western blots are from 2 technical replicates of each condition per independent experiment. (**B**): 2.8 vs 2.8 + aldometanib 0.2 ($p = 0.0043$); 2.8 + aldometanib 1 ($p = 0.0035$), 25 vs 25 + aldometanib 0.2 ($p = 0.0003$); 25 + aldometanib 1 ($p = 0.0007$). (**C**): 2.8 vs 25 ($p = 0.0319$).

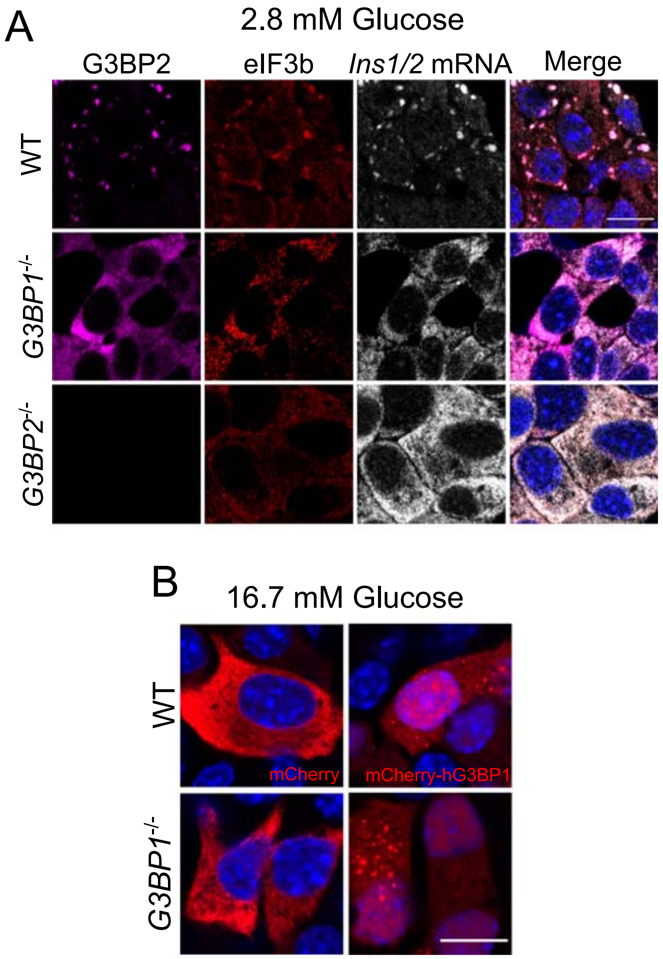

Figure EV4. Confocal microscopy of *G3BP1*$^{-/-}$, *G3BP2*$^{-/-}$ and wild-type MIN6-K8 cells.

(**A**) Immunostaining for G3BP2 (magenta), eIF3b (red) and smRNA FISH for *Ins1/2* in WT, *G3BP1*$^{-/-}$ and *G3BP2*$^{-/-}$ MIN6-K8 cells at resting glucose concentrations. Nuclei are stained with DAPI (blue). (**B**) Immunostaining of *G3BP1*$^{-/-}$ and wild-type MIN6-K8 cells transiently transfected with *mCherry* or *mCherry-hG3BP1* and exposed to high glucose levels. Signals for mCherry or mCherry-hG3BP1 are in red, as labeled in the image. Nuclei are stained with DAPI (blue). Data information: The presented results in (**A**) were derived from three independent experiments and at least 5 images per condition per experiment, and for (**B**) one independent experiment and 4 images of each condition. Scale bars = 10 μm.

 © The Author(s)

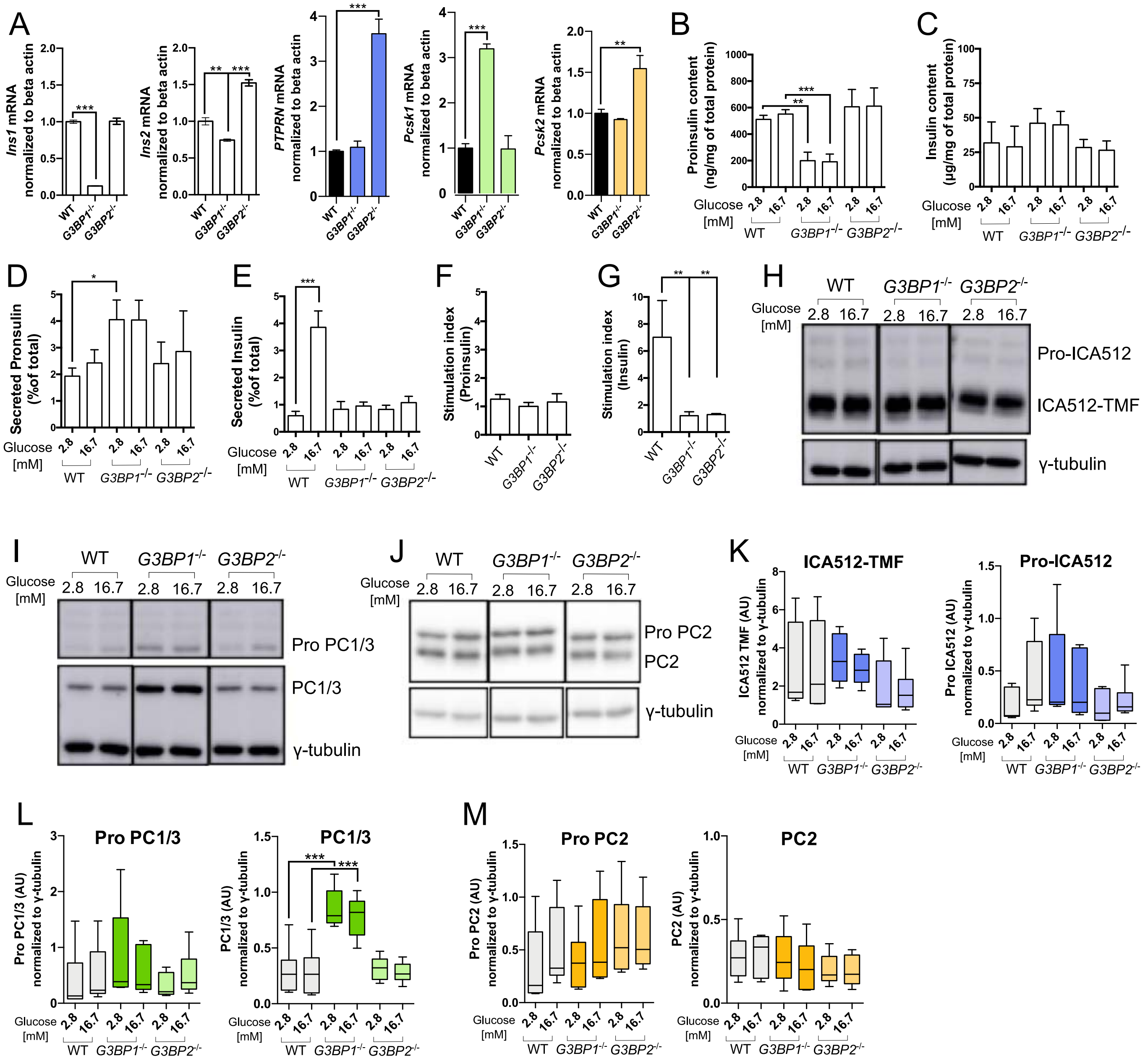

Figure EV5. mRNA and protein levels of insulin secretory granule cargoes in MIN6-K8 wild type, *G3BP1*$^{-/-}$ and *G3BP2*$^{-/-}$ cells.

(**A**) *Ins1*, *Ins2*, *Ptprn*, *Pcsk1*, and *Pcsk2* mRNA levels in WT, *G3BP1*$^{-/-}$ and *G3BP2*$^{-/-}$MIN6-K8 cells, as assessed by qRT-PCR. (**B–E**) Quantification of proinsulin and insulin levels and their secretion to the culture media as measured by ELISA and HTRF, respectively, in WT, *G3BP1*$^{-/-}$ and *G3BP2*$^{-/-}$ MIN6-K8 cells under resting and stimulating glucose concentrations. (**F**, **G**) Stimulation index for proinsulin or insulin, respectively, in WT, *G3BP1*$^{-/-}$ and *G3BP2*$^{-/-}$ MIN6-K8 cells. (**H–M**) Western blots and their respective quantifications for Pro-ICA512, ICA512 Transmembrane Fragment (ICA512-TMF), Pro-PC1/3, PC1/3, Pro-PC2 and PC2 species in glucose resting and stimulated WT, *G3BP1*$^{-/-}$ and *G3BP2*$^{-/-}$ MIN6-K8 cells. Data information: Presented values denote the mean ± SD derived from three independent experiments for qRT-PCR and five independent experiments for western blots, analyzed via one-way ANOVA. Values $^{**}p < 0.01$, and $^{***}p < 0.001$ were considered statistically significant relative to the WT condition. The results for the qRT-PCR are from 3 technical replicates and for western blot from 1 technical replicate of each condition per independent experiment. (**A**): *Ins1* graph WT vs *G3BP1*$^{-/-}$ ($p < 0.0001$), *Ins2* graph WT vs *G3BP1*$^{-/-}$ ($p = 0.0038$); *G3BP2*$^{-/-}$ ($p < 0.0001$), *PTPRN* graph WT vs *G3BP2*$^{-/-}$ ($p < 0.0001$), *Pcsk1* graph WT vs *G3BP1*$^{-/-}$ ($p < 0.0001$), *Pcsk2* graph WT vs *G3BP2*$^{-/-}$ ($p = 0.008$). (**B**): 2.8 WT vs 2.8 *G3BP1*$^{-/-}$ ($p = 0.0012$), 16.7 WT vs 16.7 *G3BP1*$^{-/-}$ (0.0009). (**D**): 2.8 WT vs 2.8 *G3BP1*$^{-/-}$ ($p = 0.029$). (**E**): 2.8 WT vs 16.7 WT ($p < 0.0001$). (**G**): WT vs *G3BP1*$^{-/-}$ ($p = 0.0041$); vs *G3BP2*$^{-/-}$ ($p = 0.0037$). (**L**): PC1/3 graph 2.8 WT vs 2.8 *G3BP1*$^{-/-}$ ($p < 0.0001$), 16.7 WT vs 16.7 *G3BP1*$^{-/-}$ ($p < 0.0001$).

© The Author(s)

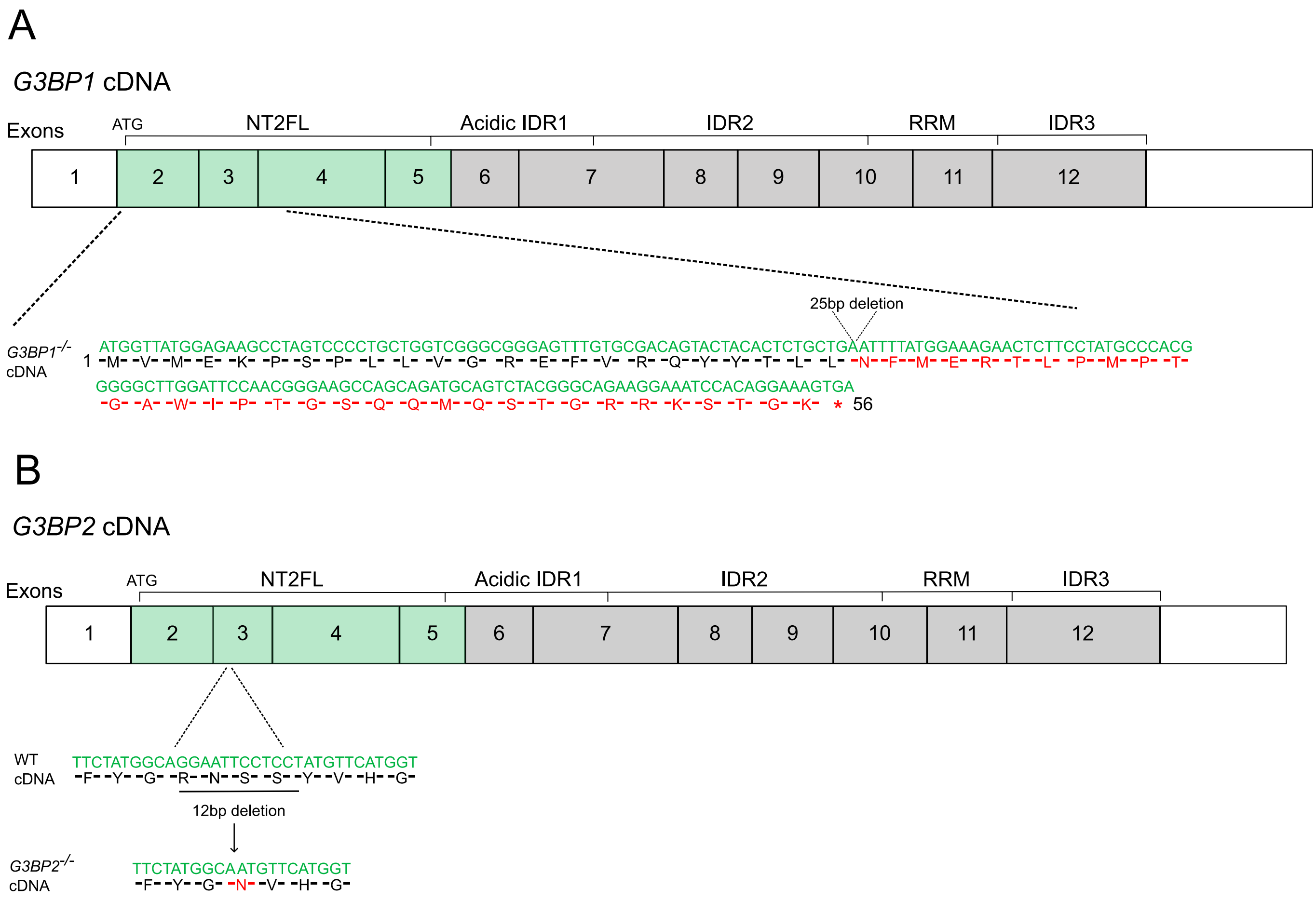

Figure EV6. Characterization of *G3BP1* and *G3BP2* deletions in *G3BP1*$^{-/-}$ and *G3BP2*$^{-/-}$ MIN6-K8 clones.

(**A**, **B**) Schematic illustrations of the G3BP1 (**A**) and G3BP2 (**B**) domain and exon structures. The exons coding for the NTF2L domain, which is responsible for G3BP1 and G3BP2 dimerization are colored in green. The location of the nucleotide deletions identified in *G3BP1*$^{-/-}$ and *G3BP2*$^{-/-}$ MIN6-K8 clones and the alterations in the corresponding amino sequences are shown. The deletion in *G3BP1* introduces a premature stop codon. The deletion in *G3BP2* removes four amino acids and converts Y40 into N.

© The Author(s)
